# Effectiveness of obstetric point-of-care ultrasound (POCUS) training: a systematic review and meta-analysis based on the *ADDIE* training model

**DOI:** 10.1186/s13089-025-00471-z

**Published:** 2025-12-31

**Authors:** Yuxuan Li, Hao Wang, Kang Yuan, Xiaoding Zhou, Mengting Wang, Yiyang Huang, Xinyi Yu, Kun Tang

**Affiliations:** 1 Vanke School of Public Health, Tsinghua University, Beijing, China; 2 School of Basic Medical Sciences, Tsinghua University, Beijing, China; 3 Bloomberg School of Public Health, Johns Hopkins University, Baltimore, MD USA; 4 School of Public Health, Peking University, Beijing, China; 5 School of Nursing, Peking Union Medical College, Beijing, China; 6 School of Health Economics and Management, Nanjing University of Chinese Medicine, Nanjing, China; 7 School of Public Health, Capital Medical University, Beijing, China

**Keywords:** Point-of-care systems, Ultrasonography, Prenatal, Education, Medical, Developing countries, Systematic review

## Abstract

**Aim:**

To identify the core components of obstetric point-of-care ultrasound (POCUS) training programs while simultaneously evaluating the effectiveness of these programs using the *Analyze*, *Design*, *Develop*, *Implement*, and *Evaluate* (*ADDIE*) model.

**Methods:**

This systematic review and meta-analysis followed a *PROSPERO*-registered protocol (CRD42024566260) and adhered to *PRISMA2020*, *Cochrane Handbook*, and *JBI Manual* guidelines. Comprehensive searches from database inception to September 22, 2024, covered international and Chinese databases to identify studies evaluating obstetric POCUS training. Two independent reviewers screened studies, assessed methodological quality with *JBI* tools, and extracted data on study, participant, intervention, and outcome characteristics. Training content was mapped to the *ADDIE* instructional design model via thematic and framework analyses. Meta-analyses of comparable quantitative outcomes used random-effects models. Integrating quantitative and qualitative findings, this review systematically evaluated the effectiveness and implementation of obstetric POCUS training programs.

**Results:**

Systematic synthesis showed that obstetric POCUS training significantly improved healthcare providers’ competencies, including knowledge, skills, sustained use, and clinical decision-making. Training also increased antenatal care attendance and identification of high-risk pregnancies, while reducing referrals and optimizing resource use. However, limitations were noted in needs assessment, implementation flexibility, and outcome evaluation. Using thematic and framework analyses combined with the *ADDIE* model, we systematically organized training phases and key components to provide a scientific basis for program improvement and optimization.

**Conclusions:**

Obstetric POCUS training effectively enhances clinical competencies and improves maternal and neonatal health outcomes. Applying the *ADDIE* model offers a replicable, practical, and sustainable approach for developing standardized training programs. Future obstetric POCUS training should leverage the *ADDIE* model and adapt to local contexts to improve maternal and neonatal health globally.

**Supplementary Information:**

The online version contains supplementary material available at 10.1186/s13089-025-00471-z.

## Introduction

Point-of-care ultrasound (POCUS), originally developed for emergency settings, has become increasingly valuable in obstetric care. It enables real-time, radiation-free imaging at the bedside, supporting critical assessments such as fetal position, gestational age, placental location, and amniotic fluid volume [1, 2]. As its use expands beyond radiologists to include midwives, nurses, and generalist physicians, training non-specialist healthcare providers has become essential [3, 4]. However, current training programs range from short courses to mentorship or lectures, often lacking clear objectives or evaluation criteria. This inconsistency raises a key question: how can POCUS training be systematically designed to ensure clinical competence?

This question is particularly critical in low- and middle-income countries (LMICs), where maternal mortality remains disproportionately high, accounting for over 90% of global maternal deaths [5]. In *sub-Saharan Africa*, the lifetime risk of maternal death is 1 in 55, over 30 times higher than in most high-income countries (HICs) [5]. Many of these deaths are preventable and stem from delays in recognizing complications such as dystocia or placenta previa. Portable ultrasound devices are now more affordable and accessible, but frontline providers often lack the training needed to use them effectively [6]. With proper training, non-specialist healthcare workers can detect high-risk pregnancies, avoid unnecessary referrals, and better manage obstetric emergencies [7, 8]. In rural *Uganda*, midwives trained in POCUS outperformed routine examinations in detecting twin pregnancies and breech presentations [3].

Despite the growing number of obstetric POCUS training programs, their design and implementation vary widely. Some are developed without assessing local learning needs or defining clear educational goals and competency standards [7, 8]. Training curricula vary widely—from self-directed modules to workshops—often lacking justification for their design [9]. Implementation also differs: some programs include supervised practice and feedback, while others rely solely on lectures [10]. When it comes to evaluation, studies often focus on short-term outcomes, such as quiz scores or observed skills, with limited attention to retention or clinical application [11, 12]. These inconsistencies highlight the lack of a unified framework to guide training development, hindering both within-study interpretation and cross-study comparison.

These limitations in original studies have prompted five reviews to synthesize existing training efforts. While these reviews offer valuable summaries, many stop at describing instructional formats or cataloging skills, with limited attention to how program components relate to health outcomes [13, 14]. Some highlight strengths like simulation fidelity [15], but few assess clinical endpoints. For example, among 27 studies reviewed by Bidner et al. [12], only 6 reported maternal or neonatal health outcomes. This lack of a design-to-outcome framework leaves two critical questions insufficiently addressed: (1) Which components are essential for effective POCUS training? (2) What criteria should be used to measure effectiveness?

To resolve these critical questions, we applied the *ADDIE* model—*Analyze*, *Design*, *Develop*, *Implement*, and *Evaluate*—as an organizing framework to examine obstetric POCUS training programs [16, 17]. By mapping existing training programs onto the five phases, we could assess their internal coherence and identify key gaps. Viewed sequentially, the *ADDIE* model links upstream planning decisions with downstream outcomes. In reverse, the model also allows retrospective analysis of training failures by identifying which phase may have broken down. Thus, the *ADDIE* model serves as both a development framework and an evaluation tool.

This systematic review aims to identify key components and assess the effectiveness of obstetric POCUS training programs using the *ADDIE* model. It focuses on studies targeting midwives, nurses, generalist physicians, and clinical educators across both high- and low-resource settings. Outcomes are examined in three domains: (1) Training effectiveness—including knowledge, skills, diagnostic accuracy, retention, and application; (2) Maternal and neonatal health outcomes; and (3) Health economic outcomes. By organizing existing evidence through the *ADDIE* model, this review offers practical insights into what works, why it works, and under which conditions it is most effective. The findings are intended to support educators, implementers, and policymakers in designing more coherent, context-appropriate training programs that contribute to safer, more equitable global maternal care.

## Methods

A systematic review and meta-analysis was conducted to comprehensively identify key components and assess the effectiveness of obstetric POCUS training programs according to the *ADDIE* model. The review protocol followed the methodological guidelines outlined in the *Cochrane Handbook for Systematic Reviews of Interventions* [18] and the *Joanna Briggs Institute (JBI) Manual for Evidence Synthesis (2024)* [19], and was reported according to the *Preferred Reporting Items for Systematic Reviews and Meta-Analyses 2020 (PRISMA2020) Statement* [20] (Supplementary Table 1). The protocol was prospectively registered in *PROSPERO* (CRD42024566260).

### Search strategy

Following the *JBI* three-step search method [19], the literature search began with an initial search in *PubMed* and *SinoMed* to identify potentially relevant studies and analyze text words from titles, abstracts, keywords, and index terms. Based on this preliminary analysis, a comprehensive search was conducted across international databases (*PubMed*, *Embase*, *CINAHL Plus*, *Web of Science*, and *Scopus*), Chinese databases (*CNKI*, *WanFang*, *CQVIP*, and *SinoMed*), clinical trial registries (*WHO ICTRP*, *CENTRAL*, and *ClinicalTrials.gov*), and grey literature sources (*ProQuest Dissertations*, *CNKI Dissertations*, and *WanFang Dissertations*). The final step involved supplementary searching through *Google Scholar* and a snowballing approach, screening reference lists of included studies to identify potentially relevant literature.

All databases were searched from inception to September 22, 2024. Search strategies combined controlled vocabulary (e.g., MeSH terms) with free-text terms, adapted for each database’s specific characteristics. Search terms were organized around three main concepts, including “obstetric”-related terms, “point-of-care ultrasound”-related terms, and “training or education”-related terms, and were combined using *Boolean* operators “AND” and “OR” to enhance search precision. To ensure comprehensiveness, no restrictions on language or study design were imposed at this stage. Detailed search strategies for each database are provided in Supplementary Table 2.

### Eligibility criteria and study selection

The PIOS (P = Population, I = Intervention, O = Outcomes, S = Study Design) framework was used to formulate the research questions and develop inclusion and exclusion criteria (Table 1). We omitted a “comparison” criterion as our primary aims were to assess the implementation process and effectiveness of obstetric POCUS training programs. This focus on training outcomes such as knowledge enhancement, skill improvement, and influence on patient care provides a comprehensive evaluation without requiring standardized comparisons across studies.

**Table 1:** Inclusion and exclusion criteria using PIOS format

Criteria	Inclusion	Exclusion
Population	Healthcare providers (doctors, nurses, midwives, clinical staff) or medical students involved in obstetric care, at any experience level	Certified sonographers, ultrasound specialists, temporary workers in the healthcare facilities, and participants concurrently enrolled in other training programs
Intervention	Training programs involving obstetric POCUS devices	Training programs lacking identifiable *ADDIE* model components
Outcomes	Training effectiveness (knowledge, skills, confidence), clinical practice changes, patient health outcomes, economic impacts	Studies reporting other content (e.g., disease diagnosis) without training outcomes, studies without extractable quantitative data
Study design	Randomized controlled trials (RCTs), quasi-experimental studies, cross-sectional studies, cohort studies, case–control studies	Reviews, abstracts, editorials, case reports, non-peer-reviewed studies
Language	All languages	Language other than English or Chinese

Following comprehensive database searches, all records were imported into *EndNoteX9* for deduplication, then transferred to the *Covidence* systematic review platform for study screening. Two independent reviewers screened titles and abstracts to identify potentially relevant studies, followed by full-text assessment according to the eligibility criteria. Disagreements were resolved through discussion, with a third reviewer consulted when consensus could not be reached. Reasons for exclusion at the full-text screen stage were systematically recorded and categorized.

### Quality assessment

The *JBI Critical Appraisal Checklists* for quasi-experimental studies (9 items), cohort studies (11 items), and cross-sectional studies (8 items) were used to assess the methodological quality of included studies (*n* = 27) [21]. These quality evaluation tools systematically evaluate key dimensions, including appropriateness of study design, reliability of measurement methods, adequacy of statistical analysis, and control of potential biases. Each checklist item was rated as “Yes,” “No,” “Unclear,” or “Not Applicable,” with overall assessment resulting in “Include,” “Exclude,” or “Seek further information.” Studies with a “Yes” rating of 50% or above were considered acceptable quality, a predetermined threshold established by two independent researchers [22, 23]. Quality assessment was conducted back-to-back by two reviewers following a pilot assessment to ensure consistency. Disagreements were resolved through discussion or third-party arbitration when consensus could not be reached.

### Data extraction

Data extraction was conducted using a standardized form adapted from the *JBI Qualitative Data Extraction Tool* [19]. A preliminary form (Supplementary File 1) was piloted on five included studies to evaluate its comprehensiveness and applicability. Based on the pilot testing, adjustments were made to streamline the extraction process and improve focus, including the removal of seven items. These comprised related certifications (Item 2.6), the subdivisions of maternal and neonatal health outcomes (Items 4.2.1–4.2.3), and the detailed health economic analyses categories (Items 4.3.1–4.3.3). The final data extraction template is available in Supplementary File 2.

Guided by the PIOS framework, the extraction process focused on four key domains: (1) Study characteristics, including first author and publication year, country, setting, study design, and sample size; (2) Participant characteristics, documenting healthcare providers’ age, gender, professional role, years of experience, previous ultrasound training, and relevant certifications; (3) Intervention characteristics, systematically extracting specific content across the five *ADDIE* model phases (*Analysis*, *Design*, *Development*, *Implementation*, and *Evaluation*); and (4) Outcome characteristics, including training effectiveness (diagnostic accuracy, knowledge acquisition, skill mastery, knowledge retention, clinical application), maternal-neonatal health outcomes (maternal health outcomes, neonatal health outcomes, maternal satisfaction), and health economic indicators (cost-effectiveness analysis, cost-utility analysis, cost–benefit analysis). Data extraction was independently conducted by two reviewers, with all data entered into an *Excel* database and double-checked. Pilot extraction ensured consistency before formal extraction. Disagreements during extraction were resolved through discussion or by referring back to the original text, with a senior team member verifying accuracy and consistency.

### Statistical analysis

A narrative synthesis and meta-analysis approach was employed for data analysis. Narrative synthesis was conducted using both thematic analysis and framework-based analysis. Thematic analysis is a method for identifying and reporting patterns through systematic coding [24, 25], which helped identify recurring themes in training implementation and outcomes across studies. Framework-based analysis applies an existing conceptual framework to organize and interpret data [26]. In our study, the *ADDIE* model was used as the analytical framework to systematically categorize training content into five instructional design phases. Through combining these two methods, study characteristics, implementation of *ADDIE* model components, outcome measurement methods, and training effects were comprehensively synthesized. Based on these synthesis findings, meta-analysis was performed for outcomes where three or more studies reported comparable data within the *ADDIE* model ‘*Evaluation*’ phase.

Meta-analyses utilized *restricted maximum likelihood* (*REML*) random-effects models to pool effect sizes, with confidence intervals (*CIs*) adjusted using the *Hartung–Knapp–Sidik–Jonkman* (*HKSJ*) method to optimize robustness in small sample analyses. Different approaches were applied based on data type. For continuous data (e.g., pre-post mean changes), effect sizes were calculated as standardized mean differences (SMD) with *Hedges’ g* correction applied to eliminate small sample bias. For single-group proportions (e.g., diagnostic accuracy, training pass rates), the *Freeman–Tukey* double arcsine transformation was used to stabilize variance before calculating raw effect sizes [27]. Heterogeneity was assessed using *Cochran’s Q* test and quantified using *Higgins’ I*^2^ statistic, with subgroup and sensitivity analyses conducted to explore sources of heterogeneity when *I*^2^ > 50%. All statistical analyses were performed in *STATA18.0* (StataCorp LLC, Texas, USA), with statistical significance defined as a two-tailed *p* value < 0.05.

## Results

### Study selection and quality assessment results

**Fig. 1:**
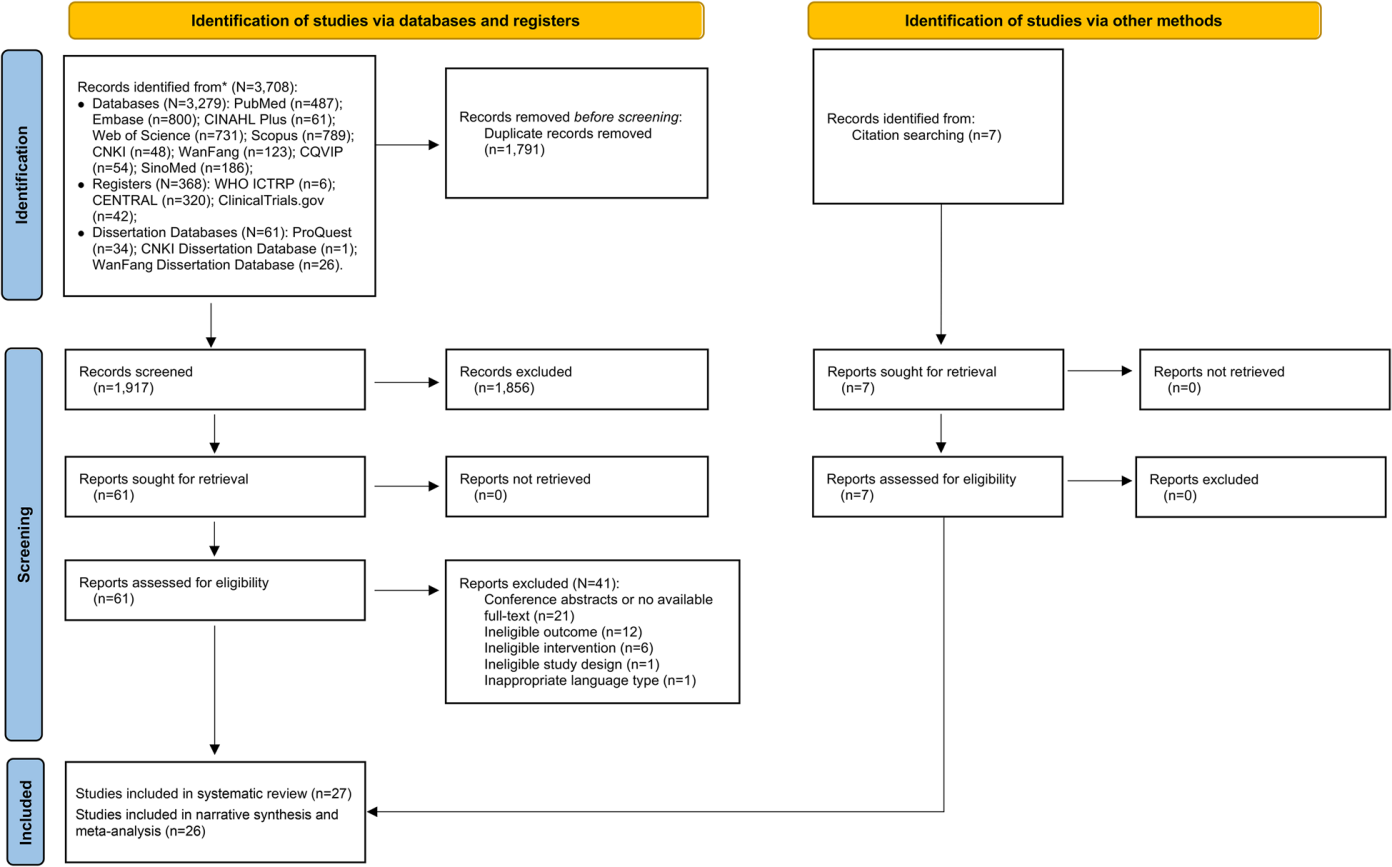
PRISMA 2020 flow diagram for new systematic reviews which included searches of databases, registers and other sources. PubMed (National Library of Medicine’s Database), Embase (Excerpta Medica Database), CINAHL Plus (Cumulative Index to Nursing and Allied Health Literature plus), Web of Science (Web of Science Core Collection Database), Scopus (Elsevier’s Abstract and Citation Database), CNKI (China National Knowledge Infrastructure), WanFang (WanFang Data Knowledge Service Platform), CQVIP (Chinese Scientific Journals Database), SinoMed (Chinese Biomedical Literature Database),WHO ICTRP (World Health Organization International Clinical Trials Registry Platform), Cochrane Central Database (Cochrane Central Register of Controlled Trials (CENTRAL)), ClinicalTrials.gov (ClinicalTrials.gov database), CNKI Dissertation Database (China Doctoral Dissertations & Master’s Theses Full-text Database). *Source*: Page et al. [20]

The systematic literature search yielded 3708 records from multiple databases. After removing duplicates and screening titles and abstracts, 61 articles remained for full-text assessment. During full-text review, 41 articles were excluded due to various reasons, including unavailable full-texts, ineligible outcomes, interventions, study designs, or language restrictions. Seven additional reports were identified through citation searching, all of which met the inclusion criteria, resulting in a total of 27 included studies (Supplementary Files 3–5). The *PRISMA2020* flowchart (Fig. 1) illustrates the search and selection process. Quality assessment using *JBI Critical Appraisal Tools* demonstrated satisfactory methodological rigor across studies. One study [28] was excluded following quality assessment due to inadequate methodological quality, leaving 26 studies for narrative synthesis and meta-analysis within the *ADDIE* model (Table 2; Supplementary Table 3).

**Table 2:** Quality evaluation results

No.	Included study	Q1	Q2	Q3	Q4	Q5	Q6	Q7	Q8	Q9	Overall appraisal
Study design I: Quasi-experimental study (*n* = 10)											
1	Cook et al. [29]	Y	N	Y	Y	Y	Y	Y	N/A	Y	Include
2	Davila Roman et al. [30]	Y	N	Y	Y	Y	Y	Y	N/A	Y	Include
3	Filler and Lettang [31]	Y	N	Y	Y	Y	Y	Y	N/A	Y	Include
4	Hall et al. [8]	Y	N	Y	Y	Y	Y	Y	N/A	Y	Include
5	Kimberly et al. [32]	Y	N	Y	Y	Y	Y	Y	Y	Y	Include
6	Kolbe et al. [33]	Y	N	Y	Y	Y	Y	Y	N/A	Y	Include
7	Lee et al. [34]	Y	N	Y	Y	Y	Y	Y	N/A	Y	Include
8	Vinayak and Sharon Brownie [35]	Y	N	Y	Y	Y	Y	Y	N/A	Y	Include
9	Wachira et al. [36]	Y	N	Y	Y	Y	Y	Y	N/A	Y	Include
10	Ward et al. [28]	N	N	Y	Y	N	Y	N	N/A	N	Exclude

No.	Included Study	Q1	Q2	Q3	Q4	Q5	Q6	Q7	Q8	Q9	Q10	Q11	Overall Appraisal
Study design II: Cohort study (*n* = 11)													
11	Bentley et al. [37]	N/A	N/A	Y	N	N	Y	Y	Y	N	N	Y	Include
12	Bidner et al. [12]	N/A	N/A	Y	Y	Y	Y	Y	Y	N	N	Y	Include
13	Erlick et al. [38]	N/A	N/A	Y	Y	N	Y	Y	Y	Y	Y	Y	Include
14	Greenwold et al. [39]	Y	Y	Y	Y	N	Y	Y	Y	Y	N	Y	Include
15	Henwood et al. [40]	N/A	N/A	Y	Y	N	Y	Y	Y	Y	N	Y	Include
16	Kotagal et al. [41]	N/A	N/A	Y	Y	N	Y	Y	Y	Y	N	Y	Include
17	Miles et al. [42]	Y	Y	Y	Y	N	Y	Y	Y	Y	N	Y	Include
18	Rominger et al. [43]	Y	N/A	Y	Y	N	Y	Y	Y	Y	N	Y	Include
19	Shah et al. [3]	Y	Y	Y	Y	Y	Y	Y	Y	Y	N	Y	Include
20	Varner et al. [44]	N/A	N/A	Y	Y	Y	Y	Y	Y	Y	N	Y	Include
21	Westerway et al. [45]	Y	N/A	Y	N	Y	Y	Y	Y	N	N	Y	Include

No	Included Study	Q1	Q2	Q3	Q4	Q5	Q6	Q7	Q8	Overall Appraisal
Study design III: Cross-sectional study (*n* = 6)										
22	Lee et al. [46]	Y	Y	Y	Y	Y	N	Y	Y	Include
23	Nathan et al. [45]	Y	Y	Y	Y	Y	N	Y	Y	Include
24	Shah et al. [47]	Y	Y	Y	Y	N	N	Y	Y	Include
25	Shokoohi et al. [4]	Y	Y	Y	Y	Y	N	Y	Y	Include
26	Vinayak et al. [48]	Y	Y	Y	Y	N	Y	Y	Y	Include
27	Wanjiku et al. [49]	Y	Y	Y	Y	N	Y	Y	Y	Include

Answers to questions: yes (Y), no (N), not applicable (N/A), or unclear

Answers to overall appraisal: include, exclude, or seek further information

### Basic characteristics of included studies

Table 3 presents the characteristics of the 27 included studies, published between 2009 and 2024. Geographically, *Africa* contributed the most studies (*n* = 15, 55.6%), followed by the *Americas* (*n* = 11, 40.7%), *Asia* (*n* = 5, 18.5%), and *Oceania* (*n* = 2, 7.4%), with 4 studies (14.8%) involving multiple regions. Training settings ranged from university medical schools to rural health centers, with 8 studies (29.6%) conducted in rural settings. Study designs comprised cohort studies (*n* = 11, 40.7%), quasi-experimental studies (*n* = 10, 37.0%), and cross-sectional studies (*n* = 6, 22.2%). Sample sizes ranged from 3 to 514, with 24 studies (88.9%) reporting specific numbers. Participants included physicians, midwives, nurses, medical students, clinical staff, and imaging technicians. Among demographic characteristics, 2 studies (7.4%) provided age data (mean 27 years or range 18–26 years) and 5 studies (18.5%) reported gender distribution, showing female predominance except in medical student cohorts. Six studies (22.2%) documented work experience ranging from 1 to 40 years. Prior ultrasound training was reported in 19 studies (70.4%), with 18 studies (66.7%) involving participants who had no previous ultrasound experience.

**Table 3:** Summary of basic characteristics of included studies (*n* = 27)

No	1. Study characteristics					2. Population characteristics					3. Intervention characteristics					4. Outcome characteristics		
	1.1 Author (publication year)	1.2 Country	1.3 Location	1.4 Study design	1.5 Sample size	2.1 Age	2.2 Gender	2.3 Professional role	2.4 Work experience (duration)	2.5 Prior ultrasound training experience	3.1 Analysis	3.2 Design	3.3 Development	3.4 Implementation	3.5 Evaluation	4.1 Training Outcomes	4.2 Maternal and Neonatal Health Outcomes	4.3 Health Economic Outcomes
1	Cook et al. [29]	USA	University of South Carolina School of Medicine Columbia	Quasi-experimental study	76	Mean: 27	(1) Male: 57.9% (44/76) (2) Female: 42.1% (32/76)	Students: 100% (76/76)	NI	None	(1) Trainee groups: Students (2) Needs analysis: To improve understanding of obstetric and gynecological anatomy and pathology (individual level) (3) Baseline survey methods: Multiple-choice questions/exams (4) Impact on instructional design: NI	(1) Training objectives: Knowledge-related objectives, skill-related objectives and attitude-related objectives (2) Training methods Classroom sessions and practical skills training (including simulation) (3) Training content/plan/syllabus: Progressive structures, practice-oriented designs and clinical case integrations	(1) Instructors ① Academic instructors: University faculty (faculty members/researchers) ② Student-to-instructor ratio: Scalable instruction (2) Teaching aids ① POCUS (3) Teaching materials: NI	(1) Time ① Training duration: Short-term trainings (1–2 weeks) ② Follow-up timing after training: NI ③ Follow-up duration: 10 weeks (2) Location/setting ① Training sites: Educational institutions ② Practice Sites: Simulation laboratories (3) Participants ① Trainer: Academic faculty members ② Trainee: Students (4) Execution process ① Practical skills training phase: Self-directed scanning practice ② Assessment and feedback phase: Written knowledge assessments (5) Adaptation Records: NI	(1) Formative evaluation: Immediate feedback during training (2) Summative evaluation: Post-training multiple-choice questions test on knowledge gain (3) Follow-up: Survey on comfort and training experience	(1) Knowledge acquisition (2) Practical application	NI	NI
2	Dávila-Román et al. [30]	Guatemala, Peru, India and Rwanda	(1) Lead site: Washington University (2) Field sites: Guatemala (Guatemala City), Peru, India (Chennai, Tamil Nadu) and Rwanda	Quasi-experimental study	18	NI	NI	Sonographers: 100% (18/18)	NI	(1) All participants had prior experience in ultrasound or healthcare (2) Completed introductory online training before the program	(1) Trainee groups: Medical imaging technologists (2) Needs analysis: To reduce inter-individual imaging variability in lung and vascular biomarker measurements within the Household Air Pollution Intervention Network (HAPIN) study (Individual level) (3) Baseline Survey Methods: NI (4) Impact on Instructional Design: NI	(1) Training objectives: Skill-related objectives (2) Training methods: Self-study modules, remote feedback and supervision (3) Training content/plan/syllabus: Modular contents	(1) Instructors ① Academic instructors: University faculty (faculty members/researchers) ② Student-to-instructor ratio: Scalable instruction (2) Teaching aids ① POCUS (3) Teaching materials: Powerpoint presentations (PPTs) and developed ultrasound guidebooks based on training design	(1) Time ① Training duration: Short-term trainings (1–2 weeks) ② Follow-up timing after training: Immediate follow-up post-training ③ Follow-up duration: NI (2) Location/setting ① Training sites: Educational institutions ② Practice sites: Local hospitals or clinics and healthcare facilities in other low-resource countries (3) Participants ① Trainer: Academic faculty members ② Trainee: Sonographers (in-service medical and nursing personnel) (4) Execution process ① Theoretical instruction phase: Lectures on ultrasound techniques ② Practical skills training phase: Trainer-guided hands-on ultrasound practice ③ Assessment and feedback phase: Performance-based skill assessments ④ Post-training support phase: Participation in refresher or continuing O15 courses, ongoing feedback and quality assurance measures (5) Adaptation Records ① Adaptations based on learner-specific differences: Adjustment based on learning pace	(1) Formative evaluation: Ongoing tests and hands-on checks during training; scores improved from 60 to 84%; extra support for low performers (2) Summative Evaluation: Final tests and certified cases; all trainees gave feedback (100% response, avg. 4.9/5) (3) Follow-up: Immediate follow-up post-training. Ongoing quality control via cloud review and retraining; ensured consistent standards post-training	(1) Knowledge acquisition (2) Skills acquisition	NI	NI
3	Filler and Lettang [31]	USA	Creighton University School of Medicine Phoenix Program, Valleywise Health Medical Center	Quasi-experimental study	32	NI	NI	Doctors: 100% (32/32)	NI	NI	(1) Trainee Groups: Doctors (2) Needs Analysis: To improve the emergency use of transvaginal ultrasound (TVUS) for evaluating first-trimester pregnancy complications and non-obstetric abdominal pain, with a focus on developing skills in image acquisition, interpretation, and probe handling (Individual level) (3) Baseline Survey Methods: NI (4) Impact on Instructional Design: NI	(1) Training Objectives: Knowledge-related objectives, skill-related objectives and attitude-related objectives (2) Training Methods: Classroom sessions (3) Training Content/Plan/Syllabus: Progressive structures and certification modules	(1) Instructors ① Academic Instructors: Certified experts or lecturers in related fields ② Student-to-Instructor Ratio: Scalable instruction (2) Teaching Aids ① POCUS ② Self-learning platforms and high-fidelity simulation tools (3) Teaching Materials: Powerpoint presentations (PPTs) and course plans/syllabi	(1) Time ① Training Duration: Short-term trainings (1–2 weeks) ② Follow-up Timing After Training: Immediate follow-up post-training ③ Follow-up Duration: NI (2) Location/Setting ① Training Sites: Healthcare institutions ② Practice Sites: Local hospitals or clinics (3) Participants ① Trainer: Academic faculty members ② Trainee: Interns and residents (In-service medical and nursing personnel) (4) Execution Process ① Theoretical Instruction Phase: Self-directed scanning practice ② Practical Skills Training Phase: Trainer-guided hands-on ultrasound practice, case-based scenario applications and discussions ③ Assessment and Feedback Phase: Performance-based skill assessments and self-confidence evaluations (5) Adaptation Records ① Adaptations Based on Training Content and Delivery Methods: Training content adjustment	(1) Formative Evaluation: Real-time observation and feedback showed improvement in probe use and image reading (2) Summative Evaluation: Pre/post surveys showed confidence gains—post-graduate year-1: 1–4; post-graduate year-2/3: 3–4 (3) Follow-Up: Immediate follow-up post-training. Faculty follow-up confirmed increased real-world use of TVPOCUS	(1) Practical Application	NI	NI
4	Hall et al. [8]	Tanzania	Chake Chake District Hospital, Wete District Hospital and Micheweni Public Health Care Center (PHCC)	Quasi-experimental study	13	NI	(1) Male: 30.8% (4/13) (2) Female: 69.2% (9/13)	(1) Midwives/Nurse: 61.5% (8/13) (2) Clinical officer: 23.1% (3/13) (3) Medical officer/Physician: 15.4% (2/13)	NI	(1) None: 84.6% (11/13) (2) Informal experience: 7.7% (1/13) (3) Formal education: 7.7% (1/13)	(1) Trainee Groups: Midwives, nurses, clinical staff and doctors (2) Needs Analysis: To address a key maternal health need identified by the PURE project by integrating antenatal ultrasound into primary health care units (PHCUs) and district hospitals to improve maternal care delivery (System level)	(1) Training Objectives Knowledge-related objectives and skill-related objectives (2) Training Methods Classroom sessions, clinical mentoring/rotations and practical skills training (including simulation)	(1) Instructors ① Clinical Instructors: Emergency physician, obstetricians and gynecologists ② Student-to-Instructor Ratio: Scalable instruction (2) Teaching Aids	(1) Time ① Training Duration: Long-term trainings (≥ 12 weeks) ② Follow-up Timing After Training: Immediate follow-up post-training ③ Follow-up Duration: 3–6 Months (2) Location/Setting ① Training Sites: Healthcare institutions ② Practice Sites: Local hospitals or clinics	(1) Formative Evaluation Mid-course Objective Structured Clinical Examination (OSCE) avg: 71.2%; final OSCE avg: 84.7% —showing clear improvement (2) Summative Evaluation	(1) Knowledge Acquisition (2) Skills Acquisition (3) Knowledge Retention (4) Practical Application	NI	NI
											(3) Baseline Survey Methods: Trainees were selected by the Ministry of Health based on hospital and community needs, individual interest, and English proficiency, serving as a form of pre-training screening (4) Impact on Instructional Design: Hierarchical structuring of instructional content	(3) Training Content/Plan/Syllabus Progressive structures, modular contents, practice-oriented designs, clinical case integrations and certification modules	① POCUS (3) Teaching Materials Lecture handouts, powerpoint presentations (PPTs) and course plans/syllabi	(3) Participants ① Trainer: Healthcare professionals ② Trainee: Midwives and clinicians (In-service medical and nursing personnel) (4) Execution Process ① Practical Skills Training Phase: Trainer-guided hands-on ultrasound practice and case-based scenario applications and discussions ② Assessment and Feedback Phase: Written knowledge assessments and practical skills examinations ③ Post-training Support Phase: Ongoing feedback and quality assurance measures (5) Adaptation Records ① Adaptations Based on Learner-specific Differences: Adjustment based on learner feedback	62% (8/13) met all requirements; final exam avg: 77.5%; more scans linked to better OSCE scores (3) Follow-up Immediate follow-up post-training. Ongoing assessment planned; “Train-the-Trainer” model proposed; further research needed on clinical impact			
5	Kimberly et al. [32]	Zambia	Kapiri District Hospital, Mukonchi Rural Health Center and Nkole Rural Health Center	Quasi-experimental study	21	NI	NI	Midwives: 100% (21/21)	NI	None	(1) Trainee Groups: Midwives (2) Needs Analysis: To reduce maternal mortality and increase facility-based births by promoting ultrasound as part of a national strategy to improve maternal care and achieve global health targets (National level) (3) Baseline Survey Methods: NI (4) Impact on Instructional Design: NI	(1) Training Objectives Skill-related objectives (2) Training Methods: Classroom sessions, clinical mentoring/rotations and practical skills training (including simulation) (3) Training Content/Plan/Syllabus: Progressive structures, modular contents, practice-oriented designs and clinical case integrations	(1) Instructors ① Clinical Instructors: Ultrasonologists ② Student-to-Instructor Ratio: Scalable instruction (2) Teaching Aids ① POCUS (3) Teaching Materials: Lecture handouts, powerpoint presentations (PPTs), course plans/syllabi and scan data sheets	(1) Time ① Training Duration: Long-term trainings (≥ 12 weeks) ② Follow-up Timing After Training: NI ③ Follow-up Duration: > 6 Months (2) Location/Setting ① Training Sites: Healthcare institutions ② Practice Sites: Local hospitals or clinics	(1) Formative Evaluation: Objective Structured Clinical Examination (OSCE) scores improved from 71% (2 months) to 83% (6 months); common difficulties included battery changes, data entry, and using presets (2) Summative Evaluation: 17% of scans impacted care (e.g., repeat scans, more visits, referrals); 1-year follow-up showed ongoing use (~ 10 scans/week) and 100% felt ultrasound changed their practice (3) Follow-up: At 1 year, main issues were time limits (46%), equipment problems (38%), and rising patient demand	(1) Diagnostic Accuracy (2) Skills Acquisition (3) Knowledge Retention (4) Practical Application	(1) Maternity Attendance Rate (2) Psychological Outcomes	NI
														(3) Participants ① Trainer: Healthcare professionals ② Trainee: Midwives (In-service medical and nursing personnel) (4) Execution Process ① Practical Skills Training Phase: Trainer-guided hands-on ultrasound practice and self-directed scanning practice ② Assessment and Feedback Phase: Practical skills examinations (5) Adaptation Records ① Adaptations in Response to External Factors: Equipment adjustment				
6	Kolbe et al. [33]	Nicaragua	Las Salinas, a small rural village in western Nicaragua	Quasi-experimental study	4	NI	NI	(1) Doctors: 50% (2/4) (2) Nurses: 25% (1/4) (3) Nursing assistants: 25% (1/4)	NI	NI	(1) Trainee Groups: Doctors, nurses and nursing assistants (2) Needs Analysis: To address the lack of local imaging and limited access to referral hospitals by training providers with no prior ultrasound experience to meet the high demand for prenatal and abdominal care (System and individual levels) (3) Baseline Survey Methods: NI (4) Impact on Instructional Design: Stratified instruction based on learner proficiency	(1) Training Objectives: Skill-related objectives (2) Training Methods: Self-study modules, remote feedback and supervision (3) Training Content/Plan/Syllabus: Progressive structures and modular contents	(1) Instructors ① Clinical Instructors: Hospital specialists ② Student-to-Instructor Ratio: Scalable instruction (2) Teaching Aids ① POCUS ② Electronic training devices, self-learning platforms and remote feedback systems (3) Teaching Materials: Lecture handouts, powerpoint presentations (PPTs), course plans/syllabi and adapted ultrasound manuals based on standardized guidelines	(1) Time ① Training Duration: Long-term trainings (≥ 12 weeks) ② Follow-up Timing After Training: Immediate follow-up post-training ③ Follow-up Duration: NI (2) Location/Setting ① Training Sites: Healthcare institutions ② Practice Sites: Local hospitals or clinics	(1) Formative Evaluation: Telemedicine-based image review; scores improved from 6.54 to 7.17 (2) Summative Evaluation: 132 patients scanned; 52% had new diagnoses, 48% had management changes (3) Follow-up: Trainees used POCUS independently; remote support scaled back for sustainability	(1) Knowledge Retention (2) Practical Application	NI	NI
														(3) Participants ① Trainer: Healthcare professionals ② Trainee: Nurses and clinicians (In-service medical and nursing personnel) (4) Execution Process ① Practical Skills Training Phase: trainee-performed scanning on real patients ② Clinical Practice Phase: Clinical practice activities ③ Assessment and Feedback Phase: Expert evaluation of scan image quality (5) Adaptation Records ① Adaptations Based on Learner-specific Differences: Adjustment based on learning pace				
7	Lee et al. [34]	Uganda	Bwindi Nursing School	Quasi-experimental study	22	Range: 18–26	(1) Male: 72.73% (16/22) (2) Female: 27.27% (6/22)	Students: 100% (22/22)	NI	None	(1) Trainee Groups: Students (2) Needs Analysis: To improve maternal health outcomes in Uganda by addressing high maternal mortality and limited access to imaging through the integration of ultrasound into national health strategies (National level) (3) Baseline Survey Methods: NI (4) Impact on Instructional Design: Stratified instruction based on learner proficiency	(1) Training Objectives: Skill-related objectives (2) Training Methods: Practical skills training (including simulation) and self-study modules (3) Training Content/Plan/Syllabus: Progressive structures, modular contents, practice-oriented designs, clinical case integrations and certification modules	(1) Instructors ① Clinical Instructors: Sonographers; Academic Instructors: University faculty (faculty members/researchers) and certified experts or lecturers in related fields ② Student-to-Instructor Ratio: Scalable instruction (2) Teaching Aids ① POCUS ② Electronic training devices (3) Teaching Materials: Lecture handouts and instructional videos	(1) Time ① Training Duration: Long-term trainings (≥ 12 weeks) ② Follow-up Timing After Training: NI ③ Follow-up Duration: > 6 Months (2) Location/Setting ① Training Sites: Healthcare and educational institutions ② Practice Sites: Local hospitals or clinics	(1) Formative Evaluation: Weekly quizzes, skill checks, and student feedback on video quality and training experience (2) Summative Evaluation: Final written and practical exams; all students passed with ≥ 80% (3) Follow-up: Graduates committed to 3 years in rural Uganda; follow-up planned to assess long-term impact	(1) Knowledge Acquisition	(1) Maternity Attendance Rate (2) Psychological Outcomes	NI
														(3) Participants ① Trainer: Healthcare professionals ② Trainee: Students (4) Execution Process ① Pre-training Phase: Completion of online learning modules with assessments ② Practical Skills Training Phase: Trainee-performed scanning on real patients ③ Assessment and Feedback Phase: Written knowledge assessments and practical skills examinations (5) Adaptation Records ① Adaptations in Response to External Factors: Equipment adjustment				
8	Vinayak and Brownie [35]	Kenya	(1) Lead Site: Aga Khan University Hospital (2) Pilot Sites: Kiambu, Embu and Malindi	Quasi-experimental study	9	NI	NI	Midwives: 100% (9/9)	NI	None	(1) Trainee Groups: Midwives (2) Needs Analysis: To reduce maternal and infant mortality by addressing low ultrasound access (< 5%) and workforce shortages through midwife training and tele-radiology support (National level) (3) Baseline Survey Methods: NI (4) Impact on Instructional Design: NI	(1) Training Objectives: Knowledge-related objectives and skill-related objectives (2) Training Methods: Self-study modules (3) Training Content/Plan/Syllabus: Progressive structures, modular contents and certification modules	(1) Instructors ① Clinical Instructors: Sonographers and radiologists; Academic Instructors: Certified experts or lecturers in related fields ② Student-to-Instructor Ratio: Scalable instruction (2) Teaching Aids ① POCUS ② Remote feedback systems (3) Teaching Materials: Powerpoint presentations (PPTs), e-learning materials and standard operating procedures (SOPs)	(1) Time ① Training Duration: Medium-term trainings (3–11 weeks) ② Follow-up Timing After Training: Immediate follow-up post-training ③ Follow-up Duration: NI (2) Location/Setting ① Training Sites: Healthcare institutions ② Practice Sites: Local hospitals or clinics	(1) Formative Evaluation: Daily assessments and expert feedback showed improved scanning efficiency (2) Summative Evaluation: Certification and tele-radiology confirmed 99.63% accuracy in high-risk pregnancy detection (3) Follow-up: Immediate follow-up post-training. Ongoing remote monitoring supported successful POCUS integration in rural care	(1) Diagnostic Accuracy (2) Skills Acquisition	NI	NI
														(3) Participants ① Trainer: Healthcare professionals ② Trainee: Midwives (In-service medical and nursing personnel) (4) Execution Process ① Theoretical Instruction Phase: On-site instruction in basic ultrasound concepts ② Practical Skills Training Phase: Trainer-guided hands-on ultrasound practice ③ Assessment and Feedback Phase: Performance-based skill assessments (5) Adaptation Records ① Adaptations Based on Learner-specific Differences: Adjustment based on learning pace				
9	Wachira et al. [36]	Kenya	(1) Rural Counties in Kenya: Kitui, Kilifi, Kakamega, Nakuru, Taita Taveta, Baringo, Samburu, Turkana (2) Nearby Facilities: Kenyatta University Skills Lab, Kibera, Kiandutu, Gatundu, Kiambu, Thika, Ruiru, University of Nairobi, and Aga Khan University Hospital	Quasi-experimental study	514	NI	NI	Most of the learners were non-physicians/non-sonographers	NI	None	(1) Trainee Groups: Clinical staff (2) Needs Analysis: To improve ultrasound access and training in low- and middle-income countries (LMICs) to help meet the WHO recommendation of at least one scan per pregnancy, which is often unmet in these settings (System level) (3) Baseline Survey Methods: NI (4) Impact on Instructional Design: NI	(1) Training Objectives: Knowledge-related objectives and skill-related objectives (2) Training Methods: Practical skills training (including simulation), self-study modules, remote feedback and supervision (3) Training Content/Plan/Syllabus: Progressive structures, modular contents and clinical case integrations	(1) Instructors ① Clinical Instructors: Emergency physicians, sonographers, obstetricians and gynecologists, and family medicine physicians ② Student-to-Instructor Ratio: Scalable instruction (2) Teaching Aids ① POCUS ② Electronic training devices and remote feedback systems (3) Teaching Materials: Powerpoint presentations (PPTs)	(1) Time ① Training Duration: Medium-term trainings (3–11 weeks) ② Follow-up Timing After Training: Immediate follow-up post-training ③ Follow-up Duration: NI (2) Location/Setting ① Training Sites: Healthcare and educational institutions ② Practice Sites: Local hospitals or clinics	(1) Formative Evaluation: Progress tracked through daily logbooks and instructor feedback (2) Summative Evaluation: Pre-test: 52.8% → Post-test: 90.6%; Objective Structured Clinical Examination (OSCE) pass rate: 99% (486/489), avg. score: 87.3% (3) Follow-up: Immediate follow-up post-training. Ongoing learning and feedback provided via the GUSI platform	(1) Knowledge Acquisition (2) Skills Acquisition	NI	NI
														(3) Participants ① Trainer: Healthcare professionals ② Trainee: Healthcare providers (In-service medical and nursing personnel) (4) Execution Process ① Theoretical Instruction Phase: Lectures on ultrasound techniques ② Practical Skills Training Phase: Trainer-guided hands-on ultrasound practice ③ Assessment and Feedback Phase: Written knowledge assessments, practical skills examinations and performance-based skill assessments (5) Adaptation Records: NI				
10	Ward et al. [28]	Nepal	2 Clinics (1) Mahalaxmi municipality clinic (Kathmandu outskirts) (2) Outreach clinic (2 h east of Kathmandu)	Quasi-experimental study	NI	NI	NI	(1) Nurses (2) Midwives	NI	NI	(1) Trainee Groups: Nurses and midwives (2) Needs Analysis: To equip rural providers with basic obstetric ultrasound skills for identifying high-risk pregnancies (Individual level) (3) Baseline Survey Methods: NI (4) Impact on Instructional Design: NI	(1) Training Objectives: Knowledge-related objectives, skill-related objectives and attitude-related objectives (2) Training Methods: Classroom sessions, clinical mentoring/rotations, practical skills training (including simulation), self-study modules, remote feedback and supervision (3) Training Content/Plan/Syllabus: Progressive structures, modular contents, practice-Oriented Designs and clinical case integrations	(1) Instructors ① Clinical Instructors: Radiologists, obstetricians and gynecologists, and nurses ② Student-to-Instructor Ratio: Individualized instruction (2) Teaching Aids ① POCUS ② Clinical practice with healthy volunteers and remote feedback systems (3) Teaching Materials: Lecture handouts, powerpoint presentations (PPTs)	(1) Time ① Training Duration: Long-term trainings (≥ 12 weeks) ② Follow-up Timing After Training: NI ③ Follow-up Duration: NI (2) Location/Setting ① Training Sites: Healthcare institutions ② Practice Sites: Local hospitals or clinics	(1) Formative Evaluation: Ongoing peer reviews and case discussions via WhatsApp/Facebook during training, refresher, and follow-up phases to monitor progress and adjust as needed (2) Summative Evaluation: Assessed through real-world application in mobile clinics; focuses on correct identification and referral of high-risk pregnancies (3) Follow-up: NI	NI	NI	NI
														(3) Participants ① Trainer: Healthcare professionals ② Trainee: Nurses and midwives (4) Execution Process ① Theoretical Instruction Phase: Lectures on ultrasound techniques ② Practical Skills Training Phase: Trainee-performed scanning on real patients ③Post-training support phase: Participation in refresher or continuing courses (5) Adaptation Records: NI				
11	Bentley et al. [37]	Libya	John F. Kennedy Hospital	Cohort study	31	NI	NI	Midwives: 100% (31/31)	NI	None	(1) Trainee Groups: Midwives (2) Needs Analysis: To build the skills needed to identify high-risk pregnancies, such as ectopic gestations and placental abnormalities, in contexts with limited access to sonographers and diagnostic equipment (Individual level) (3) Baseline Survey Methods: Questionnaires/surveys (4) Impact on Instructional Design: Stratified instruction based on learner proficiency	(1) Training Objectives: Skill-related objectives (2) Training Methods: Classroom sessions and practical skills training (including simulation) (3) Training Content/Plan/Syllabus: Progressive structures	(1) Instructors ① Clinical Instructors: Emergency physicians; Academic Instructors: Certified experts or lecturers in related fields ② Student-to-Instructor Ratio: Scalable instruction (2) Teaching Aids ① POCUS ② Clinical practice with healthy volunteers (3) Teaching Materials: Lecture handouts	(1) Time ① Training Duration: Short-term trainings (1–2 weeks) ② Follow-up Timing After Training: Follow-up after 6 months ③ Follow-up Duration: > 6 Months (2) Location/Setting ① Training Sites: Healthcare institutions ② Practice Sites: Local hospitals or clinics	(1) Formative Evaluation: Used pre/post-tests, surveys, and Objective Structured Clinical Examination (OSCE) during the 1-week course to assess knowledge gain, comfort, and skills (2) Summative Evaluation: Evaluated overall success through post-course and 1-year follow-up tests and OSCE (3) Follow-up: 1-year surveys and OSCE measured long-term skill retention and continued ultrasound use	(1) Knowledge Acquisition (2) Knowledge Retention (3) Practical Application	NI	NI
														(3) Participants ① Trainer: Healthcare professionals ② Trainee: Midwives (In-service medical and nursing personnel) (4) Execution Process ① Theoretical Instruction Phase: Lectures on ultrasound techniques ② Practical Skills Training Phase: Trainer-guided hands-on ultrasound practice and trainee-performed scanning on real patients ③ Assessment and Feedback Phase: Written knowledge assessments and practical skills examinations (5) Adaptation Records: NI				
12	Bidner et al. [12]	Australia	The University of South Australia’s Adelaide city campus	Cohort study	41	NI	NI	(1) Doctors: 39.0% (16/41) (2) Nurses/Midwives: 61.0% (25/41)	(1) Doctors ① Range: 11–40 years ② Mean (SD): 15.4 (5.46) years (2) Nurses/Midwives ① Range: 2–30 years ② Mean (SD): 13.18 (5.45) years	(1) With prior ultrasound experience: 65.9% (27/41) (2) Without prior ultrasound experience: 34.1% (14/41)	(1) Trainee Groups: Doctors and midwives/nurses (2) Needs Analysis: To mitigate system-level barriers—including geographic isolation, provider workload, and insufficient coverage—by integrating antenatal POCUS into routine care to improve diagnostic capacity and service accessibility (System level) (3) Baseline Survey Methods: Questionnaires/surveys (4) Impact on Instructional Design: Enhanced training in practical skills	(1) Training Objectives: Skill-related objectives (2) Training Methods: Classroom sessions and practical skills training (including simulation) (3) Training Content/Plan/Syllabus: Progressive structures, modular contents, practice-oriented designs, clinical case integrations and certification modules	(1) Instructors ① Clinical Instructors: Sonographers, obstetricians and gynecologists ② Student-to-Instructor Ratio: Scalable instruction (2) Teaching Aids ① POCUS ② High-fidelity simulation tools (3) Teaching Materials: Lecture handouts and adapted ultrasound manuals based on standardized guidelines	(1) Time ① Training Duration: Long-term trainings (≥ 12 weeks) ② Follow-up Timing After Training: Follow-up at 3 months post-training ③ Follow-up Duration: > 6 Months (2) Location/Setting ① Training Sites: Educational institutions ② Practice Sites: Simulation laboratories	(1) Formative Evaluation: Assessed immediately post-workshop using pre/post knowledge tests, Objective Structured Clinical Examination (OSCE), and satisfaction surveys; evaluated learning and skill acquisition via the New World Kirkpatrick Evaluation Framework (NWKEF) (2) Summative Evaluation: 3- and 6-month surveys measured POCUS use, confidence, and practice changes; 12-month OSCE assessed skill retention; paired t-tests and qualitative feedback analyzed long-term impact (3) Follow-up: Surveys at 3, 6, and 12 months tracked usage and confidence; 12-month refresher and OSCE reinforced learning; NWKEF applied to assess sustained clinical application and patient care impact	(1) Knowledge Acquisition (2) Knowledge Retention (3) Practical Application	(1) Maternity Attendance Rate (2) Psychological Outcomes	(1) Optimization of Resource Allocation
														(3) Participants ① Trainer: Healthcare professionals ② Trainee: Clinicians (In-service medical and nursing personnel) (4) Execution Process ① Theoretical Instruction Phase: on-site instruction in basic ultrasound concepts ② Practical Skills Training Phase: trainee-performed scanning on real patients ③ Assessment and Feedback Phase: Written knowledge assessments, practical skills examinations and Kirkpatrick framework evaluations ④ Post-training Support Phase: Participation in refresher or continuing courses (5) Adaptation Records ① Adaptations in Response to External Factors: Environmental adjustment				
13	Erlick et al. [38]	USA	A single tertiary care center	Cohort study	6	NI	NI	Students: 100% (6/6)	None	None	(1) Trainee Groups: Students (2) Needs Analysis: To equip non-expert providers in low-resource settings with obstetric Volume Sweep Imaging (OB VSI), a simplified ultrasound technique designed to facilitate skill acquisition and clinical use (Individual level) (3) Baseline Survey Methods: NI (4) Impact on Instructional Design: NI	(1) Training Objectives: Knowledge-related objectives and skill-related objectives (2) Training Methods: Classroom sessions and practical skills training (including simulation) (3) Training Content/Plan/Syllabus: Progressive structures, modular contents and certification modules	(1) Instructors ① Clinical Instructors: Obstetricians and gynecologists; Academic Instructors: University faculty (faculty members/researchers) ② Student-to-Instructor Ratio: NI (2) Teaching Aids ① POCUS ② Clinical practice with healthy volunteers (3) Teaching Materials: Developed ultrasound guidebooks based on training design	(1) Time ① Training Duration: Long-term trainings (≥ 12 weeks) ② Follow-up Timing After Training: Immediate follow-up post-training ③ Follow-up Duration: > 6 Months (2) Location/Setting ① Training Sites: Healthcare institutions ② Practice Sites: Local hospitals or clinics	(1) Formative Evaluation: Assessed immediately after training using pre/post knowledge tests, hands-on practice, and review of protocol deviations in trainee scans (2) Summative Evaluation: Trainees’ scans reviewed by specialists for protocol adherence, image quality (rated as excellent/acceptable/poor), and diagnostic value (e.g., fetal heart, placenta, amniotic fluid) (3) Follow-up: Immediate follow-up post-training. Ongoing tracking of protocol errors and image quality over time to assess skill retention and improvement with continued scanning experience	(1) Diagnostic Accuracy	NI	NI
														(3) Participants ① Trainer: Academic faculty members ② Trainee: Students (4) Execution Process ① Theoretical Instruction Phase: On-site instruction in basic ultrasound concepts, lectures on ultrasound techniques and online instruction in ultrasound principles ② Practical Skills Training Phase: Trainer-guided hands-on ultrasound practice ③ Clinical Practice Phase: Clinical practice activities ④ Assessment and Feedback Phase: Written knowledge assessments, expert evaluation of scan image quality and protocol adherence assessments (5) Adaptation Records: NI				
14	Greenwold et al. [39]	Mozambique	(1) Lead Site: Mandimba Health Clinic (2) Pilot Sites: Rural health centers in Lissiete, Lussungasse and Ntembo	Cohort study	10	NI	NI	Nurses and clinical officers: 100% (9/9)	NI	None	(1) Trainee Groups: Nurses and clinical staff (2) Needs Analysis: To train providers in basic obstetric ultrasound for the detection of high-risk conditions such as placenta previa and multiple pregnancies, particularly in settings without prior ultrasound access (Individual level) (3) Baseline Survey Methods: NI (4) Impact on Instructional Design: Hierarchical structuring of instructional content, diversified teaching methods and enhanced training in practical skills	(1) Training Objectives: Knowledge-related objectives, skill-related objectives and attitude-related objectives (2) Training Methods: Classroom sessions, practical skills training (including simulation), self-study modules, remote feedback and supervision (3) Training Content/Plan/Syllabus: Progressive structures, modular contents and clinical case integrations	(1) Instructors ① Clinical Instructors: Sonographers ② Student-to-Instructor Ratio: Scalable instruction (2) Teaching Aids ① POCUS ② Electronic training devices and remote feedback systems (3) Teaching Materials: Instructional videos	(1) Time ① Training Duration: Long-term trainings (≥ 12 weeks) ② Follow-up Timing After Training: Follow-up at 4 months post-training ③ Follow-up Duration: NI (2) Location/Setting ① Training Sites: Healthcare institutions ② Practice Sites: Local hospitals or clinics	(1) Formative Evaluation: During the 8-week training, trainers assessed protocol deviations and image quality, providing immediate feedback on scanning technique (2) Summative Evaluation: After training, scans were reviewed for protocol adherence and diagnostic usefulness (e.g., fetal heart, presentation, placenta) (3) Follow-up: At 4 months post-training, trainees’ independent scans were evaluated remotely via mobile tools to monitor skill retention and ongoing use	(1) Practical Application	(1) Maternity Attendance Rate (2) Identification of High-Risk Pregnancies	NI
														(3) Participants ① Trainer: Healthcare professionals ② Trainee: Nurses and clinical educators (In-service medical and nursing personnel) (4) Execution Process ① Theoretical Instruction Phase: Lectures on ultrasound techniques ② Practical Skills Training Phase: Self-directed scanning practice and trainee-performed scanning on real patients ③ Clinical Practice Phase: Clinical practice activities ④ Assessment and Feedback Phase: Expert evaluation of scan image quality and protocol adherence assessments ⑤ Post-training Support Phase: Ongoing feedback and quality assurance measures (5) Adaptation Records: NI				
15	Henwood et al. [40]	Rwanda	Rwandan hospitals	Cohort study	17	NI	NI	Doctors: 100% (17/17)	NI	NI	(1) Trainee Groups: Doctors (2) Needs Analysis: To fill diagnostic gaps identified through surveys and hospital reviews by addressing key ultrasound needs such as FAST, obstetric, and abdominal scans (System level) (3) Baseline Survey Methods: NI (4) Impact on Instructional Design: NI	(1) Training Objectives: Knowledge-related objectives, skill-related objectives and attitude-related objectives (2) Training Methods: Classroom sessions, clinical mentoring/rotations and practical skills training (including simulation) (3) Training Content/Plan/Syllabus: Progressive structures, modular contents, practice-oriented designs and clinical case integrations	(1) Instructors ① Clinical Instructors: Emergency physicians, obstetricians and gynecologists ② Student-to-Instructor Ratio: Scalable instruction (2) Teaching Aids ① POCUS ② Remote feedback systems and clinical practice with healthy volunteers (3) Teaching Materials: Lecture handouts and instructional videos	(1) Time ① Training Duration: Short-term trainings (1–2 weeks) ② Follow-up Timing After Training: NI ③ Follow-up Duration: 3–6 Months (2) Location/Setting ① Training Sites: Healthcare institutions ② Practice Sites: Local hospitals or clinics	(1) Formative Evaluation Conducted during the 10-day training using OSCE and supervised scanning to assess image acquisition and interpretation skills (2) Summative Evaluation > 6 Months, trainees’ independent scans were reviewed for quality (0–4 scale); clinical logs tracked ultrasound impact on patient management (3) Follow-up: Remote image review and expert feedback provided via cloud system; follow-up sessions every 6 weeks reinforced skill retention and clinical application	(1) Diagnostic Accuracy (2) Practical Application	NI	(1) Cost-Effectiveness (2) Optimization of Resource Allocation
														(3) Participants ① Trainer: Healthcare professionals ② Trainee: Clinicians (In-service medical and nursing personnel) (4) Execution Process ① Theoretical Instruction Phase: On-site instruction in basic ultrasound concepts ② Practical Skills Training Phase: Trainer-guided hands-on ultrasound practice ③ Assessment and Feedback Phase: Practical skills examinations, expert evaluation of scan image quality and performance-based skill assessments (5) Adaptation Records: NI				
16	Kotagal et al. [41]	USA	University of Washington	Cohort study	16	NI	NI	Doctors: 100% (16/16)	NI	NI	(1) Trainee Groups: Doctors (2) Needs Analysis: To provide training in obstetric ultrasound for essential evaluations—such as fetal presentation, gestational age, and ectopic pregnancy—particularly in low-resource settings (Individual level) (3) Baseline Survey Methods: NI (4) Impact on Instructional Design: NI	(1) Training Objectives: Knowledge-related objectives and skill-related objectives (2) Training Methods: Classroom sessions and practical skills training (including simulation) (3) Training Content/Plan/Syllabus: Progressive structures	(1) Instructors ① Clinical Instructors: Emergency physicians, obstetricians and gynecologists ② Student-to-Instructor Ratio: NI (2) Teaching Aids ① POCUS ② Clinical practice with healthy volunteers (3) Teaching Materials: Powerpoint presentations (PPTs)	(1) Time ① Training Duration: Long-term trainings (≥ 12 weeks) ② Follow-up Timing After Training: NI ③ Follow-up Duration: 3–6 Months (2) Location/Setting ① Training Sites: Healthcare institutions ② Practice Sites: Local hospitals or clinics	(1) Formative Evaluation: Immediately post-training, confidence and skills assessed via pre/post surveys and Objective Structured Clinical Examination (OSCE); data analyzed with Wilcoxon and paired t-tests (2) Summative Evaluation: Post-course surveys and expert image reviews evaluated confidence and diagnostic skill; paired t-tests compared pre/post outcomes (3) Follow-up: At 3 months, surveys assessed continued POCUS use and confidence; periodic assessments tracked long-term skill retention, analyzed with paired t-tests	NI	NI	NI
														(3) Participants ① Trainer: Healthcare professionals ② Trainee: Residents (In-service medical and nursing personnel) (4) Execution Process ① Theoretical Instruction Phase: On-site instruction in basic ultrasound concepts ② Practical Skills Training Phase: Trainee-performed scanning on real patients ③ Assessment and Feedback Phase: Practical skills examinations, expert evaluation of scan image quality and self-confidence evaluations (5) Adaptation Records: NI				
17	Miles et al. [42]	USA	An urban, tertiary, 100-bed level I trauma center in north Texas with 130,000 annual visits, including 5000 obstetric-related visits	Cohort study	11	NI	NI	Nurses: 100% (11/11)	(1) General nursing experience ① Mean: 8.27 years ② Range: 3–25 years ③ Mode: 5 years (2) Emergency department nursing experience ① Mean: 9.09 years ② Range: 4–27 years ③ Mode: 4 & 10 years	(1) With prior ultrasound experience: 72.7% (8/11) (2) Without prior ultrasound experience: 27.3% (3/11)	(1) Trainee Groups: Nurses (2) Needs Analysis: To improve the accuracy of fetal heart rate (FHR) detection using POCUS through targeted training, addressing the limitations of Doppler devices and reducing false fetal distress alarms (Individual level) (3) Baseline Survey Methods: NI (4) Impact on Instructional Design: Stratified instruction based on learner proficiency	(1) Training Objectives: Skill-related objectives (2) Training Methods: Classroom sessions, practical skills training (including simulation) and self-study modules (3) Training Content/Plan/Syllabus: Progressive structures, modular contents, practice-oriented designs and certification modules	(1) Instructors ① Clinical Instructors: Emergency physicians and nurses ② Student-to-Instructor Ratio: Individualized instruction (2) Teaching Aids ① POCUS ② Self-learning platforms and clinical practice with healthy volunteers (3) Teaching Materials: Instructional videos	(1) Time ① Training Duration: Long-term trainings (≥ 12 weeks) ② Follow-up Timing After Training: NI ③ Follow-up Duration: NI (2) Location/Setting ① Training Sites: Healthcare institutions ② Practice Sites: Simulation laboratories	(1) Formative Evaluation: Assessed nurses’ immediate competency and confidence post-training using clinical supervision and Likert-scale surveys (2) Summative Evaluation: Compared POCUS vs. Doppler for fetal heart rate timing and accuracy using Wilcoxon test and Bland–Altman plots during clinical application (3) Follow-up: Post-implementation surveys and ongoing physician supervision monitored confidence, skill retention, and continued POCUS use	(1) Skills Acquisition (2) Practical Application	(1) Psychological Outcomes	NI
														(3) Participants ① Trainer: Healthcare professionals ② Trainee: Nurses (In-service medical and nursing personnel) (4) Execution Process ① Theoretical Instruction Phase: On-site instruction in basic ultrasound concepts and lectures on ultrasound techniques ② Practical Skills Training Phase: Trainee-performed scanning on real patients ③ Clinical Practice Phase: Clinical practice activities ④ Assessment and Feedback Phase: Performance-based skill assessments and self-confidence evaluations (5) Adaptation Records: NI				
18	Rominger et al. [43]	Mexico	10 Compañeros En Salud (CES)-supported rural outpatient clinics	Cohort study	NI	NI	NI	Pasantes	NI	NI	(1) Trainee Groups: Doctors (2) Needs Analysis: To train providers in obstetric POCUS to detect common maternal complications, such as placenta previa and fetal malposition (Individual level) (3) Baseline Survey Methods: NI (4) Impact on Instructional Design: Hierarchical structuring of instructional content	(1) Training Objectives: Knowledge-related objectives and skill-related objectives (2) Training Methods: Classroom sessions, clinical mentoring/rotations and practical skills training (including simulation) (3) Training Content/Plan/Syllabus: Progressive structures and certification modules	(1) Instructors ① Clinical Instructors: Emergency physicians, obstetricians and gynecologists ② Student-to-Instructor Ratio: Individualized instruction (2) Teaching Aids ① POCUS ② Self-learning platforms and clinical practice with healthy volunteers (3) Teaching Materials: Lecture handouts	(1) Time ① Training Duration: Long-term trainings (≥ 12 weeks) ② Follow-up Timing After Training: Follow-up at 6 months post-training ③ Follow-up Duration: > 6 Months (2) Location/Setting ① Training Sites: Healthcare and educational institutions ② Practice Sites: Local hospitals or clinics	(1) Formative Evaluation: Assessed physicians’ confidence and skills immediately after training through surveys and supervised practice (2) Summative Evaluation: Analyzed ultrasound logs to evaluate the impact of POCUS on diagnostic accuracy and clinical decision-making (3) Follow-up: Conducted 6- and 12-month surveys, image reviews, and ongoing supervision to assess skill retention and continued clinical use	(1) Knowledge Retention (2) Practical Application	NI	NI
														(3) Participants ① Trainer: Healthcare professionals ② Trainee: Clinicians (In-service medical and nursing personnel) (4) Execution Process ① Theoretical Instruction Phase: On-site instruction in basic ultrasound concepts, Lectures on ultrasound techniques ② Practical Skills Training Phase: Trainee-performed scanning on real patients ③ Clinical Practice Phase: Clinical practice activities ④ Assessment and Feedback Phase: Performance-based skill assessments and self-confidence evaluations ⑤ Post-training Support Phase: Follow-up meetings (5) Adaptation Records: NI				
19	Shah et al. [3]	Uganda	(1) Public District Hospital (DH) and 3 Health Centers (HC)	Cohort study	25	NI	(1) Male: 8.0% (2/25) (2) Female: 92.0% (23/25)	(1) Doctors: 8.0% (2/25) (2) Nurses: 12.0% (3/25) (3) Midwives: 80.0% (20/25)	NI	None	(1) Trainee Groups: Nurses, midwives and doctors (2) Needs Analysis: To train providers in low-resource settings to identify high-risk conditions early in pregnancy and strengthen their diagnostic capacity (Individual level) (3) Baseline Survey Methods: Likert scales (4) Impact on Instructional Design: Hierarchical structuring of instructional content and enhanced training in practical skills	(1) Training Objectives: Skill-related objectives (2) Training Methods: Classroom sessions, practical skills training (including simulation), self-study modules (3) Training Content/Plan/Syllabus: Practice-oriented designs	(1) Instructors ① Clinical Instructors: Sonographers and nurses ② Student-to-Instructor Ratio: Individualized instruction (2) Teaching Aids ① POCUS ② Remote feedback systems and clinical practice with healthy volunteers (3) Teaching Materials: Powerpoint presentations (PPTs), instructional videos and adapted ultrasound manuals based on standardized guidelines	(1) Time ① Training Duration: Medium-term trainings (3–11 weeks) ② Follow-up Timing After Training: Follow-up at 3 months post-training ③ Follow-up Duration: NI (2) Location/Setting ① Training Sites: Healthcare institutions ② Practice Sites: Local hospitals or clinics	(1) Formative Evaluation Surveys and WhatsApp feedback used to track progress; conducted pre-training, post-training, and at 3-month follow-up (2) Summative Evaluation: Objective Structured Clinical Examination (OSCE) administered after 25 proctored scans to assess competency (3) Follow-up: 3-month post-training survey to evaluate retention	(1) Diagnostic Accuracy (2) Skills Acquisition (3) Practical Application	NI	NI
														(3) Participants ① Trainer: Healthcare professionals ② Trainee: Midwives, nurses and clinicians (In-service medical and nursing personnel) (4) Execution Process ① Pre-training Phase: Pre-course self-directed learning activities ② Theoretical Instruction Phase: Lectures on ultrasound techniques ③ Practical Skills Training Phase: Trainer-guided hands-on ultrasound practice ④ Assessment and Feedback Phase: Practical skills examinations ⑤ Post-training Support Phase: Peer learning through online communities (5) Adaptation Records ① Adaptations Based on Training Content and Delivery Methods: Delivery methods adjustment				
20	Varner et al. [44]	Canada	(2) Hospital-affiliated academic family medicine clinics	Cohort study	12	NI	NI	Doctors: 100% (12/12)	NI	NI	(1) Trainee Groups: Doctors (2) Needs Analysis: NI (3) Baseline Survey Methods: NI (4) Impact on Instructional Design: Hierarchical structuring of instructional content	(1) Training Objectives: Knowledge-related objectives, skill-related objectives and attitude-related objectives (2) Training Methods: Self-study modules, remote feedback and supervision (3) Training Content/Plan/Syllabus: Progressive structures and modular contents	(1) Instructors ① Academic Instructors: Certified experts or lecturers in related fields ② Student-to-Instructor Ratio: Individualized instruction (2) Teaching Aids ① POCUS ② Clinical practice with healthy volunteers (3) Teaching Materials: E-learning materials and adapted ultrasound manuals based on standardized guidelines	(1) Time ① Training Duration: Short-term trainings (1–2 weeks) ② Follow-up Timing After Training: Immediate follow-up post-training ③ Follow-up Duration: 3–6 Months (2) Location/Setting ① Training Sites: Healthcare institutions ② Practice Sites: Local hospitals or clinics	(1) Formative Evaluation: NI (2) Summative Evaluation: Evaluate family physicians’ POCUS use, accuracy, pregnancy outcomes, and emergency department visits by retrospective chart review, 6 months after training (3) Follow-up: Immediate follow-up post-training	(1) Diagnostic Accuracy	NI	NI
														(3) Participants ① Trainer: Academic faculty members ② Trainee: Family physicians (In-service medical and nursing personnel) (4) Execution Process ① Pre-training Phase: Completion of online learning modules with assessments ② Theoretical Instruction Phase: On-site instruction in basic ultrasound concepts ③ Practical Skills Training Phase: Trainer-guided hands-on ultrasound practice and self-directed scanning practice ④ Assessment and Feedback Phase: Performance-based skill assessments (5) Adaptation Records: NI				
21	Westerway [45]	(1) Australia (2) Overseas: Timor-Leste, Indonesia and Central Asia	(1) Australia Sites: city hospital, rural hospital and private hospital (2) Overseas Sites: women’s clinic, rural clinic and rural hospital	Cohort study	55	NI	NI	(1) Doctors: 27.3% (15/55) (2) Midwives: 41.8% (23/55) (3) Nurses: 16.4% (9/55) (4) Radiographers: 14.5% (8/55)	NI	None	(1) Trainee Groups: Doctors, midwives, nurses and medical imaging technologists (2) Needs Analysis: To improve access to third-trimester ultrasound in low-resource and rural settings, where maternal mortality remains high and WHO recommendations for minimum scanning are often unmet; training aimed to equip non-sonographers with practical scanning skills (System and Individual levels) (3) Baseline Survey Methods: Multiple-choice questions/exams (4) Instructional Design Impact: Diversified teaching methods and enhanced training in practical skills	(1) Training Objectives: Skill-related objectives (2) Training Methods: Classroom sessions and practical skills training (including simulation) (3) Training Content/Plan/Syllabus: Progressive structures, practice-oriented designs and clinical case integrations	(1) Instructors ① Clinical Instructors: Ultrasonologists; Academic Instructors: Certified experts or lecturers in related fields ② Student-to-Instructor Ratio: Individualized instruction (2) Teaching Aids ① POCUS ② Clinical practice with healthy volunteers (3) Teaching Materials: Lecture handouts	(1) Time ① Training Duration: Long-term trainings (≥ 12 weeks) ② Follow-up Timing After Training: Follow-up at 6 months post-training ③ Follow-up Duration: > 6 Months (2) Location/Setting ① Training Sites: Healthcare institutions ② Practice Sites: Local hospitals or clinics	(1) Formative Evaluation: NI (2) Summative Evaluation: Assessed participants’ knowledge, scanning skills, course satisfaction, and training effectiveness using a post-course test and evaluation forms at course end (3) Follow-up: Evaluated skill retention, practical application, confidence, and patient impact with 31 participants (21 Australia, 10 overseas) using an expanded test, image review, and practical scan assessment 6–11 months post-course	(1) Skills Acquisition	NI	NI
														(3) Participants ① Trainer: Healthcare professionals ② Trainee: Healthcare providers (In-service medical and nursing personnel) (4) Execution Process ① Pre-training Phase: Online pre-tests for baseline assessment ② Theoretical Instruction Phase: Lectures on ultrasound techniques ③ Practical Skills Training Phase: Trainer-guided hands-on ultrasound practice and trainee-performed scanning on real patients ④ Assessment and Feedback Phase: Written knowledge assessments and performance-based skill assessments (5) Adaptation Records: NI				
22	Lee et al. [46]	Indonesia	Public health care clinics	Cross-sectional study	41	NI	NI	Doctors: 100% (41/41)	NI	(1) Had no prior ultrasound experience: 53.7% (22/41) (2) Had only observed the use of ultrasound: 43.9% (18/41) (3) Had taken a prior ultrasound course: 2.4% (1/41)	(1) Trainee Groups: Doctors (2) Needs Analysis: To address the low use of sonography among general practitioners in Indonesia by providing POCUS training to improve pathology detection (Individual level) (3) Baseline Survey Methods: Multiple-choice questions/exams (4) Impact on Instructional Design: Hierarchical structuring of instructional content	(1) Training Objectives: Skill-related objectives (2) Training Methods: Classroom sessions and practical skills training (including simulation) (3) Training Content/Plan/Syllabus: Modular contents	(1) Instructors ① Academic Instructors: Medical students with ultrasound training during their studies ② Student-to-Instructor Ratio: NI(2) Teaching Aids ① POCUS ② Clinical practice with healthy volunteers (3) Teaching Materials: Lecture handouts	(1) Time ① Training Duration: Medium-term trainings (3–11 weeks) ② Follow-up Timing After Training: Immediate follow-up post-training ③ Follow-up Duration: > 6 Months (2) Location/Setting:① Training Sites: Healthcare institutions ② Practice Sites: Local hospitals or clinics (3) Participants	(1) Formative Evaluation: Assessed understanding through multiple-choice quizzes at the end of each session (2) Summative Evaluation: Measured knowledge, practical skills, and confidence with pre- and post-course exams, practical exam, and surveys at the course’s start and end (3) Follow-up: Evaluated long-term knowledge retention, POCUS use, and accuracy via repeat exams and surveys one year after training	(1) Knowledge Acquisition (2) Skills Acquisition (3) Practical Application	NI	(1) Cost-Effectiveness
														① Trainer: Academic faculty members ② Trainee: Healthcare providers (In-service medical and nursing personnel) (4) Execution Process ① Pre-training Phase: Participation in pre-course examinations ② Theoretical Instruction Phase: On-site instruction in basic ultrasound concepts, lectures on ultrasound techniques and online instruction in ultrasound principles ③ Practical Skills Training Phase: Trainer-guided hands-on ultrasound practice, self-directed scanning practice, case-based scenario applications and discussions, trainee-performed scanning on real patients ④ Assessment and Feedback Phase: Written knowledge assessments and surveys/questionnaires (5) Adaptation Records: NI				
23	Nathan et al. [50]	Congo, Guatemala, Kenya, Pakistan and Zambia	Countries: Karawa, Chimaltenango, Eldoret, Karachi and Lusaka	Cross-sectional study	41	NI	(1) Male: 39.0% (16/41) (2) Female: 61.0% (25/41)	(1) Nurse: 43.9% (18/41) (2) Midwives: 14.6% (6/41) (3) Medical Officer: 24.4% (10/41) (4) Radiographer: 17.1% (7/41)	(1) Congo ① Mean: 7.3 years ② Range 4–12 years (2) Guatemala ① Mean: 10.3 years ② Range 0.8–23 years (3) Kenya ① Mean: 2.8 years ② Range 0.7–4 years (4) Pakistan ① Mean: 0.5 years ② Range 0–1.5 years (5) Zambia ① Mean: 16.6 years ② Range 2–39 years	None	(1) Trainee Groups: Nurses, midwives, clinical staff and medical imaging technologists (2) Needs Analysis: To train local staff in rural centers to perform basic obstetric ultrasound for high-risk pregnancy screening, addressing the shortage of trained sonographers (Individual level) (3) Baseline Survey Methods: NI (4) Impact on Instructional Design: Hierarchical structuring of instructional content and enhanced training in practical skills	(1) Training Objectives Skill-related objectives and attitude-related objectives (2) Training Methods Classroom sessions, clinical mentoring/rotations and practical skills training (including simulation) (3) Training Content/Plan/Syllabus: Progressive structures and practice-oriented designs	(1) Instructors ① Clinical Instructors: Ultrasonologists ② Student-to-Instructor Ratio: Scalable instruction (2) Teaching Aids ① POCUS (3) Teaching Materials: Lecture handouts and scan data sheets	(1) Time ① Training Duration: Long-term trainings (≥ 12 weeks) ② Follow-up Timing After Training: Follow-up at 4 months post-training ③ Follow-up Duration: 3–6 Months (2) Location/Setting ① Training Sites: Healthcare institutions ② Practice Sites: Local hospitals or clinics and healthcare facilities in other low-resource countries	(1) Formative Evaluation: Trainees’ progress was monitored through monthly practical exams, scan reviews on the quality control website, and regular meetings with local trainers. Each trainee completed two supervised ultrasounds daily, with ongoing evaluations throughout the training and pilot phases (2) Summative Evaluation: The program’s overall effectiveness was assessed with written and practical exams at the end of the 2-week course, and a practical exam at the conclusion of the 12-week pilot phase (3) Follow-up: Repeat scanning skills tests over 12 weeks to monitor progress; mean score increased from 78% on the first test to 92% on the fourth test; 40 of 41 trainees passed the final test, and one withdrew from the study	(1) Diagnostic Accuracy (2) Knowledge Acquisition (3) Skills Acquisition (4) Practical Application	NI	NI
														(3) Participants ① Trainer: Healthcare professionals ② Trainee: Healthcare providers (In-service medical and nursing personnel) (4) Execution Process ① Theoretical Instruction Phase: On-site instruction in basic ultrasound concepts ② Practical Skills Training Phase: Trainer-guided hands-on ultrasound practice ③ Clinical Practice Phase: Clinical practice activities ④ Assessment and Feedback Phase: Written knowledge assessments, practical skills examinations and performance-based skill assessments ⑤ Post-training Support Phase: participation in refresher or continuing O30courses (5) Adaptation Records: NI				
24	Shah et al. [47]	Rwanda	2 Rural hospitals	Cross-sectional study	NI	NI	NI	Doctors	NI	NI	(1) Trainee Groups: Doctors (2) Needs Analysis: NI (3) Baseline Survey Methods: NI (4) Impact on Instructional Design: NI	(1) Training Objectives: Skill-related objectives (2) Training Methods: Classroom sessions and practical skills training (including simulation) (3) Training Content/Plan/Syllabus: Progressive structures, practice-oriented designs and clinical case integrations	(1) Instructors ① Clinical Instructors: Emergency physicians; Academic Instructors: Certified experts or lecturers in related fields ② Student-to-Instructor Ratio: Scalable instruction (2) Teaching Aids ① POCUS (3) Teaching Materials: Lecture handouts and scan data sheets	(1) Time ① Training Duration: Medium-term trainings (3–11 weeks) ② Follow-up Timing After Training: Immediate follow-up post-training ③ Follow-up Duration: 10 Weeks (2) Location/Setting ① Training Sites: Healthcare institutions ② Practice Sites: Local hospitals or clinics	(1) Formative Evaluation: NI (2) Summative Evaluation: Evaluate trainees’ accuracy in interpreting ultrasounds and impact on patient care using scan data and blinded review by a US expert, conducted after training and during follow-up (3) Follow-up: Assess long-term program sustainability and diagnostic accuracy through ongoing case reviews and quality checks by local physicians with remote expert support	(1) Diagnostic Accuracy (2) Practical Application	NI	(1) Optimization of Resource Allocation
														(3) Participants ① Trainer: Healthcare professionals ② Trainee: Clinicians (In-service medical and nursing personnel) (4) Execution Process ① Theoretical Instruction Phase: Lectures on ultrasound techniques ② Practical Skills Training Phase: Trainer-guided hands-on ultrasound practice ③ Clinical Practice Phase: Clinical practice activities ④ Assessment and Feedback Phase: Written knowledge assessments and evaluation of patient clinical management plans (5) Adaptation Records: NI				
25	Shokoohi et al. [4]	(1) Training place: USA (2) Pilot places: Malawi, Tanzania and Uganda	Academic institutions	Cross-sectional study	49	NI	NI	Midwives: 14.3% (7/49) Note: The professional roles of the remaining 42 participants were not specified	(1) Years from Completing Residency ① < 5 Years: 55.1% (27/49) ② 6–10 Years: 10.2% (5/49) ③ > 10 Years: 34.7% (17/49) (2) Years of Providing Service at the Host Country ① 2016–2017: 46.9% (23/49) ② 2015–2016: 26.5% (13/49) ③ 2014–2015: 24.5% (12/49) ④ 2013–2014: 22.4% (11/49)	(1) Number of scans before Global Health Service Partnership ① None: 42.9% (21/49) ② 20–40: 28.6% (14/49) ③ 40–100: 4.1% (2/49) ④ 100–1000: 16.3% (8/49) ⑤ > 1000: 8.2% (4/49)	(1) Trainee Groups: Midwives and others (2) Needs Analysis: NI (3) Baseline Survey Methods: Questionnaires/surveys (4) Impact on Instructional Design: Diversified teaching methods	(1) Training Objectives: Skill-related objectives (2) Training Methods: Clinical mentoring/rotations, practical skills training (including simulation), self-study modules, remote feedback and supervision (3) Training Content/Plan/Syllabus: Modular contents and clinical case integrations	(1) Instructors ① Academic Instructors: Authors with relevant experience in ultrasound ② Student-to-Instructor Ratio: Individualized instruction (2) Teaching Aids ① POCUS ② Remote feedback systems (3) Teaching Materials: E-learning materials	(1) Time ① Training Duration: Long-term trainings (≥ 12 weeks) ② Follow-up Timing After Training: NI ③ Follow-up Duration: > 6 Months (2) Location/Setting ① Training Sites: Educational institutions ② Practice Sites: Local hospitals or clinics and healthcare facilities in other low-resource countries	(1) Formative Evaluation: NI (2) Summative Evaluation: The program’s effectiveness was evaluated through a 35-question survey assessing educators’ medical experience, POCUS usage, training adequacy, and barriers during their 1-year deployment (3) Follow-up: Support ultrasound practice throughout the year by pairing educators with local counterparts, reviewing transmitted images remotely, and providing repeat on-site instruction during the placement period	(1) Practical Application	NI	NI
														(3) Participants ① Trainer: Academic faculty members ② Trainee: Clinical educators (In-service medical and nursing personnel) (4) Execution Process ① Pre-training Phase: Pre-course Self-directed learning activities ② Theoretical Instruction Phase: On-site instruction in basic ultrasound concepts and online instruction in ultrasound principles ③ Practical Skills Training Phase: Trainer-guided hands-on ultrasound practice, self-directed scanning practice, case-based scenario applications and discussions, trainee-performed scanning on real patients ④ Clinical Practice Phase: Clinical practice activities ⑤ Assessment and Feedback Phase: Surveys/questionnaires (5) Adaptation Records: NI				
26	Vinayak et al., [48]	Kenya	Aga Kahn University Hospital and 3 satellite clinics	Cross-sectional study	3	NI	NI	Midwives: 100% (3/3)	<3 years: 100% (3/3)	None	1) Trainee Groups: Midwives (2) Needs Analysis: To equip midwives with basic obstetric ultrasound skills for conducting POCUS examinations (Individual level) (3) Baseline Survey Methods: NI (4) Impact on Instructional Design: Hierarchical structuring of instructional content	(1) Training Objectives: Skill-related objectives (2) Training Methods: Clinical mentoring/rotations, practical skills training (including simulation), self-study modules, remote feedback and supervision (3) Training Content/Plan/Syllabus: Progressive structures, practice-oriented designs and certification modules	(1) Instructors: ① Clinical Instructors: Sonographers ② Student-to-Instructor Ratio: Scalable instruction (2) Teaching Aids: ① POCUS ② Remote feedback systems (3) Teaching Materials: Powerpoint presentations (PPTs) and e-learning materials	(1) Time: ① Training Duration: Medium-term trainings (3–11 weeks) ② Follow-up Timing After Training: Immediate follow-up post-training ③ Follow-up Duration: NI (2) Location/Setting: ① Training Sites: Healthcare institutions ② Practice Sites: Local hospitals or clinics	(1) Formative Evaluation: Assess midwives’ progress using an e-learning module and ongoing skill observation; feedback addressed by week 3. (2) Summative Evaluation: Evaluate training effectiveness by comparing midwives’ reports with radiologists’, analyzing image quality and transmission, and surveying patient satisfaction; assessments done throughout training, finalized by week 5. (3) Follow-up: Immediate follow-up post-training	(1) Diagnostic Accuracy (2) Knowledge Acquisition (3) Skills Acquisition	(1) Maternity Attendance Rate (2) Psychological Outcomes	NI
														(3) Participants: ① Trainer: Healthcare professionals ② Trainee: Midwives (In-service medical and nursing personnel) (4) Execution Process: ① Pre-training Phase: Completion of online learning modules with assessments ② Theoretical Instruction Phase: Lectures on ultrasound techniques ③ Practical Skills Training Phase: Trainer-guided hands-on ultrasound practice, self-directed scanning practice, case-based scenario applications and discussions, trainee-performed scanning on real patients ④ Clinical Practice Phase: Clinical practice activities ⑤ Assessment and Feedback Phase: Performance-based skill assessments (5) Adaptation Records: NI				
27	Wanjiku et al. [49]	Kenya	21 Rural and under-resourced healthcare facilities	Cross-sectional study	33	NI	NI	Clinical officers, nurses and medical officers	NI	None	(1) Trainee Groups: Nurses and clinical staff (2) Needs Analysis: NI (3) Baseline Survey Methods: Questionnaires/surveys (4) Impact on Instructional Design: Diversified teaching methods	(1) Training Objectives: Skill-related objectives and attitude-related objectives (2) Training Methods: Practical skills training (including simulation) and self-study modules (3) Training Content/Plan/Syllabus: Progressive structures, practice-oriented designs, clinical case integrations and certification modules	(1) Instructors ① Academic Instructors: Certified experts or lecturers in related fields ② Student-to-Instructor Ratio: NI (2) Teaching Aids ① POCUS ② Clinical practice with healthy volunteers (3) Teaching Materials: Developed ultrasound guidebooks based on training design	(1) Time ① Training Duration: Long-term trainings (≥ 12 weeks) ② Follow-up Timing After Training: Follow-up at 4 months post-training ③ Follow-up Duration: NI (2) Location/Setting ① Training Sites: Healthcare institutions ② Practice Sites: Local hospitals or clinics	(1) Formative Evaluation: Assess trainees’ progress using an online pre-test, written exam, and Objective Structured Clinical Examination (OSCE) before, during, and 3–4 months after the 1-day training (2) Summative Evaluation: Evaluate overall program effectiveness after more than one year of implementation using a 30-question written exam, structured clinical examination, clinical use survey, and image quality scoring across multiple scan types (3) Follow-up: Conduct on-site reassessments 3–4 months after each training; provide refresher training based on test performance, monitor scanning frequency, and collect feedback to improve the training design	(1) Knowledge Acquisition (2) Knowledge Retention (3) Practical Application	NI	NI
														(3) Participants ① Trainer: Academic faculty members ② Trainee: Healthcare providers (In-service medical and nursing personnel) (4) Execution Process ① Pre-training Phase: Review of multimedia manuals with online quizzes ② Theoretical Instruction Phase: On-site instruction in basic ultrasound concepts ③ Practical Skills Training Phase: Trainer-guided hands-on ultrasound practice ④ Clinical Practice Phase: Clinical practice activities ⑤ Assessment and Feedback Phase: Written knowledge assessments, practical skills examinations, surveys/questionnaires and expert evaluation of scan image quality ⑥ Post-training Support Phase: Participation in refresher or continuing courses (5) Adaptation Records: NI				

1. USA = United States of America; Tanzania = United Republic of Tanzania; Libya = The Socialist People’s Libyan Arab Jamahiriya; Mozambique = The Republic of Mozambique; Rwanda = The Republic of Rwanda; Mexico = The United Mexican States. 2. Clinical staff include clinical staff/officers, medical/hospital officers, and uncategorized healthcare providers (HCPs)

### Narrative review based on *ADDIE* model

#### Overview of the *ADDIE* model

**Fig. 2:**
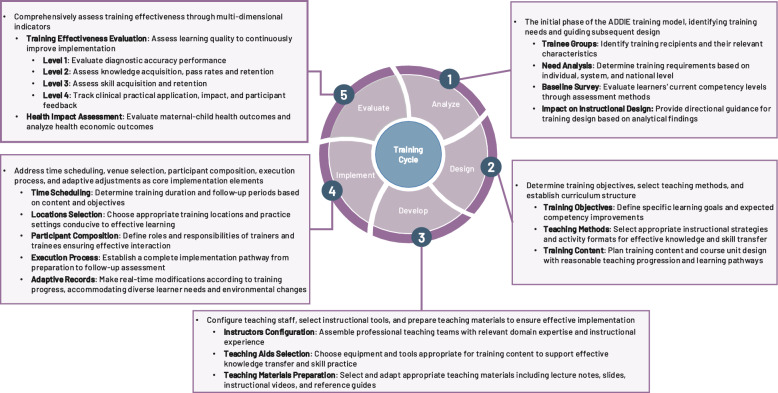
Core elements of obstetric POCUS training extracted based on ADDIE model

After a rigorous quality assessment, 26 studies were included in the narrative review and meta-analysis. The systematic analysis of the 26 included studies, using thematic analysis [24, 25] and framework-based analysis [26], revealed distinct patterns across each phase of the *ADDIE* instructional design model. The *ADDIE* model is a systematic instructional design framework comprising five interrelated phases, including *Analysis*, *Design*, *Development*, *Implementation*, and *Evaluation* (Fig. 2). It forms a training-centered, iterative cycle that provides structured methodological guidance for the planning and execution of obstetric ultrasound training programs.

The *Analysis* phase serves as the foundation, involving trainee population analysis, needs assessment, baseline surveys, and their influence on instructional design. Building upon these insights, the *Design* phase translates identified needs into specific learning objectives, instructional methods, and curriculum planning. The *Development* phase operationalizes the design by assembling instructional teams, selecting teaching tools, and preparing training materials to ensure effective content delivery. The *Implementation* phase puts the plan into action through practical arrangements, including scheduling, venue selection, participant management, and adaptive adjustments. Finally, the *Evaluation* phase, integrated throughout and extending beyond training completion, employs a multi-level framework assessing diagnostic accuracy, knowledge and skill acquisition, clinical application, and participant feedback, alongside broader health impact assessments.

These phases form a closed-loop system where each phase’s output becomes the next phase’s input, supporting continuous improvement and systematic enhancement of training programs. An overview of the basic characteristics of each *ADDIE* model phase in the included studies is presented in Table 3 and Supplementary File 6.

#### Analysis

The *Analysis* phase laid the foundation for effective training design by systematically identifying target populations, uncovering learning needs, and conducting baseline evaluations to inform instructional strategies. Across the 26 included studies, trainees came from diverse healthcare backgrounds. Doctors were the most commonly targeted group (46.2%), followed by midwives (38.5%), and nurses (34.6%). Other participants included clinical staff (19.2%), medical students (11.5%), and medical imaging technologists (11.5%). Notably, 30.8% of studies engaged multiple professional groups simultaneously, underscoring the interdisciplinary nature of obstetric POCUS training and its relevance across varied clinical settings.

To better align training with real-world gaps, 22 studies conducted formal needs assessments at three levels. At the individual level, 15 studies (57.7%) identified deficits in ultrasound knowledge or procedural skills among frontline practitioners. On the system level, 6 studies (23.1%) highlighted operational barriers such as limited access to ultrasound equipment, inadequate staffing, and insufficient supervision. At the national level, 3 studies (11.5%) addressed broader public health priorities, including high maternal mortality rates, positioning POCUS training as a strategy to mitigate critical gaps in maternal care. This multi-layered approach to needs analysis helped tailor training content to both personal competence and systemic demands.

Baseline evaluations were reported in 9 studies (34.6%) to gauge initial knowledge or skill levels before training. Tools varied, with structured questionnaires or surveys used in 4 studies (15.4%), multiple-choice exams in 3 studies (11.5%), Likert scales in 1 study (3.8%), and other tools in 1 study (3.8%). Although not universally applied, these assessments served an important role in 16 studies (61.5%) by directly shaping the instructional design. For example, 8 studies (30.8%) adjusted the sequencing of content based on learners’ starting points, while 4 (15.4%) employed stratified instruction to match varying levels of proficiency. Others diversified teaching strategies (*n* = 4, 15.4%) or emphasized practical skills training (*n* = 5, 19.2%) in response to identified baseline weaknesses. These comprehensive needs analyses laid the foundation for targeted curriculum design in the subsequent phases.

#### Design

Building on the findings from the *Analysis* phase, the *Design* phase centered on defining learning objectives, selecting appropriate teaching methods, and structuring curricula to meet diverse learner needs. All 26 studies clearly articulated training objectives, which were broadly categorized into three domains including skill-based, knowledge-based, and attitude-based. Every study (100%) emphasized skill-related goals, with a strong focus on core obstetric POCUS competencies such as probe handling, equipment operation, and image acquisition. Additionally, knowledge-based objectives such as ultrasound theory, fetal anatomy, and image interpretation were specified in 11 studies (42.3%). A smaller subset (*n* = 7, 26.9%) addressed attitudinal outcomes, particularly enhancing trainee confidence and self-efficacy in ultrasound use. Notably, half of the studies (50%) integrated objectives across multiple domains.

Instructional methods were similarly diverse, reflecting an effort to accommodate varying resource settings and learner backgrounds. Teaching strategies fell into three primary categories, namely face-to-face instruction, hands-on practical training, and remote or self-directed learning. Practical skills training was the most prevalent component (*n* = 21, 80.8%), often involving practice on simulators, volunteers, or pregnant patients under supervision. Face-to-face teaching was also common (*n* = 19, 73.1%), typically delivered through classroom-based didactics (*n* = 17, 65.4%) or supplemented with clinical mentoring and on-site rotations (*n* = 7, 26.9%). Meanwhile, remote and self-directed learning modalities were featured in 12 studies (46.2%), leveraging self-study modules and, in some cases (*n* = 7, 26.9%), incorporating remote feedback mechanisms to facilitate asynchronous learning. Importantly, 24 studies (92.3%) combined two or more of these methods, highlighting a shift toward blended learning models that integrate theoretical knowledge with experiential application.

Curriculum structure was carefully designed to support incremental learning and clinical applicability. Five distinct strategies were identified across the studies, including progressive structures, modular contents, practice-oriented designs, clinical case integration, and certification modules. A majority of studies (*n* = 22, 84.6%) employed progressive structures, gradually increasing content complexity to support skill acquisition. Modular content was used in 15 studies (57.7%) and allowed flexibility in delivery and customization. Practice-oriented designs were evident in 13 studies (50.0%), often linking teaching directly to clinical tasks. Clinical case integration appeared in 12 studies (46.2%), enhancing contextual understanding and diagnostic reasoning. Lastly, 10 studies (38.5%) embedded certification modules, either through formal evaluation or institutional recognition, to encourage learner motivation and standardization. Overall, 21 studies (80.8%) adopted multiple design strategies, reinforcing the importance of instructional flexibility and learner engagement in obstetric ultrasound training. These carefully designed curricula then needed to be operationalized through appropriate resources and expertise.

#### Development

The *Development* phase transformed the *Design* blueprint into tangible educational resources by focusing on instructor assignment, teaching tool selection, and preparation of learning materials. Clinical instructors were utilized in 19 studies (73.1%), including emergency physicians (*n* = 8), sonographers (*n* = 7), and obstetricians and gynecologists (*n* = 7). Academic instructors were involved in 13 studies (50.0%), with certified experts or lecturers in 8 studies and university faculty in 4 studies. Instructional formats varied in scale: 6 studies (23.1%) reported individualized teaching with a student-to-instructor ratio ranging from 1:1 to 1:6, while 16 studies (61.5%) described scalable formats without specific ratios.

A wide range of teaching tools was applied across the studies. All 26 studies (100%) used POCUS as the primary training tool, with 4 studies (15.4%) also incorporating electronic training devices. Self-learning platforms were used in 4 studies (15.4%), while remote feedback systems supporting image collection, storage, and expert review were implemented in 8 studies (30.8%). High-fidelity simulation tools were employed in 2 studies (7.7%). In addition, 11 studies (42.3%) included practical sessions with healthy volunteers, offering trainees direct scanning experience in non-clinical settings.

Training materials consisted of theoretical content, procedural guides, and structured data sheets. Theoretical instruction materials appeared in 23 studies (88.5%), including lecture handouts (*n* = 12), PowerPoint presentations (*n* = 10), and instructional videos (*n* = 5). Seven studies (26.9%) provided guidelines or manuals, among which 3 studies developed new ultrasound guidebooks and 4 studies adapted existing standards. Scan data sheets were reported in 3 studies (11.5%) as part of structured documentation. Eleven studies (42.3%) combined multiple types of teaching materials to reinforce learning across formats.

#### Implementation

With all resources prepared, the *Implementation* phase put the training programs into action, addressing how they were organized and delivered across diverse contexts. Training duration varied widely across studies, ranging from 1 h to 3 years. Long-term training programs (≥ 12 weeks) were the most common, reported in 14 studies (53.8%), while short-term (1–2 weeks) and medium-term (3–11 weeks) durations were each used in 6 studies (23.1%). Timing and length of follow-up assessments also differed. Immediate post-training evaluations were reported in 11 studies (42.3%), while follow-ups at 3 months (*n* = 2, 7.7%), 4 months (*n* = 3, 11.5%), 6 months (*n* = 2, 7.7%), and beyond 6 months (*n* = 1, 3.8%) were less common. In terms of follow-up duration, 9 studies (34.6%) extended beyond 6 months, 4 studies (15.4%) lasted 3–6 months, and 2 studies (7.7%) conducted follow-up at 10 weeks.

Training locations were also diverse. Most programs were implemented in healthcare institutions (*n* = 19, 73.1%), while others took place in educational institutions (*n* = 4, 15.4%) or combined settings (*n* = 3, 11.5%). Hands-on practice primarily occurred in clinical facilities (*n* = 23, 88.5%), with simulation laboratories used in 3 studies (11.5%). Three studies (11.5%) implemented training in low-resource countries, including *Guatemala*, *Peru*, *Congo*, and *Kenya*. Trainers consisted of healthcare professionals in 18 studies (69.2%) and academic faculty members in 8 studies (30.8%). Trainees were predominantly in-service medical and nursing personnel, involved in 23 studies (88.5%), while students were included in 3 studies (11.5%).

Training execution followed a multi-phase process. Pre-training activities were reported in 8 studies (30.8%), followed by theoretical instruction in 21 studies (80.8%) and practical skills training in all 26 studies (100%). Clinical practice was incorporated in 10 studies (38.5%). Assessment and feedback mechanisms were present in all studies, while post-training support was mentioned in 8 studies (30.8%). All studies included some form of follow-up evaluation. Adaptive adjustments during implementation were documented in 11 studies (42.3%). These modifications were based on training content and delivery formats (*n* = 3, 11.5%), learner-specific differences (*n* = 5, 19.2%), and external factors such as resource availability or contextual barriers (*n* = 3, 11.5%). This flexibility proved crucial to program success, as evidenced in the *Evaluation* outcomes.

#### Evaluation

The *Evaluation* phase revealed whether the carefully designed and implemented training programs achieved their intended objectives, comprehensively assessing training effectiveness through multiple dimensions. Overall, findings indicate positive trends across most reported training effectiveness evaluation indicators and health impact assessment indicators.

##### Training effectiveness evaluation

For training effectiveness, diagnostic accuracy was evaluated in 9 studies (34.6%). Among these, image quality achieved acceptability rates ranging from 88.2 to 94.8% in 3 studies (11.5%), while measurement consistency was reported in 2 studies (7.7%) with *Kappa* values equal to or greater than 0.8. Three studies (11.5%) documented diagnostic accuracy exceeding 99%, with sensitivity values ranging from 81.4 to 100% and specificity between 85.7 and 100%.

**Fig. 3:**
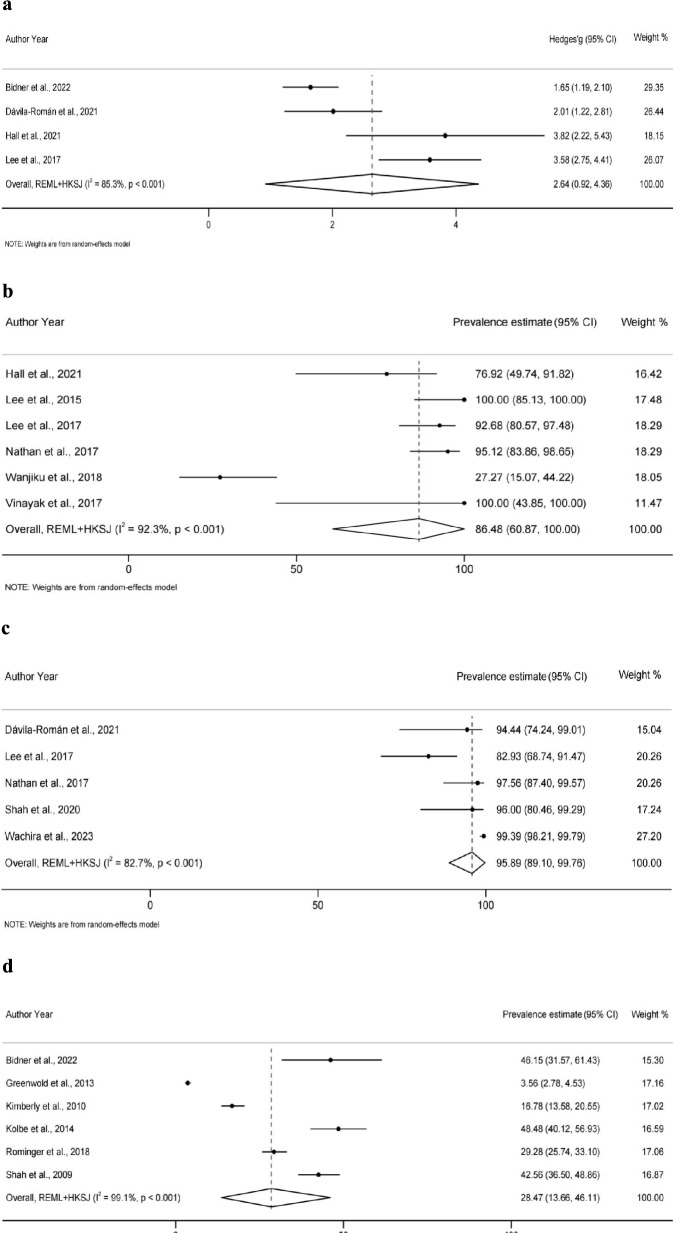
Meta-analysis forest plots of the effects of obstetric POCUS training. **a** Knowledge test scores (pre vs. post). **b** Knowledge pass rates. **c** Skill pass rates. **d** Impact on clinical decision-making. **e** Post-training use frequency

Knowledge acquisition was assessed in 11 studies (42.3%). Among them, 7 studies (26.9%) applied pre- and post-tests, reporting improvements such as the increase from 35.6 to 82.1% in Lee et al. [46]. A meta-analysis of 4 studies showed a pooled effect size (Hedges’ *g*) of 2.64 (95% CI 0.92–4.36), corresponding to an average increase of 31 points (Fig. 3a). Reported pass rates ranged from 27.27 to 100% (Fig. 3b), with meta-analysis yielding an overall pass rate of 86.48% (95% CI 60.87–100%).

Skills acquisition was evaluated in 11 studies (42.3%). Five studies (19.2%) reported pre-post improvements in scanning or interpretation performance. Another 5 studies (19.2%) reported skills-related pass rates ranging from 82.93 to 99.44% (Fig. 3c), and meta-analysis showed an overall pass rate of 95.89% (95% CI 89.10–99.76). Additional outcomes included scan time optimization in 2 studies (7.7%) and task success rates from 12 to 100% in 4 studies (15.4%). Knowledge and skill retention were assessed in 7 studies (26.9%), which generally showed performance remained above baseline, though some decline over time was noted.

Practical application of skills was evaluated in 18 studies (69.2%), spanning five thematic areas. Scan volume increases were reported in 5 studies (19.2%). Clinical decision-making changed in 28.47% of cases (95% CI 13.66–46.11), based on findings from 6 studies (Fig. 3d). Continued use of POCUS following training was reported in 3 studies (Fig. 3e), with a pooled usage rate of 83.57% (95% CI 62.78–97.43). In addition, 5 studies (19.2%) described user feedback and program feasibility, and all 10 studies (38.5%) assessing learner confidence reported improvements.

##### Health impact assessment

Beyond individual learning outcomes, the true test of these training programs lies in their impact on maternal and neonatal health services. Maternal and neonatal health impacts were assessed in 7 studies (26.9%). One study reported a 70% increase in antenatal care attendance. Screening for high-risk pregnancies identified breech (13.3%), transverse (4.8%), twins (2.1%), and placenta previa presentations (1.3%). Psychological outcomes were also described, including reduced anxiety through fetal visualization. In addition, health economic outcomes were reported in 4 studies (15.4%). These included reduced referrals and decreased imaging utilization, contributing to institutional cost-effectiveness. Furthermore, 74% of trainees reported improved resource use, and 43% of ultrasound examinations led to changes in patient management, such as avoiding unnecessary referrals or further imaging.

## Discussion

This systematic review applies the *ADDIE* model to analyze 26 obstetric POCUS training studies for the first time. Through systematic synthesis, we found that POCUS training enhances healthcare providers’ capabilities, improves maternal and neonatal health outcomes, and generates health economic benefits. However, while existing training programs incorporate various *ADDIE* model phases, there remains room for improvement in the systematization of needs assessment, flexibility of implementation processes, and comprehensiveness of outcome evaluation. By introducing the *ADDIE* model for systematic analysis, this study not only validates the effectiveness of obstetric POCUS training but also reveals the mechanisms underlying this effectiveness. These findings provide theoretical and practical guidance for designing and implementing standardized training systems in resource-limited settings, ultimately enhancing primary healthcare service capacity and improving maternal health outcomes.

Five previous reviews focused on obstetric POCUS training [7, 12, 14, 15, 51]. Among these, Bidner et al.’s study [12] is most directly relevant, using the *Kirkpatrick* framework to evaluate 27 studies that assessed knowledge, skills, and clinical impacts. While this review provided a comprehensive summary of the content related to obstetric POCUS training, it lacked quantitative analysis. Abrokwa et al. [7] and Eppel et al. [14] included obstetric content, but neither specifically targeted obstetric training. The former evaluated clinical decisions from a task-shifting perspective without assessing knowledge or skills, while the latter assessed knowledge, confidence, and skills without addressing clinical impacts. Luntsi et al. [51] discussed service accessibility, and Singh et al. [15] focused on emergency simulation, neither substantially evaluating obstetric POCUS training effectiveness. Consequently, few reviews specifically address obstetric POCUS training. Existing studies lack comprehensive quantitative evidence and a unified theoretical framework explaining how training elements interact to produce outcomes. By introducing *ADDIE* model, our study achieves a comprehensive quantitative assessment at three levels. At the individual level, we provide quantitative evidence on knowledge acquisition, skills development, POCUS usage frequency post-training, and changes in clinical decision-making rates. At the systemic level, we evaluate indicators of diagnostic accuracy and image quality. Furthermore, at the institutional level, we offer data on improvements in antenatal care attendance, the identification of high-risk pregnancies, and the associated health economic benefits.

To deeply analyze the mechanisms underlying these positive outcomes, we found that although these studies weren’t designed according to *ADDIE* model, their successful elements align remarkably with its core principles. The *ADDIE* model (*Analysis*, *Design*, *Development*, *Implementation*, *Evaluation*), initially developed by the U.S. military in the 1970s, has become a classic medical education framework [17]. Analyzing these studies through the *ADDIE* model reveals three core mechanisms driving positive outcomes. First, systematic design ensures training integrity and coherence. Successful programs universally incorporate complete design elements where needs identification (*Analysis*) guides targeted design (*Design*), which translates into resources (*Development*), supporting effective execution (*Implementation*), with experience feeding back through *Evaluation* into the next cycle. Second, flexibility and adaptability ensure effectiveness across diversified environments. The *ADDIE* model’s iterative nature enables adjustment to different contexts and implementation outcomes, explaining success despite varying resources. Third, positive feedback loops promote sustainability and scalability. When trainees master POCUS and apply it clinically, this success enhances confidence and motivation [52, 53], encouraging continued practice. This consolidates skills and internalizes technology into stable clinical behaviors, expanding effects from individual capacity to system-wide improvements. These findings clarify the pathway for designing successful obstetric POCUS training. The key is transforming the *ADDIE* model from an analytical tool to a design guide. The following sections detail essential elements and implementation points of each phase of the *ADDIE* model, providing evidence-based guidance for training designers.

The *Analysis* phase serves as the cornerstone for training success, encompassing four core elements, including trainee groups, needs analysis, baseline survey methods, and impact on instructional design. Trainee group identification ensures participants have clear application scenarios and learning motivation, with frontline personnel such as physicians, nurses, and midwives becoming ideal candidates due to their direct patient contact [54]. Multi-level needs analysis spans national public health objectives, institutional service capacity, and individual professional development, serving both macro-level goals and individual needs. Baseline surveys capture learners’ starting points through various assessment tools, providing benchmarks for personalized instruction and outcome comparison. Most critically, analysis results must transform into specific instructional strategies, including content stratification and differentiated teaching approaches. The integration of these elements ensures training possesses a clear direction and targeting from the outset. The *Design* phase transforms needs into instructional plans through systematic planning, comprising three core elements including training objectives, training methods, and training content. Training objectives have evolved from single-skill training to three-dimensional integration of knowledge, skills, and attitudes, producing synergistic effects. Knowledge provides a theoretical foundation, skills ensure operational proficiency, and attitudes overcome psychological barriers, promoting technology adoption [52]. Training methods predominantly feature blended learning approaches commonly used in medical education [55] while content adopts progressive, modular structures ensuring both systematic coverage and clinical relevance. This comprehensive design ensures training achieves both theoretical depth and practical applicability.

The *Development* phase bridges instructional design and concrete resources, with configuration strategies directly affecting feasibility and sustainability. This phase encompasses three core elements, including instructors, teaching aids, and teaching materials. Instructors emphasize theory–practice integration, with clinical teachers providing practical experience and academic teachers ensuring theoretical depth. Localized faculty strategies prove crucial for sustainability as these instructors understand local contexts and can continue training after external support withdraws [7]. All included studies utilized portable POCUS devices as teaching tools, validating their feasibility across different geographical and economic contexts [56]. Teaching materials reflect flexibility through both original content based on local needs and adapted international materials, while remote feedback systems extend expert guidance technologically. This optimized allocation ensures implementability across various conditions. The *Implementation* phase transforms preparation into practice through flexible execution. This phase comprises five core elements, including time, locations, participants, the execution process, and adaptation records. Adaptive adjustment mechanisms prove crucial [57], enabling dynamic optimization based on learner feedback and environmental changes through adjusted teaching pace, modified instructional approaches, or flexible equipment solutions. Training duration must consider skill formation patterns, providing time for complex competency development [58]. The execution process encompasses preparation, theoretical learning, skill training, and clinical application. Although post-training support is not mandatory, it provides considerable value by reinforcing outcomes through ongoing guidance and mentorship. This approach, balancing structure with flexibility, ensures training adapts to different environments while producing sustained effects.

The *Evaluation* phase examines training effectiveness and guides continuous improvement through a multidimensional assessment that validates effectiveness and reveals how *ADDIE* model’s systematic design translates into substantive outcomes. This phase encompasses two core elements, including training effectiveness evaluation and health impact assessment. Training effectiveness evaluation focuses on individual-level capacity changes across knowledge acquisition, skill proficiency, sustained application, and clinical decision-making. Knowledge and skill assessments verify instructional objectives achievement, sustained application rates reflect practicality and acceptance, while clinical decision changes demonstrate medical practice impact [59]. Health impact assessment extends to system and institutional levels, encompassing maternal-neonatal health improvements and economic benefits. These dual dimensions are essential because effective medical training must demonstrate both clinical value through improved health outcomes and sustainability through economic efficiency [60, 61], ensuring that training investments translate into lasting healthcare system improvements that can be maintained and scaled in resource-constrained environments. This dimension recognizes that medical training’s ultimate purpose extends beyond individual capabilities to improving health service quality and population health.

This assessment framework demonstrates how training guided by the *ADDIE* model produces progressive positive effects, validating that well-designed programs achieve value transformation from capacity building to health improvement. This approach offers particular value for resource-limited settings facing faculty shortages, training costs, human resource constraints, and geographic dispersion [62], where the *ADDIE* model can enable sustainable training through local faculty development, portable equipment use, and adaptive implementation despite infrastructure challenges. By providing a replicable and sustainable framework for establishing standardized training systems, future obstetric POCUS training should fully leverage *ADDIE* model while adapting to local contexts, ultimately serving the common goal of improving global maternal and neonatal health.

This study has three key strengths. First, as the first systematic review of obstetric POCUS training based on the *ADDIE* model, we achieved a comprehensive analysis across disciplines and resource settings through innovative methodology. Second, we established a complete evidence chain from training design to outcomes, quantifying key indicators including knowledge improvement effect sizes, skill mastery rates, and clinical decision change proportions. Third, our systematic framework analysis revealed not only training effectiveness but also the mechanisms of why and how training works, providing theoretical and practical guidance for low-resource settings. However, certain limitations also exist. The meta-analysis results showed substantial heterogeneity, possibly due to limited study numbers in each analysis. To address this issue, we employed a mixed-methods approach that integrates meta-analysis, where applicable, with narrative synthesis and the *ADDIE* model for systematic organization and interpretation of the heterogeneous evidence. Furthermore, retrospective mapping to the *ADDIE* model required subjective judgment when original studies inadequately described training processes. To optimize this procedure, we minimized bias through dual independent extraction, detailed mapping guidelines, and team consensus.

## Conclusion

This systematic review provides the first comprehensive analysis of obstetric POCUS training using the *ADDIE* model. Our findings confirm that POCUS training is effective in enhancing healthcare providers’ capabilities, improving maternal and neonatal health outcomes, and generating economic benefits. By mapping 26 studies to the *ADDIE* model, we identified essential elements for each phase and revealed the mechanisms driving training success. This analysis offers evidence-based guidance for designing more effective obstetric POCUS training programs, especially in resource-limited settings where systematic approaches can maximize impact on global health outcomes.

## Supplementary Information

Additional file 1.

Additional file 2.

Additional file 3.

## Data Availability

Our research team conducted systematic searches across several databases, clinical trial registries, and grey literature sources. The search results were imported into EndnoteX9 and the Covidence online platform for deduplication and screening. Subsequently, all statistical analyses were performed using the STATA18.0 (StataCorp LLC, Texas, USA), with no custom code developed specifically for the analyses conducted in this study. Therefore, the data supporting the findings are accessible within the previous published literature, as well as in the manuscript, tables, figures and supplementary materials. For additional inquiries or clarifications, please feel free to contact the corresponding author, Prof. Kun TANG.

## Abbreviations

ADDIE: Analyze, Design, Develop, Implement, Evaluate
CENTRAL: Cochrane Central Register of Controlled Trials
CINAHL Plus: Cumulative Index to Nursing and Allied Health Literature plus
CIs: Confidence intervals
ClinicalTrials.gov: ClinicalTrials.gov database
CNKI: China National Knowledge Infrastructure
CNKI Dissertation Database: China Doctoral Dissertations & Master’s Theses Full-text Database
CQVIP: Chinese Scientific Journals Database
Embase: Excerpta Medica Database
HICs: High-income countries
HKSJ: Hartung–Knapp–Sidik–Jonkman
ICTRP: International Clinical Trials Registry Platform
JBI: Joanna Briggs Institute
LMICs: Low- and middle-income countries
MeSH: Medical subject headings
PIOS: Population, intervention, outcomes, study design
POCUS: Point-of-care ultrasound
PRISMA: Preferred Reporting Items for Systematic Reviews and Meta-Analyses
ProQuest Dissertations: ProQuest Dissertations & Theses
PROSPERO: International Prospective Register of Systematic Reviews
PubMed: National Library of Medicine’s Database
RCTs: Randomized controlled trials
REML: Restricted maximum likelihood
Scopus: Elsevier’s Abstract and Citation Database
SinoMed: Chinese Biomedical Literature Database
SMD: Standardized mean difference
STATA: Stata Statistical Software
WanFang: WanFang Data Knowledge Service Platform
Web of Science: Web of Science Core Collection Database
WHO ICTRP: World Health Organization International Clinical Trials Registry Platform
