# Supplementary material for: Effectiveness of obstetric point-of-care ultrasound (POCUS) training: a systematic review and meta-analysis based on the ADDIE training model
Source: Ultrasound J. 2025 Dec 31;17:67. doi: 10.1186/s13089-025-00471-z (PMC12756214; doi:10.1186/s13089-025-00471-z)
Supplement: Supplementary file 1 — Additional file 1. [file 13089_2025_471_MOESM1_ESM.docx]

Appendix I - Supplementary Tables

**Catalog of Supplementary** **Tables**

| **No.** | **Document** | **Page** |
| --- | --- | --- |
| 1 | Supplementary Table 1: *PRISMA2020* Checklist | Pages 1-4 |
| 2 | Supplementary Table 2: Specific Search Strategies and Results of Each Database/Register | Pages 5-10 |
| 3 | Supplementary Table 3: Details of Quality Evaluation Process | Pages 11-14 |

| **Supplementary Table 1: *PRISMA2020* Checklist** | | | |
| --- | --- | --- | --- |
| **Section and Topic** | **Item #** | **Checklist item** | **Location where item is reported** |
| **TITLE** | | |  |
| Title | 1 | Identify the report as a systematic review. | P1. Title |
| **ABSTRACT** | | |  |
| Abstract | 2 | See the PRISMA 2020 for Abstracts checklist. | P1. Abstract |
| **INTRODUCTION** | | |  |
| Rationale | 3 | Describe the rationale for the review in the context of existing knowledge. | P2-3. 1. Introduction |
| Objectives | 4 | Provide an explicit statement of the objective(s) or question(s) the review addresses. | P3. 1. Introduction |
| **METHODS** | | |  |
| Eligibility criteria | 5 | Specify the inclusion and exclusion criteria for the review and how studies were grouped for the syntheses. | P4. 2.2 Eligibility Criteria and Study Selection & Table 1 |
| Information sources | 6 | Specify all databases, registers, websites, organisations, reference lists and other sources searched or consulted to identify studies. Specify the date when each source was last searched or consulted. | P3-4. 2.1 Search Strategy |
| Search strategy | 7 | Present the full search strategies for all databases, registers and websites, including any filters and limits used. | P3-4. 2.1 Search Strategy & Supplementary Table 2 |
| Selection process | 8 | Specify the methods used to decide whether a study met the inclusion criteria of the review, including how many reviewers screened each record and each report retrieved, whether they worked independently, and if applicable, details of automation tools used in the process. | P4. 2.2 Eligibility Criteria and Study Selection |
| Data collection process | 9 | Specify the methods used to collect data from reports, including how many reviewers collected data from each report, whether they worked independently, any processes for obtaining or confirming data from study investigators, and if applicable, details of automation tools used in the process. | P5. 2.4 Data Extraction |
| Data items | 10a | List and define all outcomes for which data were sought. Specify whether all results that were compatible with each outcome domain in each study were sought (e.g. for all measures, time points, analyses), and if not, the methods used to decide which results to collect. | P5. 2.4 Data Extraction |
|  | 10b | List and define all other variables for which data were sought (e.g. participant and intervention characteristics, funding sources). Describe any assumptions made about any missing or unclear information. | P5. 2.4 Data Extraction & Supplementary Files 1-2 |
| Study risk of bias assessment | 11 | Specify the methods used to assess risk of bias in the included studies, including details of the tool(s) used, how many reviewers assessed each study and whether they worked independently, and if applicable, details of automation tools used in the process. | P5. 2.3 Quality Assessment |
| Effect measures | 12 | Specify for each outcome the effect measure(s) (e.g. risk ratio, mean difference) used in the synthesis or presentation of results. | P5-6. 2.5 Statistical Analysis |
| Synthesis methods | 13a | Describe the processes used to decide which studies were eligible for each synthesis (e.g. tabulating the study intervention characteristics and comparing against the planned groups for each synthesis (item #5)). | P5-6. 2.5 Statistical Analysis |
|  | 13b | Describe any methods required to prepare the data for presentation or synthesis, such as handling of missing summary statistics, or data conversions. | P5-6. 2.5 Statistical Analysis |
|  | 13c | Describe any methods used to tabulate or visually display results of individual studies and syntheses. | P5-6. 2.5 Statistical Analysis & Table 2 & Supplementary Table 3 |
|  | 13d | Describe any methods used to synthesize results and provide a rationale for the choice(s). If meta-analysis was performed, describe the model(s), method(s) to identify the presence and extent of statistical heterogeneity, and software package(s) used. | P5-6. 2.5 Statistical Analysis |
|  | 13e | Describe any methods used to explore possible causes of heterogeneity among study results (e.g. subgroup analysis, meta-regression). | P5-6. 2.5 Statistical Analysis |
|  | 13f | Describe any sensitivity analyses conducted to assess robustness of the synthesized results. | P5-6. 2.5 Statistical Analysis |
| Reporting bias assessment | 14 | Describe any methods used to assess risk of bias due to missing results in a synthesis (arising from reporting biases). | P5-6. 2.5 Statistical Analysis |
| Certainty assessment | 15 | Describe any methods used to assess certainty (or confidence) in the body of evidence for an outcome. | P5-6. 2.5 Statistical Analysis |
| **RESULTS** | | |  |
| Study selection | 16a | Describe the results of the search and selection process, from the number of records identified in the search to the number of studies included in the review, ideally using a flow diagram. | P6. 3.1 Study Selection and Quality Assessment Results & Figure 1 & Supplementary Files 3-5 |
|  | 16b | Cite studies that might appear to meet the inclusion criteria, but which were excluded, and explain why they were excluded. | P6. 3.1 Study Selection and Quality Assessment Results & Supplementary Files 3-5 |
| Study characteristics | 17 | Cite each included study and present its characteristics. | P8. 3.2 Basic Characteristics of Included Studies & Table 3 & Supplementary File 6 |
| Risk of bias in studies | 18 | Present assessments of risk of bias for each included study. | P6. 3.1 Study Selection and Quality Assessment Results & Table 2 & Supplementary Table 3 |
| Results of individual studies | 19 | For all outcomes, present, for each study: (a) summary statistics for each group (where appropriate) and (b) an effect estimate and its precision (e.g. confidence/credible interval), ideally using structured tables or plots. | P9-15. 3.3 Narrative Review Based on *ADDIE* Model & Figures 3 |
| Results of syntheses | 20a | For each synthesis, briefly summarise the characteristics and risk of bias among contributing studies. | P9-15. 3.3 Narrative Review Based on *ADDIE* Model & Figures 3 |
|  | 20b | Present results of all statistical syntheses conducted. If meta-analysis was done, present for each the summary estimate and its precision (e.g. confidence/credible interval) and measures of statistical heterogeneity. If comparing groups, describe the direction of the effect. | P9-15. 3.3 Narrative Review Based on *ADDIE* Model & Figures 3 |
|  | 20c | Present results of all investigations of possible causes of heterogeneity among study results. | P9-15. 3.3 Narrative Review Based on *ADDIE* Model & Figures 3 |
|  | 20d | Present results of all sensitivity analyses conducted to assess the robustness of the synthesized results. | P9-15. 3.3 Narrative Review Based on *ADDIE* Model & Figures 3 |
| Reporting biases | 21 | Present assessments of risk of bias due to missing results (arising from reporting biases) for each synthesis assessed. | P9-15. 3.3 Narrative Review Based on *ADDIE* Model & Figures 3 |
| Certainty of evidence | 22 | Present assessments of certainty (or confidence) in the body of evidence for each outcome assessed. | P9-15. 3.3 Narrative Review Based on *ADDIE* Model & Figures 3 |
| **DISCUSSION** | | |  |
| Discussion | 23a | Provide a general interpretation of the results in the context of other evidence. | P15-18. 4. Discussion |
|  | 23b | Discuss any limitations of the evidence included in the review. | P18. 4. Discussion |
|  | 23c | Discuss any limitations of the review processes used. | P18. 4. Discussion |
|  | 23d | Discuss implications of the results for practice, policy, and future research. | P18. 5. Conclusion |
| **OTHER INFORMATION** | | |  |
| Registration and protocol | 24a | Provide registration information for the review, including register name and registration number, or state that the review was not registered. | P3. 2. Methods |
|  | 24b | Indicate where the review protocol can be accessed, or state that a protocol was not prepared. | P3. 2. Methods |
|  | 24c | Describe and explain any amendments to information provided at registration or in the protocol. | Unchanged |
| Support | 25 | Describe sources of financial or non-financial support for the review, and the role of the funders or sponsors in the review. | P35. Funding |
| Competing interests | 26 | Declare any competing interests of review authors. | P35. Competing Interests |
| Availability of data, code and other materials | 27 | Report which of the following are publicly available and where they can be found: template data collection forms; data extracted from included studies; data used for all analyses; analytic code; any other materials used in the review. | Title Page |

*From:*  Page MJ, McKenzie JE, Bossuyt PM, Boutron I, Hoffmann TC, Mulrow CD, et al. The PRISMA 2020 statement: an updated guideline for reporting systematic reviews. BMJ 2021;372:n71. doi: 10.1136/bmj.n71. This work is licensed under CC BY 4.0. To view a copy of this license, visit <https://creativecommons.org/licenses/by/4.0/>

**Supplementary Table 2: Specific Search Strategies and Results of Each Database/Register**

**Search Date: From Inception to 22^nd^ September 2024**

| **Database/Register** | **Search Strategies** | **Results** |
| --- | --- | --- |
| PubMed | #1 "Obstetrics"[MeSH Terms] OR "Perinatal Care"[MeSH Terms] OR "Peripartum Period"[MeSH Terms] OR "Pregnancy"[MeSH Terms] OR "Pregnancy Trimesters"[MeSH Terms] OR "Prenatal Care"[MeSH Terms] OR "Prenatal Diagnosis"[MeSH Terms] OR "Pregnancy Tests"[MeSH Terms] OR "Pregnancy Complications"[MeSH Terms] OR "Pregnancy Outcome"[MeSH Terms] | 1,098,191 |
|  | #2 "Obstetric*"[Title/Abstract] OR "Perinatal"[Title/Abstract] OR "Peripartum"[Title/Abstract] OR "Pregnan*"[Title/Abstract] OR "Antenatal"[Title/Abstract] OR "Prenatal"[Title/Abstract] OR "Intranatal"[Title/Abstract] OR "Intrapartum"[Title/Abstract] OR "Postnatal"[Title/Abstract] OR "Postpartum"[Title/Abstract] OR "Puerperium"[Title/Abstract] | 972,430 |
|  | #3 #1 OR #2 | 1,417,447 |
|  | #4 "Point-of-Care Systems"[MeSH Terms] AND "Ultrasonography, Prenatal"[MeSH Terms] | 50 |
|  | #5 "Point of care ultraso*"[Title/Abstract] OR "POCUS"[Title/Abstract] OR "Portable ultraso*"[Title/Abstract] OR "Compact ultraso*"[Title/Abstract] OR "Pocket size* ultraso*"[Title/Abstract] OR "Handheld ultraso*"[Title/Abstract] OR "Bedside ultraso*"[Title/Abstract] OR "Real time ultraso*"[Title/Abstract] OR "Rapid ultraso*"[Title/Abstract] OR "Wireless ultraso*"[Title/Abstract] OR "Mobile ultraso*"[Title/Abstract] | 12,491 |
|  | #6 #4 OR #5 | 12,509 |
|  | #7 "Competency-Based Education"[MeSH Terms] OR "Problem-Based Learning"[MeSH Terms] OR "Education, Continuing"[MeSH Terms] OR "Education, Medical"[MeSH Terms] OR "Education, Nursing"[MeSH Terms] OR "Education, Public Health Professional"[MeSH Terms] OR "Mentoring"[MeSH Terms] OR "Professional Competence"[MeSH Terms] OR "Self-Evaluation Programs"[MeSH Terms] OR "Inservice Training"[MeSH Terms] OR "Simulation Training"[MeSH Terms] | 408,970 |
|  | #8 "Evaluat*"[Title/Abstract] OR "Educat*"[Title/Abstract] OR "Train*"[Title/Abstract] OR "Workshop*"[Title/Abstract] OR "Skill*"[Title/Abstract] OR "Knowledge*"[Title/Abstract] | 6,772,588 |
|  | #9 #7 OR #8 | 6,934,968 |
|  | #10 #3 AND #6 AND #9 | 487 |
| Embase | #1 'obstetrics'/exp OR 'perinatal care'/exp OR 'pregnancy'/exp OR 'prenatal diagnosis'/exp OR 'pregnancy test'/exp OR 'pregnancy complication'/exp OR 'pregnancy outcome'/exp | 1,420,098 |
|  | #2 obstetric*:ti,ab,kw OR perinatal:ti,ab,kw OR peripartum:ti,ab,kw OR pregnan*:ti,ab,kw OR antenatal:ti,ab,kw OR prenatal:ti,ab,kw OR intranatal:ti,ab,kw OR intrapartum:ti,ab,kw OR postnatal:ti,ab,kw OR postpartum:ti,ab,kw OR puerperium:ti,ab,kw | 1,286,492 |
|  | #3 #1 OR #2 | 1,841,827 |
|  | #4 'point of care ultrasound'/exp | 5,556 |
|  | #5 'point of care ultraso*':ti,ab,kw OR 'pocus':ti,ab,kw OR 'portable ultraso*':ti,ab,kw OR 'compact ultraso*':ti,ab,kw OR 'pocket size* ultraso*':ti,ab,kw OR 'handheld ultraso*':ti,ab,kw OR 'bedside ultraso*':ti,ab,kw OR 'real-time ultraso*':ti,ab,kw OR 'rapid ultraso*':ti,ab,kw OR 'wireless ultraso*':ti,ab,kw OR 'mobile ultraso*':ti,ab,kw | 17,350 |
|  | #6 #4 OR #5 | 18,825 |
|  | #7 'teaching'/exp OR 'training'/exp OR 'continuing education'/exp OR 'curriculum'/exp OR 'curriculum development'/exp OR 'education program'/exp OR 'educational model'/exp OR 'educational technology'/exp OR 'health education'/exp OR 'in service training'/exp OR 'medical education'/exp OR 'outcome of education'/exp OR 'problem based learning'/exp OR 'simulation training'/exp OR 'teacher training'/exp | 1,114,026 |
|  | #8 'evaluat*':ti,ab,kw OR 'educat*':ti,ab,kw OR 'train*':ti,ab,kw OR 'workshop*':ti,ab,kw OR 'skill*':ti,ab,kw OR 'knowledge*':ti,ab,kw | 9,169,737 |
|  | #9 #7 OR #8 | 9,618,268 |
|  | #10 #3 AND #6 AND #9 | 800 |
| CINAHL Plus | #1 MH “Obstetrics” OR MH “Perinatal Care” OR MH “Perinatal Period” OR MH “Pregnancy” OR MH “Pregnancy Trimesters” OR MH “Prenatal Care” OR MH “Prenatal Diagnosis” OR MH “Pregnancy Tests” OR MH “Pregnancy Complications” OR MH “Pregnancy Outcomes” OR MH “Postnatal Care” | 249,135 |
|  | #2 SU “Obstetric*” OR SU “Perinatal” OR SU “Peripartum” OR SU “Pregnan*” OR SU “Antenatal” OR SU “Prenatal” OR SU “Intranatal” OR SU “Intrapartum” OR SU “Postnatal” OR SU “Postpartum” OR SU “Puerperium” | 277,306 |
|  | #3 #1 OR #2 | 277,306 |
|  | #4 MH “Point-of-Care Testing” AND MH “Ultrasonography, Prenatal” | 6 |
|  | #5 Point of care ultraso* OR POCUS OR Portable ultraso* OR Compact ultraso* OR Pocket size* ultraso* OR Handheld ultraso* OR Bedside ultraso* OR Real time ultraso* OR Rapid ultraso* OR Wireless ultraso* OR Mobile ultraso* | 4,279 |
|  | #6 #4 OR #5 | 4,281 |
|  | #7 MH “Education, Competency-Based” OR MH “Problem-Based Learning” OR MH “Education, Medical” OR MH “Education, Nursing” OR MH “Education, Midwifery” OR MH “Clinical Competence” OR MH “Program Evaluation” OR MH “Curriculum” OR MH “Models, Educational” OR MH “Educational Technology” OR MH “Health Education” OR MH “Outcomes of Education” OR MH “Problem-Based Learning” OR MH “Teaching” | 252,454 |
|  | #8 SU “Evaluat*” OR SU “Educat*” OR SU “Train*” OR SU “Workshop*” OR SU “Skill*” OR SU “Knowledge*” | 1,589,476 |
|  | #9 #7 OR #8 | 1,612,041 |
|  | #10 #3 AND #6 AND #9 | 61 |
| Web of Science | #1 TS=(Obstetric* OR Perinatal OR Peripartum OR Pregnan* OR Antenatal OR Prenatal OR Intranatal OR Intrapartum OR Postnatal OR Postpartum OR Puerperium) | 2,041,270 |
|  | #2 TS=("Point-of-care Ultraso*" OR "POCUS" OR "Portable Ultraso*" OR "Compact Ultraso*" OR "Pocket-size* Ultraso*" OR "Handheld Ultraso*" OR "Bedside Ultraso*" OR "Real-time Ultraso*" OR "Rapid Ultraso*" OR "Wireless Ultraso*" OR "Mobile Ultraso*") | 21,836 |
|  | #3 TS=(Evaluat* OR Educat* OR Train* OR Workshop* OR Skill* OR Knowledge*) | 21,254,970 |
|  | #4 #1 AND #2 AND #3 | 731 |
| Scopus | #1 TITLE-ABS-KEY ("Obstetric*" OR "Perinatal" OR "Peripartum" OR "Pregnan*" OR "Antenatal" OR "Prenatal" OR "Intranatal" OR "Intrapartum" OR "Postnatal" OR "Postpartum" OR "Puerperium") | 1,718,004 |
|  | #2 TITLE-ABS-KEY("Point-of-care Ultraso*" OR "POCUS" OR "Portable Ultraso*" OR "Compact Ultraso*" OR "Pocket-size* Ultraso*" OR "Handheld Ultraso*" OR "Bedside Ultraso*" OR "Real-time Ultraso*" OR "Rapid Ultraso*" OR "Wireless Ultraso*" OR "Mobile Ultraso*") | 19,834 |
|  | #3 TITLE-ABS-KEY("Evaluat*" OR "Educat*" OR "Train*" OR "Workshop*" OR "Skill*" OR "Knowledge*") | 17,499,286 |
|  | #4 #1 AND #2 AND #3 | 789 |
| CNKI | #1 SU = "护理点超声" + "便携超声" + "便携式超声" + "手持超声" + "手持式超声" + "掌上超声" + "口袋式超声" + "床旁超声" + "床边超声" + "即时床旁超声" + "即时超声" + "实时超声" + "快速超声" + "床旁即时超声" + "无线超声" + "移动超声" | 4,638 |
|  | #2 SU = "教育" + "教学" + "培训" + "技能" | 5,660,722 |
|  | #3 #1 AND #2 | 48 |
| WanFang | #1 主题:("护理点超声") OR 主题:("便携超声") OR 主题:("便携式超声") OR 主题:("手持超声") OR 主题:("手持式超声") OR 主题:("掌上超声") OR 主题:("口袋式超声") OR 主题:("床旁超声") OR 主题:("床边超声") OR 主题:("即时床旁超声") OR 主题:("即时超声") OR 主题:("实时超声") OR 主题:("快速超声") OR 主题:("床旁即时超声") OR 主题:("无线超声") OR 主题:("移动超声") | 5,498 |
|  | #2 主题:("教育") OR 主题:("教学") OR 主题:("培训") OR 主题:("技能") | 13,550,666 |
|  | #3 #1 AND #2 | 123 |
| CQVIP | #1 M="护理点超声" OR M="便携超声" OR M="便携式超声" OR M="手持超声" OR M="手持式超声" OR M="掌上超声" OR M="口袋式超声" OR M="床旁超声" OR M="床边超声" OR M="即时床旁超声" OR M="即时超声" OR M="实时超声" OR M="快速超声" OR M="床旁即时超声" OR M="无线超声" OR M="移动超声" | 5,639 |
|  | #2 M="教育" OR M="教学" OR M="培训" OR M="技能" | 7,457,481 |
|  | #3 #1 AND #2 | 54 |
| SinoMed | #1 "床旁诊断化验系统"[不加权:扩展] AND "超声检查, 产前"[不加权:扩展] | 0 |
|  | #2 "护理点超声"[常用字段:智能] OR "便携超声"[常用字段:智能] OR "便携式超声"[常用字段:智能] OR "手持超声"[常用字段:智能] OR "手持式超声"[常用字段:智能] OR "掌上超声"[常用字段:智能] OR "口袋式超声"[常用字段:智能] OR "床旁超声"[常用字段:智能] OR "床边超声"[常用字段:智能] OR "即时床旁超声"[常用字段:智能] OR "即时超声"[常用字段:智能] OR "实时超声"[常用字段:智能] OR "快速超声"[常用字段:智能] OR "床旁即时超声"[常用字段:智能] OR "无线超声"[常用字段:智能] OR "移动超声"[常用字段:智能] | 4,512 |
|  | #3 #1 OR #2 | 4,512 |
|  | #4 "教育，继续"[不加权:扩展] OR "教育，医学"[不加权:扩展] OR "教育，护理"[不加权:扩展] OR "教育，公共卫生专业"[不加权:扩展] OR "基于能力的教育"[不加权:扩展] OR "基于问题的学习"[不加权:扩展] OR "专业能力"[不加权:扩展] OR "自我评价方案"[不加权:扩展] OR "在职培训"[不加权:扩展] OR "模拟培训"[不加权:扩展] OR "教学"[不加权:扩展] | 175,995 |
|  | #5 "教育"[常用字段:智能] OR "教学"[常用字段:智能] OR "培训"[常用字段:智能] OR "技能"[常用字段:智能] | 794,649 |
|  | #6 #4 OR #5 | 794,649 |
|  | #7 #3 AND #6 | 186 |
| WHO ICTRP | Condition: ("Obstetric" OR "Perinatal" OR "Peripartum" OR "Pregnancy" OR "Antenatal" OR "Prenatal" OR "Intranatal" OR "Intrapartum" OR "Postnatal" OR "Postpartum" OR "Puerperium") \| Intervention: ("Point of care ultrasound" OR "POCUS" OR "Portable ultrasound" OR "Compact ultrasound" OR "Pocket size ultrasound" OR "Handheld ultrasound" OR "Bedside ultrasound" OR "Real time ultrasound" OR "Rapid ultrasound" OR "Wireless ultrasound" OR "Mobile ultrasound") AND ("Evaluate" OR "Educate" OR "Train" OR "Workshop" OR "Skill" OR "Knowledge") | 6 |
| Cochrane Central database | #1 MeSH descriptor: [Obstetrics] explode all trees | 338 |
|  | #2 MeSH descriptor: [Perinatal Care] explode all trees | 835 |
|  | #3 MeSH descriptor: [Peripartum Period] explode all trees | 50 |
|  | #4 MeSH descriptor: [Pregnancy] explode all trees | 33,459 |
|  | #5 MeSH descriptor: [Pregnancy Trimesters] explode all trees | 2,347 |
|  | #6 MeSH descriptor: [Prenatal Care] explode all trees | 2,325 |
|  | #7 MeSH descriptor: [Prenatal Diagnosis] explode all trees | 1,227 |
|  | #8 MeSH descriptor: [Pregnancy Tests] explode all trees | 40 |
|  | #9 MeSH descriptor: [Pregnancy Complications] explode all trees | 17,459 |
|  | #10 MeSH descriptor: [Pregnancy Outcome] explode all trees | 5,047 |
|  | #11 #1 OR #2 OR #3 OR #4 OR #5 OR #6 OR #7 OR #8 OR #9 OR #10 | 38,268 |
|  | #12 (Obstetric*):ti,ab,kw OR (Perinatal):ti,ab,kw OR (Peripartum):ti,ab,kw OR (Pregnan*):ti,ab,kw OR (Antenatal):ti,ab,kw OR (Prenatal):ti,ab,kw OR (Intranatal):ti,ab,kw OR (Intrapartum):ti,ab,kw OR (Postnatal):ti,ab,kw OR (Postpartum):ti,ab,kw OR (Puerperium):ti,ab,kw | 105,915 |
|  | #13 #11 OR #12 | 107,622 |
|  | #14 MeSH descriptor: [Point-of-Care Systems] explode all trees | 836 |
|  | #15 MeSH descriptor: [Ultrasonography, Prenatal] explode all trees | 790 |
|  | #16 #14 AND #15 | 1 |
|  | #17 (Point of care ultraso*):ti,ab,kw OR (POCUS):ti,ab,kw OR (Portable ultraso*):ti,ab,kw OR (Compact ultraso*):ti,ab,kw OR (Pocket size* ultraso*):ti,ab,kw OR (Handheld ultraso*):ti,ab,kw OR (Bedside ultraso*):ti,ab,kw OR (Real time ultraso*):ti,ab,kw OR (Rapid ultraso*):ti,ab,kw OR (Wireless ultraso*):ti,ab,kw OR (Mobile ultraso*):ti,ab,kw | 5,185 |
|  | #18 #16 OR #17 | 5,185 |
|  | #19 MeSH descriptor: [Competency-Based Education] explode all trees | 110 |
|  | #20 MeSH descriptor: [Problem-Based Learning] explode all trees | 566 |
|  | #21 MeSH descriptor: [Education, Continuing] explode all trees | 1,403 |
|  | #22 MeSH descriptor: [Education, Medical] explode all trees | 4,670 |
|  | #23 MeSH descriptor: [Education, Nursing] explode all trees | 1,275 |
|  | #24 MeSH descriptor: [Education, Public Health Professional] explode all trees | 3 |
|  | #25 MeSH descriptor: [Mentoring] explode all trees | 568 |
|  | #26 MeSH descriptor: [Professional Competence] explode all trees | 5,380 |
|  | #27 MeSH descriptor: [Self-Evaluation Programs] explode all trees | 36 |
|  | #28 MeSH descriptor: [Inservice Training] explode all trees | 976 |
|  | #29 MeSH descriptor: [Simulation Training] explode all trees | 1,708 |
|  | #30 #19 OR #20 OR #21 OR #22 OR #23 OR #24 OR #25 OR #26 OR #27 OR #28 | 10,834 |
|  | #31 (Evaluat*):ti,ab,kw OR (Educat*):ti,ab,kw OR (Train*):ti,ab,kw OR (Workshop*):ti,ab,kw OR (Skill*):ti,ab,kw OR (Knowledge*):ti,ab,kw | 841,113 |
|  | #32 #30 OR #31 | 841,850 |
|  | #33 #13 AND #18 AND #32 | 320 |
| ClinicalTrials.gov | Condition/disease: ("Obstetric" OR "Perinatal" OR "Peripartum" OR "Pregnancy" OR "Antenatal" OR "Prenatal" OR "Intranatal" OR "Intrapartum" OR "Postnatal" OR "Postpartum" OR "Puerperium") \| Intervention/treatment: ("Point of care ultrasound" OR "POCUS" OR "Portable ultrasound" OR "Compact ultrasound" OR "Pocket size ultrasound" OR "Handheld ultrasound" OR "Bedside ultrasound" OR "Real time ultrasound" OR "Rapid ultrasound" OR "Wireless ultrasound" OR "Mobile ultrasound") \| Other terms: ("Evaluate" OR "Educate" OR "Train" OR "Workshop" OR "Skill" OR "Knowledge") | 42 |
| ProQuest Dissertations &Theses Database | #1 mainsubject("Obstetric*" OR "Perinatal" OR "Peripartum" OR "Pregnan*" OR "Antenatal" OR "Prenatal" OR "Intranatal" OR "Intrapartum" OR "Postnatal" OR "Postpartum" OR "Puerperium") | 422,348 |
|  | #2 mainsubject(Point-of-care Ultraso*) OR mainsubject(POCUS) OR mainsubject(Portable Ultraso*) OR mainsubject(Compact Ultraso*) OR mainsubject(Pocket-size Ultraso*) OR mainsubject(Handheld Ultraso*) OR mainsubject(Bedside Ultraso*) OR mainsubject(Real time Ultraso*) OR mainsubject(Rapid Ultraso*) OR mainsubject(Wireless Ultraso*) OR mainsubject(Mobile Ultraso*) OR mainsubject(Small Ultraso*)OR mainsubject(Man-carried Ultraso*) OR mainsubject(Carry-on Ultraso*) | 6,711 |
|  | #3 mainsubject("Evaluat*" OR "Educat*" OR "Train*" OR "Workshop*" OR "Skill*" OR "Knowledge*") | 5,802,134 |
|  | #4 #1 AND #2 AND #3 | 34 |
| CNKI dissertation database | #1 SU = "护理点超声" + "便携超声" + "便携式超声" + "手持超声" + "手持式超声" + "掌上超声" + "口袋式超声" + "床旁超声" + "床边超声" + "即时床旁超声" + "即时超声" + "实时超声" + "快速超声" + "床旁即时超声" + "无线超声" + "移动超声" | 579 |
|  | #2 SU = "教育" + "教学" + "培训" + "技能" | 679,041 |
|  | #3 #1 AND #2 | 1 |
| WanFang dissertation database | #1 主题:("护理点超声") OR 主题:("便携超声") OR 主题:("便携式超声") OR 主题:("手持超声") OR 主题:("手持式超声") OR 主题:("掌上超声") OR 主题:("口袋式超声") OR 主题:("床旁超声") OR 主题:("床边超声") OR 主题:("即时床旁超声") OR 主题:("即时超声") OR 主题:("实时超声") OR 主题:("快速超声") OR 主题:("床旁即时超声") OR 主题:("无线超声") OR 主题:("移动超声") | 796 |
|  | #2 主题:("教育") OR 主题:("教学") OR 主题:("培训") OR 主题:("技能") | 688,701 |
|  | #3 #1 AND #2 | 26 |

**Total results (Search Date: From Inception to 22^nd^ September 2024)**

| **Database/Register** | **Results** |
| --- | --- |
| 1. PubMed | 487 |
| 2. Embase | 800 |
| 3. CINAHL Plus | 61 |
| 4. Web of Science | 731 |
| 5. Scopus | 789 |
| 6. CNKI | 48 |
| 7. WanFang | 123 |
| 8. CQVIP | 54 |
| 9. SinoMed | 186 |
| 10. WHO ICTRP | 6 |
| 11. Cochrane Central database | 320 |
| 12. ClinicalTrials.gov | 42 |
| 13. ProQuest Dissertations & Theses Database | 34 |
| 14. CNKI dissertation database | 1 |
| 15. WanFang dissertation database | 26 |
| Total | N=3,708 |

**Supplementary Table 3: Details of Quality Evaluation Process**

| **Study Design I: Quasi-Experimental Study (n=10)** | | | | | | | | | | | | | | | |
| --- | --- | --- | --- | --- | --- | --- | --- | --- | --- | --- | --- | --- | --- | --- | --- |
| **No.** | **Included Study** | | | **Assessor** | **Q1** | **Q2** | **Q3** | **Q4** | **Q5** | **Q6** | **Q7** | | **Q8** | **Q9** | **Overall Appraisal** |
| 1 | Cook et al., 2020 | | | 1 | Y | N | Y | Y | Y | Y | Y | | N/A | Y | Include |
|  |  |  |  | 2 | Y | N | Y | Y | Y | Y | Y | | N/A | Y | Include |
|  |  |  |  | Final Results | Y | N | Y | Y | Y | Y | Y | | N/A | Y | Include |
| 2 | Davila Roman et al., 2021 | | | 1 | Y | N | Y | Y | Y | Y | Y | | N/A | Y | Include |
|  |  |  |  | 2 | Y | N | Y | Y | Y | Y | Y | | N/A | Y | Include |
|  |  |  |  | Final Results | Y | N | Y | Y | Y | Y | Y | | N/A | Y | Include |
| 3 | Filler & Lettang, 2024 | | | 1 | Y | N | Y | Y | Y | Y | Y | | N/A | Y | Include |
|  |  |  |  | 2 | Y | N | Y | Y | Y | Y | Y | | N/A | Y | Include |
|  |  |  |  | Final Results | Y | N | Y | Y | Y | Y | Y | | N/A | Y | Include |
| 4 | Hall et al., 2021 | | | 1 | Y | N | Y | Y | Y | Y | Y | | N/A | Y | Include |
|  |  |  |  | 2 | Y | N | Y | Y | Y | Y | Y | | N/A | Y | Include |
|  |  |  |  | Final Results | Y | N | Y | Y | Y | Y | Y | | N/A | Y | Include |
| 5 | Kimberly et al., 2010 | | | 1 | Y | N | Y | Y | Y | Y | Y | | N/A | Y | Include |
|  |  |  |  | 2 | Y | N | Y | Y | Y | Y | Y | | Y | Y | Include |
|  |  |  |  | Final Results | Y | N | Y | Y | Y | Y | Y | | Y | Y | Include |
| 6 | Kolbe et al., 2015 | | | 1 | Y | N | Y | Y | Y | Y | Y | | N/A | Y | Include |
|  |  |  |  | 2 | Y | N | Y | Y | Y | Y | Y | | N/A | Y | Include |
|  |  |  |  | Final Results | Y | N | Y | Y | Y | Y | Y | | N/A | Y | Include |
| 7 | Lee et al., 2015 | | | 1 | Y | N | Y | Y | Y | Y | Y | | N/A | Y | Include |
|  |  |  |  | 2 | Y | N | Y | Y | Y | Y | Y | | N/A | Y | Include |
|  |  |  |  | Final Results | Y | N | Y | Y | Y | Y | Y | | N/A | Y | Include |
| 8 | | Vinayak & Sharon Brownie, 2018 | 1 | | Y | N | Y | Y | Y | Y | | Y | N/A | Y | Include |
|  |  |  | 2 | | Y | N | Y | Y | Y | Y | | Y | N/A | Y | Include |
|  |  |  | Final Results | | Y | N | Y | Y | Y | Y | | Y | N/A | Y | Include |
| 9 | | Wachira et al., 2023 | 1 | | Y | N | Y | Y | Y | Y | | Y | N/A | Y | Include |
|  |  |  | 2 | | Y | N | Y | Y | Y | Y | | Y | N/A | Y | Include |
|  |  |  | Final Results | | Y | N | Y | Y | Y | Y | | Y | N/A | Y | Include |

**Supplementary Table 3: Details of Quality Evaluation Process *(Continued)***

| **Study Design I: Quasi-Experimental Study (n=10)** | | | | | | | | | | | | | | | | | | | | | | | | |
| --- | --- | --- | --- | --- | --- | --- | --- | --- | --- | --- | --- | --- | --- | --- | --- | --- | --- | --- | --- | --- | --- | --- | --- | --- |
| **No.** | **Included Study** | | | **Assessor** | **Q1** | | **Q2** | | **Q3** | | **Q4** | **Q5** | | **Q6** | | **Q7** | | | **Q8** | | **Q9** | | **Overall Appraisal** | |
| 10 | | Ward et al., 2024 | 1 | | N | | N | | Y | | Y | Y | | Y | | | N | | N/A | | N | | Include | |
|  |  |  | 2 | | N | | N | | Y | | Y | N | | Y | | | N | | N/A | | N | | Exclude | |
|  |  |  | Final Results | | N | | N | | Y | | Y | N | | Y | | | N | | N/A | | N | | Exclude | |
| **Study Design II: Cohort Study (n=11)** | | | | | | | | | | | | | | | | | | | | | | | | |
| **No.** | | **Included Study** | **Assessor** | | **Q1** | **Q2** | | **Q3** | | **Q4** | **Q5** | | **Q6** | **Q7** | **Q8** | | | **Q9** | | **Q10** | | **Q11** | **Overall Appraisal** |  |
| 11 | | Bentley et al., 2015 | 1 | | N/A | N/A | | Y | | N | N | | Y | Y | Y | | | N | | N | | Y | Include |  |
|  |  |  | 2 | | N/A | N/A | | Y | | N | N | | Y | Y | Y | | | N | | N | | Y | Include |  |
|  |  |  | Final Results | | N/A | N/A | | Y | | N | N | | Y | Y | Y | | | N | | N | | Y | Include |  |
| 12 | | Bidner et al., 2022 | 1 | | N/A | N/A | | Y | | Y | Y | | Y | Y | Y | | | N | | N | | Y | Include |  |
|  |  |  | 2 | | N/A | N/A | | Y | | Y | Y | | Y | Y | Y | | | N | | N | | Y | Include |  |
|  |  |  | Final Results | | N/A | N/A | | Y | | Y | Y | | Y | Y | Y | | | N | | N | | Y | Include |  |
| 13 | | Erlick et al., 2022 | 1 | | N/A | N/A | | Y | | Y | N | | Y | Y | Y | | | N | | N | | Y | Include |  |
|  |  |  | 2 | | N/A | N/A | | Y | | Y | N | | Y | Y | Y | | | Y | | Y | | Y | Include |  |
|  |  |  | Final Results | | N/A | N/A | | Y | | Y | N | | Y | Y | Y | | | Y | | Y | | Y | Include |  |
| 14 | | Greenwold et al., 2013 | 1 | | Y | Y | | Y | | Y | N | | Y | Y | Y | | | Y | | N | | Y | Include |  |
|  |  |  | 2 | | Y | Y | | Y | | Y | N | | Y | Y | Y | | | Y | | N | | Y | Include |  |
|  |  |  | Final Results | | Y | Y | | Y | | Y | N | | Y | Y | Y | | | Y | | N | | Y | Include |  |
| 15 | | Henwood et al., 2017 | 1 | | N/A | N/A | | Y | | Y | Y | | Y | Y | Y | | | Y | | N | | Y | Include |  |
|  |  |  | 2 | | N/A | N/A | | Y | | Y | N | | Y | Y | Y | | | Y | | N | | Y | Include |  |
|  |  |  | Final Results | | N/A | N/A | | Y | | Y | N | | Y | Y | Y | | | Y | | N | | Y | Include |  |
| 16 | | Kotagal et al., 2015 | 1 | | N/A | N/A | | Y | | Y | N | | Y | Y | Y | | | Y | | N | | Y | Include |  |
|  |  |  | 2 | | N/A | N/A | | Y | | Y | N | | Y | Y | Y | | | Y | | N | | Y | Include |  |
|  |  |  | Final Results | | N/A | N/A | | Y | | Y | N | | Y | Y | Y | | | Y | | N | | Y | Include |  |
| 17 | | Miles et al., 2023 | 1 | | Y | Y | | Y | | Y | N | | Y | Y | Y | | | Y | | N | | Y | Include |  |
|  |  |  | 2 | | Y | Y | | Y | | Y | N | | Y | Y | Y | | | Y | | N | | Y | Include |  |
|  |  |  | Final Results | | Y | Y | | Y | | Y | N | | Y | Y | Y | | | Y | | N | | Y | Include |  |
| 18 | | Rominger et al., 2018 | 1 | | Y | N/A | | Y | | Y | N | | Y | Y | Y | | | Y | | N | | Y | Include |  |

**Supplementary Table 3: Details of Quality Evaluation Process *(Continued)***

| **Study Design II: Cohort Study (n=11)** | | | | | | | | | | | | | | | | | | | | | | | | | | | | | | |
| --- | --- | --- | --- | --- | --- | --- | --- | --- | --- | --- | --- | --- | --- | --- | --- | --- | --- | --- | --- | --- | --- | --- | --- | --- | --- | --- | --- | --- | --- | --- |
| **No.** | **Included Study** | | **Assessor** | **Q1** | | **Q2** | | | **Q3** | | | **Q4** | **Q5** | | **Q6** | | **Q7** | | | **Q8** | | | **Q9** | | **Q10** | | | **Q11** | **Overall Appraisal** |  |
| 18 | Rominger et al., 2018 | | 2 | N/A | | N/A | | | Y | | | Y | N | | Y | | Y | | | Y | | | Y | | N | | | Y | Include |  |
|  |  |  | Final Results | Y | | N/A | | | Y | | | Y | N | | Y | | Y | | | Y | | | Y | | N | | | Y | Include |  |
| 19 | Shah et al., 2020 | | 1 | Y | | Y | | | Y | | | Y | Y | | Y | | Y | | | Y | | | Y | | N | | | Y | Include |  |
|  |  |  | 2 | Y | | Y | | | Y | | | Y | Y | | Y | | Y | | | Y | | | Y | | N | | | Y | Include |  |
|  |  |  | Final Results | Y | | Y | | | Y | | | Y | Y | | Y | | Y | | | Y | | | Y | | N | | | Y | Include |  |
| 20 | Varner et al., 2022 | | 1 | N/A | | N/A | | | Y | | | Y | Y | | Y | | Y | | | Y | | | Y | | N | | | Y | Include |  |
|  |  |  | 2 | N/A | | N/A | | | Y | | | Y | Y | | Y | | Y | | | Y | | | N | | N | | | Y | Include |  |
|  |  |  | Final Results | N/A | | N/A | | | Y | | | Y | Y | | Y | | Y | | | Y | | | Y | | N | | | Y | Include |  |
| 21 | Westerway et al., 2019 | 1 | | Y | N/A | | | Y | | | N | | Y | Y | | | Y | | Y | | | N | | N | | | Y | | Include |  |
|  |  | 2 | | Y | N/A | | | Y | | | Y | | Y | Y | | | Y | | Y | | | N | | N | | | Y | | Include |  |
|  |  | Final Results | | Y | N/A | | | Y | | | N | | Y | Y | | | Y | | Y | | | N | | N | | | Y | | Include |  |
| **Study Design III: Cross-Sectional Study (n=6)** | | | | | | | | | | | | | | | | | | | | | | | | | | | | | |  |
| **No.** | **Included Study** | **Assessor** | | **Q1** | | | **Q2** | | | **Q3** | | | **Q4** | | | **Q5** | | **Q6** | | | **Q7** | | | | | **Q8** | | | **Overall Appraisal** |  |
| 22 | Lee JB et al., 2017 | 1 | | Y | | | Y | | | Y | | | Y | | | N | | N | | | Y | | | | | Y | | | Include |  |
|  |  | 2 | | Y | | | Y | | | Y | | | Y | | | Y | | N | | | Y | | | | | Y | | | Include |  |
|  |  | Final Results | | Y | | | Y | | | Y | | | Y | | | Y | | N | | | Y | | | | | Y | | | Include |  |
| 23 | Nathan et al., 2016 | 1 | | Y | | | Y | | | Y | | | Y | | | Y | | N | | | Y | | | | | Y | | | Include |  |
|  |  | 2 | | Y | | | Y | | | Y | | | Y | | | Y | | N | | | Y | | | | | Y | | | Include |  |
|  |  | Final Results | | Y | | | Y | | | Y | | | Y | | | Y | | N | | | Y | | | | | Y | | | Include |  |
| 24 | Shah SP et al., 2009 | 1 | | Y | | | Y | | | Y | | | Y | | | N | | N | | | Y | | | | | Y | | | Include |  |
|  |  | 2 | | Y | | | Y | | | Y | | | Y | | | N | | N | | | Y | | | | | Y | | | Include |  |
|  |  | Final Results | | Y | | | Y | | | Y | | | Y | | | N | | N | | | Y | | | | | Y | | | Include |  |
| 25 | Shokoohi et al., 2019 | 1 | | Y | | | Y | | | Y | | | Y | | | N | | N | | | Y | | | | | Y | | | Include |  |
|  |  | 2 | | Y | | | Y | | | Y | | | Y | | | Y | | N | | | Y | | | | | Y | | | Include |  |
|  |  | Final Results | | Y | | | Y | | | Y | | | Y | | | Y | | N | | | Y | | | | | Y | | | Include |  |
| 26 | Vinayak et al., 2017 | 1 | | Y | | | Y | | | Y | | | Y | | | N | | Y | | | Y | | | | | Y | | | Include | |
|  |  | 2 | | Y | | | Y | | | Y | | | Y | | | Y | | N | | | Y | | | | | Y | | | Include | |

**Supplementary Table 3: Details of Quality Evaluation Process *(Continued)***

| **Study Design III: Cross-Sectional Study (n=6)** | | | | | | | | | | | | | | | | |  |
| --- | --- | --- | --- | --- | --- | --- | --- | --- | --- | --- | --- | --- | --- | --- | --- | --- | --- |
| **No.** | **Included Study** | **Assessor** | | | **Q1** | **Q2** | | **Q3** | | **Q4** | | **Q5** | **Q6** | **Q7** | **Q8** | **Overall Appraisal** |  |
| 26 | Vinayak et al., 2017 | Final Results | | | Y | Y | | Y | | Y | | N | Y | Y | Y | Include | |
| 27 | Wanjiku et al., 2018 | | 1 | Y | | | Y | | Y | | Y | N | Y | Y | Y | Include | |
|  |  |  | 2 | Y | | | Y | | Y | | Y | Y | Y | Y | Y | Include | |
|  |  |  | Final Results | Y | | | Y | | Y | | Y | N | Y | Y | Y | Include | |

**Notes:**

①Answers to questions: Yes (Y), No (N), Not applicable (N/A), or Unclear.

②Answers to overall appraisal: Include, Exclude, or Seek further information.
