# Supplementary material for: Effectiveness of obstetric point-of-care ultrasound (POCUS) training: a systematic review and meta-analysis based on the ADDIE training model
Source: Ultrasound J. 2025 Dec 31;17:67. doi: 10.1186/s13089-025-00471-z (PMC12756214; doi:10.1186/s13089-025-00471-z)
Supplement: Supplementary file 2 — Additional file 2. [file 13089_2025_471_MOESM2_ESM.docx]

Appendix II - Supplementary Files

**Catalog of Supplementary** **Files**

| **No.** | **Document** | **Page** |
| --- | --- | --- |
| 1 | Supplementary File 1: Preliminary Data Extraction Form | Pages 1-2 |
| 2 | Supplementary File 2: Standardized Data Extraction Form After Pilot Testing | Pages 3-4 |
| 3 | Supplementary File 3: List of Included Studies (N=27) | Pages 5-6 |
| 4 | Supplementary File 4: Studies Ineligible Following Full-Text Review (N=41) | Pages 7-9 |
| 5 | Supplementary File 5: List of Supplementary Search (N=7) | Page 10 |
| 6 | Supplementary File 6: Data Extraction Results and Details from Included Studies (N=27) | Pages 11-176 |

**Supplementary File 1: Preliminary Data Extraction Form**

**JBI Qualitative Data Extraction Tool**

**Reviewer: Date:**

**Author: Year:**

**Journal: Record Number:**

| **1. Study Characteristics** | |
| --- | --- |
| **Item** | **Extracted Data** |
| 1.1 Author (Publication Year) |  |
| 1.2 Country |  |
| 1.3 Location |  |
| 1.4 Study Design |  |
| 1.5 Sample Size |  |
|  | |
| **2. Population Characteristics (Healthcare Providers Receiving POCUS Training)** | |
| **Item** | **Extracted Data** |
| 2.1 Age |  |
| 2.2 Gender |  |
| 2.3 Professional Role |  |
| 2.4 Years of Experience |  |
| 2.5 Prior Ultrasound Training Experience |  |
| 2.6 Related Certifications |  |
|  | |
| **3. Intervention Characteristics (According to ADDIE Training Model)** | |
| **Item** | **Extracted Data** |
| 3.1 Analysis |  |
| 3.2 Design |  |
| 3.3 Development |  |
| 3.4 Implementation |  |
| 3.5 Evaluation |  |
|  | |
| **4. Outcome Characteristics** | |
| **Item** | **Extracted Data** |
| **4.1 Training Outcomes** |  |
| 4.1.1 Diagnostic Accuracy |  |
| 4.1.2 Knowledge Acquisition |  |
| 4.1.3 Skills Acquisition |  |
| 4.1.4 Knowledge Retention |  |
| 4.1.5 Practical Application |  |
| **4.2 Maternal and Neonatal Health Outcomes** |  |
| 4.2.1 Maternal Health Outcomes |  |
| 4.2.2 Neonatal Health Outcomes |  |
| 4.2.3 Maternal Satisfaction |  |
| **4.3 Health Economic Indicators** |  |
| 4.3.1 Cost-Effectiveness Analysis |  |
| 4.3.2 Cost-Utility Analysis |  |
| 4.3.3 Cost-Benefit Analysis |  |

**Supplementary File 2****:**

**Standardized Data Extraction Form After Pilot Testing**

**JBI Qualitative Data Extraction Tool (Final)**

**Reviewer: Date:**

**Author: Year:**

**Journal: Record Number:**

| **1. Study Characteristics** | |
| --- | --- |
| **Item** | **Extracted Data** |
| 1.1 Author (Publication Year) |  |
| 1.2 Country |  |
| 1.3 Location |  |
| 1.4 Study Design |  |
| 1.5 Sample Size |  |
|  | |
| **2. Population Characteristics (Healthcare Providers Receiving POCUS Training)** | |
| **Item** | **Extracted Data** |
| 2.1 Age |  |
| 2.2 Gender |  |
| 2.3 Professional Role |  |
| 2.4 Work Experience (Duration) |  |
| 2.5 Prior Ultrasound Training Experience |  |
|  | |
| **3. Intervention Characteristics (According to ADDIE Training Model)** | |
| **Item** | **Extracted Data** |
| 3.1 Analysis |  |
| 3.2 Design |  |
| 3.3 Development |  |
| 3.4 Implementation |  |
| 3.5 Evaluation |  |
|  | |
| **4. Outcome Characteristics** | |
| **Item** | **Extracted Data** |
| **4.1 Training Outcomes** |  |
| 4.1.1 Diagnostic Accuracy |  |
| 4.1.2 Knowledge Acquisition |  |
| 4.1.3 Skills Acquisition |  |
| 4.1.4 Knowledge Retention |  |
| 4.1.5 Practical Application |  |
| **4.2 Maternal and Neonatal Health Outcomes** (Including Physiological and Psychological Outcomes) |  |
| **4.3 Health Economic Outcomes** |  |

**Supplementary File 3: List of Included Studies (N=27)**

1. Cook J, Rao V, Bell F, et al. Simulation‐based clinical learning for the third year medical student: effectiveness of transabdominal and transvaginal ultrasound for elucidation of OB/GYN scenarios [J]. Journal of Clinical Ultrasound, 2020, 48(8): 457-461.
2. Dávila-Román V G, Toenjes A K, Meyers R M, et al. Ultrasound core laboratory for the household air pollution intervention network trial: standardized training and image management for field studies using portable ultrasound in fetal, lung, and vascular evaluations [J]. Ultrasound in Medicine & Biology, 2021, 47(6): 1506-1513.
3. Filler L, Lettang K. High-fidelity simulation with transvaginal ultrasound in the emergency department [J]. Journal of Education & Teaching in Emergency Medicine, 2024, 9(3): S65.
4. Hall E A, Matilsky D, Zang R, et al. Analysis of an obstetrics point-of-care ultrasound training program for healthcare practitioners in Zanzibar, Tanzania [J]. The Ultrasound Journal, 2021, 13: 1-12.
5. Kimberly H H, Murray A, Mennicke M, et al. Focused maternal ultrasound by midwives in rural Zambia [J]. Ultrasound in Medicine & Biology, 2010, 36(8): 1267-1272.
6. Kolbe N, Killu K, Coba V, et al. Point of care ultrasound (pocus) telemedicine project in rural Nicaragua and its impact on patient management [J]. Journal of Ultrasound, 2015, 18: 179-185.
7. Lee E, Alsofrom J, Streeter M, et al. Obstetric and gynecologic ultrasound training at the Uganda nursing school Bwindi: initial experiences and challenges [C]. 2015 IEEE Global Humanitarian Technology Conference (GHTC). IEEE, 2015: 425-430.
8. Vinayak S, Brownie S. Collaborative task-sharing to enhance the point-of-care ultrasound (pocus) access among expectant women in Kenya: the role of midwife sonographers [J]. Journal of Interprofessional Care, 2018, 32(5): 641-644.
9. Wachira J, Matheka D M, Masheti S A, et al. A training program for obstetrics point-of-care ultrasound to 514 rural healthcare providers in Kenya [J]. BMC Medical Education, 2023, 23(1): 922.
10. Ward R E, Joe W B, Jha S, et al. Point-of-care ultrasound for high-risk pregnancy screening in rural Nepal [J]. Journal of the American College of Radiology, 2024, 21(8): 1235-1238.
11. Bentley S, Hexom B, Nelson B P. Evaluation of an obstetric ultrasound curriculum for midwives in Liberia [J]. Journal of Ultrasound in Medicine, 2015, 34(9): 1563-1568.
12. Bidner A, Bezak E, Parange N. Evaluation of antenatal point-of-care ultrasound training workshops for rural/remote healthcare clinicians: a prospective single cohort study [J]. BMC Medical Education, 2022, 22(1): 906.
13. Erlick M, Marini T, Drennan K, et al. Assessment of a brief standardized obstetric ultrasound training program for individuals without prior ultrasound experience [J]. Ultrasound Quarterly, 2023, 39(3): 124-128.
14. Greenwold N, Wallace S, Prost A, et al. Implementing an obstetric ultrasound training program in rural Africa [J]. International Journal of Gynecology & Obstetrics, 2014, 124(3): 274-277.
15. Henwood P C, Mackenzie D C, Liteplo A S, et al. Point‐of‐care ultrasound use, accuracy, and impact on clinical decision making in Rwanda hospitals [J]. Journal of Ultrasound in Medicine, 2017, 36(6): 1189-1194.
16. Kotagal M, Quiroga E, Ruffatto B J, et al. Impact of point-of-care ultrasound training on surgical residents’ confidence [J]. Journal of Surgical Education, 2015, 72(4): e82-e87.
17. Miles G, Newcomb P, Spear D. Feasibility of teaching emergency department nurses to determine fetal heart rates using bedside ultrasound versus the hand-held doppler [J]. Journal of Radiology Nursing, 2023, 42(4): 431-439.
18. Rominger A H, Gomez G A A, Elliott P. The implementation of a longitudinal pocus curriculum for physicians working at rural outpatient clinics in Chiapas, Mexico [J]. Critical Ultrasound Journal, 2018, 10: 1-8.
19. Shah S, Santos N, Kisa R, et al. Efficacy of an ultrasound training program for nurse midwives to assess high-risk conditions at labor triage in rural Uganda [J]. PLoS One, 2020, 15(6): e0235269.
20. Varner C E, Lee S, McLeod S, et al. Point-of-care ultrasound for evaluation of vaginal bleeding or abdominal pain in early pregnancy: use by family physicians following focused training and certification [J]. Canadian Family Physician, 2022, 68(11): e326-e332.
21. Westerway S C. Comparing the effectiveness of training course formats for point‐of‐care ultrasound in the third trimester of pregnancy [J]. Australasian Journal of Ultrasound in Medicine, 2019, 22(1): 45-50.
22. Lee J B, Tse C, Keown T, et al. Evaluation of a point of care ultrasound curriculum for Indonesian physicians taught by first-year medical students [J]. World Journal of Emergency Medicine, 2017, 8(4): 281.
23. Nathan R O, Swanson J O, Swanson D L, et al. Evaluation of focused obstetric ultrasound examinations by health care personnel in the Democratic Republic of Congo, Guatemala, Kenya, Pakistan, and Zambia [J]. Current Problems in Diagnostic Radiology, 2017, 46(3): 210-215.
24. Shah S P, Epino H, Bukhman G, et al. Impact of the introduction of ultrasound services in a limited resource setting: rural Rwanda 2008 [J]. BMC International Health and Human Rights, 2009, 9: 1-6.
25. Shokoohi H, Raymond A, Fleming K, et al. Assessment of point-of-care ultrasound training for clinical educators in Malawi, Tanzania and Uganda [J]. Ultrasound in Medicine & Biology, 2019, 45(6): 1351-1357.
26. Vinayak S. Training midwives to perform basic obstetric pocus in rural areas using a tablet platform and mobile phone transmission technology [J]. Ultrasound in Medicine and Biology, 2017, 43(Suppl 1): S84.
27. Wanjiku G W, Bell G, Wachira B. Assessing a novel point-of-care ultrasound training program for rural healthcare providers in Kenya [J]. BMC Health Services Research, 2018, 18: 1-7.

**Supplementary File 4****:**

**Studies Ineligible Following Full-Text Review (N=41)**

**Reason 1 for Exclusion: Conference abstracts or no available full-text (n=21)**

1. Tolsgaard M G, Dreisler E, Nørgaard L, et al. Long-term effect of simulation-based training on clinical performance: a randomised trial [J]. Ultrasound in Obstetrics and Gynecology, 2014, 44: 33-33.
2. Sanchez O, Baker J, Vaughan J, et al. The rural obstetrical ultrasound triage exam (route): teaching health care workers in international settings [J]. Obstetrics & Gynecology, 2015, 125: 122S.
3. Kolbe N, Killu K, Coba V, et al. Implementing point of care ultrasound (pocu) in rural Nicaragua changes patient management [J]. Critical Care Medicine, 2013, 41(12): A31.
4. Dyre L, Nørgaard L N, Rosthøj S, et al. The effects of simulation-based transvaginal ultrasound training on quality and efficiency of care [J]. Annals of Surgery, 2017, 265: 630-637.
5. Mbuyita S, Tillya R, Godfrey R, et al. Measuring correctness in assessing maternal complications using a handheld ultrasound device by trained mid-level providers at dispensary and health centre levels in rural Tanzania: a longitudinal study [J]. International Journal of Gynecology & Obstetrics, 2015, 131: E72-E313.
6. Walker T S, Millman N, Taylor M. Integration of a portable ultrasound device into community midwife practice: a feasibility study [J]. Archives of Disease in Childhood-Fetal and Neonatal Edition, 2010, 95(Suppl 1): Fa103-Fa103.
7. Lee E, Rubio X B, Chang Y, et al. Integrating obstetric point-of-care-ultrasonography (pocus) simulation curriculum [J]. Obstetrics & Gynecology, 2023, 141(5S): 95S.
8. Henwood P C, Rempell J S, Liteplo A S, et al. Point-of-care ultrasound use over six-month training period in Rwandan district hospitals [J]. African Journal of Emergency Medicine, 2013, 3(4): S5-S6.
9. Buchanan I M, Sandhanwalia S K, Chan T M, et al. Procedural learning dynamics of a point-of-care ultrasound education experience [C]. Plenary Oral Presentations. CJEM, 2015, 17(S2): S4-S88.
10. Sharma R, Guedry M, Davison J, et al. Emergency physicians evaluation of second and third trimester pregnancy using point of care ultrasound: a pilot study [J]. Western Journal of Emergency Medicine: Integrating Emergency Care with Population Health, 2024, 25(3.1).
11. Shah S, Adedipe A, Ruffatto B, et al. Effect of educational intervention on ed physician ability to perform a rapid, bedside ultrasound assessment in late pregnancy: 645 [J]. Academic Emergency Medicine, 2013, 20: S258.
12. Parange N, Bezak E, Bidner A. OP04. 02: Employing kirpatrick evaluation framework (kef) for evaluating antenatal point of care ultrasound (pocus) training to health professionals in remote South Australia in low‐resource settings [J]. Ultrasound in Obstetrics & Gynecology, 2018, 52.
13. Tkacik A, Reuter Q. First trimester ultrasound: utilizing advanced practice providers to reduce emergency department length of stay [J]. Academic Emergency Medicine, 2021, 28(S1):S9-S398.
14. Kimberly H, Murray A, Mennicke M, et al. Teaching focused obstetric ultrasound to midwives in rural Zambia [J]. Annals of Emergency Medicine, 2009, 54(3): S87.
15. Varner C, Bearss E, Hu S, et al. The family medicine obstetrical ultrasound (famous) course: a model for training office-based family physicians in first trimester point of care ultrasound [J]. Canadian Journal of Emergency Medicine, 2016, 18(S1): S124-S124.
16. Das D, Lema P C, Gupta S, et al. Assessment of point-of-care ultrasound in Tanzania: 582 [J]. Academic Emergency Medicine, 2012, 19: S306-S307.
17. Packer N, McLarty R, Byrne C, et al. Are interactive workshops effective at introducing medical students to pocus? [C]. CAEP/ACMU Scientific Abstracts. CJEM, 2014, 16(S1): S19-S114.
18. Bidner A, Parange N, Bezak E. Antenatal point-of-care ultrasound (pocus) in rural and remote Australian communities: an evaluation of training [J]. Ultrasound in Medicine and Biology, 2019, 45: S81-S82.
19. Wong A, Hall E A, Matilsky D, et al. Analysis of an obstetrics bedside ultrasound training program for health care practitioners in Zanzibar, Tanzania [J]. Academic Emergency Medicine, 26: S9-S304.
20. Cassella C R, Panebianco N, Lockwood A, et al. 348 Training and competency of emergency department advanced practice providers in first trimester pregnancy ultrasound [J]. Annals of Emergency Medicine, 2019, 74(4): S137.
21. Shindruk A, Schick M, McBeth C, et al. 272 An approach to point-of-care ultrasound training in a teaching hospital in the Gambia [J]. Annals of Emergency Medicine, 2022, 80(4): S118-S119.

**Reason 2 for Exclusion: Ineligible outcome (n=12)**

1. Mans P A, Yogeswaran P, Adeniyi O V. Building consensus on the point-of-care ultrasound skills required for effective healthcare service delivery at district hospitals in South Africa: a delphi study [J]. International Journal of Environmental Research and Public Health, 2023, 20(23): 7126.
2. Johnston B K, Darling E K, Malott A, et al. Canadian midwives’ perspectives on the clinical impacts of point of care ultrasound in obstetrical care: a concurrent mixed-methods study [J]. Heliyon, 2024, 10(6).
3. Davis N R, Alade K H. Twelve tips for point-of-care ultrasound teaching in low-resource settings [J]. Medical Teacher, 2021, 43(10): 1134-1138.
4. Jalil R, Warren R, Ma I W Y. Point of care ultrasound training needs for primary care physicians: practice setting matters [J]. Cogent Education, 2019, 6(1): 1617826.
5. Woo M Y, Frank J R, Lee A C. Point-of-care ultrasonography adoption in Canada: using diffusion theory and the evaluation tool for ultrasound skills development and education (etude) [J]. Canadian Journal of Emergency Medicine, 2014, 16(5): 345-351.
6. Nguyen J, Amirnovin R, Ramanathan R, et al. The state of point-of-care ultrasonography use and training in neonatal–perinatal medicine and pediatric critical care medicine fellowship programs [J]. Journal of Perinatology, 2016, 36(11): 972-976.
7. Wanjiku G W, Bell G, Kapadia S, et al. Impact of point-of-care ultrasound use on patient referral decisions in rural Kenya: a mixed methods study [J]. BMC Health Services Research, 2024, 24(1): 212.
8. Kumar M B, Mulongo C M, Pincerato L, et al. Nurse-led point-of-care ultrasonography with telemedicine review to improve the impact of antenatal care: a formative qualitative study in Kenya [J]. medRxiv: the Preprint Server for Health Sciences, 2023: 2023.10. 12.23296931.
9. Fasina A A, Dean A J, Panebianco N L, et al. Evaluation of diagnostic imaging capacity and the role for point-of-care ultrasound (pocus) within the Zanzibar health system [J]. POCUS Journal, 2021, 6(1): 45.
10. Ramirez J B, Chen X X, Ludwig N, et al. Point-of-care ultrasonography (pocus) in obstetric anesthesia fellowship training: survey of North American programs [J]. Brazilian Journal of Anesthesiology, 2024, 74(6): 844547.
11. Ienghong K, Cheung L W, Tiamkao S, et al. Integrating point of care ultrasound education into clinical practice at the emergency department [J]. Tomography, 2022, 8(2): 1052-1059.
12. Dornhofer K, Farhat A, Guan K, et al. Evaluation of a point‐of‐care ultrasound curriculum taught by medical students for physicians, nurses, and midwives in rural Indonesia [J]. Journal of Clinical Ultrasound, 2020, 48(3): 145-151.

**Reason 3 for Exclusion: Ineligible intervention (n=6)**

1. Wanjiku G, Dreizler L, Wu S, et al. Utility of hand-held ultrasound for image acquisition and interpretation by trained Kenyan providers [J]. The Ultrasound Journal, 2023, 15(1): 12.
2. Gomes D J, Kaufman B, Aluisio A R, et al. Assessment of acute obstetrical needs and the potential utility of point-of-care ultrasound in the north east region of Haiti: a cross-sectional study [J]. Annals of Global Health, 2020, 86(1): 72.
3. Stather D R, Maceachern P, Rimmer K, et al. Assessment and learning curve evaluation of endobronchial ultrasound skills following simulation and clinical training [J]. Respirology. 2011;16(4):698-704.
4. Pedersen J K, Sira C, Trovik J. Handheld transabdominal ultrasound, after limited training, may confirm first trimester viable intrauterine pregnancy: a prospective cohort study [J]. Scandinavian Journal of Primary Health Care, 2021, 39(2): 123-130.
5. Toscano M, Marini T, Lennon C, et al. Diagnosis of pregnancy complications using blind ultrasound sweeps performed by individuals without prior formal ultrasound training [J]. Obstetrics & Gynecology, 2023, 141(5): 937-948.
6. 杨秀华, 孟涛. 床旁胎儿超声可视化教学在产科住培教学查房中的应用 [J]. 卫生职业教育, 2019, 37: 151-152.

**Reason 4 for Exclusion: Ineligible study design (n=1)**

1. Mubuuke A G, Erem G, Nassanga R, et al. Point of care obstetric ultrasound training for midwives and nurses: implementation and experiences of trainees at a rural based hospital in Sub-saharan Africa: a qualitative study [J]. BMC Research Notes, 2023, 16(1): 287.

**Reason 5 for Exclusion: Inappropriate language type (n=1)**

1. Rolin J, Corini E, Michel J, et al. Repérage échographique du fœtus: évaluation prospective de l’impact d’un dispositif de formation continue des sages-femmes sur l’organisation des soins en salle de naissance [J]. Pédagogie Médicale, 2022, 23(2): 115-123.

**Supplementary File 5: List of Supplementary Search (N=7)**

1. Lee J B, Tse C, Keown T, et al. Evaluation of a point of care ultrasound curriculum for Indonesian physicians taught by first-year medical students [J]. World Journal of Emergency Medicine, 2017, 8(4): 281.
2. Bidner A, Bezak E, Parange N. Evaluation of antenatal point-of-care ultrasound training workshops for rural/remote healthcare clinicians: a prospective single cohort study [J]. BMC Medical Education, 2022, 22(1): 906.
3. Erlick M, Marini T, Drennan K, et al. Assessment of a brief standardized obstetric ultrasound training program for individuals without prior ultrasound experience [J]. Ultrasound Quarterly, 2023, 39(3): 124-128.
4. Greenwold N, Wallace S, Prost A, et al. Implementing an obstetric ultrasound training program in rural Africa [J]. International Journal of Gynecology & Obstetrics, 2014, 124(3): 274-277.
5. Henwood P C, Mackenzie D C, Liteplo A S, et al. Point‐of‐care ultrasound use, accuracy, and impact on clinical decision making in Rwanda hospitals [J]. Journal of Ultrasound in Medicine, 2017, 36(6): 1189-1194.
6. Nathan R O, Swanson J O, Swanson D L, et al. Evaluation of focused obstetric ultrasound examinations by health care personnel in the Democratic Republic of Congo, Guatemala, Kenya, Pakistan, and Zambia [J]. Current Problems in Diagnostic Radiology, 2017, 46(3): 210-215.
7. Shah S P, Epino H, Bukhman G, et al. Impact of the introduction of ultrasound services in a limited resource setting: rural Rwanda [J]. BMC International Health and Human Rights, 2009, 9: 1-6.

**Supplementary File 6:**

**Data Extraction Results and Details from Included Studies (N=27)**

**JBI Qualitative Data Extraction Tool**

**Reviewer: XXX & XXX & XXX Date: 27^th^ February 2025**

**Author: Cook et al. Year: 2020**

**Journal: Journal of Clinical Ultrasound Record Number: 1**

| **1. Study Characteristics** | |
| --- | --- |
| **Item** | **Extracted Data** |
| 1.1 Author (Publication Year) | Cook et al., 2020 |
| 1.2 Country | United States of America |
| 1.3 Location | University of South Carolina School of Medicine Columbia, Columbia, South Carolina, USA |
| 1.4 Study Design | Quasi-experimental study |
| 1.5 Sample Size | 76 (68 included, 8 dropped out) |
|  | |
| **2. Population Characteristics (Healthcare Providers Receiving POCUS Training)** | |
| **Item** | **Extracted Data** |
| 2.1 Age | Mean: 27 |
| 2.2 Gender | (1) Male: 57.9% (44/76);  (2) Female: 42.1% (32/76). |
| 2.3 Professional Role | Students: 100% (76/76) |
| 2.4 Work Experience (Duration) | NI |
| 2.5 Prior Ultrasound Training Experience | None (0/76) |
|  | |
| **3. Intervention Characteristics (According to ADDIE Training Model)** | |
| **Item** | **Extracted Data** |
| 3.1 Analysis | 1. Trainee Group:    1. Third-year medical students (M3) in the Obstetrics and Gynecology (Ob/Gyn) clerkship at the University of South Carolina School of Medicine.    2. Participation was voluntary and would not impact clerkship grades. 2. Needs Analysis:    1. Point-of-care ultrasound (POCUS) is gaining recognition as a teaching tool for medical students transitioning to clinical rotations.    2. The study aimed to determine if ultrasound simulation improves understanding of Ob/Gyn anatomy and pathology. 3. Baseline Survey (Pre-training):    1. Initial multiple-choice question (MCQ) test assessing knowledge of pelvic ultrasound anatomy and pathology. 4. Impact on Instructional Design:    1. The results of the baseline test influenced the structure and focus of the training. |
| 3.2 Design | 1. Training Objectives:    1. Improve students' knowledge of pelvic ultrasound anatomy and pathology.    2. Enhance confidence in performing ultrasound examinations. 2. Training Methods (Course Format):    1. One-hour OB/Gyn ultrasound simulation session, including hands-on practice. 3. Training Plan/Syllabus:    1. Content included scanning normal pelvic anatomy and identifying pathological findings. |
| 3.3 Development | 1. Instructors/Teachers:    1. Faculty members conducted training sessions using standardized cases. 2. Teaching Aids:    1. 3D ultrasound simulator with transabdominal and transvaginal probes. 3. Teaching Materials:    1. Lesson plans included ultrasound interpretation and hands-on scanning practice. |
| 3.4 Implementation | 1. Time:    1. One-hour training session per group of students. 2. Location/Setting:    1. Conducted in a simulation lab at the medical school. 3. Participants:    1. Each session had an average of eight students. 4. Execution Process:    1. Students took turns independently scanning and identifying pathologies. |
| 3.5 Evaluation | 1. Formative Evaluation:    1. Students received immediate feedback during the training session. 2. Summative Evaluation:    1. Post-training multiple-choice test to measure knowledge improvement. 3. Follow-up:    1. Students were surveyed on their comfort level and training experience. |
|  | |
| **4. Outcome Characteristics** | |
| **Item** | **Extracted Data** |
| **4.1 Training Outcomes** |  |
| 4.1.1 Diagnostic Accuracy | NI |
| 4.1.2 Knowledge Acquisition | 1. Pre-Test:    1. Average Score: 59.6%.    2. Median (IQR): 61.1% (3%).    3. Range: 22.2% to 94.4%. 2. Post-Test:    1. Average Score: 76.2%.    2. Range: 44.4% to 100.0%. 3. Knowledge Improvement:    1. Students answered an average of +3.0 additional questions correctly (out of 18), corresponding to a 16.6% relative improvement.    2. Statistical Significance: Paired t-test showed *P* < 0.001.    3. Standard Deviation (SD): 2.42. |
| 4.1.3 Skills Acquisition | 1. Comfort Level Improvement:    1. 48/68 (70.6%) students reported increased comfort.    2. 41/68 (60.3%) improved by one level.    3. 5/68 (7.4%) improved by two levels.    4. 2/68 (2.9%) improved by three levels (capable of performing and teaching). 2. Statistical Analysis:   Gamma statistic: 0.726 (95% *CI*: 0.479-0.973), *P* < 0.001. |
| 4.1.4 Knowledge Retention | NI |
| 4.1.5 Practical Application | NI |
| **4.2 Maternal and Neonatal Health Outcomes** (Including Physiological and Psychological Outcomes) | NI |
| **4.3 Health Economic Outcomes** | NI |

**JBI Qualitative Data Extraction Tool**

**Reviewer: XXX & XXX & XXX Date: 27^th^ February 2025**

**Author: Dávila-Román et al. Year: 2021**

**Journal: Ultrasound in Medicine & Biology Record Number: 2**

| **1. Study Characteristics** | |
| --- | --- |
| **Item** | **Extracted Data** |
| 1.1 Author (Publication Year) | Dávila-Román et al., 2021 |
| 1.2 Country | Guatemala, Peru, India, Rwanda |
| 1.3 Location | Core Laboratory: Washington University in St. Louis, Missouri, USA  Field Sites:   1. Guatemala (Guatemala City) 2. Peru 3. India (Chennai, Tamil Nadu) 4. Rwanda |
| 1.4 Study Design | Quasi-experimental study |
| 1.5 Sample Size | 18 |
|  | |
| **2. Population Characteristics (Healthcare Providers Receiving POCUS Training)** | |
| **Item** | **Extracted Data** |
| 2.1 Age | NI |
| 2.2 Gender | NI |
| 2.3 Professional Role | Sonographers: 100% (18/18) |
| 2.4 Work Experience (Duration) | NI |
| 2.5 Prior Ultrasound Training Experience | 1. All participants had prior experience in ultrasound or healthcare. 2. Completed introductory online training before the program. |
|  | |
| **3. Intervention Characteristics (According to ADDIE Training Model)** | |
| **Item** | **Extracted Data** |
| 3.1 Analysis | Trainee Group:Target group: Sonographers recruited from research centers (IRCs) in Guatemala, Peru, India, and Rwanda.Selection criteria: Individuals with prior sonography and/or healthcare experience were chosen for training.  1. Needs Analysis:    1. Context:       1. Nearly three billion people worldwide rely on solid biomass fuels, causing indoor air pollution, a major environmental health risk.       2. This pollution is linked to cardiovascular and pulmonary diseases.    2. HAPIN Study:       1. Evaluates the impact of liquefied petroleum gas (LPG) stoves on lung and vascular health.       2. Requires portable ultrasound to measure biomarkers, aiming to reduce imaging variability and improve measurement precision. 2. Baseline Survey:    1. Purpose:       1. Assess trainees' initial knowledge of ultrasound principles, device operation, and image interpretation.       2. Establish baseline scores for comparison after training. |
| 3.2 Design | 1. Training Objectives:    1. Knowledge goals: Understand ultrasound principles and imaging biomarkers.    2. Skill goals: Operate portable ultrasound devices and acquire high-quality images.    3. Attitude goals: Develop a standardized approach to ensure data comparability. 2. Training Methods:    1. Blended learning approach:       1. Online modules: Introduced fundamental ultrasound concepts.    2. In-person training: Combination of lectures, hands-on practice, and assessment. |
| 3.3 Development | 1. Instructors/Teachers:    1. The faculty and instructors came from:       1. Washington University in St. Louis (cardiovascular imaging).       2. Johns Hopkins University (pulmonary ultrasound). 2. Teaching Aids:    1. Equipment:    2. Standardized use of Sonosite Edge portable ultrasound devices with three transducers.    3. Materials:       1. Lecture slides, PPTs, test questions, and simulated cases.       2. Interactive multimedia training (SonoSim software).       3. Step-by-step ultrasound imaging guides (“knobology” manuals). |
| 3.4 Implementation | 1. Time & Schedule:    1. 2-week on-site training at Washington University in St. Louis.    2. Daily structure: Morning lectures + Afternoon hands-on scanning. 2. Location/Setting:    1. Primary site: Washington University in St. Louis.    2. Fieldwork: Guatemala, Peru, India, Rwanda (home-based & clinic-based ultrasound). 3. Participants:    1. Trainers: Experts from Washington University & Johns Hopkins University.    2. Trainees: 18 sonographers (3-4 per country + 5 from the UCL). 4. Execution Process:    1. Phase 1: Online training (ultrasound principles, study protocols).    2. Phase 2: On-site training (lectures + hands-on practice).    3. Phase 3: Practical assessments.    4. Phase 4: Certification (submit 25 high-quality cases).    5. Phase 5: Continuous quality control & retraining if needed. 5. Adaptation Records:    1. Quality control tracking: Ensuring standardization across sites.    2. Retraining when necessary: Sonographers not meeting QC standards were identified and re-trained. |
| 3.5 Evaluation | 1. Formative Evaluation (Ongoing Assessment):    1. Purpose: Track progress, adjust training as needed.    2. Methods: Pre- and post-training tests, hands-on assessments.    3. Timing: Throughout training.    4. Results: Scores improved from 60% to 84%; additional training provided for those below standards. 2. Summative Evaluation:    1. Purpose: Determine if trainees achieved required competencies.    2. Methods: Final tests, completion of 25 certified ultrasound cases, trainee feedback.    3. Timing: End of training.    4. Results: 100% feedback response rate, average rating 4.9/5. 3. Follow-up:    1. Purpose: Ensure long-term standardization and performance consistency.    2. Methods: Cloud-based image tracking, expert review, periodic retraining if necessary.    3. Timing: Ongoing post-training.    4. Results: Remote quality control maintained; retraining conducted for non-compliant sonographers. |
|  | |
| **4. Outcome Characteristics** | |
| **Item** | **Extracted Data** |
| **4.1 Training Outcomes** |  |
| 4.1.1 Diagnostic Accuracy | NI |
| 4.1.2 Knowledge Acquisition | 1. **Pre-Training Scores**:    1. Fetal ultrasound: 71% ± 13%.    2. Vascular/lung ultrasound: 60% ± 8%. 2. **Post-Training Scores**:    1. Fetal ultrasound: 93% ± 7%.    2. Vascular/lung ultrasound: 84% ± 10%. 3. **Statistical Significance**:    1. Both showed significant improvement (p < 0.0001)*.* |
| 4.1.3 Skills Acquisition | 1. **Certification Pass Rate**:    1. 17/18 sonographers (94.4%) successfully passed certification.    2. 1/18 (5.6%) required additional training. 2. **Practical Certification**:    1. Each sonographer completed 25 full ultrasound studies for certification. 3. Feedback:    1. Qualitative written feedback indicated high confidence with newly acquired ultrasound skills. |
| 4.1.4 Knowledge Retention | NI |
| 4.1.5 Practical Application | 1. Certified sonographers performed ultrasound imaging across four countries. 2. **Ultrasound Modalities**:    1. Fetal ultrasound (biometry, anatomy, pregnancy dating).    2. Lung ultrasound (pneumonia evaluation).    3. Vascular ultrasound (CIMT, BART). |
| **4.2 Maternal and Neonatal Health Outcomes** (Including Physiological and Psychological Outcomes) | NI |
| **4.3 Health Economic Outcomes** | NI |

**JBI Qualitative Data Extraction Tool**

**Reviewer: XXX & XXX & XXX Date: 27^th^ February 2025**

**Author: Filler & Lettang Year: 2024**

**Journal: Journal of Education and Teaching in Emergency Medicine**

**Record Number: 3**

| **1. Study Characteristics** | |
| --- | --- |
| **Item** | **Extracted Data** |
| 1.1 Author (Publication Year) | Filler & Lettang, 2024 |
| 1.2 Country | United States of America |
| 1.3 Location | Creighton University School of Medicine Phoenix Program, Valleywise Health Medical Center, Department of Emergency Medicine, Phoenix, Arizona, USA |
| 1.4 Study Design | Quasi-experimental study |
| 1.5 Sample Size | 32 |
|  | |
| **2. Population Characteristics (Healthcare Providers Receiving POCUS Training)** | |
| **Item** | **Extracted Data** |
| 2.1 Age | NI |
| 2.2 Gender | NI |
| 2.3 Professional Role | Doctors: 100% (32/32) |
| 2.4 Work Experience (Duration) | NI |
| 2.5 Prior Ultrasound Training Experience | NI |
|  | |
| **3. Intervention Characteristics (According to ADDIE Training Model)** | |
| **Item** | **Extracted Data** |
| 3.1 Analysis | 1. Trainee Group:    1. Participants: 32 emergency medicine (EM) residents (PGY-1 to PGY-3).    2. Roles:       1. 12 active learners performing hands-on simulations.       2. 20 observers engaging in discussions and debriefing. 2. Training Needs:    1. Clinical Requirement:       1. Emergency assessment of first-trimester pregnancy complications:          1. Intrauterine pregnancy (IUP) confirmation.          2. Ectopic pregnancy detection and hemorrhagic shock management.          3. Identification of non-obstetric causes of abdominal pain (e.g., appendicitis in pregnancy).       2. Integration of bedside transvaginal ultrasound (TVUS) into emergency workflow to enhance diagnostic speed and patient outcomes.    2. Skill Development:       1. Hands-on experience in transvaginal point-of-care ultrasound (TVPOCUS) to improve:          1. Probe insertion, orientation, and sweeping techniques.          2. Ultrasound image interpretation and clinical decision-making.          3. Proper disinfection and storage of endocavitary probes. 3. Baseline Survey (Pre-Training Assessment):    1. Identified Gaps:       1. Limited confidence and hands-on experience in TVUS application for emergency cases.       2. Difficulties in image interpretation and procedural unfamiliarity.       3. Need for structured simulation-based training to build proficiency in TVPOCUS.    2. Supporting Data:       1. PGY-1 residents: Confidence median score 1 (“not confident”).       2. PGY-2/PGY-3 residents: Confidence median score 3 (“neutral”).       3. Most significant deficiencies:          1. Clinical application of TVUS.          2. Technical skills (probe insertion and manipulation).          3. Workflow integration in emergency settings. |
| 3.2 Design | 1. Training Objectives:    1. By the end of the session, trainees should be able to:       1. Recognize clinical indications for transvaginal ultrasound (TVUS) in the emergency department.       2. Perform probe insertion, orientation, and scanning techniques.       3. Interpret TVUS images for intrauterine pregnancy (IUP) and other pathologies.       4. Implement infection control protocols for probe disinfection and storage. 2. Training Methods:    1. High-fidelity simulation for hands-on practice in a controlled setting.    2. Problem-Based Learning (PBL) and case-driven scenarios for skill enhancement. 3. Training Content & Structure:    1. Three High-Fidelity Simulation Cases:    2. IUP confirmation using TVPOCUS.    3. Ectopic pregnancy with hemorrhagic shock.    4. Pregnancy complicated by appendicitis. |
| 3.3 Development | 1. Instructors & Training Staff:    1. Ultrasound faculty & emergency medicine specialists provide hands-on guidance. 2. Teaching Aids & Equipment:    1. Electronic platforms for image analysis & case studies.    2. Simulation tools:       1. High-fidelity female mannequin with vaginal canal for probe insertion.       2. Ultrasound machines for real-time scanning practice. 3. Teaching Materials:    1. Course syllabus, lecture slides, case studies, test questions, and assessment rubrics. |
| 3.4 Implementation | 1. Time & Schedule:    1. Total duration: Half-day session.    2. Morning: Theoretical instruction.    3. Afternoon: Simulation practice & case discussions. 2. Location & Setting:    1. Conducted in an emergency medicine training center with ultrasound facilities. 3. Participants:    1. 32 EM residents (PGY-1 to PGY-3).    2. Ultrasound-trained faculty members as instructors. 4. Execution Process:    1. Theoretical lecture on TVUS fundamentals.    2. Hands-on simulation training with guided feedback.    3. Case-based scenario application & discussion.    4. Debriefing and Q&A session for knowledge reinforcement. 5. Adaptation Records:    1. Extended simulation time based on participant feedback.    2. Modified case sequence to improve session flow and engagement. |
| 3.5 Evaluation | 1. Formative Evaluation (Ongoing Assessment):    1. Purpose: Monitor learning progress and identify areas for improvement.    2. Methods: Direct observation, oral feedback, and real-time coaching.    3. Results: Participants showed gradual improvement in probe handling and image interpretation. 2. Summative Evaluation (Post-Training Assessment):    1. Purpose: Measure effectiveness of the training.    2. Methods:       1. Pre- & post-training surveys using a Likert scale for confidence assessment.    3. Results:       1. PGY-1 confidence improved from 1 (“not confident”) to 4 (“confident”).       2. PGY-2/PGY-3 confidence improved from 3 (“neutral”) to 4 (“confident”). 3. Follow-Up Evaluation:    1. Purpose: Assess clinical application post-training.    2. Methods: Faculty follow-up on TVUS utilization in real-world settings.    3. Results: Increased willingness among trainees to use TVPOCUS in emergency practice. |
|  | |
| **4. Outcome Characteristics** | |
| **Item** | **Extracted Data** |
| **4.1 Training Outcomes** |  |
| 4.1.1 Diagnostic Accuracy | NI |
| 4.1.2 Knowledge Acquisition | NI |
| 4.1.3 Skills Acquisition | 1. Confidence Improvement:    1. Workflow integration: Median Likert score 1.5 to 4    2. Probe insertion and orientation: Median Likert score 2.5 to 5    3. Overall confidence:       1. PGY-1: Median Likert score 1 to 4       2. PGY-2/PGY-3: Median Likert score 3 to 4 2. Participant Feedback:    1. Participants valued hands-on practice with probe handling, insertion, and sweeping motions in a controlled and safe training environment. |
| 4.1.4 Knowledge Retention | NI |
| 4.1.5 Practical Application | NI |
| **4.2 Maternal and Neonatal Health Outcomes** (Including Physiological and Psychological Outcomes) | NI |
| **4.3 Health Economic Outcomes** | NI |

**JBI Qualitative Data Extraction Tool**

**Reviewer: XXX & XXX & XXX Date: 27^th^ February 2025**

**Author: Hall et al. Year: 2021**

**Journal: The Ultrasound Journal Record Number: 4**

| **1. Study Characteristics** | |
| --- | --- |
| **Item** | **Extracted Data** |
| 1.1 Author (Publication Year) | Hall et al., 2021 |
| 1.2 Country | United Republic of Tanzania |
| 1.3 Location | Pemba Island, Zanzibar, Tanzania  This includes the specific training sites:   1. Chake Chake District Hospital 2. Wete District Hospital 3. Micheweni Public Health Care Center (PHCC) |
| 1.4 Study Design | Quasi-experimental study |
| 1.5 Sample Size | 13 |
|  | |
| **2. Population Characteristics (Healthcare Providers Receiving POCUS Training)** | |
| **Item** | **Extracted Data** |
| 2.1 Age | NI |
| 2.2 Gender | 1. Male: 30.8% (4/13) 2. Female: 69.2% (9/13) |
| 2.3 Professional Role | 1. Midwives/Nurse: 61.5% (8/13) 2. Clinical officer: 23.1% (3/13) 3. Medical officer/Physician: 15.4% (2/13) |
| 2.4 Work Experience (Duration) | NI |
| 2.5 Prior Ultrasound Training Experience | 1. None: 84.6% (11/13) 2. Informal experience: 7.7% (1/13) 3. Formal education: 7.7% (1/13) |
|  | |
| **3. Intervention Characteristics (According to ADDIE Training Model)** | |
| **Item** | **Extracted Data** |
| 3.1 Analysis | 1. Trainee Group:    1. The trainees were antenatal healthcare practitioners in rural Zanzibar, including midwives, clinical officers, and physicians.    2. Participants were selected based on hospital and community needs, their interest in ultrasound, and proficiency in English.    3. 85% had no prior ultrasound training, and none routinely performed ultrasound in their practice before the program. 2. Needs Analysis:    1. Conducted by PURE (Point-of-care Ultrasound in Resource-limited Environments) in 2015-2016.    2. Identified antenatal and obstetric ultrasound training as the most valuable intervention for improving maternal healthcare.    3. Focused on building ultrasound capacity in primary healthcare units (PHCUs) and district hospitals. 3. Baseline Survey:    1. Pre-training assessment:       1. Written exam average score: 33.7% (indicating low baseline knowledge).       2. No structured clinical ultrasound experience among trainees. 4. Impact on Instructional Design:    1. Training completion criteria:       1. ≥75% on the written exam.       2. ≥85% on OSCE.       3. At least 75 proctored ultrasound exams completed.    2. Adjustments were made based on challenges encountered, e.g., femur length measurement was excluded from final requirements due to difficulties in mastery. |
| 3.2 Design | 1. Training Objectives:    1. Develop competency in key ultrasound skills, including:       1. Basic ultrasound physics.       2. Gestational age estimation.       3. Fetal presentation and biometry.       4. Placental location and amniotic fluid assessment.       5. Identification of obstetric abnormalities.       6. Integration of ultrasound into clinical decision-making. 2. Training Methods:    1. 2-week intensive classroom training (lectures + hands-on scanning).    2. 6-month on-site longitudinal supervision and practical training.    3. Detailed structure of practical training:    4. A dedicated ultrasound trainer was continually present in Zanzibar to provide direct proctoring and review of recorded exams.    5. Trainers rotated among three training sites weekly, ensuring:       1. Two sites received two visits per week.       2. One site received one visit per week.    6. Each trainee had 6-7 hours of in-person supervision per week.    7. Training time varied due to factors such as:       1. Number of patients requiring ultrasound.       2. Clinical workload of trainees.       3. Machine availability at training sites. 3. Training Content/Plan:    1. Based on WHO Antenatal Care Guidelines and AIUM-ACR-ACOG-SMFM-SRU Practice Parameters.    2. Training followed a progressive structure, moving from fundamental skills to real-world clinical applications.    3. The course combined theoretical knowledge, practical skill-building, and clinical case integration. |
| 3.3 Development | 1. Instructors:    1. Training was delivered by a multidisciplinary team:       1. 4 emergency physicians (including ultrasound fellowship-trained experts).       2. 2 obstetricians/gynecologists.    2. Trainers provided continuous feedback, image review, and case-based learning. 2. Teaching Aids:    1. Three portable SonoSite M-Turbo ultrasound machines were deployed across hospital sites.    2. Each machine had:       1. Abdominal and linear transducers for fetal and maternal imaging.       2. Battery backup to address power supply issues in remote settings. 3. Teaching Materials:    1. Lesson plans, lecture notes, PPTs, written tests, and OSCE assessments.    2. Based on WHO recommendations for ultrasound in antenatal care. |
| 3.4 Implementation | 1. Time:    1. 2-week full-time classroom training.    2. 6-month on-site supervision, with trainees receiving 6-7 hours of weekly direct guidance. 2. Location/Setting:    1. Training conducted at district hospitals and Public Health Care Centers (PHCCs) in Pemba, Zanzibar.    2. Sites selected based on ultrasound service gaps and community healthcare needs. 3. Participants:    1. Trainers: Experienced emergency medicine and obstetrics specialists.    2. Trainees: Midwives, clinical officers, and physicians from Zanzibar's healthcare system. 4. Execution Process:    1. Trainees performed an average of 99 ultrasound exams during training, with a total of 1,338 proctored scans completed.    2. Emphasis on direct skill application in clinical settings. 5. Adaptation Records:    1. Training adjustments included:       1. Exclusion of femur length measurement from the OSCE due to learning difficulties.       2. Modifications in training delivery due to equipment maintenance issues.       3. Tailored feedback sessions to address individual skill gaps. |
| 3.5 Evaluation | 1. Formative Evaluation:    1. Mid-course OSCE (week 19):       1. Average score 71.2%, indicating progress but highlighting areas needing improvement.    2. Final OSCE (week 27):       1. Average score 84.7%, showing significant skill acquisition. 2. Summative Evaluation:    1. 62% (8 out of 13 trainees) successfully met all training requirements.    2. Final written exam average score: 77.5%.    3. Strong correlation between higher scan volume and better OSCE performance. 3. Follow-up:    1. Ongoing assessment needed to determine long-term skill retention.    2. Plans to implement a “Train-the-Trainer” model to sustain and expand the program.    3. Further research required to evaluate the impact of ultrasound training on clinical outcomes. |
|  | |
| **4. Outcome Characteristics** | |
| **Item** | **Extracted Data** |
| **4.1 Training Outcomes** |  |
| 4.1.1 Diagnostic Accuracy | NI |
| 4.1.2 Knowledge Acquisition | 1. Knowledge Improvement:    1. Mean Scores: Improved from 33.7% (SD 9.4%) to 77.5% (SD 11.9%). “Written exam scores improved from a mean of 33.7% (95% *CI*: 28.6 - 38.8%) at pre-course assessment to 77.5% (95% *CI*: 71 - 84%) at course completion (*P* < 0.0001).”    2. Median Scores: Increased from 37.5% to 82.1%.    3. By Profession:       1. Physicians: 87.5 ± 2.5%.       2. Clinical Officers: 80.9 ± 5.4%.       3. Nurses/Midwives: 73.7 ± 13.5%.    4. Pass Rates:       1. Pre-course: 0% passed.       2. Post-course: 76.9% passed (10/13 trainees). |
| 4.1.3 Skills Acquisition | 1. OSCE Scores:    1. Mean Scores: Increased from 71.2% (95% *CI*: 62.3 - 80.1%) to 84.7% (95% *CI*: 78.5 - 90.8%). 2. By Profession:    1. Physicians: 93 ± 1%.    2. Clinical Officers: 92.7 ± 5.6%.    3. Nurses/Midwives: 79.7 ± 11.2%. 3. Correlation with Scan Numbers:    1. Weak correlation between OSCE performance and number of scans performed (*p* = 0.034, *r²* = 0.349). |
| 4.1.4 Knowledge Retention | 1. OSCE Performance Over Time:    1. Scores improved from 71.1% at 19 weeks to 86.7% at 27 weeks.    2. Pass Rates:       1. Mid-course (19 weeks): 23.1% (3/13).       2. Final exam (27 weeks): 46.2% (6/13). |
| 4.1.5 Practical Application | 1. Number of Scans Performed:    1. Total: 1,338 scans.    2. Per Trainee: Average 99 scans (range: 42-128; median: 109).    3. Successful Trainees: 112 ± 9.4 scans.    4. Unsuccessful Trainees: 76.6 ± 33.2 scans. |
| **4.2 Maternal and Neonatal Health Outcomes** (Including Physiological and Psychological Outcomes) | NI |
| **4.3 Health Economic Outcomes** | NI |

**JBI Qualitative Data Extraction Tool**

**Reviewer: XXX & XXX & XXX Date: 27^th^ February 2025**

**Author: Kimberly et al. Year: 2010**

**Journal: Ultrasound In Medicine &Biology Record Number: 5**

| **1. Study Characteristics** | |
| --- | --- |
| **Item** | **Extracted Data** |
| 1.1 Author (Publication Year) | Kimberly et al., 2010 |
| 1.2 Country | Zambia |
| 1.3 Location | At three rural sites in the Kapiri Mposhi District of the Central Province of Zambia.  Three rural sites:   1. Kapiri District Hospital 2. Mukonchi Rural Health Center 3. Nkole Rural Health Center |
| 1.4 Study Design | Quasi-experimental study |
| 1.5 Sample Size | 21 |
|  | |
| **2. Population Characteristics (Healthcare Providers Receiving POCUS Training)** | |
| **Item** | **Extracted Data** |
| 2.1 Age | NI |
| 2.2 Gender | NI |
| 2.3 Professional Role | Midwives: 100% (21/21) |
| 2.4 Work Experience (Duration) | NI |
| 2.5 Prior Ultrasound Training Experience | None (0/21) |
|  | |
| **3. Intervention Characteristics (According to ADDIE Training Model)** | |
| **Item** | **Extracted Data** |
| 3.1 Analysis | 1. Trainee Group:    1. 21 midwives from rural Zambia, with no prior ultrasound training.    2. Practicing in three rural health facilities without ultrasound access.    3. Responsible for maternal care in areas with limited obstetricians. 2. Needs Analysis:    1. High maternal mortality (591 per 100,000 live births) due to limited imaging access.    2. Only 33% of rural women deliver in health facilities.    3. UN Millennium Development Goal: Reduce maternal mortality by 75%.    4. Ultrasound may improve decision-making and increase facility-based births. 3. Baseline Survey:    1. No ultrasound access or prior training at study sites.    2. Limited referral capabilities for complications.    3. Ethical approval obtained from local health authorities. 4. Impact on Instructional Design:    1. Training focused on:       1. Machine operation (powering on/off, inputting data, saving images).       2. Core obstetric applications (fetal presentation, heart rate, placental position, gestational age).       3. Clinical decision-making based on ultrasound findings.    2. Structured for progressive skill-building: theory, hands-on practice, OSCE evaluation. |
| 3.2 Design | 1. Training Objectives:    1. Teach midwives basic obstetric ultrasound skills.    2. Develop the ability to identify key obstetric indicators:       1. Fetal number, presentation, heart rate, placental location, gestational age.    3. Enable integration of ultrasound findings into clinical decision-making. 2. Training Methods:    1. Phased approach:       1. Initial 2-3 weeks: Didactic sessions (2-3 hours per site) + supervised hands-on scanning.       2. Independent scanning (2-3 months) with minimal supervision.       3. Follow-up training & OSCE assessment at 6 months. 3. Training Content/Plan/Syllabus:    1. Machine operation: Powering on/off, data entry, image saving, maintenance.    2. Basic obstetric ultrasound skills:       1. Fetal presentation (vertex/breech).       2. Fetal heart rate measurement.       3. Placental location.       4. Gestational age estimation (BPD, femur length). 4. Data Recording & Review: All images saved; structured data sheets completed. |
| 3.3 Development | 1. Instructors/Teachers:    1. Three emergency ultrasound fellowship-trained physicians conducted the training.    2. Training included hands-on supervision, OSCE evaluations, and skill retention assessments.    3. Visiting obstetricians and emergency physicians provided additional mentorship. 2. Teaching Aids:    1. Four portable ultrasound machines (SonoSite 180) were provided.    2. Each machine included:       1. Curved array abdominal probe.       2. Extra battery for portability.       3. Software for image uploading and storage.    3. Machines were distributed as follows:       1. Two at Kapiri District Hospital (labor ward + maternal clinic).       2. One at Mukonchi Rural Health Center.       3. One at Nkole Rural Health Center. 3. Teaching Materials:    1. Lesson plans & lecture notes covering:       1. Ultrasound operation & maintenance.       2. Basic obstetric ultrasound techniques.    2. Training tools included:       1. PPT slides & printed materials.       2. OSCE checklists for skill evaluation.       3. Structured data sheets for tracking scans.    3. Development process:       1. Training materials were refined based on feedback from midwives and trainers.       2. Initial data sheets were found to be too complex, leading to simplification for better compliance. |
| 3.4 Implementation | 1. Time:    1. Training spanned 6 months, followed by a 1-year follow-up.    2. Training schedule:       1. Phase 1: 2-3 weeks of intensive training.       2. Phase 2: 2-3 months of independent practice.       3. Phase 3: Second training with OSCE at 6 months.       4. Phase 4: 1-year follow-up to assess skill retention. 2. Location/Setting:    1. Three study sites in Kapiri Mposhi District, Zambia:       1. Kapiri District Hospital (main regional hospital).       2. Mukonchi Rural Health Center.       3. Nkole Rural Health Center.    2. None of these facilities had ultrasound before the study. 3. Participants:    1. 21 midwives trained.    2. Pregnant women presenting at antenatal clinics or labor wards were scanned.    3. Inclusion criteria: Patients were scanned based on midwife availability.    4. Consent: Patients provided verbal informed consent. 4. Execution Process:    1. 441 ultrasound scans recorded over 6 months.    2. Supervised scans:       1. 43% were supervised by trainers.       2. The rest were performed independently.    3. Most scans were conducted in the second or third trimester.    4. Common indications:       1. Size vs. dates discrepancy (44%).       2. Fetal position check (39%).       3. Vaginal bleeding (7%). 5. Adaptation Records:    1. Challenges identified & adaptations made:       1. Variability in scanning frequency among midwives:          1. Some performed many scans (up to 179).          2. Others performed very few (only 1).    2. Administrative challenges:       1. Image storage & data entry compliance was low.       2. Data sheets were simplified for better usability. 6. Equipment Issues:    1. One ultrasound machine malfunctioned and was repaired.    2. Some sites ran out of ultrasound gel, affecting usage. |
| 3.5 Evaluation | 1. Formative Evaluation:    1. Purpose: Assess skill development and knowledge retention.    2. Method: OSCE evaluations at 2 months and 6 months.    3. Results:       1. 2-month OSCE average score: 10.0/14 (71%).       2. 6-month OSCE average score: 11.6/14 (83%).    4. Difficult tasks:       1. Changing batteries (72% accuracy).       2. Entering patient data (52%).       3. Using ultrasound presets for fetal heart rate (48%). 2. Summative Evaluation:    1. Impact on clinical decision-making:       1. 17% of scans changed management:          1. 36% led to repeat ultrasound.          2. 23% increased antenatal visits.          3. 18% led to referrals.    2. 1-year follow-up:       1. Midwives continued scanning (~10 scans/week).       2. 100% reported ultrasound changed their practice. 3. Follow-up:    1. Challenges at 1-year review:       1. 46% reported time constraints.       2. 38% faced equipment problems.       3. Increased patient demand for ultrasounds. |
|  | |
| **4. Outcome Characteristics** | |
| **Item** | **Extracted Data** |
| **4.1 Training Outcomes** |  |
| 4.1.1 Diagnostic Accuracy | 1. Fetal Heart Rate (FHR): Accuracy of interpretation: 96%. 2. Placental Location: Accuracy of interpretation: 91%. 3. Biparietal Diameter (BPD): 70% of measurements were off-axis, leading to likely incorrect gestational age assessment. 4. Femur Length: Consistency too low for meaningful evaluation. |
| 4.1.2 Knowledge Acquisition | NI |
| 4.1.3 Skills Acquisition | 1. Observed Structured Clinical Examination (OSCE):    1. At 2 months: Mean score was 10.0/14 (71.4%) with SD: 3.9.    2. At 6 months: Mean score was 11.6/14 (82.9%) with SD: 1.8.    3. Paired t-test: No significant difference between scores at 2 and 6 months (p = 0.15). 2. Most Successfully Acquired Skills:    1. Identifying the number of gestations: 100%.    2. Determining fetal presentation: 96%.    3. Operating the ultrasound machine (turning on/off): 96%. 3. More Challenging Tasks:    1. Changing the battery: 72%.    2. Entering patient data: 52%.    3. Using presets to calculate FHR: 48%. |
| 4.1.4 Knowledge Retention | 1. Skills were retained over 6 months, with a slight improvement in OSCE scores. 2. No significant statistical difference between 2-month and 6-month OSCE scores (*p* = 0.15). |
| 4.1.5 Practical Application | 1. Ultrasounds Performed:    1. Kapiri District Hospital: 170 scans (38.5%).    2. Mukonchi Rural Health Clinic: 64 scans (14.5%).    3. Nkole Rural Health Clinic: 207 scans (46.9%).    4. Total: 441 scans. 2. Changes in Clinical Decision-Making:    1. Total cases: 17% (74/441).       1. Repeat ultrasound: 36%.       2. Increased antenatal visits: 23%.       3. Referral for advanced care: 18%.       4. Other actions (e.g., induction, observation): 23%. 3. Clinical Conditions Identified:    1. Non-vertex presentation: 61% (76/124).    2. Multiple gestations: 24% (30/124).    3. No fetal heart rate (FHR): 8% (10/124).    4. Low-lying placenta: 2% (3/124).    5. Other findings: Ectopic pregnancies, polyhydramnios, and fetal distress. 4. Challenges:    1. Time constraints during clinical schedules: 46% of midwives reported significant limitations.    2. Equipment-related issues: 38% reported technical problems (e.g., lack of ultrasound gel, screen malfunction). |
| **4.2 Maternal and Neonatal Health Outcomes** (Including Physiological and Psychological Outcomes) | 1. Increased Clinic Births:    1. Mukonchi Clinic: +11%.    2. Nkole Clinic: +36% (accompanied by the addition of a new birthing center). 2. Psychological benefits: Patients reassured upon seeing fetal heartbeat during ultrasounds. |
| **4.3 Health Economic Outcomes** | NI |

**JBI Qualitative Data Extraction Tool**

**Reviewer: XXX & XXX & XXX Date: 27^th^ February 2025**

**Author: Kolbe et al. Year: 2014**

**Journal: Journal of Ultrasound Record Number: 6**

| **1. Study Characteristics** | |
| --- | --- |
| **Item** | **Extracted Data** |
| 1.1 Author (Publication Year) | Kolbe et al., 2014 |
| 1.2 Country | Nicaragua |
| 1.3 Location | Las Salinas, a small rural village in western Nicaragua. |
| 1.4 Study Design | Quasi-experimental study |
| 1.5 Sample Size | 4 |
|  | |
| **2. Population Characteristics (Healthcare Providers Receiving POCUS Training)** | |
| **Item** | **Extracted Data** |
| 2.1 Age | NI |
| 2.2 Gender | NI |
| 2.3 Professional Role | 1. Doctors: 50% (2/4) 2. Nurses: 25% (1/4) 3. Nursing assistants: 25% (1/4) |
| 2.4 Work Experience (Duration) | NI |
| 2.5 Prior Ultrasound Training Experience | NI |
|  | |
| **3. Intervention Characteristics (According to ADDIE Training Model)** | |
| **Item** | **Extracted Data** |
| 3.1 Analysis | 1. Trainee Group:    1. Trainees: Two postgraduate physicians, one head clinic nurse, one nursing assistant.    2. Goal: Train local healthcare workers to use POCUS for diagnostic and treatment decisions. 2. Needs Analysis:    1. Medical limitations in the region:       1. No local medical imaging was available.       2. Nearest hospital was over 7 hours away by car.       3. High demand for prenatal care and abdominal pain diagnosis.    2. Challenges identified:       1. Lack of prior ultrasound training among local practitioners.       2. Need for sustainable training and continued supervision. 3. Baseline Survey:    1. Before POCUS implementation, all diagnostic decisions were made based solely on clinical symptoms without imaging confirmation.    2. Patients had to travel long distances for basic ultrasound services. 4. Impact on Instructional Design:    1. Training structure was designed to address:       1. The knowledge gap in sonographic diagnostics.       2. The need for practical, hands-on training.       3. The importance of follow-up support through telemedicine to reinforce learning. |
| 3.2 Design | 1. Training Objectives:    1. Knowledge: Understanding the basics of ultrasound physics and image interpretation.    2. Skills: Learning to operate ultrasound devices and recognize common pathologies.    3. Attitude: Encouraging reliance on ultrasound for diagnosis instead of empirical treatment. 2. Training Methods:    1. Phase 1: On-site Training       1. Conducted by international ultrasound experts.       2. Included daily didactic sessions and hands-on workshops.    2. Phase 2: Remote Training       1. Weekly telemedicine sessions (60-90 minutes each) for 3 months.       2. Real-time feedback on ultrasound scans via Skype and Epiphan VGA2USB. 3. Training Content/Plan/Syllabus:    1. Training followed a progressive structure:       1. Basic topics: Introduction to ultrasound physics, probe handling.       2. Intermediate topics: Common findings in abdominal, cardiac, and obstetric ultrasound.       3. Advanced topics: Identifying pathologies such as kidney stones, ovarian cysts, pleural effusion. |
| 3.3 Development | 1. Instructors/Teachers:    1. Identity & Qualifications:       1. Experts from Henry Ford Hospital, Ultrasound University, and Mission of Grace.    2. Instructor-to-Trainee Ratio:       1. Small-group teaching for personalized feedback.    3. Instructor Training Program:       1. Remote experts graded performance from 1-10, allowing for skill progression. 2. Teaching Aids:    1. Equipment Provided:       1. Sonosite Titan ultrasound machine (donated to the clinic).       2. Three laptops for telemedicine sessions.    2. Online Learning Support:       1. A Spanish-translated ultrasound manual for continued reference. 3. Teaching Materials:    1. Developed resources included:       1. Lesson plans covering diagnostic techniques.       2. Lecture notes on sonographic interpretation.       3. PPT presentations with case studies.       4. Test questions for knowledge evaluation.       5. Checklists for skill assessment. |
| 3.4 Implementation | 1. Time:    1. Duration: 3 months.    2. Daily Schedule: Morning theory + Afternoon patient scans. 2. Location/Setting:    1. Rural clinic in Las Salinas, Nicaragua.    2. Patients traveled long distances to access medical care. 3. Participants (Trainer/Trainee):    1. Trainers: International ultrasound experts.    2. Trainees: Local physicians, nurses, and assistants. 4. Execution Process:    1. Clinical assessment by trainees.    2. POCUS examination performed.    3. Diagnosis confirmed or modified based on findings.    4. Remote experts provided feedback. 5. Adaptation Records:    1. Initial sessions fully guided, but autonomy increased over time.   Performance graded on a 1-10 scale to measure improvement. |
| 3.5 Evaluation | 1. Formative Evaluation (Process Evaluation):    1. Methods: Real-time review of ultrasound images via telemedicine.    2. Performance Scores:       1. Initial average: 6.54.       2. Final average: 7.17. 2. Summative Evaluation (Outcome Evaluation):    1. Impact on patient care:       1. 132 patients scanned.       2. 52% received new diagnoses.       3. 48% had changes in management. 3. Follow-up:    1. Trainees continued using POCUS independently.    2. Remote support was reduced but maintained for sustainability. |
|  | |
| **4. Outcome Characteristics** | |
| **Item** | **Extracted Data** |
| **4.1 Training Outcomes** |  |
| 4.1.1 Diagnostic Accuracy | NI |
| 4.1.2 Knowledge Acquisition | NI |
| 4.1.3 Skills Acquisition | 1. Skill assessment method: Performance score (1-10 scale) assigned by remote experts. 2. Scores over time:    1. First 6 weeks: Mean score 6.54    2. Final 6 weeks: Mean score 7.17    3. Overall average score: 6.85 3. 52% (70/132) of ultrasound scans were formally evaluated by remote experts. |
| 4.1.4 Knowledge Retention | 1. Retention Assessment: Skill scores showed continued improvement over 3 months. 2. Evidence Of Sustained Learning: Final scores were higher than initial scores (6.54 → 7.17). |
| 4.1.5 Practical Application | 1. Impact of POCUS on Clinical Management:    1. Total patients who underwent POCUS evaluation: 132    2. Patients with new diagnoses identified via POCUS: 69/132 (52.27%)    3. Patients whose management was changed due to POCUS findings:       1. All cases: 64/132 (48.48%), 95% CI: 39.96–57.01%       2. Excluding pregnancy cases: 62/101 (61.39%), 95% CI: 51.89–70.88% 2. Types of Management Changes Due to POCUS Findings:    1. Initiation of new treatment    2. Referral for further evaluation    3. Immediate interventions |
| **4.2 Maternal and Neonatal Health Outcomes** (Including Physiological and Psychological Outcomes) | NI |
| **4.3 Health Economic Outcomes** | NI |

**JBI Qualitative Data Extraction Tool**

**Reviewer: XXX & XXX & XXX Date: 28^th^ February 2025**

**Author: Lee et al. Year: 2015**

**Journal: Global Humanitarian Technology Conference Record Number: 7**

| **1. Study Characteristics** | |
| --- | --- |
| **Item** | **Extracted Data** |
| 1.1 Author (Publication Year) | Lee et al., 2015 |
| 1.2 Country | Uganda |
| 1.3 Location | Bwindi Nursing School, Bwindi, Kanungu District, Uganda |
| 1.4 Study Design | Quasi-experimental study |
| 1.5 Sample Size | 22 |
|  | |
| **2. Population Characteristics (Healthcare Providers Receiving POCUS Training)** | |
| **Item** | **Extracted Data** |
| 2.1 Age | Range: 18-26 |
| 2.2 Gender | 1. Male: 72.73% (16/22) 2. Female: 27.27% (6/22) |
| 2.3 Professional Role | Students: 100% (22/22) |
| 2.4 Work Experience (Duration) | NI |
| 2.5 Prior Ultrasound Training Experience | None (0/22) |
|  | |
| **3. Intervention Characteristics (According to ADDIE Training Model)** | |
| **Item** | **Extracted Data** |
| 3.1 Analysis | 1. Trainee Group:    1. The trainees were nursing students at Uganda Nursing School Bwindi (UNSB), selected to develop obstetric and gynecologic ultrasound skills to serve rural healthcare facilities.       1. 22 students enrolled (6 female, 16 male), aged 18-26.       2. Many traveled long distances to attend the program. 2. Needs Analysis:    1. Uganda has a high maternal mortality rate (438 per 100,000 live births), worsened by a severe shortage of healthcare professionals and imaging technology. The introduction of ultrasound training was expected to significantly improve maternal health outcomes. 3. Baseline Survey:    1. Before UNSB’s program, Imaging the World (ITW) had integrated obstetric ultrasound training into rural Ugandan clinics, showing success in early risk detection and referral systems. The program at UNSB aimed to scale this effort and build long-term capacity. 4. Impact on Instructional Design:    1. Due to unreliable internet, electricity issues, and limited local expertise, the program adopted a hybrid training model (online + hands-on sessions).       1. Adapted from U.S. radiology residency training but tailored for local challenges. |
| 3.2 Design | 1. Training Objectives:    1. The curriculum was designed to achieve:    2. Knowledge: Understanding ultrasound physics, anatomy, and pathology.    3. Skills: Mastering hands-on ultrasound scanning and optimizing imaging parameters.    4. Attitude: Gaining confidence to use ultrasound independently in clinical settings. 2. Training Methods:    1. Online video modules for foundational knowledge.    2. Four on-site practical training sessions per semester, supervised by Ugandan sonographers.    3. Blended learning approach combining videos, lectures, and practical exercises. 3. Training Content/Plan/Syllabus:    1. Core topics: Ultrasound physics, clinical applications, diagnostic techniques.    2. Weekly quizzes and a final 30-question exam.    3. Goal: Train nurses to independently conduct ultrasound scans. |
| 3.3 Development | 1. Instructors/Teachers:    1. Faculty included U.S. radiology experts, Ugandan sonographers, and UNSB faculty.    2. Long-term goal: Transition course leadership to local instructors. 2. Teaching Aids:    1. Online platform for lectures and quizzes.    2. Tablet devices for students to access the course.    3. Portable ultrasound machines for hands-on training. 3. Teaching Materials:    1. 10-15 min pre-recorded video lectures (unlimited replays).    2. Lecture notes on physics, anatomy, and pathology.    3. Hands-on scanning of real patients in sonography labs. |
| 3.4 Implementation | 1. Time Schedule:    1. Three-year program integrated into UNSB’s nursing curriculum.    2. Weekly structured online lectures and quizzes.    3. Four hands-on training sessions per semester. 2. Location/Setting:    1. Online learning in UNSB’s library or personal devices.    2. Hands-on training conducted at Bwindi Community Hospital (BCH). 3. Execution Process:    1. Online modules completed before hands-on practice.    2. Practical training involved real patient scanning. 4. Adaptation Records:    1. Group video screenings scheduled to overcome internet disruptions.    2. Basic IT training introduced to help students navigate the online platform. |
| 3.5 Evaluation | 1. Formative Evaluation:    1. Weekly quizzes, practical skill assessments, and faculty feedback.    2. Student surveys evaluated video clarity, content difficulty, and hands-on experiences. 2. Summative Evaluation:    1. Final 30-question exam + practical skills evaluation.    2. All students passed (≥80%), confirming program effectiveness. 3. Follow-up:    1. Graduates committed to working in rural Uganda for three years.    2. Post-graduation follow-ups planned to evaluate long-term impact. |
|  | |
| **4. Outcome Characteristics** | |
| **Item** | **Extracted Data** |
| **4.1 Training Outcomes** |  |
| 4.1.1 Diagnostic Accuracy | NI |
| 4.1.2 Knowledge Acquisition | Pass rate: 100% (22/22 students) |
| 4.1.3 Skills Acquisition | NI |
| 4.1.4 Knowledge Retention | NI |
| 4.1.5 Practical Application | NI |
| **4.2 Maternal and Neonatal Health Outcomes** (Including Physiological and Psychological Outcomes) | 1. Antenatal visits increased: 70% (baseline numbers not provided)   The integration of ultrasound training at the Uganda Nursing School Bwindi (UNSB) has led to a 70% increase in antenatal visits, though baseline numbers were not provided.  This increase is likely due to:   - 1. Early Risk Detection & Referral: Trained students can identify high-risk pregnancies and ensure timely medical intervention.   2. Greater Patient Confidence: Seeing ultrasound images reassures pregnant women, encouraging them to attend more check-ups.   3. Improved Maternal Care: More antenatal visits have helped detect and treat malaria, HIV, parasites, tetanus, and anemia. |
| **4.3 Health Economic Outcomes** | NI |

**JBI Qualitative Data Extraction Tool**

**Reviewer: XXX & XXX & XXX Date: 28^th^ February 2025**

**Author: Vinayak & Brownie Year: 2018**

**Journal: Journal of Interprofessional Care Record Number: 8**

| **1. Study Characteristics** | |
| --- | --- |
| **Item** | **Extracted Data** |
| 1.1 Author (Publication Year) | Vinayak & Brownie, 2018 |
| 1.2 Country | Kenya |
| 1.3 Location | Nairobi, Kenya (Aga Khan University Hospital)  Pilot Sites: Kiambu (20 km from hospital), Embu (120 km from hospital), and Malindi (400 km from hospital). |
| 1.4 Study Design | Quasi-experimental study |
| 1.5 Sample Size | 9 |
|  | |
| **2. Population Characteristics (Healthcare Providers Receiving POCUS Training)** | |
| **Item** | **Extracted Data** |
| 2.1 Age | NI |
| 2.2 Gender | NI |
| 2.3 Professional Role | Midwives: 100% (9/9). |
| 2.4 Work Experience (Duration) | NI |
| 2.5 Prior Ultrasound Training Experience | None (0/9) |
|  | |
| **3. Intervention Characteristics (According to ADDIE Training Model)** | |
| **Item** | **Extracted Data** |
| 3.1 Analysis | 1. Trainee Group:    1. Target Group: Midwife sonographers    2. Purpose: Address shortage of radiologists & improve Point-Of-Care Ultrasound (POCUS) access. 2. Needs Analysis:    1. Problem:       1. High maternal & infant mortality rates.       2. Less than 5% of pregnant women have ultrasound access.       3. Shortage of trained radiologists & sonographers.       4. Undetected pregnancy risks lead to complications.    2. Solution:       1. Train midwives in basic ultrasound scanning.       2. Implement tele-radiology for expert verification. 3. Baseline Survey:    1. Conducted in three rural locations (Kiambu, Embu, Malindi).    2. Evaluated demand, midwives’ knowledge, and tech feasibility.    3. Findings:       1. High demand for ultrasound services.       2. Midwives had maternal health knowledge but no ultrasound skills.       3. Tele-radiology was viable for expert consultation. 4. Impact on Instructional Design:    1. Curriculum Adjustments:       1. Online + in-person blended learning.       2. Focus on basic ultrasound scanning & high-risk pregnancy detection.       3. Integrated tele-radiology for expert verification. |
| 3.2 Design | 1. Training Objectives:    1. Knowledge: Ultrasound principles & pregnancy risks.    2. Skills: Using ultrasound machines, identifying abnormalities.    3. Attitude: Promoting maternal health & teamwork. 2. Training Methods:    1. 4-week structured program:       1. 1 hour theory.       2. 6 hours practical training.       3. 1 hour review & feedback.    2. Remote expert guidance via tele-radiology. 3. Training Content/Plan:    1. Phase 1: Ultrasound basics.    2. Phase 2: Hands-on scanning techniques.    3. Phase 3: High-risk pregnancy identification.    4. Phase 4: Certification & assessment. |
| 3.3 Development | 1. Instructors/Teachers:    1. Expert Team: Radiologists, experienced sonographers, and tele-radiology specialists. 2. Teaching Aids:    1. Equipment:       1. Portable ultrasound machines.       2. Tele-radiology system for remote expert verification.    2. Software: CCC Teleradiology system. 3. Teaching Materials:    1. Learning Resources:       1. Digital training modules, PPTs, SOPs.       2. Case study database for reference. |
| 3.4 Implementation | 1. Time:    1. Training Duration: 4 weeks + ongoing supervision.    2. Scan time reduced from 45 to 20 minutes. 2. Location/Setting:    1. Training at Aga Khan University Hospital.    2. Field practice at 3 outreach clinics. 3. Participants:    1. Trainees: Midwives.    2. Trainers: Radiologists & sonographers.    3. Patients: Pregnant women receiving ultrasound. 4. Execution Process:    1. Training → hands-on scanning → tele-radiology verification. 5. Adaptation Records:    1. Adjusted training pace based on midwives’ progress.    2. Optimized tele-radiology workflow. |
| 3.5 Evaluation | 1. Formative Evaluation:    1. Methods: Daily assessments, expert feedback.    2. Results: Scanning efficiency improved. 2. Summative Evaluation:    1. Methods: Final certification & tele-radiology verification.    2. Results: 99.63% accuracy in detecting high-risk pregnancies. 3. Follow-up:    1. Methods: Ongoing remote monitoring.    2. Results: POCUS successfully integrated into rural healthcare. |
|  | |
| **4. Outcome Characteristics** | |
| **Item** | **Extracted Data** |
| **4.1 Training Outcomes** |  |
| 4.1.1 Diagnostic Accuracy | 99.63% accuracy in post-delivery follow-up (271 scans, 220 participants). |
| 4.1.2 Knowledge Acquisition | NI |
| 4.1.3 Skills Acquisition | Scan time reduced from 45 minutes to 20 minutes. |
| 4.1.4 Knowledge Retention | NI |
| 4.1.5 Practical Application | NI |
| **4.2 Maternal and Neonatal Health Outcomes** (Including Physiological and Psychological Outcomes) | NI |
| **4.3 Health Economic Outcomes** | NI |

**JBI Qualitative Data Extraction Tool**

**Reviewer: XXX & XXX & XXX Date: 28^th^ February 2025**

**Author: Wachira et al. Year: 2023**

**Journal: BMC Medical Education Record Number: 9**

| **1. Study Characteristics** | |
| --- | --- |
| **Item** | **Extracted Data** |
| 1.1 Author (Publication Year) | Wachira et al., 2023 |
| 1.2 Country | Kenya |
| 1.3 Location | 8 rural counties in Kenya (Kitui, Kilifi, Kakamega, Nakuru, Taita Taveta, Baringo, Samburu, Turkana) and nearby facilities such as Kenyatta University Skills Lab, Kibera, Kiandutu, Gatundu, Kiambu, Thika, Ruiru, University of Nairobi, and Aga Khan University Hospital. |
| 1.4 Study Design | Quasi-experimental study |
| 1.5 Sample Size | 514 |
|  | |
| **2. Population Characteristics (Healthcare Providers Receiving POCUS Training)** | |
| **Item** | **Extracted Data** |
| 2.1 Age | NI |
| 2.2 Gender | NI |
| 2.3 Professional Role | Most of the learners were non-physicians/non-sonographers in order to task-shift diagnostic ability to frontline providers since the majority of obstetric care is provided by them. |
| 2.4 Work Experience (Duration) | NI |
| 2.5 Prior Ultrasound Training Experience | Most participants had no prior ultrasound training and limited familiarity with the tools. "As POCUS is a relatively new technology, most frontline physicians in Low- and Middle-Income Countries (LMICs) have little or no experience with its use." |
|  | |
| **3. Intervention Characteristics (According to ADDIE Training Model)** | |
| **Item** | **Extracted Data** |
| 3.1 Analysis | 1. Trainee Group:    1. The training program targeted 514 mid-level healthcare providers (HCPs) from 8 rural counties in Kenya with poor maternal and neonatal health outcomes.    2. An additional 46 participants came from Kenyatta University (KU) and nearby healthcare facilities. 2. Needs Analysis:    1. In low- and middle-income countries (LMICs), limited access to ultrasound machines and training programs affects the quality of maternal healthcare.    2. WHO recommends that all pregnant women receive at least one ultrasound scan, but in LMICs, this is difficult due to a lack of equipment and trained personnel. 3. Baseline Survey:    1. Before training, participants completed an online pre-test to assess their baseline knowledge.    2. After the training, participants completed a post-test and an Objective Structured Clinical Examination (OSCE) to evaluate learning outcomes. 4. Impact on Instructional Design:    1. The five core topics covered in the training program were:    2. Multiple gestation (Identifying twin or multiple pregnancies)    3. Fetal presentation and lie (Checking if the baby is in the correct position for delivery)    4. Placental location (Determining whether the placenta is in a safe position)    5. Amniotic fluid assessment (Ensuring there is enough fluid for the baby)    6. Fetal heart rate assessment (Checking if the baby's heart is beating normally) |
| 3.2 Design | 1. Training Objectives:    1. The goal was to provide basic obstetric POCUS skills to nurses, midwives, clinical officers, and medical officers in a short, intensive 5-day training program. 2. Training Methods:    1. Blended learning approach, including:    2. Online resources (videos, literature)    3. Didactic lectures (classroom-based learning)    4. Live demonstrations (instructor-led ultrasound scanning)    5. Hands-on practice (scanning real pregnant women)    6. Remote mentorship (continued support via an online platform) 3. Training Content/Plan/Syllabus:    1. The program followed International Society of Ultrasound in Obstetrics and Gynecology (ISUOG) guidelines.    2. The course structure included:    3. Lectures on ultrasound fundamentals    4. Probe handling and scanning techniques    5. Practice using Butterfly iQ+ handheld ultrasound devices |
| 3.3 Development | 1. Instructors/Teachers:    1. The teaching team included:       1. Sonographers       2. Obstetrics and gynecology residents       3. Family medicine residents       4. Emergency physicians    2. The program included “Lunch and Learn” sessions to train the instructors themselves. 2. Teaching Aids:    1. Training Equipment:       1. Butterfly iQ+ handheld ultrasound devices       2. Apple iPads with preloaded learning materials    2. Remote support via GUSI online platform:       1. Participants uploaded their ultrasound scans for expert feedback. 3. Teaching Materials:    1. Lecture slides, PPTs, assessment tools    2. Logbooks: Each participant had to complete at least 20 scans per topic. |
| 3.4 Implementation | 1. Time Schedule:    1. 514 healthcare workers trained over 10 weeks    2. Each 5-day session trained 50 participants with 10 instructors 2. Location/Setting:    1. Training was conducted at:       1. Kenyatta University Skills Lab       2. Local hospitals and health facilities 3. Participants:    1. Nurses, midwives, clinical officers, radiographers, and sonographers participated.    2. The program focused on training non-physicians to expand diagnostic capabilities. 4. Execution Process:    1. Daily instructor reflection sessions ensured continuous improvement.    2. Extra support was given to struggling learners. |
| 3.5 Evaluation | 1. Formative Evaluation:    1. Daily logbooks and instructor feedback ensured learning progress. 2. Summative Evaluation:    1. Pre-test vs. Post-test scores:       1. Pre-test average: 52.8%       2. Post-test average: 90.6%    2. OSCE exam:       1. 99% pass rate (486/489 learners)       2. Average OSCE score: 87.3% 3. Follow-up:    1. Participants received continued GUSI platform support for further learning and feedback. |
|  | |
| **4. Outcome Characteristics** | |
| **Item** | **Extracted Data** |
| **4.1 Training Outcomes** |  |
| 4.1.1 Diagnostic Accuracy | NI |
| 4.1.2 Knowledge Acquisition | 1. Pre-test mean score: 52.8% (446 learners completed the pre-test). 2. Post-test mean score: 90.6% (432 learners completed the post-test). |
| 4.1.3 Skills Acquisition | 1. OSCE Results:    1. Number of learners completing OSCE: 489    2. Pass rate: 99% (486/489 learners achieved the pass mark of 13 out of 26).    3. Mean OSCE score: 87.3%.    4. Retake success rate: All learners who failed the first OSCE attempt passed on the second attempt. 2. Scans Completed During Training:    1. Minimum scans per learner: 20 scans for each of the five thematic areas (total: 100 scans per learner). |
| 4.1.4 Knowledge Retention | NI |
| 4.1.5 Practical Application | NI |
| **4.2 Maternal and Neonatal Health Outcomes** (Including Physiological and Psychological Outcomes) | NI |
| **4.3 Health Economic Outcomes** | NI |

**JBI Qualitative Data Extraction Tool**

**Reviewer: XXX & XXX & XXX Date: 28^th^ February 2025**

**Author: Ward et al. Year: 2024**

**Journal: Journal of the American College of Radiology Record Number: 10**

| **1. Study Characteristics** | |
| --- | --- |
| **Item** | **Extracted Data** |
| 1.1 Author (Publication Year) | Ward et al., 2024 |
| 1.2 Country | Nepal |
| 1.3 Location | Clinic in the Mahalaxmi municipality on the outskirts of Kathmandu; outreach clinic in a village located 2 hours east of Kathmandu |
| 1.4 Study Design | Quasi-experimental study |
| 1.5 Sample Size | NI |
|  | |
| **2. Population Characteristics (Healthcare Providers Receiving POCUS Training)** | |
| **Item** | **Extracted Data** |
| 2.1 Age | NI |
| 2.2 Gender | NI |
| 2.3 Professional Role | (1) Nurses  (2) Midwives |
| 2.4 Work Experience (Duration) | NI |
| 2.5 Prior Ultrasound Training Experience | NI |
|  | |
| **3. Intervention Characteristics (According to ADDIE Training Model)** | |
| **Item** | **Extracted Data** |
| 3.1 Analysis | 1. Trainee Group:   The trainees are healthcare providers (midwives and nurses) working in rural Nepal, where geographical isolation and resource scarcity limit access to advanced medical education.   1. Needs Analysis:   The training need arises because the target trainees lack the necessary skills to perform obstetric ultrasound, which is essential for detecting complications like placenta previa, fetal heart rate abnormalities, and polyhydramnios. The analysis indicates that the training focuses on basic obstetric ultrasound skills to help identify high-risk pregnancies and improve maternal and fetal outcomes in rural settings. The needs analysis considered the lack of ultrasound experience, existing clinical skills, and resource constraints in rural settings.   1. Baseline Survey: None. 2. Impact on Instructional Design:   The lack of prior ultrasound experience among the trainees and the identified need for obstetric ultrasound in rural areas directly influenced the training design. The curriculum was structured to teach basic ultrasound skills such as detecting placental abnormalities, abnormal fetal heart rates, and complications like polyhydramnios, with an emphasis on practical applications in the rural healthcare setting. |
| 3.2 Design | 1. Training Objectives:   Equip nurses and midwives with the knowledge and skills to identify high-risk pregnancies using ultrasound, while fostering a proactive approach to maternal care and timely referrals.   1. Training Methods:    1. Wave-Based Training: Initial training, a refresher course after six months, and advanced peer reviews to ensure continuous learning and skill development.    2. Blended Learning: A mix of hands-on ultrasound training and theoretical sessions, with support from online platforms (e.g., WhatsApp, Facebook) for ongoing learning.    3. Field-Based Learning: Training in real rural settings with actual patient cases for practical application and immediate feedback. 2. Training Content/Plan/Syllabus    1. Module 1 (Introductory): Basic ultrasound skills for detecting high-risk pregnancy markers, focusing on fundamental techniques and common complications (e.g., placenta previa).    2. Module 2 (Intermediate): Refresher course with real case studies to reinforce skills and address common challenges in rural practice.    3. Module 3 (Advanced): Peer review and case discussions for advanced nurses, focusing on complex cases and problem-solving. |
| 3.3 Development | 1. Instructors/Teachers    1. Identity/Qualifications: The trainers in this program include both physicians and experienced nurses. The physicians are typically specialists in radiology and obstetrics, while the nurses have expertise in maternal healthcare and bring practical knowledge to the training process.    2. Instructor-to-Trainee Ratio: The program uses a three-wave training model. During the first wave, a larger group of instructors teaches new nurses, though the exact ratio of instructors to trainees is not specified. The second wave involves fewer instructors providing refresher training, and the third wave involves experienced nurses mentoring their peers in ongoing peer review.    3. Instructor Training Program: The program ensures that instructors receive continuous training and updates to maintain their teaching skills. Instructors are also involved in peer learning through platforms like WhatsApp and Facebook, which help them stay engaged and share challenging cases with the wider community. 2. Teaching Aids    1. Electronic Platforms: While the article doesn’t mention specific electronic platforms, it highlights the use of WhatsApp and Facebook for ongoing peer learning. These platforms enable trainees and instructors to share complex cases and receive feedback in real time, providing a virtual classroom environment.    2. Devices: The primary teaching aid used in this training is the portable ultrasound device, specifically with curved probes, which are ideal for use in rural, low-resource settings due to their compact size and cost-effectiveness.    3. Models or Patients: Real patients are used as the key teaching tools for ultrasound training. This hands-on approach is vital for building practical scanning skills, as there are no mentions of using simulation models. 3. Teaching Materials    1. Lesson Plans, Lecture Notes, PPT: The training materials include lesson plans, lecture notes, and presentations (likely PowerPoint slides). These materials are developed with a focus on essential POCUS skills, such as recognizing abnormal amniotic fluid volumes, abnormal placenta positioning, and abnormal fetal heart rates.    2. Scales, Test Questions: While the article doesn’t mention specific scales or tests, it’s implied that some form of assessment, both theoretical and practical, is likely included in the program to measure trainees’ understanding and skills.    3. Development of Materials: The materials are developed iteratively, based on feedback from previous training sessions. Trainers adapt the content to meet the needs of local healthcare workers, with updates made as necessary to ensure the material remains relevant and practical for rural settings. |
| 3.4 Implementation | 1. Time:   The training occurs in three waves:   - 1. The first wave involves trainers (physicians and expert nurses) teaching a new group of nurses in a designated rural area.   2. The second wave takes place approximately six months later when the nurses return for a refresher course.   3. The third wave involves the selection of the most skilled nurses for ongoing peer review. The entire process spans over a year, with intermittent waves of training and refresher courses.  1. Location/Setting:   The training is conducted in rural villages in Nepal, including regions like Phaplu and Mahalaxmi municipality, which are remote and geospatially isolated. Due to resource constraints, healthcare providers in these regions rely heavily on portable ultrasound technology to detect high-risk pregnancies. Training takes place both on-site in these rural areas and occasionally at hospitals closer to urban centers like Kathmandu.   1. Participants:    1. Trainers: Physicians and expert nurses, including American radiology residents who contribute to the training of Nepalese nurses and midwives.    2. Trainees: Nurses and midwives from rural areas of Nepal, with ongoing peer review and additional training provided for the most skilled participants. Additionally, American radiology residents participate in the outreach clinics and gain hands-on experience. 2. Execution Process   The execution of the training and outreach program is structured in three waves:   - 1. In the first wave, nurses are trained in basic ultrasound techniques to identify high-risk pregnancies.   2. After six months, a refresher course is offered to reinforce knowledge and skills.   3. The third wave focuses on the ongoing development of the most skilled nurses, with opportunities for peer review and continued education via social media platforms like WhatsApp and Facebook. |
| 3.5 Evaluation | 1. Formative Evaluation (Process Evaluation)    1. Purpose: To assess the ongoing effectiveness of the training program for nurses and midwives in rural Nepal on using point-of-care ultrasound (POCUS) for identifying high-risk pregnancies. The focus is on ensuring the training process is working effectively and to identify areas for improvement.    2. Methods/Tools: Case discussions and peer reviews via social media platforms (WhatsApp, Facebook) for group feedback on challenging cases.    3. Timing: Occurs during the training process, including the initial training, refresher course after 6 months, and continuous peer reviews via social media. 2. Summative Evaluation    1. Purpose: To assess whether trained nurses and midwives are effectively applying their ultrasound skills in real-world clinical settings and whether high-risk pregnancies are correctly identified and referred for further care.    2. Methods/Tools: The evaluation is based on the practical application of ultrasound skills during mobile clinics. There is no explicit mention of structured assessment tools beyond observing the actual use of skills during these clinics.    3. Timing: Occurs after the initial and refresher training, during real-world mobile ultrasound clinics where trained nurses apply their skills. |
|  | |
| **4. Outcome Characteristics** | |
| **Item** | **Extracted Data** |
| **4.1 Training Outcomes** |  |
| 4.1.1 Diagnostic Accuracy | NI |
| 4.1.2 Knowledge Acquisition | NI |
| 4.1.3 Skills Acquisition | NI |
| 4.1.4 Knowledge Retention | NI |
| 4.1.5 Practical Application | NI |
| **4.2 Maternal and Neonatal Health Outcomes** (Including Physiological and Psychological Outcomes) | NI |
| **4.3 Health Economic Outcomes** | NI |

**JBI Qualitative Data Extraction Tool**

**Reviewer: XXX & XXX & XXX Date: 28^th^ February 2025**

**Author: Bentley et al. Year: 2015**

**Journal: Journal of Ultrasound in Medicine Record Number: 11**

| **1. Study Characteristics** | |
| --- | --- |
| **Item** | **Extracted Data** |
| 1.1 Author (Publication Year) | Bentley et al., 2015 |
| 1.2 Country | The Socialist People’s Libyan Arab Jamahiriya |
| 1.3 Location | The Obstetrics Labor and Delivery Outpatient Department at the John F. Kennedy Hospital, Monrovia |
| 1.4 Study Design | Cohort study |
| 1.5 Sample Size | 31 |
|  | |
| **2. Population Characteristics (Healthcare Providers Receiving POCUS Training)** | |
| **Item** | **Extracted Data** |
| 2.1 Age | NI |
| 2.2 Gender | NI |
| 2.3 Professional Role | Midwives: 100% (31/31) |
| 2.4 Work Experience (Duration) | NI |
| 2.5 Prior Ultrasound Training Experience | None (0/31) |
|  | |
| **3. Intervention Characteristics (According to ADDIE Training Model)** | |
| **Item** | **Extracted Data** |
| 3.1 Analysis | 1. Trainee Group:   The trainees are midwives working in Liberia, particularly in an urban hospital in Monrovia. These midwives are the primary providers of prenatal care in Liberia, and they have limited prior experience with ultrasound.   1. Needs Analysis:   The training need arises because the midwives lacked the skills to perform obstetric ultrasound, which is essential for detecting pregnancy complications such as ectopic pregnancies, placental abnormalities, and fetal heart rate issues. The training aims to equip midwives with the ability to use ultrasound for basic obstetric assessments, especially to help with early detection of high-risk pregnancies. The needs analysis considers the midwives’ baseline knowledge, lack of prior ultrasound experience, and the clinical environment where there is a shortage of trained sonographers and limited access to advanced diagnostic tools.   1. Baseline Survey:   The baseline survey included a pre-test and presurvey, assessing the midwives’ knowledge and comfort with ultrasound prior to the training. The results showed that most midwives had very low baseline knowledge and comfort in using ultrasound, with ultrasound use occurring infrequently, typically only once every 6 months.   1. Impact on Instructional Design:   The baseline data, indicating low prior knowledge and infrequent ultrasound use, directly influenced the training design. The curriculum was structured to be intensive, focusing on fundamental obstetric ultrasound skills for identifying complications that could impact patient care. The program was condensed into a 1-week course, divided into 4 modules, each combining didactic teaching and practical sessions. This approach was tailored to provide immediate knowledge improvement and to build long-term confidence in using ultrasound for obstetric care. |
| 3.2 Design | 1. Training Objectives:    1. Knowledge Goals: Improve midwives’ understanding of obstetric ultrasound, including fetal presentation, fetal heart rate, pregnancy dating, and placental location.    2. Skill Goals: Equip midwives with the skills to perform ultrasound scans, particularly for detecting complications in late-stage pregnancies, such as preterm labor, placenta previa, and fetal development issues.    3. Attitude Goals: Increase comfort and confidence in using ultrasound to make clinical decisions and incorporate it into routine prenatal care. 2. Training Methods:    1. Course Duration: 1-week intensive training course.    2. Course Structure: The curriculum consisted of 4 modules, each with a combination of didactic teaching, practical hands-on sessions, and supervised patient encounters. The midwives also underwent pre- and post-assessments to evaluate their knowledge and comfort with ultrasound.       1. Supervision: The course was taught by attending physicians and senior emergency medicine residents. The instructors were credentialed in emergency ultrasound and had additional training in late-trimester ultrasound.       2. Equipment: Portable SonoSite 180 ultrasound machines were used during the training, with an available transabdominal probe.       3. Assessment: Knowledge-based tests, surveys, and Objective Structured Clinical Examinations (OSCE) were conducted before, immediately after, and one year after the training. 3. Training Content/Plan/Syllabus:    1. Module 1: Ultrasound Basics/Normal Pregnancy   Introduction to ultrasound, machine orientation, patient positioning, creating images, and identifying normal early pregnancy features such as fetal heart tones.   - 1. Module 2: First-Trimester Pregnancy Complications   Focus on detecting complications such as missed abortion, threatened abortion, and ectopic pregnancy.   - 1. Module 3: Fetal Dating and Measurement   Introduction to measurements like crown-rump length, head circumference, and biparietal diameter to assess fetal growth.   - 1. Module 4: Second- and Third-Trimester Pregnancy.   Focus on complications in later stages, including preterm labor, placental issues (placenta previa, placental abruption), and cervical assessment. |
| 3.3 Development | 1. Instructors/Teachers:    1. Identity/Qualifications: The training was led by attending physicians and senior emergency medicine residents from the Icahn School of Medicine at Mount Sinai. All instructors were credentialed in emergency ultrasound and had additional training in late-trimester ultrasound, ensuring they were well-prepared to teach obstetric ultrasound.    2. Instructor-to-Trainee Ratio: The article mentions that 31 midwives underwent the training, with 17 midwives participating in the objective structured clinical examination (OSCE) and 8 of them retained for the 1-year follow-up. The exact instructor-to-trainee ratio isn’t specified, but given the small group size and intensive training, it can be inferred that the ratio was quite manageable.    3. Instructor Training Program: Instructors underwent preparation specific to the training curriculum, which included emergency ultrasound and late-trimester ultrasound. They provided direct supervision during patient encounters to ensure quality training and immediate feedback to trainees. 2. Teaching Aids:    1. Electronic Platforms: The article does not mention the use of electronic platforms for the training, but the curriculum includes hands-on practical sessions and supervised patient encounters, indicating a non-electronic, real-time, in-person approach to teaching.    2. Devices: The training utilized a portable SonoSite 180 ultrasound machine with a 2.5-MHz curvilinear transabdominal probe. This ultrasound device was crucial to the curriculum, as it was used both during the course and remained available for continued practice by the trainees before and after the course.    3. Models or Patients: The curriculum focused on real patient encounters. Midwives performed ultrasounds on third-trimester pregnant patients, assessing fetal presentation, fetal heart rate, number of fetuses, pregnancy dating, and placental location. This practical, patient-centered approach was a key component of the training. 3. Teaching Materials:    1. Lesson Plans, Lecture Notes, PPT: The curriculum was structured into 4 modules, each consisting of a didactic component, practical hands-on training, and supervised patient encounters. The modules covered a range of topics, including ultrasound basics, early pregnancy complications, fetal measurements, and second/third-trimester pregnancy issues.    2. Scales, Test Questions: The curriculum involved pre- and post-tests to assess knowledge retention and comfort levels. Additionally, participants were evaluated using an objective structured clinical examination (OSCE), which included a critical actions checklist. The OSCE was used to assess how well participants could perform ultrasound examinations on pregnant patients, testing their ability to identify critical obstetric indicators.    3. Development of Materials: The materials were adapted from Emergency Ultrasound, Second Edition, with the training curriculum customized to meet the specific needs of midwives in Liberia. The initial curriculum included a broader range of ultrasound applications, but certain topics (such as early pregnancy complications) were later dropped to better fit the late-pregnancy focus of the clinic where the midwives worked. |
| 3.4 Implementation | 1. Time:    1. Training Duration: The obstetric ultrasound curriculum is a 1-week course, which includes a didactic component, practical sessions, and supervised patient encounters.    2. Follow-Up: A follow-up is conducted 1 year after the training to assess knowledge retention and comfort levels. 2. Location/Setting:   The training takes place at the Obstetrics Labor and Delivery Outpatient Department of the John F. Kennedy Hospital in Monrovia, Liberia. This facility sees over 100 prenatal patients daily, with the majority of care provided by midwives.   1. Participants:    1. Trainers: The instructors are attending physicians and senior emergency medicine residents from the Icahn School of Medicine at Mount Sinai, who are credentialed in emergency ultrasound and have additional training in late-trimester ultrasound.    2. Trainees: 31 midwives from the John F. Kennedy Hospital participated in the training, with 14 midwives followed up for longitudinal assessment. 2. Execution Process:    1. The training began with theoretical lectures covering the basics of ultrasound, including machine operation and patient positioning. Midwives were then introduced to scanning normal early pregnancy.    2. Over the next days, the curriculum advanced to more complex topics, such as fetal dating, identifying first-trimester complications, and second- and third-trimester pregnancy complications like placenta previa and preterm labor.    3. Hands-on practice was integrated into the sessions, where midwives worked with real patients, performing ultrasound on third-trimester pregnant women. This focus on late pregnancy was influenced by the availability of patients at the clinic.    4. On the final day, midwives performed supervised scanning on patients and underwent an Objective Structured Clinical Examination (OSCE) to assess their ability to perform ultrasound for tasks such as identifying fetal presentation, measuring fetal heart rate, dating the pregnancy, and assessing placental location.    5. The OSCE and other assessments were conducted immediately after the training, and 1 year later, to track any changes or knowledge retention. |
| 3.5 Evaluation | 1. Formative Evaluation:    1. Purpose: To assess the effectiveness of the 1-week ultrasound curriculum for midwives in Liberia, with a focus on increasing their knowledge and comfort with using obstetric ultrasound.    2. Methods/Tools:       1. Pre- and posttests to assess knowledge improvement.       2. Pre- and post-surveys to evaluate comfort with ultrasound.       3. Objective Structured Clinical Examination (OSCE) to evaluate practical skills.    3. Timing: The formative evaluation occurs during the 1-week training course and is followed by posttests and OSCE immediately after the course and then again at 1-year follow-up. 2. Summative Evaluation:    1. Purpose: To assess the overall success of the training program, especially in terms of midwives’ long-term retention of knowledge and ability to apply ultrasound skills in practice.    2. Methods/Tools:       1. Knowledge-based posttests immediately after the course and again 1 year later.       2. OSCE scores assessed both immediately after the training and at 1-year follow-up.    3. Timing: Summative evaluation occurs at two points: immediately after the 1-week course and again at a 1-year follow-up. 3. Follow-up:    1. Purpose: To assess the long-term retention of ultrasound skills and the continued comfort of midwives using ultrasound after the training.    2. Methods/Tools:       1. Post-survey at 1-year follow-up to evaluate continued comfort and use of ultrasound.       2. OSCE repeated at 1-year follow-up to assess skill retention.    3. Timing: Follow-up occurs 1 year after the training, with follow-up surveys and OSCE assessments. |
|  | |
| **4. Outcome Characteristics** | |
| **Item** | **Extracted Data** |
| **4.1 Training Outcomes** |  |
| 4.1.1 Diagnostic Accuracy | NI |
| 4.1.2 Knowledge Acquisition | 1. Pretest vs. Immediate Posttest Scores:    1. Pretest: 36.6%    2. Immediate Posttest: 90%    3. 1-Year Posttest: 66%.    4. The difference between pretest and immediate posttest scores was statistically significant (*P* < 0.001), but the decline at the 1-year posttest was not statistically significant (*P* > 0.05). |
| 4.1.3 Skills Acquisition | 1. Comfort Level with Ultrasound:    1. Presurvey: Mean = 1.8.    2. Immediate Postsurvey: Mean = 3.8 (*P* < 0.001).    3. 1-Year Postsurvey: Mean = 3.4 (*P* < 0.05). 2. OSCE Performance:    1. Immediate OSCE: 78%    2. 1-Year OSCE: 55%    3. Statistically, the decline in OSCE scores was not significant (*P* > 0.05). |
| 4.1.4 Knowledge Retention | 1. Retention of Test Scores:    1. Knowledge Retention Rate (Pretest vs. 1-Year Posttest): Significant improvement from pretest to 1-year posttest (36.6% vs. 66%; *P* < 0.001).    2. No significant decline in knowledge from immediate posttest to 1-year posttest (90% vs. 66%; *P* > 0.05). 2. Retention of Specific OSCE Tasks:    1. Presentation: Immediate 94% (16/17), 1-Year 88% (7/8).    2. Parity: Immediate 100% (17/17), 1-Year 88% (7/8).    3. Placental Assessment: Immediate 94% (16/17), 1-Year 88% (7/8).    4. Fetal Heart Rate Measurement: Immediate 65% (11/17), 1-Year 38% (3/8).    5. Dating: Immediate 70% (12/17), 1-Year 38% (3/8). |
| 4.1.5 Practical Application | 1. Increased Ultrasound Usage:    1. Before Intervention: Mean = 1.68 on a 6-point Likert scale (“once every 6 months”).    2. 1-Year Follow-Up: Mean = 2.42 (P < 0.001), reflecting increased use of ultrasound in clinical practice. 2. Interest in Further Training: 90% (28/31) of participants expressed interest in additional ultrasound education. |
| **4.2 Maternal and Neonatal Health Outcomes** (Including Physiological and Psychological Outcomes) | NI |
| **4.3 Health Economic Outcomes** | NI |

**JBI Qualitative Data Extraction Tool**

**Reviewer: XXX & XXX & XXX Date: 28^th^ February 2025**

**Author:**  **Bidner et al. Year: 2022**

**Journal: BMC Medical Education Record Number: 12**

| **1. Study Characteristics** | |
| --- | --- |
| **Item** | **Extracted Data** |
| 1.1 Author (Publication Year) | Bidner et al., 2022 |
| 1.2 Country | Australia |
| 1.3 Location | The University of South Australia’s Adelaide city campus in a simulated ultrasound laboratory |
| 1.4 Study Design | Cohort study |
| 1.5 Sample Size | 41 |
|  | |
| **2. Population Characteristics (Healthcare Providers Receiving POCUS Training)** | |
| **Item** | **Extracted Data** |
| 2.1 Age | NI |
| 2.2 Gender | NI |
| 2.3 Professional Role | (1) Doctors: 39.0% (16/41)  (2) Nurses/Midwives: 61.0% (25/41) |
| 2.4 Work Experience (Duration) | 1. Doctors:    1. Range: 11–40 years    2. Mean (SD): 15.4 years (5.46) 2. Nurses/Midwives:    1. Range: 2–30 years    2. Mean (SD): 13.18 years (5.45) |
| 2.5 Prior Ultrasound Training Experience | 1. Overall: 65.9% (27/41) had prior ultrasound training experience 2. With Clinical Use Before Training:   43.9% (18/41) were performing ultrasound before the training (average 2 scans/week, range 1–5 scans/week).   1. Previous Formal Training:   53.6% (22/41) had attended prior ultrasound training.   1. 63.4% (26/41) had used ultrasound clinically before. 2. Special Cases:    1. 7.3% (3/41) were performing ultrasound without formal training.    2. 17.1% (7/41) had formal training but were not using ultrasound clinically. |
|  | |
| **3. Intervention Characteristics (According to ADDIE Training Model)** | |
| **Item** | **Extracted Data** |
| 3.1 Analysis | 1. Trainee Group:   The target trainees are healthcare professionals from rural and remote Australia, including general practitioners (GPs) and midwives/nurses (M/Ns). These clinicians are working in areas with limited access to ultrasound services and may have varied levels of prior ultrasound experience. The group is diverse, with some participants already possessing ultrasound skills, while others are new to the practice.   1. Needs Analysis:   The needs analysis identified that rural clinicians face significant barriers to accessing formal ultrasound training, including geographical isolation, heavy clinical workloads, and the lack of substitute staff to cover duties during training. This analysis was conducted through surveys, where the trainees’ reported challenges, such as travel distances for training, and the need for practical skills to perform antenatal point-of-care ultrasound (POCUS). The analysis highlighted the urgency of improving ultrasound competency in rural healthcare settings to ensure timely diagnoses and improve patient management.   1. Baseline Survey:   A pre-training survey was conducted to gather demographic information and assess the trainees’ prior experience with ultrasound, including their clinical roles and the frequency of ultrasound use in their practice. This survey revealed that nearly half of the participants were already performing ultrasound clinically, though many had minimal formal training. The survey also identified the common reasons for using ultrasound, such as estimating gestation, determining fetal presentation, and assessing fetal viability. Additionally, it was noted that most participants had patients who required significant travel for ultrasound services.   1. Impact on Instructional Design:   The baseline data directly influenced the design of the training program, which focused on hands-on experience with ultrasound equipment and practical skills in antenatal care. Given the rural setting, where access to trained sonographers is limited, the training emphasized POCUS as an accessible solution to meet the needs of these clinicians. The training was structured to accommodate both experienced and novice learners by providing a mix of theoretical content and practical sessions using high-fidelity simulators and live pregnant models. The content was designed to improve confidence in performing POCUS and its integration into clinical practice, with a focus on issues such as accurate gestational dating and identifying high-risk pregnancies. |
| 3.2 Design | 1. Training Objectives:    1. Knowledge Goals: Enhance knowledge in ultrasound principles, fetal biometry, obstetric anatomy, and pathology, along with image optimization techniques.    2. Skill Goals: Teach how to perform antenatal ultrasound, including fetal heart trace, crown-to-rump length measurement, and fetal presentation and placental position.    3. Attitude Goals: Improve confidence and comfort in using ultrasound in clinical practice, particularly in rural and remote settings, to support timely clinical decision-making. 2. Training Methods:    1. Course Duration: 2-day workshop, followed by a 1-day follow-up session after 12 months.    2. Course Structure:       1. Day 1: Didactic sessions (7 hours) combined with practical scanning sessions (6 hours).       2. Day 2: More practical training using live pregnant models and simulators.    3. Tools Used: High-fidelity simulators (Vimedix OB-GYN), live pregnant models (second and third trimester), and portable ultrasound equipment (SonoSite Edge-II, M-Turbo, Phillips iU22xMatrix).    4. Instructors: The instructors were accredited sonographers and specialists with experience in rural ultrasound training.    5. Assessment:       1. Pre- and Post-training Knowledge Tests: To measure improvement in theoretical understanding.       2. Objective Structured Clinical Exam (OSCE): To evaluate practical scanning competence, particularly in real clinical situations.       3. Follow-up: Follow-up surveys at 3 and 6 months to assess how the training translated into clinical practice. 3. Training Content/Plan/Syllabus:    1. Module 1: Basic Scanning Principles (Day 1)   Ultrasound physics, transducer manipulation, image optimization, ergonomics.   - 1. Module 2: First-Trimester Ultrasound (Day 1)   Gestational sac, fetal heart trace, crown-to-rump length measurement, early pregnancy complications like ectopic pregnancy.   - 1. Module 3: Second and Third-Trimester Ultrasound (Day 2)   Biometry (biparietal diameter, head circumference, abdominal circumference, femur length), placental position, and amniotic fluid volume.   - 1. Module 4: Clinical Applications and Advanced Scanning (Day 2)   Fetal viability assessment, multiple pregnancy, miscarriage, and patient communication. This module included hands-on training on live pregnant models. |
| 3.3 Development | 1. Instructors/Teachers:    1. Identity/Qualifications: The instructors in this program were accredited sonographers with 3 to 14 years of experience in medical sonography, as well as one instructor with specialist obstetrics and gynaecology medical training. These instructors had a background in delivering POCUS workshops in rural areas and developing countries.    2. Instructor-to-Trainee Ratio: The workshops were conducted in groups of 12–16 participants. During practical sessions, the faculty-to-trainee ratio ranged from 1:2 to 1:4, with occasional 1:1 supervision during hands-on training. This ensured personalized attention during skill practice.    3. Instructor Training Program: The instructors had extensive training and experience, both in ultrasound techniques and teaching. They were also involved in follow-up mentoring sessions after the workshops, providing remote guidance and ongoing support to trainees via online platforms. 2. Teaching Aids:    1. Electronic Platforms: Online mentoring and tele-ultrasound technologies were utilized, allowing trainees to connect with instructors remotely. The online platform allowed instructors to share content and control the ultrasound equipment, providing real-time feedback and troubleshooting.    2. Devices: The workshops used high-fidelity simulators (Vimedix OB-GYN) for initial learning of probe manipulation and ultrasound techniques. In addition, live pregnant models were used to practice real-world scanning techniques. For practical training, Sonosite Edge-II, Sonosite M-Turbo, and Phillips iU22xMatrix portable ultrasound devices were employed.    3. Models or Patients: Both simulated models and real pregnant models were used for hands-on practice. Simulators provided a safe and controlled environment for practicing scanning techniques, while live models helped trainees gain experience with real-world applications. The use of live models was limited to healthy, low-risk pregnancies, with each model being scanned for 30 minutes. 3. Teaching Materials:    1. Lesson Plans, Lecture Notes, PPT: The course design included didactic lectures and practical sessions, covering essential ultrasound principles, fetal biometry, obstetric anatomy, image optimization, and patient communication. Lecture sessions accounted for 7 hours, while practical sessions took up 6 hours of the training. Pre-reading materials were provided before the workshop to ensure trainees had background knowledge.    2. Scales, Test Questions: Pre- and post-training assessments were conducted to evaluate trainees’ knowledge of ultrasound principles, image optimization, and biometry measurements. The assessments included multiple-choice questions and practical evaluations to assess skills in patient communication and image review.    3. Development of Materials: The materials were developed by a multidisciplinary research team, with input from healthcare professionals. They were customized to suit the needs of rural clinicians and were aligned with ASUM guidelines for POCUS training. The training also involved competency assessments to evaluate the effectiveness of the training. |
| 3.4 Implementation | 1. Time:    1. Point of time: The workshops were held in 2018 and 2019.    2. Total duration: 2 days for each session.    3. Schedule:       1. Day 1: A full day of lectures on ultrasound principles, followed by hands-on practice with simulators.       2. Day 2: Focused on practical scanning, where participants worked on live pregnant models to apply techniques. 2. Location/Setting:    1. The training took place at the University of South Australia (UniSA), Adelaide, in a simulated ultrasound laboratory equipped with high-fidelity Vimedix OB-GYN simulators and live pregnant models.    2. The setting was designed to simulate a real clinical environment to help participants practice scanning techniques they would later use in rural clinics. 3. Participants:    1. Trainers: The instructors were experienced sonographers with specialized training in obstetrics and gynecology.    2. Trainees: The course included 41 clinicians (16 doctors and 25 midwives/nurses) from rural or remote healthcare settings, with varying levels of prior ultrasound experience. 4. Execution Process:    1. The workshop started with theoretical training on basic ultrasound concepts, focusing on how to operate the equipment, adjust settings, and identify basic fetal structures. This was followed by practical sessions where participants practiced using simulators and live models.    2. On the second day, the focus shifted to more advanced techniques, such as fetal biometry and placenta positioning, using the live models for hands-on practice.    3. While the training design initially aimed to cater to both early and late pregnancy ultrasound, in practice, the workshop focused heavily on second- and third-trimester scanning. This shift was due to the availability of live pregnant models in the later stages of pregnancy, which was not fully accounted for in the original design.    4. By the end of Day 2, participants were assessed through practical tests using both simulators and live models to ensure competency in key skills such as fetal presentation and placental location.    5. The workshop concluded with knowledge assessments and participant feedback to evaluate the immediate impact of the training, while a follow-up workshop was planned 12 months later to reinforce skills and provide further practice. |
| 3.5 Evaluation | 1. Formative Evaluation:    1. Purpose: To assess the immediate effectiveness of the 2-day antenatal POCUS workshop for rural and remote clinicians in improving their knowledge, confidence, and practical skills related to POCUS in antenatal care.    2. Methods/Tools:       1. Pre- and post-workshop knowledge assessments on ultrasound principles, obstetric anatomy, image optimization, biometry measurements, and patient communication.       2. Post-workshop evaluation surveys to assess participant satisfaction and perceived relevance of the content.       3. Objective Structured Clinical Examination (OSCE) to evaluate practical skills in performing antenatal ultrasound on live pregnant models and simulators.       4. The New World Kirkpatrick Evaluation Framework (NWKEF) was applied to assess the effectiveness of the training, focusing on the immediate reactions, learning, and the application of knowledge gained.    3. Timing: Formative evaluation occurs immediately after the 2-day workshop, including knowledge assessments and OSCE. 2. Summative Evaluation:    1. Purpose: To assess the long-term impact of the training on the clinicians’ ability to integrate POCUS into their clinical practice, and its effect on patient management and clinical outcomes.    2. Methods/Tools:       1. Follow-up surveys at 3 and 6 months to evaluate the application of POCUS in clinical practice, self-reported confidence, and changes in clinical behavior (e.g., frequency of scanning, diagnostic accuracy).       2. Follow-up OSCE at 12 months to assess retention of practical skills.       3. Data analysis: Paired t-tests were performed on pre- and post-test knowledge assessments, and follow-up surveys to compare changes over time.       4. Qualitative data from follow-up surveys were analyzed to evaluate the impact of the training on patient outcomes and clinical management.    3. Timing: Summative evaluation occurs at the 3-month and 6-month follow-ups, with a 12-month follow-up training and OSCE for skill retention. 3. Follow-up:    1. Purpose: To assess the retention of knowledge and skills gained from the training, and to evaluate the continued use of POCUS in clinical settings.    2. Methods/Tools:       1. Follow-up surveys at 3, 6, and 12 months to track the frequency of POCUS use, confidence, and perceived impact on patient outcomes.       2. Follow-up training session and OSCE at 12 months to evaluate knowledge retention and practical skills, with emphasis on more complex second-trimester scanning.       3. New World Kirkpatrick Evaluation Framework (NWKEF) was used to assess the longer-term impact of the training, evaluating the application of learning in clinical practice and the broader impact on patient care.    3. Timing: Follow-up takes place at 3, 6, and 12 months after the initial training, with follow-up surveys and the 12-month refresher training. |
|  | |
| **4. Outcome Characteristics** | |
| **Item** | **Extracted Data** |
| **4.1 Training Outcomes** |  |
| 4.1.1 Diagnostic Accuracy | NI |
| 4.1.2 Knowledge Acquisition | 1. Pre- and Post-Test Results (Initial Workshops):    1. Overall Improvement:       1. Mean pre-test score: 54.6% (SD 13.2).       2. Mean post-test score: 77.0% (SD 13.5).       3. Improvement: 22.4% (95% CI 17.1–27.8, P < 0.0001).    2. Stratified by Role:       1. Doctors (n = 16):          1. Pre-test: 62.5% (SD 8.2), Post-test: 82.2% (SD 12.4).          2. Improvement: 19.7% (95% CI 11.2–28.1, P = 0.00018).       2. Midwives/Nurses (n = 25):          1. Pre-test: 49.5% (SD 13.5), Post-test: 73.0% (SD 13.0).          2. Improvement: 23.5% (95% CI 15.6–30.6, P = 0.000052).    3. Stratified by Previous Training/Experience:       1. With Prior Training/Experience (n = 27):          1. Pre-test: 58.4% (SD 10.5), Post-test: 76.4% (SD 13.4).          2. Improvement: 18.3% (95% CI 13.2–23.5, P = 0.000065).       2. No Prior Training/Experience (n = 14):          1. Pre-test: 47.8% (SD 15.4), Post-test: 78.1% (SD 14.2).          2. Improvement: 30.4% (95% CI 18.0–42.3, P = 0.00014). 2. Follow-Up Workshop (Workshop 4):    1. Mean pre-test score: 80.6% (SD 12.2).    2. Mean post-test score: 89.8% (SD 13.5).    3. Improvement: 9.2% (95% CI 3.1–15.0, P = 0.008). 3. Key Observations:    1. Significant improvement was observed in pre- and post-test knowledge scores across all groups, with the greatest improvement seen in participants with no prior ultrasound experience (30.4%).    2. General Practitioners had higher pre- and post-test scores compared to Midwives/Nurses, but the latter demonstrated greater relative improvement. |
| 4.1.3 Skills Acquisition | 1. Scanning Frequency: 30 trainees (74%, 30/41) reported an increase in ultrasound scanning frequency post-training. 2. Scanning Confidence: 38 trainees (93%, 38/41) reported improved confidence in performing ultrasound scans. |
| 4.1.4 Knowledge Retention | (1) Pre- and Post-Test Results (Follow-Up Workshop):   - 1. Pre-test score: 80.6% (SD 12.2).   2. Post-test score: 89.8% (SD 13.5).   3. Improvement: 9.2% (95% CI 3.1–15.0, P = 0.008).   4. Results indicate knowledge retention and enhanced understanding of ultrasound concepts following clinical scanning experience.   (2) Self-Reported Needs for Retention:   - 1. 78% (28/36) of trainees named follow-up training and practice as necessary to consolidate skills, improve confidence, and further increase the frequency of POCUS use. |
| 4.1.5 Practical Application | (1) Knowledge Application:   - 1. At 6 months post-training, 87% (34/39) of trainees reported applying knowledge gained from the training in clinical practice.   (2) Scanning Frequency and Barriers:   - 1. At 3 months, 11% (4/38) of trainees were not performing POCUS, decreasing to 5% (2/38) at 6 months.   2. Limited POCUS use was mainly due to broken or sub-optimal equipment, lack of antenatal patients, and non-clinical work roles.   (3) Confidence and Practice Improvements:   - 1. 69% (27/39) of trainees reported increased confidence in performing antenatal POCUS.   2. Improved skills were observed in basic scanning techniques (97%, 39/39), fetal heart/M-mode scanning (76%, 30/39), and fetal lie assessment (75%, 29/39). |
| **4.2 Maternal and Neonatal Health Outcomes** (Including Physiological and Psychological Outcomes) | (1) Impact on Patient Management:   - 1. 74% (28/39) of trainees reported that POCUS had an impact on patient outcomes, as perceived by the clinician, demonstrating the practical benefits of the training.   2. Trainees noted improvements in care planning, patient engagement, and antenatal care compliance.   (2) Reduced Travel:   - 1. Many instances were described where patients no longer needed to travel for early dating scans, which had previously been required to plan antenatal care and schedule formal ultrasound imaging.   (3) Early Diagnosis and Better Care:   - 1. POCUS facilitated early diagnosis and more accurate estimation of gestation, which is crucial for delivery planning, particularly for women located in remote areas. |
| **4.3 Health Economic Outcomes** | The early detection of problems and reduction of unnecessary travel/transfers can also provide direct economic benefits to healthcare systems. |

**JBI Qualitative Data Extraction Tool**

**Reviewer: XXX & XXX & XXX Date: 28^th^ February 2025**

**Author: Erlick et al. Year: 2023**

**Journal: Ultrasound Quarterly Record Number: 13**

| **1. Study Characteristics** | |
| --- | --- |
| **Item** | **Extracted Data** |
| 1.1 Author (Publication Year) | Erlick et al., 2023 |
| 1.2 Country | The United States of America |
| 1.3 Location | A single tertiary care center |
| 1.4 Study Design | Cohort study |
| 1.5 Sample Size | 6 |
|  | |
| **2. Population Characteristics (Healthcare Providers Receiving POCUS Training)** | |
| **Item** | **Extracted Data** |
| 2.1 Age | NI |
| 2.2 Gender | NI |
| 2.3 Professional Role | Students: 100% (6/6) |
| 2.4 Work Experience (Duration) | Students: None (0/6) |
| 2.5 Prior Ultrasound Training Experience | None (0/6) |
|  | |
| **3. Intervention Characteristics (According to ADDIE Training Model)** | |
| **Item** | **Extracted Data** |
| 3.1 Analysis | 1. Trainee Group:   The trainees are medical students in their first to fourth years, with no prior ultrasound experience. They were selected to evaluate the feasibility of training non-experts in performing Obstetric VSI (Volume Sweep Imaging) in a brief, condensed training session.   1. Needs Analysis:   The training need stems from the fact that the trainees had no prior experience with ultrasound. The training aimed to teach them OB VSI, a simplified ultrasound protocol that doesn’t require prior knowledge of anatomy. The training focused on basic obstetric ultrasound, such as assessing fetal heart rate, placental location, fetal presentation, and amniotic fluid volume. The needs analysis considered the trainees’ lack of ultrasound experience, their educational background, and the environment in which this training could be applied, specifically in low-resource settings.   1. Baseline Survey: None. 2. Impact on Instructional Design:   The lack of ultrasound experience and the trainees’ low comfort levels with ultrasound influenced the design of the training program. It was structured to teach the OB VSI protocol, which is a simple and quick method of performing ultrasound. The focus was on enabling trainees to perform basic obstetric scans efficiently, using an intensive 3-hour session with didactic teaching and hands-on practice. |
| 3.2 Design | 1. Training Objectives:    1. Knowledge Goals: Improve knowledge of obstetric ultrasound principles, including the use of external landmarks, fetal presentation, placental location, and other key features relevant to second and third trimester obstetric care.    2. Skill Goals: Enable trainees to perform obstetric volume sweep imaging (OB VSI), focusing on external body landmarks for obtaining quality images and understanding basic ultrasound techniques.    3. Attitude Goals: Build confidence in using ultrasound to assist in pregnancy assessment, especially in low-resource settings, while emphasizing the importance of accurate scanning and protocol adherence. 2. Training Methods:    1. Course Duration: 3 hours (one-time session).    2. Course Structure:       1. The course included a didactic portion (60 minutes) covering the OB VSI protocol and basic ultrasound principles.       2. Hands-on Practice (120 minutes) involved using ultrasound equipment to perform OB VSI on live pregnant models and simulators.    3. Facilitators: The training was led by a board-certified maternal-fetal medicine specialist and a postdoctoral clinical fellow with expertise in obstetrics and ultrasound.    4. Equipment: The training used ultrasound systems, including the Butterfly IQ system with iPad and standard high-end ultrasound machines.    5. Assessment:       1. Primary Outcome: Frequency of protocol deviations during the OB VSI scans, measured and reviewed by maternal-fetal medicine specialists.       2. Secondary Outcomes: Evaluation of image quality and examination quality using Likert scales (excellent, acceptable, poor).       3. Trainees performed OB VSI independently after the training, with their scans reviewed for protocol adherence and diagnostic quality. 3. Training Content/Plan/Syllabus:    1. Module 1: Basic Ultrasound Principles   Introduction to the OB VSI protocol, transducer orientation, and understanding of anatomy landmarks.   - 1. Module 2: OB VSI Protocol   Focused on performing the 8-step protocol, including positioning the transducer and obtaining full volumetric acquisitions of the target region.   - 1. Module 3: Clinical Practice   Practical session with live pregnant models and simulators to apply the OB VSI protocol in real-world conditions. |
| 3.3 Development | 1. Instructors/Teachers:    1. Identity/Qualifications: The training facilitators in this study were experienced healthcare professionals with specialized knowledge in ultrasound. Specifically, the training was led by a board-certified maternal-fetal medicine specialist with 15 years of experience in obstetric ultrasound and a postdoctoral clinical fellow with expertise in obstetrics and gynecology.    2. Instructor-to-Trainee Ratio: The training was delivered one-on-one, where each trainee received individualized instruction. This format allowed for highly focused attention on each participant’s progress.    3. Instructor Training Program: The instructors themselves were well-prepared, with experience in both performing ultrasound and teaching. Although they did not require proficiency in interpreting ultrasound images, they were skilled in using ultrasound equipment for obstetric applications. This ensured that the focus remained on the practical, procedural aspects of scanning. 2. Teaching Aids:    1. Electronic Platforms: While the study does not specifically mention the use of an electronic platform during the training itself, it suggests that the images obtained during the scans were uploaded for telemedicine-based review. This suggests that the use of remote image interpretation played a role in the training.    2. Devices: The training utilized the Butterfly IQ ultrasound system, a portable device designed for point-of-care use, paired with an Apple iPad. This choice of equipment emphasizes the program’s goal of making ultrasound more accessible in low-resource settings. The device’s portability was key for training in environments where traditional ultrasound equipment might not be available.    3. Models or Patients: The training involved scanning real pregnant patients, specifically those with pregnancies greater than 14 weeks. The hands-on scanning was performed on patients in a tertiary care setting, which helped ensure the trainees were exposed to real-world clinical scenarios. 3. Teaching Materials:    1. Lesson Plans, Lecture Notes, PPT: The curriculum was designed to include both didactic learning and hands-on clinical practice. The didactic portion covered the 8-step OB VSI protocol, which guides the operator through a series of external body landmark-based movements to capture the necessary images. Trainees also had access to a training video demonstrating the protocol.    2. Scales, Test Questions: Although the study did not specify the use of traditional written tests, the primary assessment involved protocol deviations in the trainees’ performance during their hands-on scanning. These errors were analyzed to assess the success of the training program.    3. Development of Materials: The training materials were developed by the research team to ensure they were appropriate for non-medically trained individuals. The materials focused on simplicity and were designed to be easily followed without prior ultrasound experience. The 8-step OB VSI protocol served as the core teaching framework, which was supported by visual aids, a facilitator guide, and video demonstrations. |
| 3.4 Implementation | 1. Time:    1. Point of time: The training was conducted from July 2019 to July 2021.    2. Total duration: The training program lasted for 3 hours per individual.    3. Schedule:       1. 60 minutes of didactic training on the OB VSI protocol, covering ultrasound principles and the steps involved in the volume sweep imaging (VSI) technique.       2. 120 minutes of hands-on clinical scanning, where participants practiced using the ultrasound machine to perform the OB VSI protocol. 2. Location/Setting:    1. The training was conducted at the Obstetrics and Gynecology Ultrasound Unit in a tertiary care academic medical center.    2. Setting: The didactic portion took place in a clinical office building equipped with workstations for theoretical content, and the hands-on portion occurred in clinical examination rooms where participants performed scans on real obstetric patients. 3. Participants:    1. Trainers: The training was facilitated by a maternal-fetal medicine specialist with 15 years of experience in obstetric ultrasound and a clinical fellow in obstetrics and gynecology. Both trainers had expertise in performing ultrasound and teaching hands-on scanning.    2. Trainees: The program was aimed at ultrasound-naive individuals with no prior experience. In this study, the participants were medical students (first to fourth year) without prior ultrasound experience. 4. Execution Process:    1. The training began with a didactic session covering the OB VSI protocol—a simple ultrasound technique that requires minimal prior knowledge of anatomy. The protocol involves a series of eight steps, including transducer placement and movement guided by external body landmarks, and takes about 10 minutes to perform.    2. Following the didactic session, participants moved on to clinical scanning practice in a clinical setting, where they performed the OB VSI protocol on real patients. The protocol involved sweeping the ultrasound transducer across the abdomen to capture a full volumetric image of the target region.    3. Trainees were assessed on their ability to follow the OB VSI protocol during scanning, with particular attention to correct transducer orientation and appropriate sweep duration. Errors such as marker orientation and sweep timing were identified as common deviations.    4. After completing the training, trainees independently performed OB VSI scans on patients with known prenatal abnormalities. These scans were reviewed by specialists to evaluate the quality and diagnostic potential of the images. The focus was on ensuring image quality and ensuring that the scan captured key variables such as fetal heart motion, fetal presentation, and placental position. |
| 3.5 Evaluation | 1. Formative Evaluation:    1. Purpose: To evaluate the immediate effectiveness of a brief, standardized obstetric ultrasound training program designed for individuals with no prior ultrasound experience. The focus is on assessing the trainees’ ability to perform the Obstetric Volume Sweep Imaging (OB VSI) protocol after a brief training session.    2. Methods/Tools:       1. Knowledge-based pre- and post-assessments to evaluate the increase in theoretical knowledge related to obstetric ultrasound.       2. Practical training involving hands-on practice with live models and simulators to demonstrate the OB VSI protocol.       3. Protocol deviation tracking: The scans performed by trainees were reviewed for protocol deviations (e.g., errors in transducer placement, sweep speed, or marker orientation).    3. Timing: The formative evaluation occurs immediately after the 3-hour training session, focusing on immediate knowledge gain and practical skill development. 2. Summative Evaluation:    1. Purpose: To assess the long-term effectiveness and real-world application of the training, focusing on the ability of trainees to perform OB VSI scans accurately and the diagnostic value of their images.    2. Methods/Tools:       1. Follow-up assessments: Trainees performed OB VSI scans on pregnant patients, which were then reviewed for protocol deviations and image quality by maternal-fetal medicine specialists.       2. Error rates and image quality assessment: The frequency of protocol deviations and the quality of images (rated on a Likert scale: excellent, acceptable, or poor) were assessed.       3. Diagnostic potential evaluation: Determining whether protocol deviations impacted the diagnostic quality of scans, particularly in terms of fetal heart motion, fetal presentation, placental location, and amniotic fluid volume.    3. Timing: Summative evaluation is performed after trainees have conducted the OB VSI scans independently, with continuous assessment over the study period as trainees progress through their scans. 3. Follow-up:    1. Purpose: To evaluate the retention of skills and the continued application of OB VSI after the initial training session, and to monitor the progress in reducing protocol deviations over time.    2. Methods/Tools:       1. Longitudinal tracking of protocol deviation rates over time to assess how the accuracy of the scans improves as trainees gain experience.       2. Follow-up image quality assessment: Periodic review of the scans to evaluate whether the trainees’ images maintain diagnostic quality and whether protocol adherence improves.       3. Retention of knowledge: Analysis of protocol deviations over time and how these impact the ability to diagnose key obstetric conditions.    3. Timing: Follow-up is conducted after trainees have performed scans independently, with assessment of protocol adherence and image quality over several months. |
|  | |
| **4. Outcome Characteristics** | |
| **Item** | **Extracted Data** |
| **4.1 Training Outcomes** |  |
| 4.1.1 Diagnostic Accuracy | 1. Protocol Deviation Rate:    1. Total scans with deviations: 25.8% (50/194)    2. Deviations affecting diagnostic potential: 7.7% (15/194) 2. Image Quality:    1. Rated “excellent” or “acceptable”: 88.2% (171/194)    2. Rated “poor”: 11.9% (23/194) 3. Examination Quality:    1. Diagnostic or limited: 96.4% (187/194)    2. Non-diagnostic: 3.6% (7/194) |
| 4.1.2 Knowledge Acquisition | NI |
| 4.1.3 Skills Acquisition | NI |
| 4.1.4 Knowledge Retention | 1. Retention of Training Program:   The frequency of protocol deviations decreased over time for the majority of trainees, indicating retention of the training program.   1. Statistically Significant Retention:    1. Trainee 1: Significant decrease in protocol deviations (slope, −0.12; R² = 0.56; P < 0.01).    2. Trainee 5: Significant decrease in protocol deviations (slope, −0.80; R² = 0.98; P < 0.01).    3. Trainee 6: Significant increase in protocol deviations (slope, 0.03; R² = 0.63; P < 0.01).    4. Other trainees showed no significant relationship between protocol deviations and time. |
| 4.1.5 Practical Application | NI |
| **4.2 Maternal and Neonatal Health Outcomes** (Including Physiological and Psychological Outcomes) | NI |
| **4.3 Health Economic Outcomes** | NI |

**JBI Qualitative Data Extraction Tool**

**Reviewer: XXX & XXX & XXX Date: 28^th^ February 2025**

**Author: Greenwold et al. Year: 2014**

**Journal: International Journal of Gynecology and Obstetrics Record Number: 14**

| **1. Study Characteristics** | |
| --- | --- |
| **Item** | **Extracted Data** |
| 1.1 Author (Publication Year) | Greenwold et al., 2014 |
| 1.2 Country | the Republic of Mozambique |
| 1.3 Location | (1) Lead Site:  Mandimba Health Clinic  (2) Pilot Sites:  Rural health centers in Lissiete, Lussungasse, and Ntembo |
| 1.4 Study Design | Cohort study |
| 1.5 Sample Size | 10 |
|  | |
| **2. Population Characteristics (Healthcare Providers Receiving POCUS Training)** | |
| **Item** | **Extracted Data** |
| 2.1 Age | NI |
| 2.2 Gender | NI |
| 2.3 Professional Role | Nurses and clinical officers: 100% (9/9) |
| 2.4 Work Experience (Duration) | NI |
| 2.5 Prior Ultrasound Training Experience | Nurses and clinical officers: None (0/9) |
|  | |
| **3. Intervention Characteristics (According to ADDIE Training Model)** | |
| **Item** | **Extracted Data** |
| 3.1 Analysis | 1. Trainee Group:   The target trainees in this study were healthcare workers from rural Mozambique, including 9 nurses and clinical officers with no prior exposure to ultrasound imaging. A doctor also observed the training. These trainees were selected due to their positions in rural health centers where there was no prior access to ultrasound services. Following the training, four trainees demonstrated a special interest in ultrasound and were chosen as the local ultrasound coordinators for specific sites.   1. Needs Analysis:   The training need arises because the trainees lacked the skills to perform basic obstetric ultrasound. The training focused on teaching them how to use ultrasound to detect high-risk pregnancy conditions like placenta previa, multiple pregnancies, and abnormal fetal positions. The needs analysis considered the trainees’ lack of prior ultrasound experience, their clinical roles, and the rural setting where ultrasound was previously unavailable. The goal was to equip the trainees with the basic skills required for prenatal screening and to help them improve maternal and fetal outcomes in this low-resource setting.   1. Baseline Survey: None. 2. Impact on Instructional Design:   The lack of prior ultrasound experience among the trainees influenced the design of the training program. The curriculum was designed to teach basic obstetric ultrasound skills over an 8-week period, including hands-on training with the use of portable ultrasound devices. The content prioritized detecting high-risk pregnancies, such as identifying abnormal fetal positions and placental abnormalities. The training program was practical, incorporating real patient scans, and included remote support after the initial training to ensure continuity and sustainability of the skills. |
| 3.2 Design | 1. Training Objectives:    1. Knowledge Goals: Provide healthcare workers in rural Mozambique with the foundational knowledge required to perform basic obstetric ultrasound, including detecting pregnancy complications such as breech presentations, placenta previa, and multiple pregnancies.    2. Skill Goals: Enable trainees to operate portable ultrasound equipment and perform basic obstetric ultrasound scans to assess fetal position, number of gestations, and placental location.    3. Attitude Goals: Foster confidence in rural healthcare workers to use ultrasound for obstetric screening, improving prenatal care in low-resource settings. 2. Training Methods:    1. Course Duration: 8 weeks total       1. First Week: Theoretical lectures and supporting videos.       2. Following 7 Weeks: Hands-on, practical training on ultrasound devices with supervised scanning.    2. Delivery Mode:       1. In-person training with hands-on sessions at health clinics and rural health centers.       2. Training Support: Use of training videos and Android tablets for remote data collection and image transfer.    3. Equipment:       1. Portable ultrasound devices: M-Turbo (Sonosite) ultrasound machines and high-resolution portable units.       2. Mobile tablets (Android) for data collection and remote image transfer via the EpiCollect web application.    4. Instructors:       1. UK-based certified sonographers with experience in obstetrics and ultrasound education.       2. The trainers were experts in ultrasound techniques and had a background in teaching healthcare workers in low-resource settings. 3. Training Content/Plan/Syllabus:    1. Module 1: Basic Ultrasound Skills (First Week)       1. Introduction to ultrasound equipment and basic scanning principles.       2. Learning fetal anatomy and identifying key features such as gestational sac, crown-to-rump length, and early pregnancy markers.    2. Module 2: First Trimester Ultrasound (Weeks 2-8)   Estimation of gestational age, fetal viability, and identifying common early pregnancy complications.   - 1. Module 3: Second and Third Trimester Ultrasound (Weeks 2-8)   Focus on fetal positioning, detecting breech and transverse presentations, and assessing placental location.   - 1. Module 4: Practical Application (Weeks 2-8)   Hands-on scanning with direct supervision, followed by independent scanning with remote support. |
| 3.3 Development | 1. Instructors/Teachers:    1. Identity/Qualifications: The training was conducted by UK-based certified sonographers. The instructors had specialized expertise in obstetric ultrasound and were able to train local health workers effectively. They spent two-week stints at the Mandimba Health Clinic in Mozambique, providing both direct instruction and support to the trainees.    2. Instructor-to-Trainee Ratio: The program trained 9 local nurses and clinical officers with no prior exposure to ultrasound. The training was delivered in small groups, with the instructors providing hands-on supervision during the scanning sessions. The overall instructor-to-trainee ratio was not explicitly stated, but the direct supervision indicates a manageable ratio.    3. Instructor Training Program: The instructors were experienced in both performing and teaching ultrasound. They provided an 8-week curriculum consisting of 1 week of formal lectures and 7 weeks of practical hands-on training in using ultrasound machines, covering first-trimester ultrasound, gestational age estimation, and evaluation of fetal and placental positions. 2. Teaching Aids:    1. Electronic Platforms: The study utilized Android tablets and the EpiCollect web application for data collection, enabling easy management and transfer of patient information and ultrasound images. These devices were used for image transfer and for remote support of the trainees.    2. Devices: The ultrasound machines used in the training were Sonosite M-Turbo portable devices. These machines were equipped with integrated obstetric biometric charts, making them ideal for use in rural, low-resource settings. Their portability allowed for effective use in both the main clinic and remote health centers.    3. Models or Patients: The training involved real pregnant patients who attended the prenatal clinics at the Mandimba Health Clinic and rural health centers. The trainees performed ultrasound scans on pregnant women to assess key aspects such as fetal position, placental location, and the presence of multiple pregnancies or fetal anomalies. 3. Teaching Materials:    1. Lesson Plans, Lecture Notes, PPT: The training program included didactic lessons supported by training videos, which helped to reinforce the theoretical concepts. The curriculum was designed to be comprehensive yet simple, focusing on the basics of obstetric ultrasound.    2. Scales, Test Questions: The primary assessment for the trainees was through supervised scanning, where their ability to perform the ultrasound protocol was directly evaluated. While the article did not mention formal written tests, the trainees were evaluated on their practical skills in real-world settings.    3. Development of Materials: The training materials were designed to be accessible for healthcare workers in rural areas without prior ultrasound experience. The use of training videos translated into local languages provided an ongoing educational resource. Additionally, the data collection software installed on tablets allowed for real-time feedback and remote evaluation by the trainers, ensuring continuous learning and quality control. |
| 3.4 Implementation | 1. Time:    1. Point of time: The training took place over 8 weeks from August 2011 to July 2012.    2. Total duration: The training consisted of 1 week of theoretical training, followed by 7 weeks of practical hands-on training.    3. Schedule:       1. Week 1: The first week was dedicated to theoretical lectures on ultrasound basics, fetal development, and maternal health risks.       2. Week 2-8: The trainees participated in practical scanning sessions at Mandimba Health Clinic and various rural health centers. The sessions involved direct supervised scanning of pregnant women, with hands-on use of the M-Turbo portable ultrasound machine. 2. Location/Setting:    1. The training was conducted in Mandimba District, located in northwestern Mozambique, where healthcare infrastructure is limited.    2. Training setting: Practical training sessions were held at the Mandimba Health Clinic and rural health centers in nearby towns like Lissiete, Lussungasse, and Ntembo.    3. Equipment: The training utilized M-Turbo portable ultrasound machines equipped with obstetric biometric charts, facilitating scans for gestational age estimation, fetal position, twin detection, and placenta localization. 3. Participants:    1. Trainers: The program was led by UK-based certified sonographers who traveled in pairs to Mandimba for 2-week intervals to provide the hands-on training and supervision.    2. Trainees: The trainees included 9 nurses and clinical officers with no previous ultrasound experience. One doctor attended as an observer. After training, 4 trainees (3 nurses and 1 clinical officer) showed special aptitude for ultrasound and were appointed as local ultrasound coordinators for future sessions. 4. Execution Process:    1. The training commenced with 1 week of theoretical lectures, where trainees learned to operate the ultrasound machine and the basic principles of obstetric ultrasound.    2. From Week 2 to Week 8, the trainees were involved in practical scanning of pregnant women at local health centers. They learned to use the ultrasound to detect fetal malposition, multiple pregnancies, placenta previa, and fibroids.    3. Supervised scanning was initially done by trainers to ensure proper technique, and as trainees gained confidence, they performed the scans independently.    4. Throughout the practical training, the trainees performed routine obstetric ultrasound exams on pregnant women, with real-time feedback from trainers. The focus was on accurately identifying key obstetric conditions that could affect delivery, such as breech presentation, multiple pregnancies, and placenta previa.    5. After the initial 8 weeks, the trainees continued practicing independently, with remote support from the trainers through mobile phones for data transfer and feedback. This ongoing support was facilitated by mobile technology to ensure quality control and assist with any difficulties the trainees encountered. |
| 3.5 Evaluation | 1. Formative Evaluation:    1. Purpose: To evaluate how well the trainees are acquiring the skills to perform basic obstetric ultrasound, focusing on the immediate outcomes of the training.    2. Methods/Tools:       1. Protocol deviation tracking: The scans performed by trainees were reviewed for protocol deviations (such as incorrect probe orientation or sweep errors).       2. Assessment of image quality: The quality of the ultrasound images (rated as excellent, acceptable, or poor) was assessed.    3. Timing: Formative evaluation occurred during the 8-week training period, with immediate feedback on the scans performed by the trainees. 2. Summative Evaluation:    1. Purpose: To assess whether the trainees can independently perform accurate obstetric ultrasound scans and whether these scans provide clinically useful information.    2. Methods/Tools:       1. Protocol adherence: Evaluation of how well trainees follow the established scanning protocol and whether deviations impact diagnostic accuracy.       2. Diagnostic quality of images: Images from scans performed by trainees were evaluated for their ability to identify key obstetric features (e.g., fetal heart motion, presentation, placental location).    3. Timing: Summative evaluation occurred after the completion of the 8-week training, with ongoing assessments during the scans performed independently by the trainees. 3. Follow-up:    1. Purpose: To track the retention of skills and the continued use of ultrasound in clinical practice, assessing whether trainees are applying their skills effectively over time.    2. Methods/Tools:       1. Follow-up ultrasound scans: Trainees performed scans independently, and these were assessed for adherence to the protocol and diagnostic quality.       2. Ongoing remote support: Use of mobile technology for remote evaluation of scans, enabling feedback and continued learning.    3. Timing: Follow-up evaluation occurred 4 months after the training, with remote support continuing for a period afterward. |
|  | |
| **4. Outcome Characteristics** | |
| **Item** | **Extracted Data** |
| **4.1 Training Outcomes** |  |
| 4.1.1 Diagnostic Accuracy | 1. Detection rates for major obstetric risks:    1. Twin pregnancies: 2.6% (21/804) supervised vs. 1.6% (15/930) unsupervised.    2. Placenta previa: 0.7% (6/804) supervised vs. 1.7% (16/930) unsupervised.    3. Breech presentation: 14.7% (118/804) supervised vs. 12.0% (112/930) unsupervised.    4. Fetal anomalies: 2.6% (21/804) supervised vs. 0.2% (2/930) unsupervised (P < 0.001). |
| 4.1.2 Knowledge Acquisition | NI |
| 4.1.3 Skills Acquisition | 1. Trainees effectively acquired ultrasound skills:    1. A total of 1744 scans were performed, with 53.9% (940/1744) conducted independently.    2. Detection rates for most complications (e.g., breech, placenta previa) were similar between supervised and unsupervised groups. |
| 4.1.4 Knowledge Retention | NI |
| 4.1.5 Practical Application | The 4 trainees who were selected at the end of the training phase have not only completed their task of scanning and collecting data to the end of the project but also continued to provide a similar ultrasound service to the local population of pregnant women after the end of the project. |
| **4.2 Maternal and Neonatal Health Outcomes** (Including Physiological and Psychological Outcomes) | 1. Identification of Obstetric and Neonatal Risks: Ultrasound identified several key clinical features among 1734 cases:    1. Breech presentations: 13.3% (230/1734).    2. Transverse presentations: 4.8% (83/1734).    3. Twin pregnancies: 2.1% (36/1734).    4. Placenta previa: 1.3% (22/1734).    5. Fetal structural defects: 1.3% (23/1734).    6. Second-trimester pregnancy loss or intrauterine fetal death: 0.5% (9/1734).    7. Large fibroids resulting in unstable fetal lie: 0.2% (4/1734). 2. Follow-Up and Referrals:    1. 13.3% (230/1734) women received follow-up scans.    2. 3.6% (42/1734) women were referred to a doctor, with 21 requiring cesarean delivery. 3. Improved Delivery Outcomes: Ultrasound screening helped healthcare workers manage high-risk pregnancies, improving maternal and neonatal outcomes. 4. Increased Prenatal Clinic Attendance: The use of ultrasound imaging attracted more mothers to prenatal clinics, improving access to antenatal care. |
| **4.3 Health Economic Outcomes** | NI |

**JBI Qualitative Data Extraction Tool**

**Reviewer: XXX & XXX & XXX Date: 28^th^ February 2025**

**Author: Henwood et al. Year: 2017**

**Journal: Journal of Ultrasound in Medicine Record Number: 15**

| **1. Study Characteristics** | |
| --- | --- |
| **Item** | **Extracted Data** |
| 1.1 Author (Publication Year) | Henwood et al., 2017 |
| 1.2 Country | the Republic of Rwanda |
| 1.3 Location | Rwandan hospitals |
| 1.4 Study Design | Cohort study |
| 1.5 Sample Size | 17 (15 submitted recorded studies) |
|  | |
| **2. Population Characteristics (Healthcare Providers Receiving POCUS Training)** | |
| **Item** | **Extracted Data** |
| 2.1 Age | NI |
| 2.2 Gender | NI |
| 2.3 Professional Role | Doctors: 100% (17/17) |
| 2.4 Work Experience (Duration) | NI |
| 2.5 Prior Ultrasound Training Experience | NI |
|  | |
| **3. Intervention Characteristics (According to ADDIE Training Model)** | |
| **Item** | **Extracted Data** |
| 3.1 Analysis | 1. Trainee Group:   The target trainees in this study were physicians from various hospitals in Rwanda, including district hospitals. These participants had no prior point-of-care ultrasound experience. Seventeen physicians started the program, with 15 continuing to participate throughout the study. The group was selected in conjunction with the Rwandan Ministry of Health, and the program targeted physicians practicing in resource-limited settings where access to advanced diagnostic imaging was limited.   1. Needs Analysis:   A needs assessment was carried out prior to the program’s implementation, which involved surveys of potential participants, meetings with hospital administrators, and a review of hospital records to understand the clinical conditions most relevant for ultrasound application. This assessment helped determine the ultrasound applications to be included in the curriculum, such as abdominal ultrasound for free fluid (FAST), obstetric scans, and others relevant to the trainees’ clinical settings. The needs analysis emphasized the necessity of equipping local clinicians with basic diagnostic ultrasound skills to address gaps in diagnostic capacity.   1. Baseline Survey: None. 2. Impact on Instructional Design:   The baseline data from the needs assessment, including insights into the lack of diagnostic ultrasound in Rwanda, influenced the training program’s design. The focus was placed on practical applications of ultrasound that would have an immediate impact on clinical decision-making. The curriculum was designed to address common issues in low-resource settings, such as trauma (FAST), obstetrics, and abdominal conditions, all of which had direct implications for patient management. Follow-up training ensured that skills were retained and refined over time. Additionally, a cloud-based system was implemented for image archiving and remote review to ensure continuous quality control and feedback. |
| 3.2 Design | 1. Training Objectives:    1. Knowledge Goals: Provide physicians with the knowledge required for point-of-care ultrasound (POCUS) applications, specifically for abdominal imaging (free fluid) and obstetric imaging (fetal presentation, placental location).    2. Skill Goals: Enable physicians to independently perform POCUS scans, acquire diagnostic-quality images, and interpret findings to make informed clinical decisions.    3. Attitude Goals: Build confidence in using POCUS to guide clinical management and decision-making, particularly in resource-limited settings. 2. Training Methods:    1. Course Duration: 10-day initial training program, followed by 6 months of follow-up and data collection.    2. Course Structure:       1. First 10 Days: Theoretical lectures and practical scanning sessions. Trainees received hands-on training in performing POCUS with a focus on specific clinical applications (e.g., FAST for free fluid, obstetric imaging).       2. Follow-up: After the initial training, physicians continued using ultrasound in their hospitals, uploading images for remote review and receiving follow-up support every 6 weeks. This ensured ongoing skill retention and problem-solving.    3. Instructors:       1. PURE Faculty: Composed of emergency physicians, obstetricians, and radiologists with expertise in POCUS. They provided the core training and conducted image reviews for quality assurance.    4. Equipment:       1. Ultrasound Machines: Various ultrasound devices, including portable point-of-care ultrasound systems, were used in the training. Specific models are not mentioned in the article, but the focus is on portable devices suitable for field use in rural settings.       2. Cloud-based Data Storage: Images were stored on a cloud-based platform (e.g., Dropbox) for remote review by the instructors.    5. Assessment:       1. Objective Structured Clinical Examination (OSCE): Conducted at the end of the initial 10-day course to evaluate trainee competency in machine operation, image acquisition, and interpretation.       2. Image Quality Review: Trainee-performed scans were evaluated for quality by the PURE faculty using a standardized 0-4 grading scale.       3. Clinical Decision Making: Data was collected on whether ultrasound findings resulted in changes to patient management, including medication adjustments, procedure recommendations, or patient referrals. 3. Training Content/Plan/Syllabus:    1. Module 1: Introduction to Point-of-Care Ultrasound (First 10 Days)       1. Overview of POCUS principles, machine operation, and applications in emergency care and obstetrics.       2. Focus on abdominal ultrasound for free fluid assessment and obstetric imaging for fetal presentation, placental location, and biometry.    2. Module 2: Practical Ultrasound Scanning (First 10 Days)   Hands-on sessions for acquiring images and performing scans in a clinical setting, with supervision from experienced instructors.   - 1. Module 3: Data Collection and Follow-up (6 months post-training)      1. Trainees performed independent POCUS scans in their hospitals, recorded clinical data, and uploaded ultrasound images to the cloud for ongoing review and feedback.      2. Regular follow-up sessions provided continued support and guidance, reinforcing training and ensuring high-quality clinical practices. |
| 3.3 Development | 1. Instructors/Teachers:    1. Identity/Qualifications: The instructors for the obstetric point-of-care ultrasound training were consultant obstetricians and emergency physicians with expertise in ultrasound. They were part of the PURE faculty, which conducted the training for Rwandan physicians.    2. Instructor-to-Trainee Ratio: The training program had 17 participants, and the faculty provided personalized guidance. The instructors’ exact ratio to trainees wasn’t specified, but the training involved OSCE assessments and regular follow-up sessions for hands-on teaching.    3. Instructor Training Program: The instructors were responsible for teaching basic obstetric ultrasound techniques such as measuring fetal heart rate, placental location, and evaluating the gestational sac. They also provided continuous support through on-site follow-ups and remote image reviews to ensure ongoing skill development. 2. Teaching Aids:    1. Electronic Platforms: The trainees uploaded their ultrasound images to a cloud-based system (Dropbox) for review by the instructors, enabling remote feedback. This system allowed the instructors to assess the images and provide guidance, ensuring the quality and accuracy of the obstetric scans.    2. Devices: The training program used portable ultrasound devices suitable for obstetric imaging, including transabdominal obstetric ultrasounds for assessing gestational sac, fetal heart rate, and placental position. These devices were chosen to ensure that they could be used in resource-limited settings.    3. Models or Patients: The obstetric ultrasound training was conducted with real pregnant patients. Trainees practiced real-time scanning to assess fetal position, placental location, and other obstetric parameters. There was no mention of using models or simulators in the obstetric training. 3. Teaching Materials:    1. Lesson Plans, Lecture Notes, PPT: The curriculum for the obstetric ultrasound included theoretical sessions on the fetal heart rate, gestational age estimation, and placental location. Trainees were taught to recognize fetal lie and placenta previa, with instructional films and case studies supporting their learning.    2. Scales, Test Questions: The OSCE served as the primary evaluation tool, focusing on machine handling, image acquisition, and interpretation. Specific to obstetrics, the trainees were assessed on their ability to identify key obstetric conditions, such as fetal heart rate abnormalities and placental position.    3. Development of Materials: The training materials were designed to be contextual and practical, addressing the key needs of obstetric care in low-resource settings. The cloud-based image review system was integral for quality control, helping instructors assess the fetal images and guide trainees on their performance in real-world settings. |
| 3.4 Implementation | 1. Time:    1. Point of time: The training was conducted in 2012.    2. Total duration: The program lasted 10 days with 6 months of follow-up for continued support and skill assessment.    3. Schedule:       1. Days 1-10: Focused on theoretical training in obstetric ultrasound, covering topics like fetal heart rate, gestational sac visualization, and placental location.       2. Follow-up: After the initial 10 days, monthly follow-up sessions were conducted to reinforce the techniques and evaluate skill retention. 2. Location/Setting:    1. The training took place in Rwanda, within district hospitals and rural healthcare centers, providing an opportunity for hands-on practice in the local clinical setting.    2. Setting: Training occurred in clinical environments where participants practiced performing obstetric ultrasound scans on real patients. The focus was on practical application in low-resource settings, with ultrasound machines available for hands-on learning. 3. Participants:    1. Trainers: Experienced sonographers and obstetricians from the Point-of-Care Ultrasound in Resource-limited Environments (PURE) faculty facilitated the training. These trainers specialized in obstetric ultrasound and had experience in conducting training in resource-limited settings.    2. Trainees: 17 district hospital physicians were selected for the training. These individuals were primarily involved in obstetric care in rural regions and had limited prior ultrasound experience. 4. Execution Process:    1. The program began with 10 days of intensive, hands-on training, where participants learned to perform basic obstetric ultrasound, including fetal heart rate monitoring, gestational sac identification, and placental location.    2. Day 1-5: Focused on theoretical training in ultrasound physics and the clinical applications of obstetric ultrasound, with an emphasis on how ultrasound could aid in identifying gestational age, fetal viability, and placenta previa.    3. Day 6-10: The focus shifted to practical training, with supervised scanning on pregnant women in clinical settings. Trainees practiced performing ultrasound exams, using real-time scans to assess fetal positioning and placental location, with immediate feedback from trainers.    4. On the final day, participants completed an Objective Structured Clinical Examination (OSCE) to assess their practical skills in performing obstetric ultrasound scans.    5. Follow-up training was conducted every 6 weeks over the next 6 months, with trainers providing remote support via mobile technology, reviewing ultrasound images uploaded by the trainees and providing feedback. This was designed to ensure continued competency in the technique and reinforce the skills learned during the initial training. |
| 3.5 Evaluation | 1. Formative Evaluation:    1. Purpose: To evaluate the effectiveness of a Point-of-Care Ultrasound (POCUS) training program for physicians in Rwanda, with a focus on improving their skills in performing obstetric ultrasound (OB POCUS) and assessing the immediate outcomes of training.    2. Methods/Tools:       1. Objective Structured Clinical Examination (OSCE): An assessment at the end of the training course to evaluate competency in ultrasound operation, image acquisition, and interpretation.       2. Direct supervision: Initial training involved supervised practice, with trainees performing ultrasound scans under the guidance of instructors.    3. Timing: Formative evaluation occurred during the 10-day training course, which included both theoretical and hands-on components. 2. Summative Evaluation:    1. Purpose: To assess the long-term outcomes of the POCUS training, focusing on the trainees’ ability to independently perform accurate obstetric ultrasounds and their impact on clinical decision-making.    2. Methods/Tools:       1. Follow-up ultrasound scans: Trainees performed POCUS scans independently, with their images reviewed for quality and accuracy.       2. Clinical decision-making surveys: Data collection sheets were used to track how ultrasound findings influenced clinical decisions (e.g., changes in medication, referral, surgery, or discharge).       3. Image quality assessment: Expert reviewers graded the quality of ultrasound images on a scale from 0 to 4, assessing their completeness and diagnostic value.    3. Timing: Summative evaluation took place over 6 months following the initial training, with regular follow-up and periodic OSCEs to ensure skill retention. 3. Follow-up:    1. Purpose: To monitor the continued use of POCUS and the retention of skills over time, ensuring that trainees maintain competency and continue applying ultrasound in their clinical practice.    2. Methods/Tools:       1. Remote image review and feedback: Images were uploaded to a cloud-based system for expert review, with feedback provided to trainees to support ongoing learning.       2. Follow-up training: Periodic follow-up training sessions (every 6 weeks) were provided to reinforce skills and address any challenges in using ultrasound in practice.    3. Timing: Follow-up training and evaluation continued throughout the 6-month data collection period. |
|  | |
| **4. Outcome Characteristics** | |
| **Item** | **Extracted Data** |
| **4.1 Training Outcomes** |  |
| 4.1.1 Diagnostic Accuracy | 1. Test Performance of Obstetric Ultrasound:    1. Sensitivity: 100%    2. Specificity: 98% |
| 4.1.2 Knowledge Acquisition | NI |
| 4.1.3 Skills Acquisition | NI |
| 4.1.4 Knowledge Retention | NI |
| 4.1.5 Practical Application | NI |
| **4.2 Maternal and Neonatal Health Outcomes** (Including Physiological and Psychological Outcomes) | NI |
| **4.3 Health Economic Outcomes** | 1. Value of Training in Resource-Limited Settings: The study supports the cost-effectiveness and clinical value of ultrasound training for clinicians in resource-limited settings. 2. Cost-Effectiveness Considerations: Training costs, machine purchase and maintenance, and image archiving expenses must be offset by improvements in diagnostic accuracy and expedited care to justify the allocation of limited resources for ultrasound training and implementation in low-resource environments. |

**JBI Qualitative Data Extraction Tool**

**Reviewer: XXX & XXX & XXX Date: 28^th^ February 2025**

**Author:** **Kotagal et al. Year: 2015**

**Journal: Journal of Surgical Education Record Number: 16**

| **1. Study Characteristics** | |
| --- | --- |
| **Item** | **Extracted Data** |
| 1.1 Author (Publication Year) | Kotagal et al., 2015 |
| 1.2 Country | The United States of America |
| 1.3 Location | University of Washington, Seattle, Washington |
| 1.4 Study Design | Cohort study |
| 1.5 Sample Size | 16 |
|  | |
| **2. Population Characteristics (Healthcare Providers Receiving POCUS Training)** | |
| **Item** | **Extracted Data** |
| 2.1 Age | NI |
| 2.2 Gender | NI |
| 2.3 Professional Role | Doctors: 100% (16/16) |
| 2.4 Work Experience (Duration) | NI |
| 2.5 Prior Ultrasound Training Experience | NI |
|  | |
| **3. Intervention Characteristics (According to ADDIE Training Model)** | |
| **Item** | **Extracted Data** |
| 3.1 Analysis | 1. Trainee Group:   The trainees are surgical residents with limited prior experience in obstetric ultrasound.   1. Needs Analysis:   The training need arises because the trainees had no significant exposure to obstetric ultrasound, which is critical for assessing conditions such as fetal presentation, gestational age estimation, and ectopic pregnancy. Given the high importance of ultrasound in resource-limited settings, especially for obstetrics, the program aimed to fill this gap and increase confidence in using ultrasound for obstetric evaluations.   1. Baseline Survey: None. 2. Impact on Instructional Design:   The lack of experience with obstetric ultrasound influenced the training structure. The curriculum included lectures and hands-on practice specifically focusing on obstetric ultrasound applications, such as early pregnancy diagnosis, fetal heart rate, and fetal presentation. This curriculum was designed to address the trainees’ low baseline knowledge of obstetric ultrasound and build confidence in performing key obstetric ultrasound exams. |
| 3.2 Design | 1. Training Objectives:    1. Knowledge Goals: Enhance the knowledge of surgical residents in using point-of-care ultrasound (POCUS) for obstetrics, particularly in early and late pregnancy to identify fetal position, heart rate, and gestational age.    2. Skill Goals: Equip residents with the ability to perform basic obstetric ultrasound scans, including detecting early pregnancy, assessing fetal heart rate, and estimating gestational age using ultrasound.    3. Attitude Goals: Build confidence in using ultrasound to make clinical decisions regarding obstetric care, especially in resource-limited settings where ultrasound might be the primary diagnostic tool. 2. Training Methods:    1. Course Duration: 7 sessions, each lasting 2 hours, conducted over the course of 3 months.    2. Course Structure:       1. Lecture-Based Didactics: Each session included theoretical lectures that covered different ultrasound applications, including obstetrics.       2. Hands-On Workshops: After the lectures, residents participated in hands-on workshops using live models to practice ultrasound applications.    3. Instructors:       1. The course was taught by a multidisciplinary faculty including experts from the Departments of Surgery, Radiology, Anesthesia, and the Division of Emergency Medicine.    4. Equipment:       1. Ultrasound Devices: Portable ultrasound machines (e.g., M-Turbo from Sonosite) were used during training, suitable for clinical and field settings.       2. Hands-On Training Models: Live models were used for practical ultrasound scanning practice.    5. Assessment:       1. Pre- and Post-Course Surveys: Confidence and self-assessments of skill were evaluated using a 6-point Likert scale before and after the course.       2. OSCE (Objective Structured Clinical Examination): Residents performed scans under supervision to demonstrate competency in various ultrasound applications, including obstetrics. 3. Training Content/Plan/Syllabus:    1. Module 1: Introduction to Point-of-Care Ultrasound (Session 1)       1. Introduction to ultrasound principles and applications, including obstetrics, machine operation, and common artifacts.    2. Module 2: Obstetric Ultrasound (Sessions 2-7)       1. Early Pregnancy: Identifying early intrauterine pregnancy and signs of ectopic pregnancy.       2. Late Pregnancy: Assessing fetal heart rate, estimating gestational age, and determining fetal lie and position.    3. Module 3: Procedural Guidance and Other Applications (Sessions 2-7)       1. Vascular and Soft Tissue Ultrasound: Applications of ultrasound in clinical decision-making for soft tissue and vascular conditions.       2. Hands-on Practice: Application of learned skills on live models in a clinical setting, with feedback from instructors. |
| 3.3 Development | 1. Instructors/Teachers:    1. Identity/Qualifications: The instructors for the obstetric POCUS training were specialists in obstetrics and emergency medicine, with significant expertise in point-of-care ultrasound. These instructors were responsible for both the didactic teaching and hands-on training in obstetric ultrasound.    2. Instructor-to-Trainee Ratio: The course involved 16 surgical residents, and the training included 7 sessions of 2 hours each. The small group size allowed for a manageable instructor-to-trainee ratio, ensuring adequate hands-on supervision and practical experience during obstetric ultrasound applications.    3. Instructor Training Program: The instructors were highly experienced in teaching and practicing POCUS. They provided both theoretical knowledge in lectures and practical guidance during live patient scanning. Instructors ensured that each resident received personalized attention during the training, especially during hands-on practice with obstetric ultrasound applications. 2. Teaching Aids:    1. Electronic Platforms: The article did not specifically mention the use of electronic platforms for continued support or remote learning, but the hands-on training involved the use of live models for real-time practice of obstetric ultrasound techniques.    2. Devices: The training utilized portable ultrasound devices appropriate for obstetric applications, such as assessing fetal heart rate and estimating gestational age. These devices were chosen for their suitability in point-of-care environments, particularly in settings with limited resources.    3. Models or Patients: The practical training involved real patients in both early and late stages of pregnancy. The residents practiced obstetric ultrasound to assess fetal positioning, fetal heart rate, and gestational age. This hands-on approach with real patients was critical for developing skills that would be used in clinical practice. 3. Teaching Materials:    1. Lesson Plans, Lecture Notes, PPT: The curriculum featured lecture-based sessions on various obstetric ultrasound techniques, including early pregnancy detection (e.g., intrauterine pregnancy, ectopic pregnancy), as well as late-stage pregnancy assessments like fetal heart rate and gestational age estimation. PowerPoint slides and lecture notes were used to support these topics, providing a structured approach to learning.    2. Scales, Test Questions: Confidence surveys were used to assess the residents’ self-reported improvement in their ability to perform obstetric ultrasounds. These surveys were conducted before and after the training to evaluate the effectiveness of the program in boosting residents’ confidence in their skills.    3. Development of Materials: The training materials were carefully developed by a multidisciplinary faculty, designed to be simple and effective for surgical residents. These materials focused on practical, clinically relevant applications such as gestational age estimation, fetal heart rate, and fetal positioning. The training emphasized hands-on experience with real patients, which helped ensure that the materials were directly applicable to the residents’ clinical practice. The curriculum was designed to cater to both high-resource and low-resource settings, where such ultrasound skills are critical for diagnosing and managing obstetric conditions. |
| 3.4 Implementation | 1. Time:    1. Point of time: The training took place in 2014.    2. Total duration: The program lasted 3 months, with 7 sessions, each lasting 2 hours.    3. Schedule:       1. The sessions included didactic teaching and hands-on practice.       2. The focus of the training was on obstetric ultrasound, including early pregnancy (such as gestational sac visualization and fetal heart rate monitoring) and late pregnancy (like fetal presentation and placental location). 2. Location/Setting:    1. The training was conducted at a surgical training program in Seattle, Washington, in a clinical simulation environment.    2. Setting: Trainees practiced using live models and ultrasound machines in a hands-on setting, with immediate feedback from instructors to refine techniques and improve accuracy. 3. Participants:    1. Trainers: The program was led by experienced obstetricians, radiologists, and ultrasound specialists who had extensive knowledge in obstetric ultrasound.    2. Trainees: The program included 16 surgical residents who had limited prior ultrasound experience but were motivated to expand their skills in obstetric ultrasound for clinical use in surgical practice. 4. Execution Process:    1. The training began with theoretical teaching on obstetric ultrasound applications, specifically targeting early pregnancy and late pregnancy scans. The theoretical session covered gestational sac visualization, fetal heart rate monitoring, and placental position.    2. In the practical component, residents performed hands-on ultrasound scans on live models, learning how to visualize the fetus in the early and later stages of pregnancy.    3. Emphasis was placed on developing confidence in ultrasound application for assessing fetal position, gestational age, and placental location. Residents received real-time feedback from instructors during the hands-on scanning sessions to ensure correct technique and imaging.    4. By the end of the program, residents were expected to demonstrate proficiency in performing obstetric ultrasound and interpreting ultrasound findings related to fetal presentation and placenta localization. |
| 3.5 Evaluation | 1. Formative Evaluation:    1. Purpose: To evaluate the immediate outcomes of the POCUS training program, focusing on residents’ ability to perform ultrasound scans and their initial confidence levels.    2. Methods/Tools:       1. Pre- and post-course surveys to assess confidence in performing ultrasound for obstetric applications.       2. Objective Structured Clinical Examinations (OSCEs) to evaluate competency in ultrasound skills (machine operation, image acquisition, interpretation).       3. Data analysis: The results from the pre- and post-course surveys were analyzed using nonparametric tests (Wilcoxon rank-sum test) and paired t-tests to evaluate confidence improvements and skill acquisition.    3. Timing: Formative evaluation occurred immediately after the 7-session training program, including surveys and OSCEs. 2. Summative Evaluation:    1. Purpose: To assess the long-term impact of the POCUS training program, with a focus on residents’ diagnostic accuracy and their clinical decision-making skills post-training.    2. Methods/Tools:       1. Post-training confidence surveys to assess how the training influenced residents’ confidence in performing POCUS for obstetric applications.       2. Ultrasound image review: Residents’ images were graded for quality by expert reviewers.       3. Data analysis: The post-training data were analyzed using paired t-tests to compare pre- and post-training confidence and knowledge scores.    3. Timing: Summative evaluation occurred after the training program and involved post-course surveys and ultrasound image review. 3. Follow-up:    1. Purpose: To monitor the retention of ultrasound skills and assess whether residents continue using POCUS in their clinical practice.    2. Methods/Tools:       1. Follow-up surveys conducted at 3 months to track continued use of POCUS and confidence levels.       2. Ongoing feedback and periodic assessments to evaluate the long-term retention of skills.       3. Data analysis: Follow-up survey responses were analyzed using paired t-tests to evaluate the retention of confidence and skill improvements over time.    3. Timing: Follow-up evaluations occurred during the 6-month post-training period, with continuous monitoring of skills and confidence. |
|  | |
| **4. Outcome Characteristics** | |
| **Item** | **Extracted Data** |
| **4.1 Training Outcomes** |  |
| 4.1.1 Diagnostic Accuracy | NI |
| 4.1.2 Knowledge Acquisition | NI |
| 4.1.3 Skills Acquisition | NI |
| 4.1.4 Knowledge Retention | NI |
| 4.1.5 Practical Application | NI |
| **4.2 Maternal and Neonatal Health Outcomes** (Including Physiological and Psychological Outcomes) | NI |
| **4.3 Health Economic Outcomes** | NI |

**JBI Qualitative Data Extraction Tool**

**Reviewer: XXX & XXX & XXX Date: 28^th^ February 2025**

**Author: Miles et al. Year: 2023**

**Journal: Journal of Radiology Nursing Record Number: 17**

| **1. Study Characteristics** | |
| --- | --- |
| **Item** | **Extracted Data** |
| 1.1 Author (Publication Year) | Miles et al., 2023 |
| 1.2 Country | The United States of America |
| 1.3 Location | An urban, tertiary, 100-bed level I trauma center located in north Texas |
| 1.4 Study Design | Cohort study |
| 1.5 Sample Size | 11 |
|  | |
| **2. Population Characteristics (Healthcare Providers Receiving POCUS Training)** | |
| **Item** | **Extracted Data** |
| 2.1 Age | NI |
| 2.2 Gender | NI |
| 2.3 Professional Role | Nurses: 100% (11/11) |
| 2.4 Work Experience (Duration) | 1. General nursing experience:   Mean = 8.27 years (Range: 3-25 years; Mode: 5 years)   1. Emergency department nursing experience:   Mean = 9.09 years (Range: 4-27 years; Mode: 4 & 10 years) |
| 2.5 Prior Ultrasound Training Experience | (1) With prior ultrasound experience: 72.7% (8/11);  (2) Without prior ultrasound experience: 27.3% (3/11). |
|  | |
| **3. Intervention Characteristics (According to ADDIE Training Model)** | |
| **Item** | **Extracted Data** |
| 3.1 Analysis | 1. Trainee Group:   The trainees are emergency department nurses with varying levels of experience. The average years of nursing experience was 8.27 years (ranging from 3 to 25 years), and the average years of experience in the emergency department was 9.09 years. Several of these nurses had prior ultrasound experience specifically with ultrasound-guided intravenous catheter placement.   1. Needs Analysis:   The training need arose due to the limitations of hand-held Doppler (HHD) in fetal heart rate (FHR) assessment, which can sometimes result in misidentifying the maternal heart rate as the fetal heart rate, leading to false concerns about fetal distress. The nurses needed to be trained to accurately determine the FHR using bedside ultrasound (POCUS), specifically through M-mode to visualize and count the fetal heart rate. This is more reliable and provides a visual confirmation of fetal viability, especially when fetal positioning makes auditory detection via Doppler difficult.   1. Baseline Survey:   The baseline survey collected data on the nurses’ work experience and prior ultrasound training. It was found that 8 out of 11 nurses had prior ultrasound experience related to IV catheter placement, while 3 nurses had no previous ultrasound experience. This data influenced the training structure, particularly by recognizing that nurses with prior ultrasound experience may require less time to achieve competency.   1. Impact on Instructional Design:   The baseline data highlighted the varying levels of prior ultrasound experience among the nurses. The curriculum was structured to ensure that even nurses without prior ultrasound experience could achieve competency in using POCUS for fetal heart rate determination. The training included self-paced learning modules, followed by a 2-hour hands-on simulation, with supervised practice to ensure competency in fetal heart rate (FHR) detection using M-mode ultrasound. |
| 3.2 Design | 1. Training Objectives:    1. Knowledge Goals: Equip emergency department (ED) nurses with the knowledge to use point-of-care ultrasound (POCUS) to accurately determine fetal heart rates (FHR), especially in trauma or acute scenarios where fetal viability needs to be confirmed quickly.    2. Skill Goals: Train nurses to use POCUS to locate the fetal amniotic sac and determine the FHR using M-mode ultrasound technology.    3. Attitude Goals: Build confidence among ED nurses in utilizing ultrasound as a reliable and accurate tool for determining fetal heart rates, improving patient care and reducing the risk of misidentifying maternal heart rates. 2. Training Methods:    1. Course Duration: A self-paced learning module followed by a 2-hour hands-on clinical session.    2. Course Structure:       1. Pre-Course Training: Nurses completed a 27-minute instructional video covering the basics of POCUS to locate the fetal amniotic sac and measure the FHR using M-mode.       2. Classroom and Hands-On Training: A 1-hour classroom session followed by practical training in the ED simulation lab, where nurses practiced using POCUS on live patients at different stages of pregnancy.    3. Instructors:       1. The training was led by ED physicians and nurses with expertise in ultrasound. The instructors were credentialed in ultrasound use and supervised the clinical application of the technique.    4. Equipment:       1. Ultrasound Device: The Sonosite X-Porte was used, a portable bedside ultrasound machine designed for clinical settings to perform diagnostic imaging, including obstetric scans.       2. Hand-Held Doppler (HHD): Nurses were also trained using the HHD for FHR assessment to compare against POCUS.    5. Assessment:       1. Clinical Supervision: Nurses demonstrated their ability to independently use POCUS to locate the fetal heart and measure the FHR, with physician supervision.       2. Competency Achievement: Nurses needed to achieve competency in locating the fetal heart and determining the FHR using POCUS, with competency being confirmed through clinical supervision and documentation. 3. Training Content/Plan/Syllabus:    1. Module 1: Introduction to Bedside Ultrasound for FHR (Pre-Course)       1. Self-paced video covering basic ultrasound principles, probe positioning, and the use of M-mode to measure FHR.    2. Module 2: Hands-On Practical Training (2 hours)       1. In-depth hands-on session where nurses practiced locating the fetal amniotic sac and measuring the FHR using the M-mode feature of the ultrasound device.    3. Module 3: Application and Competency Validation (Clinical Setting)       1. Nurses applied their skills on patients in the ED under supervision. They were required to demonstrate competency in locating the fetal heart and measuring the FHR using POCUS. |
| 3.3 Development | 1. Instructors/Teachers:    1. Identity/Qualifications: The instructors were the ED ultrasound program director and two nurses who had already been trained and competent in fetal heart rate (FHR) determination using POCUS. They had experience teaching ultrasound techniques in the emergency department (ED) setting.    2. Instructor-to-Trainee Ratio: The training was attended by 13 nurses, with 11 nurses completing the program and assessed for competency. The instructor-to-trainee ratio was 1:5.5 for the 11 participants, providing adequate hands-on supervision and guidance.    3. Instructor Training Program: The instructors delivered didactic education and hands-on training with a focus on guiding the nurses through the process of fetal heart rate determination using ultrasound, specifically M-mode. They also oversaw practice on real patients to ensure competency. 2. Teaching Aids:    1. Electronic Platforms: The nurses were given access to a 27-minute instructional video titled “The Possibly Pregnant Patient,” which covered fetal heart rate assessment using POCUS and demonstrated the proper technique for using M-mode on the ultrasound machine.    2. Devices: The Sonosite X-Porte ultrasound system was utilized for the POCUS training. This portable ultrasound device with M-mode allowed the nurses to accurately measure fetal heart rate and practice scanning real patients.    3. Models or Patients: Nurses performed the training on real pregnant patients. These patients, at various stages of pregnancy, volunteered for the nurses to practice determining fetal heart rate using the ultrasound system. 3. Teaching Materials:    1. Lesson Plans, Lecture Notes, PPT: The training included both didactic sessions and practical training. The lesson plans involved a 1-hour classroom session supplemented by the instructional video and live demonstrations on ultrasound probe positioning and fetal heart rate determination.    2. Scales, Test Questions: Nurse participants were assessed for competency based on their ability to locate the fetal heart rate using POCUS. Competency worksheets were signed off by the bedside ED physicians. Nurses also filled out post-training surveys to gauge their confidence in using POCUS for fetal heart rate determination.    3. Development of Materials: The instructional materials, including the video and competency assessment worksheets, were developed by the ED ultrasound program director. These materials were designed to ensure that the nurses could effectively use POCUS for fetal heart rate determination in an emergency department setting. |
| 3.4 Implementation | 1. Time:    1. Point of time: The training took place between May 9 to November 29, 2022.    2. Total duration: The training included self-paced learning followed by a 2-hour hands-on simulation.    3. Schedule:       1. Pre-class: Nurses watched a 27-minute video on fetal heart rate (FHR) determination using POCUS.       2. Classroom training: A 1-hour lecture followed by hands-on practice on fetal amniotic sac location and FHR measurement using M-mode ultrasound. 2. Location/Setting:    1. The training occurred at a 100-bed Level I trauma center in north Texas.    2. Setting: The class was held in an ED simulation lab where nurses practiced on patients at various stages of pregnancy. 3. Participants:    1. Trainers: The training was led by an ED ultrasound program director and 2 nurses trained and deemed competent by the program director.    2. Trainees: 13 nurses attended, with 11 completing the clinical component. These nurses had varying levels of ultrasound experience, including some with experience in US-guided IV insertion. 4. Execution Process:    1. Pre-class: Nurses watched a video explaining how to position the probe and use the M-mode on the POCUS machine to locate the fetal amniotic sac and measure the FHR.    2. Classroom training: Nurses received 1 hour of lecture on fetal heart rate determination and POCUS techniques, followed by hands-on training with real patients at different stages of pregnancy to practice locating the fetal heart and measuring FHR.    3. Clinical practice: Nurses practiced FHR determination under the supervision of an ED physician credentialed to read ultrasound images. After sufficient practice, they transitioned to performing the procedure independently to achieve clinical competence.    4. Competency evaluation: Nurses’ competency in using POCUS to determine FHR was assessed using a Likert scale based on clinical supervision and hands-on application. Competence was achieved by performing a specified number of successful scans (average of 3.25 attempts to obtain competent measurements). |
| 3.5 Evaluation | 1. Formative Evaluation:    1. Purpose: To assess the immediate effectiveness of the POCUS training program, focusing on nurses’ ability to determine fetal heart rates (FHR) and their confidence in using the technology.    2. Methods/Tools:       1. Competency assessments: Nurses’ ability to use POCUS to locate the fetal amniotic sac and measure the FHR was evaluated under clinical supervision by physicians.       2. Confidence surveys: Nurses completed two separate Likert-scale surveys: one post-class survey to assess immediate confidence after training and one post-training survey to evaluate feedback on the program and their confidence in using POCUS for FHR determination.    3. Timing: Formative evaluation occurred immediately after the training and hands-on practice, with both surveys and clinical assessments used to measure initial competency and confidence. 2. Summative Evaluation:    1. Purpose: To assess the long-term impact of POCUS training, focusing on the accuracy of FHR determination and comparing the time efficiency of POCUS versus the hand-held Doppler (HHD) method.    2. Methods/Tools:       1. Clinical performance: Nurses performed FHR measurements on patients using both POCUS and HHD, and the time taken for each method was recorded.       2. Statistical analysis:The Wilcoxon signed-rank test was used to compare the time taken to determine FHR using POCUS versus HHD. Bland-Altman plot was used to assess the agreement between the two methods.    3. Timing: Summative evaluation occurred after clinical application of POCUS, during real patient assessments in the emergency department. 3. Follow-up:    1. Purpose: To monitor the retention of POCUS skills and assess continued use in clinical practice, ensuring that nurses maintain competency and confidence in FHR determination.    2. Methods/Tools:       1. Follow-up surveys: Nurses completed follow-up surveys after the clinical implementation to assess continued confidence and identify any barriers or challenges faced in using POCUS.       2. Clinical performance review: Physicians continued to supervise and assess nurses using POCUS in clinical practice.    3. Timing: Follow-up occurred after clinical implementation of POCUS in the emergency department, with ongoing supervision over several months. |
|  | |
| **4. Outcome Characteristics** | |
| **Item** | **Extracted Data** |
| **4.1 Training Outcomes** |  |
| 4.1.1 Diagnostic Accuracy | NI |
| 4.1.2 Knowledge Acquisition | NI |
| 4.1.3 Skills Acquisition | 1. Competency Achievement:    1. 72.7% (8/11) nurses achieved clinical competency.    2. Mean attempts to achieve competency: 3.25 (Range: 2-5 attempts; Mode: 2 attempts).    3. Nurses with prior ultrasound experience (8/11) showed higher likelihood of achieving competency (7/8 succeeded), but prior experience was not mandatory as one nurse without experience also achieved competency.   (2) Confidence Improvement:   - 1. Post-class Confidence Survey: Completed by all 13 participants (including 2 nurses did not participate in the clinical setting) with positive responses.   2. Post-project Confidence Survey: Completed by 10 of 11 nurses after the clinical application phase, with favorable responses regarding confidence in obtaining fetal heart rates (FHRs) using point-of-care ultrasound (POCUS). |
| 4.1.4 Knowledge Retention | NI |
| 4.1.5 Practical Application | NI |
| **4.2 Maternal and Neonatal Health Outcomes** (Including Physiological and Psychological Outcomes) | 1. Psychological Outcomes:   Nurses noted reduced patient anxiety when visual confirmation of FHR was achieved using POCUS. |
| **4.3 Health Economic Outcomes** | NI |

**JBI Qualitative Data Extraction Tool**

**Reviewer: XXX & XXX & XXX Date: 28^th^ February 2025**

**Author: Rominger et al. Year: 2018**

**Journal: Critical Ultrasound Journal Record Number: 18**

| **1. Study Characteristics** | |
| --- | --- |
| **Item** | **Extracted Data** |
| 1.1 Author (Publication Year) | Rominger et al., 2018 |
| 1.2 Country | the United Mexican States |
| 1.3 Location | Rural clinics in the state of Chiapas |
| 1.4 Study Design | Cohort study |
| 1.5 Sample Size | Supervisors: 8  Trainees: NI |
|  | |
| **2. Population Characteristics (Healthcare Providers Receiving POCUS Training)** | |
| **Item** | **Extracted Data** |
| 2.1 Age | NI |
| 2.2 Gender | NI |
| 2.3 Professional Role | NI |
| 2.4 Work Experience (Duration) | NI |
| 2.5 Prior Ultrasound Training Experience | NI |
|  | |
| **3. Intervention Characteristics (According to ADDIE Training Model)** | |
| **Item** | **Extracted Data** |
| 3.1 Analysis | 1. Trainee Group:   The trainees are physicians working at rural outpatient clinics in Chiapas, Mexico. These physicians, who are fulfilling their social service year, had little to no prior experience with ultrasound. They are responsible for supervising medical interns (pasantes) and for providing patient care. These physicians were selected due to their long-term presence in their positions, which makes them ideal for sustaining the use of Point-of-Care Ultrasound (POCUS) in these rural settings.   1. Needs Analysis:   The article does not mention conducting a formal needs assessment. However, the curriculum was designed to address the identified needs of the clinics, specifically focusing on obstetric POCUS. Local physicians were consulted to identify which ultrasound applications would be most beneficial. Based on the healthcare challenges in the region, the curriculum prioritized obstetric ultrasound to detect conditions like placenta previa, multiple pregnancies, fetal position abnormalities, and other maternal health complications. This decision was driven by the physicians’ expertise and the common health issues faced by pregnant women in the area.   1. Baseline Survey:   There is no mention of a formal baseline survey or assessment in the article. However, it is clear that the physicians had minimal prior experience with ultrasound before the start of the program. The primary goal of the training was to build their skills from the ground up, focusing on obstetric ultrasound applications relevant to the region’s health concerns.   1. Impact on Instructional Design:   The lack of prior ultrasound experience among the trainees, along with the identified healthcare needs in the region, strongly influenced the design of the curriculum. The training was structured to start with the basics of ultrasound technology and gradually move towards more complex applications, such as obstetric ultrasound. The curriculum was divided into multiple sessions over the course of the year, with each session focusing on different aspects of ultrasound use. Obstetric ultrasound was emphasized early on due to the high relevance for maternal and fetal health in this rural, resource-limited setting. |
| 3.2 Design | 1. Training Objectives:    1. Knowledge Goals: Equip physicians with the knowledge to perform obstetric POCUS, including evaluating fetal heart rate, fetal position, placental location, and diagnosing complications like ectopic pregnancy and placenta previa.    2. Skill Goals: Enable physicians to perform transabdominal obstetric ultrasound to assess fetal viability, determine fetal presentation, and estimate gestational age.    3. Attitude Goals: Build confidence in using ultrasound for accurate clinical decision-making in obstetrics, especially in resource-limited settings. 2. Training Methods:    1. Course Duration: A 12-month longitudinal program, consisting of four training sessions over the year.    2. Structure:       1. Each session involves a combination of lectures and hands-on practice, with a focus on obstetric ultrasound applications. Sessions are spaced every 3-4 months to allow for skill practice and retention.       2. Hands-On Training: Physicians practice using the ultrasound on real patients at rural community clinics to develop their skills in obstetric imaging.    3. Instructors: The training is conducted by a team of experienced professionals, including an OB/GYN instructor from Harvard University, as well as Emergency Medicine and Pediatric Emergency Medicine experts.    4. Equipment:       1. Sonosite Nanomaxx Ultrasound Machines with both low-frequency (phased array) and high-frequency (linear) probes for obstetric scanning.       2. Portable ultrasound devices are rotated among clinics, giving physicians hands-on experience at their own clinical sites.    5. Assessment:       1. Ultrasound Logs: Physicians document each obstetric ultrasound, noting the type, clinical reason, and whether the results influenced patient management.       2. Image Review: Ultrasound images are periodically reviewed for quality and accuracy, with feedback provided to ensure continuous improvement. 3. Training Content/Plan/Syllabus:    1. Session 1: Introduction to obstetric ultrasound, including early pregnancy assessment, fetal heartbeat identification, and basic techniques for detecting ectopic pregnancies.    2. Session 2: Advanced obstetric ultrasound, focusing on fetal heart rate, fetal lie, and placental location, as well as early detection of complications such as placenta previa.    3. Session 3: Hands-on practice in rural community clinics, where physicians apply their skills to real patient cases.    4. Session 4: Review and refinement of techniques, with feedback on challenging cases and real-time problem-solving. |
| 3.3 Development | 1. Instructors/Teachers:    1. Identity/Qualifications: The instructors for the obstetric POCUS sessions included a Pediatric Emergency Medicine (PEM) physician, an Emergency Medicine (EM) physician, a PEM fellow, and two upper-level EM residents from the University of Louisville. Additionally, an Obstetrician from Harvard University/Brigham and Women’s Hospital taught the obstetric ultrasound module. The faculty members had extensive bedside ultrasound experience and had taught ultrasound at major national and international conferences.    2. Instructor-to-Trainee Ratio: The training had a small group size with 6-10 learners per two instructors, ensuring close supervision and individualized attention during both lectures and hands-on sessions.    3. Instructor Training Program: Instructors delivered both theoretical lectures and hands-on teaching, guiding physicians through various POCUS topics such as focused assessment with sonography in trauma (FAST), renal ultrasound, and basic obstetrics. The instructors’ experience ensured effective teaching of practical skills and immediate feedback for learners. 2. Teaching Aids:    1. Electronic Platforms: The article does not specify the use of electronic platforms for ongoing training, but the program included video lectures and hands-on practice during the sessions.    2. Devices: The Sonosite Nanomaxx ultrasound machines were used for the training, each equipped with low-frequency (phased array) and high-frequency (linear) probes suitable for obstetric imaging. These machines were portable, making them ideal for use in the rural clinics of Chiapas.    3. Models or Patients: The training sessions included both healthy volunteers and patients with known relevant pathologies. The trainees practiced performing obstetric ultrasounds on these patients, which included assessing conditions like placenta previa, multiple pregnancies, and breech presentation. 3. Teaching Materials:    1. Lesson Plans, Lecture Notes, PPT: The curriculum was divided into four teaching sessions over the course of the year. Each session covered different POCUS topics, including obstetric ultrasound. The obstetric ultrasound training was delivered through lectures and hands-on practice on both healthy volunteers and patients. Presentations from each session were provided to the participants for later reference.    2. Scales, Test Questions: The training was assessed based on clinical logs that tracked the types of ultrasound performed, the reason for the study, and whether the ultrasound findings changed the diagnosis or management. This log data provided a way to track the learning outcomes and identify areas for further instruction. However, specific written tests or formal exams were not mentioned.    3. Development of Materials: The teaching materials were developed in collaboration with the CES organization and were adapted to address the regional needs of physicians working in rural clinics. The curriculum focused on practical, clinical applications of ultrasound, ensuring that the materials were relevant to the healthcare challenges faced in rural Chiapas. |
| 3.4 Implementation | 1. Time:    1. Point of time: The training sessions started in September 2015 and continued for 12 months, with repeated sessions at intervals.    2. Total duration: The course lasted 4 days per session, with four sessions conducted over the year.    3. Schedule:       1. Day 1-1.5: Lecture-based teaching on ultrasound fundamentals, machine use, and the basics of obstetric ultrasound.       2. Day 2-4: Hands-on training on healthy volunteers and patients with known relevant pathologies in rural community clinics. 2. Location/Setting:    1. The training took place at rural clinics in Chiapas, Mexico.    2. Setting: The program used portable Sonosite ultrasound machines, and training occurred both in lecture halls and community health clinics. The practical training sessions took place directly with patients in rural areas, allowing trainees to apply their skills in real-life clinical situations. 3. Participants:    1. Trainers: The trainers included an Obstetrician from Harvard University and instructors from the University of Louisville, with extensive POCUS experience.    2. Trainees: The participants were local physicians working in the Compañeros En Salud (CES) clinics in Chiapas, Mexico. These were supervising physicians who had completed their social service year and were responsible for educating other healthcare staff. 4. Execution Process:    1. The first session of the program focused on introducing ultrasound machine use, followed by basic obstetric ultrasound, including gestational sac identification, fetal heart rate monitoring, and placental localization.    2. Hands-on training was conducted in the field with real patients to practice these skills, specifically on obstetric cases such as placenta previa, multiple gestations, and fetal positioning.    3. The training was broken down into 4 sessions spread across the year, each session with specific hands-on practice (approx. 48-56 hours) and didactic teaching (approx. 16 hours of lectures).    4. After each session, the trainees practiced on patients and documented their ultrasound findings. These findings were reviewed, with feedback from instructors during the sessions and via remote channels.    5. The program included follow-up sessions to reinforce the skills learned and ensure continuous competency. |
| 3.5 Evaluation | 1. Formative Evaluation:    1. Purpose: To assess the immediate effectiveness of the POCUS training for physicians, focusing on their ability to use ultrasound to evaluate obstetric cases and their confidence in using this tool.    2. Methods/Tools:       1. Likert-scale surveys: Two separate surveys were conducted—one post-class and one post-training—to evaluate participants’ confidence in performing obstetric ultrasounds and their feedback on the training program.       2. Competency assessments: Physicians’ performance was assessed through clinical supervision and feedback during their use of POCUS in real patient encounters.    3. Timing: Formative evaluation occurred immediately after the training sessions, with feedback provided to assess knowledge and confidence. 2. Summative Evaluation:    1. Purpose: To evaluate the long-term impact of the POCUS training, focusing on the accuracy of diagnoses made using POCUS and the clinical management changes influenced by ultrasound findings.    2. Methods/Tools:       1. Clinical logs: Physicians kept logs of all ultrasound studies, including the reason for the study, the diagnosis, and whether the ultrasound findings changed the initial diagnosis or clinical management.       2. Data analysis: Ultrasound logs were reviewed to assess the effect of POCUS on clinical decision-making. The Wilcoxon signed-rank test and Bland-Altman analysis were used to compare pre- and post-training clinical management and diagnoses.    3. Timing: Summative evaluation occurred over the 12-month curriculum, with ongoing monitoring of clinical practice and diagnostic impact. 3. Follow-up:    1. Purpose: To assess the retention of skills and continued application of POCUS in clinical practice after the initial training, and to ensure that physicians-maintained competency in obstetric ultrasound.    2. Methods/Tools:       1. Follow-up surveys: Physicians were surveyed at 6 and 12 months after the initial training to assess their continued confidence and challenges in using POCUS for obstetric care.       2. Image review: A random sample of ultrasound images was reviewed to assess the quality and accuracy of diagnoses and the continued adherence to clinical protocols.       3. Clinical performance review: Supervisors continued to monitor and evaluate the use of POCUS in patient care.    3. Timing: Follow-up occurred 6- and 12-months post-training, with continuous feedback and ongoing supervision provided. |
|  | |
| **4. Outcome Characteristics** | |
| **Item** | **Extracted Data** |
| **4.1 Training Outcomes** |  |
| 4.1.1 Diagnostic Accuracy | NI |
| 4.1.2 Knowledge Acquisition | NI |
| 4.1.3 Skills Acquisition | NI |
| 4.1.4 Knowledge Retention | 1. Skill Retention Over Time: POCUS usage peaked immediately after educational sessions and decreased to a higher plateau after each session, suggesting improved long-term skill retention. 2. Reinforcement Through Repeated Sessions: Repeated training sessions with technical reviews and skill reinforcement improved retention and revalidated previously taught skills, evidenced by a low disagreement rate in scan interpretations. |
| 4.1.5 Practical Application | NI |
| **4.2 Maternal and Neonatal Health Outcomes** (Including Physiological and Psychological Outcomes) | 1. The obstetric ultrasounds were the most commonly done and changed the diagnosis in 24.4% (64/262) patient encounters and changed the management in 20.2% (53/262) patient encounters. |
| **4.3 Health Economic Outcomes** | NI |

**JBI Qualitative Data Extraction Tool**

**Reviewer: XXX & XXX & XXX Date: 28^th^ February 2025**

**Author: Shah et al. Year: 2020**

**Journal: PLOS ONE Record Number: 19**

| **1. Study Characteristics** | |
| --- | --- |
| **Item** | **Extracted Data** |
| 1.1 Author (Publication Year) | Shah et al., 2020 |
| 1.2 Country | Uganda |
| 1.3 Location | At one Public District Hospital (DH) and three Health Centers (HC) in Busoga Region. |
| 1.4 Study Design | Cohort study |
| 1.5 Sample Size | 25 |
|  | |
| **2. Population Characteristics (Healthcare Providers Receiving POCUS Training)** | |
| **Item** | **Extracted Data** |
| 2.1 Age | NI |
| 2.2 Gender | 1. Male: 8.0% (2/25); 2. Female: 92.0% (23/25). |
| 2.3 Professional Role | 1. Doctors: 8.0% (2/25); 2. Nurses: 12.0% (3/25); 3. Midwives: 80.0% (20/25). |
| 2.4 Work Experience (Duration) | NI |
| 2.5 Prior Ultrasound Training Experience | 1. Doctors: None (0/2); 2. Nurses: None (0/3); 3. Midwives: None (0/20). |
|  | |
| **3. Intervention Characteristics (According to ADDIE Training Model)** | |
| **Item** | **Extracted Data** |
| 3.1 Analysis | 1. Trainee Group:   Nurse, midwives and physicians providing assessment, diagnostic, and management services to women in maternity ward at one district hospital and three health centers in Uganda.   1. Needs Analysis:   Health care workers in low-resource areas need to be trained to identify high-risk conditions before pregnancy.   1. Baseline Survey:   All trainees had no prior ultrasound experience and rated themselves as “not confident, 1” on a 7-point Likert scale regarding ultrasound-related skills.   1. Impact on Instructional Design:   Given the trainees’ lack of ultrasound experience, the training curriculum focused on basic knowledge and skills. The training also emphasized hands-on practice to quickly build practical skills. |
| 3.2 Design | 1. Training Objectives:    1. Skill goals: To train trainees’ ability to identify high-risk pregnancies and interpret ultrasound images.    2. Attitude goals: Improving learner confidence. 2. Training Methods:   The training adopted a blended learning approach, including home study, in-person short lectures, hands-on demonstrations on healthy volunteers, hands-on live scanning practice on antenatal patients, and mock enrollments with active labor patients.   1. Training Content:   The training content focused on identifying high-risk conditions in late pregnancy. It started with basic knowledge like ultrasound physics and knob functions, then moved on to more complex aspects such as fetal position determination, placenta location assessment, and gestational age estimation. |
| 3.3 Development | 1. Instructors/Teachers:    1. Instructors included a certified Ugandan sonographer and several American-based providers with relevant experience. The instructor-to-trainee ratio was maintained at 1:3 during hands-on scanning activities.    2. Three district hospital nurse midwives with excellent ultrasound skills underwent a 1 day “Training of Trainers (TOT)” to assist in training health centers trainees. 2. Teaching Aids:    1. The main teaching aids were ultrasound machines used for hands-on practice.    2. Pregnant volunteers from antenatal clinics served as models for scanning practice. 3. Teaching Materials:   The course materials included flash drives with manuals (Printed materials from International Society of Ultrasound in Obstetrics and Gynecology (ISUOG) Manual, Partners in Health Manual of Ultrasound were used), video lectures, printed slides from lecture series, and practical scanning sessions. |
| 3.4 Implementation | 1. Time:   The training was rolled out in 2 phases, and each training phase lasted 2 weeks.   - 1. Phase 1: District hospital trainees was in October 2018. There were daily hour-long lectures and hands-on scanning practice;   2. Phase 2: Health centers trainees was in January 2019. Health centers trainees had a 2-day intensive training at the district hospital, followed by continued hands-on training at their own facilities for several weeks;  1. Location/Setting:    1. Phase 1 training for district hospital trainees was likely at the district hospital;    2. Phase 2 training for health centers trainees started with a 2-day intensive training at the district hospital and then continued at their respective health centers;    3. All training mainly took place in clinical settings such as antenatal clinics and labor triage areas for practical scanning; 2. Participants:    1. Trainers: A Ugandan sonographer and American-based providers;    2. Trainees: 20 nurse midwives, 3 nurses, and 2 physicians from one district hospital and three health centers; 3. Execution Process:   Trainees first studied at home using provided materials, then attended in-person lectures, hands-on demonstrations, and scanning practice. After that, they completed 25 proctored scans and an Observed Structured Clinical Ultrasound Exam (OSCE). Throughout the training, twice-weekly communication via WhatsApp was used for feedback and troubleshooting.   1. Adaptation Records:   The training was adjusted based on the experience of Phase 1. For Phase 2, the training schedule was modified to include shorter lectures, more hands-on practice, and the use of Master Trainers. |
| 3.5 Evaluation | 1. Formative Evaluation:    1. Purpose: To monitor trainees’ progress during the training;    2. Methods: Surveys and twice-weekly communication via WhatsApp for feedback.    3. Timing: At pre-training, immediately after, and 3-month follow-up. 2. Summative Evaluation:    1. Purpose: To assess trainees' overall achievement at the end of the training.    2. Method: Observed Structured Clinical Ultrasound Exam (OSCE) exam.    3. Timing: After trainees completed 25 proctored scans. 3. Follow-up:    1. Method: A follow-up survey.    2. Timing: 3 months after training. |
|  | |
| **4. Outcome Characteristics** | |
| **Item** | **Extracted Data** |
| **4.1 Training Outcomes** |  |
| 4.1.1 Diagnostic Accuracy | 1. Overall quality of image and measurements on the Image (Kappa):    1. Fetal heart rate, head position, fluid and femur length: 1.0.    2. All other measures: 0.829-0.928. 2. Accuracy of Images (For 8 weeks training):   Weeks 1-4   - 1. Quality (Images rated as 3,4, or 5 in ACEP Quality Assurance 5-point Grading Scale are considered to be of interpretable quality):      1. Fetal Heart Rate (Images reviewed n=262): 100% (262/262);      2. Estimated Gestational Age: Biparietal Diameter (Images reviewed n=255): 80.0% (204/255);      3. Estimated Gestational Age: Head Circumference (Images reviewed n=253): 81.8% (207/253);      4. Estimated Gestational Age: Femur Length (Images reviewed n=242): 97.1% (235/242);      5. Estimated Gestational Age: Transcerebellar Diameter (Images reviewed n=4): 75.0% (3/4).      6. Head Position (Images reviewed n=259): 98.8% (256/259).      7. Placental Location (Images reviewed n=246): 98.0% (241/246).      8. Amniotic Fluid (Images reviewed n=233): 97.0% (226/233).   2. Measure Acceptable (Any measurement errors were categorized individually as reasons to deem images unacceptable):      1. Fetal Heart Rate (Images reviewed n=262): 100% (262/262).      2. Estimated Gestational Age: Biparietal Diameter (Images reviewed n=255): 80.0% (204/255).      3. Estimated Gestational Age: Head Circumference (Images reviewed n=253): 66.0% (167/253).      4. Estimated Gestational Age: Femur Length (Images reviewed n=242): 95.0% (230/242).      5. Estimated Gestational Age: Transcerebellar Diameter (Images reviewed n=4): 75.0% (3/4).   Weeks 5-8   - 1. Quality      1. Fetal Heart Rate (Images reviewed n=224): 100% (224/224).      2. Estimated Gestational Age: Biparietal Diameter (Images reviewed n=204): 89.2% (182/204).      3. Estimated Gestational Age: Head Circumference (Images reviewed n=204): 88.2% (180/204).      4. Estimated Gestational Age: Femur Length (Images reviewed n=200): 99.0% (198/200).      5. Estimated Gestational Age: Transcerebellar Diameter (Images reviewed n=8): 75% (6/8).      6. Head Position (Images reviewed n=218): 98.2% (214/218).      7. Placental Location (Images reviewed n=195): 97.9% (191/195).      8. Amniotic Fluid (Images reviewed n=200): 99.0% (198/200).   2. Measure Acceptable      1. Fetal Heart Rate (Images reviewed n=224): 100% (224/224).      2. Estimated Gestational Age: Biparietal Diameter (Images reviewed n=204): 89.2% (182/204).      3. Estimated Gestational Age: Head Circumference (Images reviewed n=204): 78.9% (161/204).      4. Estimated Gestational Age: Femur Length (Images reviewed n=200): 99.0% (198/200).      5. Estimated Gestational Age: Transcerebellar Diameter (Images reviewed n=8): 75% (6/8);   Weeks 8 (Reviewers Cohen’s kappa)   - 1. Quality      1. Fetal Heart Rate: 1.      2. Estimated Gestational Age: Biparietal Diameter: 0.83.      3. Estimated Gestational Age: Head Circumference: 0.848.      4. Estimated Gestational Age: Femur Length: 1.      5. Head Position: 1.      6. Placental Location: 1.      7. Amniotic Fluid: 0.928.   2. Measure Acceptable      1. Fetal Heart Rate: 1.      2. Estimated Gestational Age: Biparietal Diameter: 0.829.      3. Estimated Gestational Age: Head Circumference: 0.92.      4. Estimated Gestational Age: Femur Length: 1. |
| 4.1.2 Knowledge Acquisition | NI |
| 4.1.3 Skills Acquisition | 1. Changes in learner confidence immediately post-course (All measures are from 1):    1. Measuring fetal heart rate-Mean (SD): 6.63 (0.5).    2. Assessing malpresentation-Mean (SD): 6.63 (0.5).    3. Identifying multiple gestation-Mean (SD): 6.06 (0.77).    4. Placenta previa-Mean (SD): 5.60 (0.99).    5. Oligohydramnios-Mean (SD): 6.06 (0.77).    6. Measures of gestational age for Biparietal Diameter-Mean (SD): 6.07 (0.80).    7. Measures of gestational age for Head Circumference-Mean (SD): 6.13 (0.81);    8. Measures of gestational age for Transcranial doppler-Mean (SD): 3.80 (1.41). 2. Overall Participants Pass Rate: 96.0% (24/25). 3. OSCE Passing Results:    1. Number of participants passing exam:       1. District Hospital (n = 11): 90.9% (10/11);       2. Health Centers (n = 14): 85.7% (12/14);    2. Average score of first-attempt passing candidates (With a total score is 34, a trainee passed when achieving 80% correct):       1. District Hospital (n = 11): 88.2% (29.988/34).       2. Health Centers (n = 14): 90.4% (30.736/34). 4. Numbers of participants performing correctly in OSCE:    1. Fetal Heart Rate: 92.0% (23/25).    2. Fetal Position/ Presentation: 100% (25/25).    3. Placenta Location: 92.0% (23/25).    4. Deepest Vertical Pocket of Amniotic Fluid: 84.0% (21/25).    5. Estimated Gestational Age: Transcranial Doppler: 52.0% (13/25).    6. Estimated Gestational Age: Biparietal Diameter: 56.0% (14/25).    7. Estimated Gestational Age: Femur Length: 48.0% (12/25).    8. Estimated Gestational Age: Transcranial Doppler: 12.0% (3/25).    9. Estimated Gestational Age: use of aggregate report function: 92.0% (23/25). 5. The total error rate in ultrasound performance and the rates of common errors such as “Uninterpretable Image”, “Wrong Plane of Measurement", and “Overestimating Gestational Age (Calipers too wide)” generally show a downward - trend over the 8-week period after training, indicating an improvement in ultrasound operation skills over time. |
| 4.1.4 Knowledge Retention | NI |
| 4.1.5 Practical Application | 1. Number of Clinical Scans:    1. Range: 30-250.    2. Mean: 80. |
| **4.2 Maternal and Neonatal Health Outcomes** (Including Physiological and Psychological Outcomes) | NI |
| **4.3 Health Economic Outcomes** | NI |

**JBI Qualitative Data Extraction Tool**

**Reviewer: XXX & XXX & XXX Date: 28^th^ February 2025**

**Author: Varner et al. Year: 2022**

**Journal: Canadian Family Physician Record Number: 20**

| **1. Study Characteristics** | |
| --- | --- |
| **Item** | **Extracted Data** |
| 1.1 Author (Publication Year) | Varner et al., 2022 |
| 1.2 Country | Canada |
| 1.3 Location | Practical Sites: 2 hospital-affiliated academic family medicine clinics in Toronto, ONT |
| 1.4 Study Design | Cohort study |
| 1.5 Sample Size | 12 |
|  | |
| **2. Population Characteristics (Healthcare Providers Receiving POCUS Training)** | |
| **Item** | **Extracted Data** |
| 2.1 Age | NI |
| 2.2 Gender | NI |
| 2.3 Professional Role | Doctors: 100% (12/12) |
| 2.4 Work Experience (Duration) | NI |
| 2.5 Prior Ultrasound Training Experience | NI |
|  | |
| **3. Intervention Characteristics (According to ADDIE Training Model)** | |
| **Item** | **Extracted Data** |
| 3.1 Analysis | 1. Trainee Group:   The trainees were family physicians from two hospital-affiliated academic family medicine clinics in Toronto.   1. Needs Analysis: NI 2. Baseline Survey: None. 3. Impact on Instructional Design:   Since family physicians lacked experience in first-trimester POCUS and faced application barriers, the course design emphasized basic theoretical knowledge, equipment operation skills, and a large amount of practical training to help them master POCUS application in early pregnancy assessment. |
| 3.2 Design | 1. Training Objectives:    1. Skill goals: The primary goal was to enable family physicians to safely and accurately use POCUS to diagnose intrauterine pregnancy (IUP) and fetal cardiac activity (FCA) in first-trimester pregnancies, reducing reliance on radiologists for urgent scans. 2. Teaching Methods:   The curriculum consisted of a blended method using online learning materials, seminars, and hands-on training.   1. Training Content/Plan/Syllabus:   The Family Medicine Obstetrical Ultrasound (FaMOUS) course was designed based on the deliberate practice-mastery model and divided into three progressive phases, including e-learning of core materials, orientation to the ultrasound machine and techniques in seminars, and hands-on training. |
| 3.3 Development | 1. Instructors/Teachers:   7 Canadian Emergency Ultrasound Society (CEUS) instructors were responsible for teaching, with a student-to-instructor ratio of approximately 2:1;   1. Teaching Aids:    1. Devices: Portable and hand-held ultrasound devices were used;    2. Ultrasound models: 70 volunteers (10 of whom were pregnant); 2. Teaching Materials:   This course was modeled after the Canadian Emergency Ultrasound Society (CEUS) Emergency Department Echo course and certification process15, developed an interactive e-learning modules, ultrasound guides, and training manuals for first-trimester POCUS. These materials were designed to ensure that learners could access content at their own pace before attending the seminar and hands-on training. |
| 3.4 Implementation | 1. Time:   The total duration of the hands-on training workshop was 12 hours, 2 -hour seminar at the beginning for orientation, and the remaining 10 hours for hands-on training;   1. Location/Setting:   The training took place in two hospital -affiliated family medicine clinics in Toronto.   1. Participants:    1. Trainers: 7 CEUS instructors.    2. Trainees: 12 family physicians. 2. Execution Process:    1. Phase 1: Trainees first completed an e-learning module with a required 100% score.    2. Phase 2: The first 2 hours of the workshop seminar oriented learners to the ultrasound machine and specific techniques for image generation.    3. Phase 3: The remaining 10 hours of the workshop were dedicated to hands-on training with Canadian Emergency Ultrasound Society (CEUS) instructor supervision to complete the course certification process, with each person completing at least 60 successful supervised scans. |
| 3.5 Evaluation | 1. Summative Evaluation:    1. Purpose: To evaluate the use of POCUS by family physicians, including the proportion of use, usage indications, diagnostic accuracy, pregnancy outcomes, and emergency department visits.    2. Methods/Tools: Retrospective chart review was used to collect data, and the effectiveness of the training was assessed by comparing participants’ POCUS results with those from radiologist-interpreted ultrasound;    3. Timing: 6 months after the family physicians completed the FaMOUS course. |
|  | |
| **4. Outcome Characteristics** | |
| **Item** | **Extracted Data** |
| **4.1 Training Outcomes** |  |
| 4.1.1 Diagnostic Accuracy | 1. POCUS documenting intrauterine pregnancy (Compared with radiologist interpreted ultrasound):    1. Sensitivity: 91.3% (95% CI: 79.2% to 97.6%);    2. PPV (Positive Predictive Value): 100% (95% CI: 92.3% to 100%); 2. POCUS documenting the presence of fetal cardiac activity (Compared with radiologist interpreted ultrasound):    1. Sensitivity: 81.4% (95% CI: 66.6% to 91.6%);    2. Specificity: 100% (95% CI: 29.2% to 100%);    3. PPV (Positive Predictive Value): 100% (95% CI: 91.0% to 100%);    4. NPV (Negative predictive value): 27.3% (95% CI: 16.7% to 41.2%). |
| 4.1.2 Knowledge Acquisition | NI |
| 4.1.3 Skills Acquisition | NI |
| 4.1.4 Knowledge Retention | NI |
| 4.1.5 Practical Application | 1. The proportion of patients who completed POCUS assessment to the total number of pregnant women attending the two clinics during the same period (Total number of pregnant women, n=496): 11.3% (56/496); 2. Number of family doctors using POCUS to assess early pregnancy patients within 6 months of completion of training as a proportion of the total number of trained family doctors: 58.3% (7/12). |
| **4.2 Maternal and Neonatal Health Outcomes** (Including Physiological and Psychological Outcomes) | NI |
| **4.3 Health Economic Outcomes** | NI |

**JBI Qualitative Data Extraction Tool**

**Reviewer: XXX & XXX & XXX Date: 28^th^ February 2025**

**Author: Westerway Year: 2019**

**Journal: Australasian Society for Ultrasound in Medicine Record Number: 21**

| **1. Study Characteristics** | |
| --- | --- |
| **Item** | **Extracted Data** |
| 1.1 Author (Publication Year) | Westerway, 2019 |
| 1.2 Country | 1. Australia 2. Timor-Leste 3. Indonesia 4. Central Asia |
| 1.3 Location | 1. Australia Sites:   At city hospital, rural hospital and private hospital;   1. Overseas Sites:    1. Women’s clinic, Timor Leste;    2. Rural clinic, Indonesia;    3. Rural Hospital, Central Asia. |
| 1.4 Study Design | Cohort study |
| 1.5 Sample Size | 55  (Australia n=32, Overseas n=23) |
|  | |
| **2. Population Characteristics (Healthcare Providers Receiving POCUS Training)** | |
| **Item** | **Extracted Data** |
| 2.1 Age | NI |
| 2.2 Gender | NI |
| 2.3 Professional Role | 1. Doctors: 27.3% (15/55); 2. Midwives: 41.8% (23/55); 3. Nurses: 16.4% (9/55); 4. Radiographers: 14.5% (8/55). |
| 2.4 Work Experience (Duration) | NI |
| 2.5 Prior Ultrasound Training Experience | 1. Doctors: None (0/15); 2. Midwives: None (0/23); 3. Nurses: None (0/9); 4. Radiographers: None (0/8). |
|  | |
| **3. Intervention Characteristics (According to ADDIE Training Model)** | |
| **Item** | **Extracted Data** |
| 3.1 Analysis | 1. Trainee Group:   Healthcare learners with no prior ultrasound experience, among them, and 23 had English as a second language (EASL).   1. Needs Analysis:   The need for healthcare providers in rural and remote areas to learn the skills to perform obstetric ultrasound.   1. Baseline Survey:   A pre-course multiple-choice test was used to determine participants' prior ultrasound knowledge.   1. Impact on Instructional Design:   Since participants had no prior ultrasound experience, the training focused on basic knowledge and skills. Also, considering the language barrier of English as a second language (EASL) participants, interpreters were provided during the pre-course test, which could have influenced the overall instructional design to be more inclusive. |
| 3.2 Design | 1. Training Objectives:    1. Knowledge goals: Understand how ultrasound works, ultrasound machine controls, and essential knowledge for performing a limited-scope pregnancy ultrasound.    2. Skill goals: Obtain and optimize an ultrasound image and perform basic fetal biometry.    3. Attitude goals: Develop confidence in scanning skills. 2. Training Methods:   Combined didactic lectures, simulative teaching methods for probe manipulation, and hands-on practice.   1. Training Content/Plan/Syllabus:   It started with basic knowledge about ultrasound and machine controls, followed by probe manipulation teaching, then moved on to clinical components like identifying fetal and uterine anatomy and performing measurements. The practical scanning sessions accounted for a significant portion of the course hours (ranging from 60-100% of total course hours), indicating a heavy emphasis on hands-on skills development. |
| 3.3 Development | 1. Instructors/Teachers:   The lead tutor conducted all didactic lectures, and skill sets for every course, and experienced obstetric ultrasound tutors assisted with hands-on sessions. The instructor-to-trainee ratio varied from 1:1 to 1:6.   1. Teaching Aids:    1. Devices: Ultrasound machines, commercial or homemade phantoms.    2. Ultrasound model: Volunteer pregnant women in the third trimester of pregnancy were used as teaching aids. 2. Teaching Materials:   Teaching materials included lecture notes for didactic lectures, and a checklist for practical scanning assessments. |
| 3.4 Implementation | 1. Time:   The study was conducted 18 months. Course duration ranged from 4 to 18 hours.   1. Location/Setting:   Three courses were conducted in Australia and other three overseas locations.   1. Participants:    1. Trainers: A lead tutor and experienced obstetric ultrasound tutors.    2. Trainees: 55 healthcare providers with no prior ultrasound experience. 2. Execution Process:   First, participants took a pre-course multiple-choice test to know their basic knowledge. Then, they received didactic lectures on ultrasound theory and machine controls. After that, they learned probe manipulation through simulative teaching. Next, they practiced using machine controls and defined skill sets on phantoms and pregnant women under the guidance of experienced tutors. Finally, they took a post-course multiple-choice test and completed a course evaluation. |
| 3.5 Evaluation | 1. Summative Evaluation:    1. Purpose: Assess participants' understanding of the taught content, their ability to perform scanning tasks, and their satisfaction with the course, as well as the overall effectiveness of the training.    2. Methods/Tools: Post-course multiple-choice test identical to the pre-course test and course evaluation forms.    3. Timing: At the end of the training course. 2. Follow-up:    1. Purpose: Assess skill retention, application of learned knowledge in practice, confidence in scanning skills, and the impact on patient outcomes.    2. Participant: A total number of follow-up participants are 31, with the sample size of the Australia group is 21 and the overseas group is 10.    3. Methods/Tools: 3. Expanded pre-course multiple-choice test. 4. Image-commenting task. 5. Practical scanning assessment of a third - trimester pregnancy.    1. Timing: 6 to 11 months after the completion of the initial course (21 participants from Australian courses at 6 months and 10 from overseas courses at 11 months). |
|  | |
| **4. Outcome Characteristics** | |
| **Item** | **Extracted Data** |
| **4.1 Training Outcomes** |  |
| 4.1.1 Diagnostic Accuracy | NI |
| 4.1.2 Knowledge Acquisition | 1. Understanding of knowledge: 100% (55/55). |
| 4.1.3 Skills Acquisition | 1. Targets achievement rate (being able to independently obtain and optimise an ultrasound image with depth, focus and gain controls and to correctly move the ultrasound Probe): 100% (55/55); 2. Of the 10 overseas personnel who participated in the follow-up:    1. Pass rate of visual assessment: 80.0% (8/10);    2. Successfully completed the practical test with minimal prompting: 60.0% (6/10). |
| 4.1.4 Knowledge Retention | 1. Correctly identified the best image examples among all 31 participants who took part in the follow-up: 67.7% (21/31); 2. Correctly identified fetal anatomy among all 21 Australian participants who took part in the follow-up: 100% (21/21). |
| 4.1.5 Practical Application | 1. Clinical scan (commitment to continue scanning on their return to their clinics): 100% (55/55). |
| **4.2 Maternal and Neonatal Health Outcomes** (Including Physiological and Psychological Outcomes) | NI |
| **4.3 Health Economic Outcomes** | NI |

**JBI Qualitative Data Extraction Tool**

**Reviewer: XXX & XXX & XXX Date: 28^th^ February 2025**

**Author: Lee et al. Year: 2017**

**Journal: World Journal of Emergency Medicine Record Number: 22**

| **1. Study Characteristics** | |
| --- | --- |
| **Item** | **Extracted Data** |
| 1.1 Author (Publication Year) | Lee et al., 2017 |
| 1.2 Country | Indonesia |
| 1.3 Location | Trial Sites: Public health care clinics |
| 1.4 Study Design | Cross-sectional study |
| 1.5 Sample Size | Total: 41 |
|  | |
| **2. Population Characteristics (Healthcare Providers Receiving POCUS Training)** | |
| **Item** | **Extracted Data** |
| 2.1 Age | NI |
| 2.2 Gender | NI |
| 2.3 Professional Role | Doctors: 100% (41/41) |
| 2.4 Work Experience (Duration) | NI |
| 2.5 Prior Ultrasound Training Experience | 1. Had no prior ultrasound experience: 53.7% (22/41); 2. Had only observed the use of ultrasound: 43.9% (18/41); 3. Had taken a prior ultrasound course: 2.4% (1/41). |
|  | |
| **3. Intervention Characteristics (According to ADDIE Training Model)** | |
| **Item** | **Extracted Data** |
| 3.1 Analysis | 1. Trainee Group:   Indonesian health care practitioners from public clinics in Bandung, Indonesia, including general practitioners.   1. Needs Analysis:   Less than 50% of general practitioners in the country offer sonography. It’s necessary to learn POCUS which could help identify pathology in patients.   1. Baseline Survey:    1. A pre-course examination was conducted before the training. It included questions about prior ultrasound knowledge, familiarity with sonography, the ability to access an ultrasound machine, and primary language.    2. Participants were also asked to rate their comfort level using ultrasound on a scale from 0 to 5 and took a 32-question multiple-choice exam to test their knowledge related to the course topics. 2. Impact on Instructional Design:   Since most participants had little to no prior ultrasound experience, the curriculum started with basic ultrasound knowledge such as physics, knobology, probe manipulation, and scanning techniques. |
| 3.2 Design | 1. Training Objectives:    1. Knowledge goals: Understand ultrasound physics, knobology, and the knowledge related to different ultrasound applications.    2. Skill goals: Be able to perform ultrasound scans, manipulate the probe correctly, acquire and interpret ultrasound images for different anatomical structures and pathologies.    3. Attitude goals: Increase participants' confidence and positive attitude in using ultrasound in clinical practice. 2. Training Methods:   Combined lecture presentations and hands on training.   1. Training Content/Plan/Syllabus:   It began with basic ultrasound concepts and then moved on to specific organ systems and applications. The first session covered basic ultrasound, and subsequent sessions focused on pulmonary, cardiac, abdominal, obstetric and gynecologic ultrasound. And hands-on training accounted for a significant portion of the course time. |
| 3.3 Development | 1. Instructors/Teachers:   The instructors were American first-year medical students. They had completed a first year of medical education with ten hours of supervised hands-on ultrasound training and pre-session podcasts on eight organ systems.   1. Teaching Aids:    1. Devices: SonoSite Nanomaxx ultrasound machines were used.    2. Ultrasound model: Healthy models were used for practice. 2. Teaching Materials:   Teaching materials included course materials in English and Indonesian. The course materials were designed to cover the 38 ultrasound milestones in the curriculum. |
| 3.4 Implementation | 1. Time:   The training course lasted 4 weeks and consisted of six sessions. Each session started with a 30-minute lecture followed by 2 hours of hands-on training, with a 10-minute break during the hands-on part.   1. Location/Setting:    1. At the public clinics.    2. A related medical facility in Bandung where the participants worked. 2. Participants:    1. Trainers: American first-year medical students.    2. Trainees: 41 Indonesian health care practitioners. 3. Execution Process:   First, participants took a pre-course examination. Then, they attended 6 sessions of the training course with lectures and hands on training and took multiple choice quizzes after each session. At the end of the course, they took a post-test identical to the pre-test, a practical exam, and completed post-course and "Intent to Use" surveys. |
| 3.5 Evaluation | 1. Formative Evaluation:    1. Purpose: To assess participants’ comprehension of the content taught in each session.    2. Method: A multiple-choice quiz.    3. Timing: At the end of each session. 2. Summative Evaluation:    1. Purpose: Assessing participants' knowledge acquisition, practical skills, and change in comfort level with ultrasound.    2. Methods/Tools: 3. Pre-and post-course written exams. 4. Practical exam. 5. Post-course surveys.    1. Timing: The pre-course exams were taken before the training, and the post-course exams and surveys were taken at the end of the 4-week training. 6. Follow-up:    1. Purpose: Re-evaluate the long-term retention of ultrasound knowledge, the frequency of POCUS uses in current practice, and the accuracy of POCUS in identifying pathology.    2. Methods/Tools: 7. Repeat examinations. 8. Surveys.    1. Timing: Scheduled for one year after the course. |
|  | |
| **4. Outcome Characteristics** | |
| **Item** | **Extracted Data** |
| **4.1 Training Outcomes** |  |
| 4.1.1 Diagnostic Accuracy | NI |
| 4.1.2 Knowledge Acquisition | 1. Average post-course exam score: 82.1%. 2. Post-course exam passes rate (score > 75%): 92.7% (38/41). 3. Comparison of pre-course and post-course final exam score:    1. Pre-course scores: 35.6% ± 13.9%.    2. Post-course score: 82.1% ± 11.5%.    3. Two-sample t-test: t= –16.5, P<0.001. |
| 4.1.3 Skills Acquisition | 1. Comfort levels comparison in abdominal ultrasound:    1. Pre-course abdominal-Mean (SD): 1.78 (1.97);    2. Post-course abdominal-Mean (SD): 3.95 (0.86);    3. The degrees of freedom (df): 54.9.    4. The two-sample t-test: –6.46, and the *P* <0.001. 2. Average practical score at the completion of course (SD): 83.2% (0.145). 3. Passing rate of class practical score at the completion of the course (score > 75.0%): 82.9% (34/41). |
| 4.1.4 Knowledge Retention | NI |
| 4.1.5 Practical Application | 1. Comparison of the training methods’ effectiveness based on participants’ 1-5 scale ratings on course aspects:    1. Watching: 4.60 ± 0.63    2. Practicing the scans: 4.28 ± 1.21    3. Attending class lecture: 4.23 ± 0.80    4. Studying online materials: 3.54 ± 0.98    5. Using other study tools: 3.50 ± 0.92    6. Using the study guides: 3.42 ± 1.36 2. Frequency of obstetrics scans: 7.8 times per week (7.8 ± 12.9) |
| **4.2 Maternal and Neonatal Health Outcomes** (Including Physiological and Psychological Outcomes) | NI |
| **4.3** **Health Economic Outcomes** | “This ultrasound training course serves as a cost-effective method to increase exposure of general practitioners to POCUS.” |

**JBI Qualitative Data Extraction Tool**

**Reviewer: XXX & XXX & XXX Date: 28^th^ February 2025**

**Author: Nathan et al. Year: 2017**

**Journal: Current Problems in Diagnostic Radiology Record Number: 23**

| **1. Study Characteristics** | |
| --- | --- |
| **Item** | **Extracted Data** |
| 1.1 Author (Publication Year) | Nathan et al., 2017 |
| 1.2 Country | 1. Congo 2. Guatemala 3. Kenya 4. Pakistan 5. Zambia |
| 1.3 Location | Pilot sites:  Karawa, Chimaltenango, Eldoret, Karachi, Lusaka |
| 1.4 Study Design | Cross-sectional study |
| 1.5 Sample Size | 41  (Congo n=6, Guatemala n=10, Kenya n=12, Pakistan n=4, Zambia n=9) |
|  | |
| **2. Population Characteristics (Healthcare Providers Receiving POCUS Training)** | |
| **Item** | **Extracted Data** |
| 2.1 Age | NI |
| 2.2 Gender | (1) Male: 39.0% (16/41)  (2) Female: 61.0% (25/41) |
| 2.3 Professional Role | (1) Nurse: 43.9% (18/41)  (2) Midwives: 14.6% (6/41)  (3) Medical Officer: 24.4% (10/41)  (4) Radiographer: 17.1% (7/41) |
| 2.4 Work Experience (Duration) | 1. Congo (Mean): 7.3 years (Range 4-12) 2. Nurse: 100% (6/6) 3. Guatemala (Mean): 10.3 years (Range 0.8-23) 4. Nurse: 60.0% (6/10) 5. Medical Officer: 40.0% (4/10) 6. Kenya (Mean): 2.8 years (Range 0.7-4) 7. Nurse: 50.0% (6/12) 8. Medical Officer: 50.0% (6/12) 9. Pakistan (Mean): 0.5 years (Range 0-1.5) 10. Midwives: 100.0% (4/4) 11. Zambia (Mean): 16.6 years (Range 2-39) 12. Midwives: 22.2% (2/9) 13. Radiographer: 77.8% (7/9) |
| 2.5 Prior Ultrasound Training Experience | (1) Nurse: None (0/18)  (2) Midwives: None (0/6)  (3) Medical Officer: None (0/10)  (4) Radiographers: None (0/7) |
|  | |
| **3. Intervention Characteristics (According to ADDIE Training Model)** | |
| **Item** | **Extracted Data** |
| 3.1 Analysis | 1. Trainee Group:   Healthcare workers from 5 low-and middle-income countries (LMIC).   1. Needs Analysis:   Since trained sonographers were unavailable for rural health centers, there was a need for local healthcare workers to learn basic obstetrics ultrasound for high-risk pregnancy screening.   1. Baseline Survey: None. 2. Impact on Instructional Design:   The training focused on basic obstetrics ultrasound skills. The content prioritized essential scanning parameters and high-risk pregnancy detection. |
| 3.2 Design | 1. Training Objectives:    1. Knowledge goals: Understand basic obstetrics ultrasound knowledge related to high-risk pregnancy screening.    2. Skill goals: Be able to perform a basic obstetrics ultrasound examination.    3. Attitude goals: Develop confidence in using obstetrics ultrasound for high-risk pregnancy screening in a limited-resource setting. 2. Training Methods:   The training course was a combination of didactic sessions and supervised hands-on training.   1. Training Content/Plan/Syllabus:   The hands-on training was rudimentary but gradually included all aspects of a basic examination. Approximately one-third of the course time was spent in didactic sessions, and two-thirds in supervised hands-on training. |
| 3.3 Development | 1. Instructors/Teachers:   The course was conducted at the study sites by practitioners experienced in ultrasound training. The lead trainer at each site was assisted by local practitioners with substantial ultrasound experience.   1. Teaching Aids: GE LOGIQ e systems. 2. Teaching Materials:    1. Basic obstetrics ultrasound, covering specific parameters such as fetal biometry, placental position, and cervical length.    2. Scanning worksheets were developed for documenting ultrasound findings and measurements.    3. Quality control (QC) website. |
| 3.4 Implementation | 1. Time:   The pretrial training period consisted of a 2-week intensive course in basic obstetrics ultrasound followed by a 12-week pilot phase.   1. Location/Setting:   The examinations during the pilot phase were conducted at intervention health centers in these regions.   1. Participants:    1. Trainers: Experienced ultrasound practitioners at each site, including lead trainers and local assistants.    2. Trainees: 41 healthcare workers from the 5 countries. 2. Execution Process:   First, the 41 trainees attended a 2-week basic obstetrics ultrasound course. Then, during the 12-week pilot phase, they performed ultrasound examinations on pregnant patients at intervention health centers. They saved relevant images and filled out obstetrics worksheets. All examinations were reviewed on a quality control website, and trainees had regular meetings with local trainers. Targeted remedial training was provided based on performance evaluations. |
| 3.5 Evaluation | 1. Formative Evaluation:    1. Purpose: Monitor trainees' progress during the training and pilot phase, identify areas for improvement, and provide timely feedback.    2. Methods/Tools: 2. Monthly practical examinations. 3. Scan reviews on the QC website. 4. Regular meetings with local trainers.    1. Timing: The daily schedule for each trainee is to complete 2 supervised ultrasound examinations. Then, monthly practical examinations during the 12-week pilot phase, and continuous scan reviews and meetings throughout the training and pilot phase. 5. Summative Evaluation:    1. Purpose: Evaluate the overall effectiveness of the training program in enabling trainees to perform basic obstetrics ultrasound for high-risk pregnancy screening.    2. Methods/Tools: 6. Written exam. 7. Practical exam.    1. Timing: Written and practical examinations at the end of the 2-week course, and a practical examination at the end of the 12-week pilot phase. |
|  | |
| **4. Outcome Characteristics** | |
| **Item** | **Extracted Data** |
| **4.1 Training Outcomes** |  |
| 4.1.1 Diagnostic Accuracy | 1. During the 12-week pilot phase, participants performed 3,801 examinations. The concordance between trainee and reviewer ultrasound diagnosis: 99.4% (3778/3801); 2. Secondary images quality control (QC) review accuracy (Kappa): 0.33 (Range: 0.3-0.4). |
| 4.1.2 Knowledge Acquisition | 1. Passing rate of written test (score ≥ 75%):    1. First test: 95.1% (39/41);    2. Two trainees passed after a second attempt one week later, resulting in a final pass rate of 100% (41/41). |
| 4.1.3 Skills Acquisition | 1. Mean scanning skills scores increased from 78% on the first test to 92% on the fourth test; 2. Passing rate of scanning skill test:    1. A week after training: 87.8% (36/41);    2. After 12 weeks pilot phase: 97.6% (40/41); 3. During the 12-week pilot phase, participants performed 32,480 images. The overall reviewers satisfactory of images interpretation: 94.8% (30799/32480). |
| 4.1.4 Knowledge Retention | NI |
| 4.1.5 Practical Application | NI |
| **4.2 Maternal and Neonatal Health Outcomes** (Including Physiological and Psychological Outcomes) | NI |
| **4.3 Health Economic Outcomes** | NI |

**JBI Qualitative Data Extraction Tool**

**Reviewer: XXX & XXX & XXX Date: 28^th^ February 2025**

**Author: Shah et al. Year: 2009**

**Journal: BMC International Health and Human Rights Record Number: 24**

| **1. Study Characteristics** | |
| --- | --- |
| **Item** | **Extracted Data** |
| 1.1 Author (Publication Year) | Shah et al., 2009 |
| 1.2 Country | Rwanda |
| 1.3 Location | Plots Sites: At two rural hospitals located in the villages of Kirehe and Rwinkwavu in Eastern Province. |
| 1.4 Study Design | Cross-sectional study |
| 1.5 Sample Size | NI |
|  | |
| **2. Population Characteristics (Healthcare Providers Receiving POCUS Training)** | |
| **Item** | **Extracted Data** |
| 2.1 Age | NI |
| 2.2 Gender | NI |
| 2.3 Professional Role | Doctors |
| 2.4 Work Experience (Duration) | NI |
| 2.5 Prior Ultrasound Training Experience | NI |
|  | |
| **3. Intervention Characteristics (According to ADDIE Training Model)** | |
| **Item** | **Extracted Data** |
| 3.1 Analysis | 1. Trainee Group:   Interested physician staff at two rural hospitals in Rwanda's Eastern Province.   1. Baseline Survey: NI. 2. Need Analysis: NI. 3. Impact on Instructional Design: NI. |
| 3.2 Design | 1. Training Objectives:    1. Knowledge goals: Trainees were expected to understand the principles of ultrasound physics, operation of the ultrasound machine, and potential uses of ultrasound. They also needed to learn about different ultrasound applications for various medical conditions.    2. Skill goals: Be able to perform different types of ultrasound exams (includes obstetric), and accurately interpret the images, and train physicians to use ultrasound in real-world clinical scenarios.    3. Attitude goals: Develop confidence in using ultrasound as a diagnostic tool in a rural, resource-limited setting and integrate it into daily clinical practice. 2. Training Methods:   The training curriculum combined lectures with practical hands-on scanning sessions.   1. Training Content/Plan/Syllabus:   It started with an introduction to ultrasound physics, machine operation, and recording instructions. Then, it covered specific ultrasound applications for different medical conditions. The curriculum weighted more on practical skills through hands on scanning sessions. |
| 3.3 Development | 1. Instructors/Teachers:   The main instructor was a fourth-year emergency medicine resident with prior ultrasound experience and relevant certification. A cardiologist from the United States gave the cardiac ultrasound lecture;   1. Teaching Aids:   A SonoSite Micromaxx was used for training and post-training clinical studies. |
| 3.4 Implementation | 1. Time:   The training lasted 11 weeks. The initial training period is 9-week training and the post-training period is the followed 11-week period after the departure of foreign staff.   1. Location/Setting:   The training took place at two rural hospitals in Rwanda's Eastern Province, in the villages of Kirehe and Rwinkwavu.   1. Participants:    1. Trainers: A fourth-year emergency medicine resident and a cardiologist.    2. Trainees: Interested physician staff at the two hospitals. 2. Execution Process:   The training started with lectures on ultrasound physics, machine operation, and basic applications. Then, there were hands on scanning sessions for 9 weeks. The instructor accompanied local physician staff on daily ward rounds to demonstrate the integration of bedside ultrasound into clinical practice. After the training (during post-training period), local providers continued to use the ultrasound and record their scans and interpretations. |
| 3.5 Evaluation | 1. Summative Evaluation:    1. Purpose: Evaluate the overall effectiveness of the training in terms of trainees' ability to accurately interpret ultrasound images and its impact on patient management.    2. Methods/Tools: 2. Data sheets were collected for each ultrasound scan, recording patient demographics, scan type, and whether the scan changed the patient management plan. 3. Hard copies of scans from the post-training period were sent to the US for blinded review by an ultrasound-trained emergency physician to assess accuracy and scan quality.    1. Timing: After the initial training period and during the post-training period. 4. Follow-up:    1. Purpose: Assess the long-term sustainability of the ultrasound program and the diagnostic accuracy of local health care providers months to years after training.    2. Methods/Tools: Longitudinal evaluation of the training program, and Rwandese staff physicians emailing interesting or concerning cases for quality assurance and interpretation assistance. |
|  | |
| **4. Outcome Characteristics** | |
| **Item** | **Extracted Data** |
| **4.1 Training Outcomes** |  |
| 4.1.1 Diagnostic Accuracy | 1. 97 valid scans were performed in the post-training, the scan accuracy:    1. True positive results: 78.4% (76/97).    2. True negative results: 18.6% (18/97).    3. False positive results: 3.1% (3/97).    4. False negative results: None (0/97). 2. The concordance rate of interpretation (between the Rwandese physicians and the ultrasound trained physicians doing quality review): 96%. |
| 4.1.2 Knowledge Acquisition | NI |
| 4.1.3 Skills Acquisition | NI |
| 4.1.4 Knowledge Retention | NI |
| 4.1.5 Practical Application | 1. Of the 242 patients who underwent ultrasound, the use of the ultrasound changed patient management in 42.6% (103/242) of cases; 2. Of the 345 ultrasound scans performed during the study, obstetrics is the most common ultrasound exam performed during routine clinical care: 29.6% (102/345); 3. After the completion of the ultrasound training course and the departure of the foreign instructor, 245 new ultrasound scans were performed by local staff in 11 weeks. |
| **4.2 Maternal and Neonatal Health Outcomes** (Including Physiological and Psychological Outcomes) | NI |
| **4.3 Health Economic Outcomes** | NI |

**JBI Qualitative Data Extraction Tool**

**Reviewer: XXX & XXX & XXX Date: 28^th^ February 2025**

**Author: Shokoohi et al. Year: 2019**

**Journal: Ultrasound in Medicine & Biology Record Number: 25**

| **1. Study Characteristics** | |
| --- | --- |
| **Item** | **Extracted Data** |
| 1.1 Author (Publication Year) | Shokoohi et al., 2019 |
| 1.2 Country | 1. Training place: United States of America 2. Pilot places: Malawi, United Republic of Tanzania, and Uganda |
| 1.3 Location | Pilot Sites: Academic institutions |
| 1.4 Study Design | Cross-sectional study |
| 1.5 Sample Size | 49 |
|  | |
| **2. Population Characteristics (Healthcare Providers Receiving POCUS Training)** | |
| **Item** | **Extracted Data** |
| 2.1 Age | NI |
| 2.2 Gender | NI |
| 2.3 Professional Role | (1) Midwives: 14.3% (7/49) |
| 2.4 Work Experience (Duration) | 1. Years from Completing Residency:    1. <5 Years: 55.1% (27/49).    2. 6-10 Years: 10.2% (5/49).    3. >10 Years: 34.7% (17/49). 2. Years of Providing Service at the Host Country:    1. 2016–2017: 46.9% (23/49).    2. 2015–2016: 26.5% (13/49).    3. 2014–2015: 24.5% (12/49).    4. 2013–2014: 22.4% (11/49).   Note: “Some of the clinical educators served in multiple years for which the percentage is greater than 100%.” |
| 2.5 Prior Ultrasound Training Experience | 1. Number of scans before Global Health Service Partnership:    1. None: 42.9% (21/49)    2. 20-40: 28.6% (14/49)    3. 40-100: 4.1% (2/49)    4. 100-1000: 16.3% (8/49)    5. >1000: 8.2% (4/49) |
|  | |
| **3. Intervention Characteristics (According to ADDIE Training Model)** | |
| **Item** | **Extracted Data** |
| 3.1 Analysis | 1. Trainee Group:   The trainee group consists Global Health Service Partnership (GHSP) clinical educators.   1. Needs Analysis: NI. 2. Baseline Survey:   The baseline information collected included the number of scans the clinical educators had performed before the Global Health Service Partnership (GHSP) training. Most of the workers had no prior experience with independent ultrasound scanning.   1. Impact on Instructional Design:   The content focused on applications relevant to the resource-limited settings. |
| 3.2 Design | 1. Training Objectives:    1. Knowledge goals: Understand the principles, applications and indications of POCUS.    2. Skill goals: Be able to perform POCUS examinations independently, and interpret the images accurately.    3. Attitude goals: Develop confidence in using POCUS in clinical practice, and be willing to integrate it into patient care and teaching activities. 2. Training Methods:    1. A pre-departure training, with lectures and hands on sessions.    2. An on-site hands-on training.    3. Self-study online modules and educational materials were provided, along with online feedback on transmitted images. 3. Training Content/Plan/Syllabus:   The pre-departure training introduced the basic concepts and applications of POCUS. The on-site training built on this foundation, covering more in-depth topics like machine operation. Sites with midwives and obstetricians received focused training on gynecologic ultrasound and first-and third-trimester ultrasound, as well as other relevant topics. The emphasis was on practical skills, with hands-on training both in the classroom and at the bedside. |
| 3.3 Development | 1. Instructors/Teachers:   The instructors included two authors who had relevant experience in ultrasound. The volunteer-to-instructor ratio during the hands-on training in Washington, DC, was 3:1 to 4:1.   1. Teaching Aids:    1. Devices: Handheld ultrasound.    2. Data collection: An electronic platform, REDCap. 2. Teaching Materials: Clinical educators were provided with online modules, educational materials. |
| 3.4 Implementation | 1. Time:    1. The pre-departure training took place over 2 days during the Global Health Service Partnership (GHSP) orientation in Washington, DC.    2. The on-site training occurred 2 to 4 months after the clinical educators arrived in the host countries. The overall training period was part of the 1-year deployment of the clinical educators. 2. Location/Setting:    1. The pre-departure training was in Washington, DC.    2. The on-site training was conducted at academic institutions in Malawi, Tanzania, and Uganda.    3. The clinical practice and use of POCUS mainly occurred in hospitals and clinics in these host countries. 3. Participants:    1. Trainers: Experienced in ultrasound, including two authors.    2. Trainees: 49 Global Health Service Partnership (GHSP) clinical educators, consisting of physicians and midwives, with various medical specialties. 4. Execution Process:   First, clinical educators received pre-departure training in Washington, DC, with lectures on US application and hands-on POCUS training. After arriving in the host countries, they had on-site training that included reviewing concepts, learning machine operation, and practicing different ultrasound exams. They also had access to online modules and received online feedback on their scans. The educators were expected to use POCUS in their clinical and teaching activities during their 1-year deployment. |
| 3.5 Evaluation | 1. Summative Evaluation:    1. Purpose: Evaluate the overall effectiveness of the POCUS training program, including its impact on knowledge, skills, and the use of POCUS in clinical practice.    2. Methods/Tools: A 35-question cross-sectional survey was used to collect data on the educators’ medical experience, POCUS use, training adequacy, and barriers.    3. Timing: The survey was conducted during the 1-year deployment of the clinical educators, with the time between training and survey completion being variable. |
|  | |
| **4. Outcome Characteristics** | |
| **Item** | **Extracted Data** |
| **4.1 Training Outcomes** |  |
| 4.1.1 Diagnostic Accuracy | NI |
| 4.1.2 Knowledge Acquisition | NI |
| 4.1.3 Skills Acquisition | 1. Of the 41 people who responded to the question “level of confidence in the continuity of ultrasound use at their institution after leaving”:    1. Very confident: 29.3% (12/41)    2. Somewhat confident: 22.0% (9/41)    3. Neutral: 17.1% (7/41)    4. Not very confident: 24.4% (10/41)    5. Not at all confident: 7.3% (3/41) |
| 4.1.4 Knowledge Retention | NI |
| 4.1.5 Practical Application | 1. Frequency in clinical sites:    1. More than once a week: 57.1% (28/49)    2. Every day: 36.7% (18/49)    3. More than once a week but less than daily: 20.4% (10/49)    4. More than once a month: 24.5% (12/49)    5. Less than once a month: 10.2% (5/49) 2. Clinical application of POCUS:    1. 69.4% of clinical educators (34/49) reported using POCUS often in aiding diagnosis    2. 44.9% (22/49) used POCUS in determining treatment    3. 30.6% (15/49) used POCUS in monitoring the clinical course of patients    4. 16.3% (8/49) reported frequent use of POCUS for the procedural applications 3. 61.2% (30/49) of clinical educators believed POCUS should be used to examine the second- and third-trimester obstetrics exams; and 42.9% (21/49) clinical educators mentioned the gynecologic and first-trimester obstetric exams. |
| **4.2 Maternal and Neonatal Health Outcomes** (Including Physiological and Psychological Outcomes) | NI |
| **4.3 Health Economic Outcomes** | NI |

**JBI Qualitative Data Extraction Tool**

**Reviewer: XXX & XXX & XXX Date: 28^th^ February 2025**

**Author: Vinayak et al. Year: 2017**

**Journal: Ultrasound in Medicine & Biology Record Number: 26**

| **1. Study Characteristics** | |
| --- | --- |
| **Item** | **Extracted Data** |
| 1.1 Author (Publication Year) | Vinayak et al., 2017 |
| 1.2 Country | Kenya |
| 1.3 Location | Pilot Sites: Aga Kahn University Hospital (the main study center at the hospital);  Provenance of Midwives: 3 satellite clinics were 20-, 120- and 400-km away from the main study center at the hospital. |
| 1.4 Study Design | Cross-sectional study |
| 1.5 Sample Size | 3 |
|  | |
| **2. Population Characteristics (Healthcare Providers Receiving POCUS Training)** | |
| **Item** | **Extracted Data** |
| 2.1 Age | NI |
| 2.2 Gender | NI |
| 2.3 Professional Role | Midwives: 100% (3/3) |
| 2.4 Work Experience (Duration) | <3 years: 100% (3/3) |
| 2.5 Prior Ultrasound Training Experience | None (0/3) |
|  | |
| **3. Intervention Characteristics (According to ADDIE Training Model)** | |
| **Item** | **Extracted Data** |
| 3.1 Analysis | 1. Trainee Group:   Midwives with less than 3 years of midwifery experience and no prior exposure to ultrasound practice. They were selected from three satellite centers located 20, 120 and 400 km away from the main study center, representing short, medium, and long-distance sites.   1. Needs Analysis:   Midwives lack basic obstetric ultrasound scanning skills and need to learn how to perform obstetric examinations using POCUS.   1. Baseline Survey: NI. 2. Impact on Instructional Design:   Since the midwives had no prior ultrasound knowledge, the content priority was given to basic obstetric ultrasound applications and image interpretation relevant to identifying high-risk pregnancies. |
| 3.2 Design | 1. Training Objectives:    1. Knowledge goals: Understand the general principles of ultrasound, ultrasound physics, and obstetric-specific ultrasound knowledge.    2. Skill goals: Determine the accuracy of images and reports generated by trained midwives performing basic obstetric ultrasound examinations at our satellite sites.    3. Attitude goals: Develop confidence in using ultrasound in their practice and a positive attitude towards teamwork with radiologists for accurate diagnosis. 2. Training Methods:   The training combined e-learning and in-person training, and the in-person training includes lectures, hands-on practical experience, and observation of scans by qualified sonographers.   1. Training Content/Plan/Syllabus:   It started with an online e-learning module that the midwives had to complete and pass a test on, and the e-learning module covered basic knowledge. Then, they participated in an in-person training program, the in-person training began with an introduction to ultrasound equipment and knobology, followed by hands-on scanning of phantoms, and finally independent scanning. |
| 3.3 Development | 1. Instructors/Teachers:   The instructors included experienced sonographers.   1. Teaching Aids:    1. VISIQ tablet-sized ultrasound scanner (Philips Ultrasound, Inc) as the main device for training and patient scanning.    2. A cell phone with a modem was used for transmitting images and reports.    3. Philips Connected Care (CCC) software was used as an electronic platform for teleradiology and remote reporting. 2. Teaching Materials:    1. The e-learning module was developed by the ultrasound manufacturer (Philips Medical Solutions);    2. PowerPoint presentations used for in-person lectures. |
| 3.4 Implementation | 1. Time:   The training period was just over 1 month. Each day of the 4-week in-person training started with a 1-hour lecture, followed by 6 hours of practical hands-on work, and ended with another 1-hour lecture in the evening. In week 5, the principal investigator assessed the midwives, and they received ultrasonography certification.   1. Location/Setting:    1. The e-learning part of the training could be completed at the midwives' respective satellite centers.    2. The in-person training was carried out at the main Aga Khan University Hospital.    3. Patient scanning took place at the three satellite antenatal clinics, and image and report validation occurred at the main hospital. 2. Participants:    1. Trainers: Experienced sonographers at the main study center.    2. Trainees: 3 midwives. 3. Execution Process:   First, midwives completed an e-learning module and passed a test, which covers the basic theoretical knowledge of ultrasound. Then, they underwent in-person training at the main center, which included lectures and hands-on practice. After training, they returned to their satellite clinics, selected eligible patients, obtained informed consent, performed ultrasound examinations following a strict protocol, acquired images, generated provisional reports, and uploaded everything to the Philips Connected Care (CCC) software. Radiologists at the main hospital reviewed and validated the images and reports and provided feedback. |
| 3.5 Evaluation | 1. Formative Evaluation:    1. Purpose: Assess the midwives' progress during training to determine when they were ready for independent practice.    2. Methods/Tools: 2. An e-learning module. 3. Continuous assessment during the training period, including observing midwives' practical skills development and collect feedback and questions from trainees.    1. Timing: 4. Complete the electronic module before the training begins. 5. Feedback and matters arising were addressed in week 3. 6. Summative Evaluation:    1. Purpose: Evaluate the overall effectiveness of the training program, including image and report accuracy, scanner performance, and the teleradiology system.    2. Methods/Tools: 7. Comparing midwives' image interpretations and reports with those of experienced radiologists, analyzing transmission times and image quality. 8. Conducting a patient satisfaction survey.    1. Timing: Throughout the training period, with the final assessment at the end of week 4 and additional assessment in week 5. |
|  | |
| **4. Outcome Characteristics** | |
| **Item** | **Extracted Data** |
| **4.1 Training Outcomes** |  |
| 4.1.1 Diagnostic Accuracy | 1. Of the 271 ultrasounds performed by midwives, the scan accuracy (corresponding measurements) rate: 99.6% (270/271); 2. Of the scans completed by the midwife, 20 were defined as high-risk pregnancies with an accuracy of 90.0% (18/20). |
| 4.1.2 Knowledge Acquisition | 1. Passing rate of the online e-module examination for midwives: 100% (3/3). |
| 4.1.3 Skills Acquisition | 1. The time it takes the midwife to complete a scan has been reduced from the initial 20 minutes to 10 minutes. |
| 4.1.4 Knowledge Retention | NI |
| 4.1.5 Practical Application | 1. Flow turnaround time (The time from patients receiving ultrasound to confirmation of their ultrasound report) was reduced from 35 minutes to 25 minutes. |
| **4.2 Maternal and Neonatal Health Outcomes** (Including Physiological and Psychological Outcomes) | 1. After completing the scan, feedback was received from a total of 246 pregnant women, which shows:    1. All of them 100% (246/246) had a better antenatal visit experience and increased confidence in the delivery of care cases;    2. All the mothers 100% (246/246) reported that the scan fostered a stronger bonding between expecting fathers and their baby. |
| **4.3 Health Economic Outcomes** | NI |

**JBI Qualitative Data Extraction Tool**

**Reviewer: XXX & XXX & XXX Date: 28^th^ February 2025**

**Author: Wanjiku et al. Year: 2018**

**Journal: BMC Health Services Research Record Number: 27**

| **1. Study Characteristics** | |
| --- | --- |
| **Item** | **Extracted Data** |
| 1.1 Author (Publication Year) | Wanjiku et al., 2018 |
| 1.2 Country | Kenya |
| 1.3 Location | 21 rural and under-resourced healthcare facilities |
| 1.4 Study Design | Cross-sectional study |
| 1.5 Sample Size | 33 |
|  | |
| **2. Population Characteristics (Healthcare Providers Receiving POCUS Training)** | |
| **Item** | **Extracted Data** |
| 2.1 Age | NI |
| 2.2 Gender | NI |
| 2.3 Professional Role | Clinical officers, nurses, and medical officers |
| 2.4 Work Experience (Duration) | NI |
| 2.5 Prior Ultrasound Training Experience | None (0/33) |
|  | |
| **3. Intervention Characteristics (According to ADDIE Training Model)** | |
| **Item** | **Extracted Data** |
| 3.1 Analysis | 1. Trainee Group:   Healthcare providers from 21 rural and under-resourced healthcare facilities in Kenya.   1. Needs Analysis: NI. 2. Baseline Survey:    1. Clinical designation.    2. Whether have any prior experience with ultrasound training (If so, what is the dates of the training and the outcomes);    3. Whether use ultrasound over the previous 3 months. 3. Impact on Instructional Design:   Since the trainees had no prior ultrasound experience and faced difficulties in accessing extended training, the program emphasized pre-program preparation with a multimedia manual. The content focused on applications relevant to rural healthcare (including obstetric ultrasounds). |
| 3.2 Design | 1. Training Objectives:    1. Knowledge goals: Understand the principles and applications of POCUS.    2. Skill goals: Be able to perform POCUS examinations independently and obtain good quality images for accurate diagnosis.    3. Attitude goals: Develop confidence in using POCUS in clinical practice and a positive attitude towards continuous learning and improvement in POCUS skills. 2. Training Methods:   The training combined self-study with hands-on training.   1. Training Content/Plan/Syllabus:   Trainees first studied a multimedia manual, the multimedia manual first provided basic knowledge. Then, they participated in a supervised hands-on training session in small groups, the hands-on training then allowed trainees to apply this knowledge in practice. The follow-up testing and refresher training further enhanced their skills. |
| 3.3 Development | 1. Instructors/Teachers:   The instructors were certified.   1. Teaching Aids:    1. Devices: Ultrasound machines.    2. Ultrasound model: Healthy volunteers (a male, a first-trimester pregnant female, and a second/third-trimester pregnant female) were used as models. 2. Teaching Materials:   The teaching materials included a multimedia manual. In 2013, it was revised to be more focused and include illustrations. In 2014, multimedia videos were embedded. |
| 3.4 Implementation | 1. Time:    1. The training program conducted since November 2014. The initial training is a 1-day hands-on training session.    2. The follow-up in-facility testing was scheduled 3-4 months after initial training.    3. Refresher training was provided 3-4 months after the in-facility assessment if needed. 2. Location/Setting:    1. The pre-training self-study could be done at the trainees' own facilities.    2. Follow-up in-facility testing and refresher training took place at the trainees' current healthcare facilities. 3. Participants:    1. Trainers: Certified instructors.    2. Trainees: Rural healthcare providers from 21 facilities. 4. Execution Process:   Since November 2014, to enroll in the programme, trainees first studied the multimedia manual and took an online test. Then, they attended the initial training, which is a 1-day supervised hands-on training in a small group. Then, they could use the ultrasound machines at their facilities for 3-4 months. After that, an in-facility follow-up testing included a written exam and an Observed Structured Clinical Exam (OSCE) conducted, those who did not pass were invited for a refresher training, which involved restudying the manual and hands-on practice. |
| 3.5 Evaluation | 1. Formative Evaluation:    1. Purpose: Assess trainees' progress during training and identify areas for improvement.    2. Methods/Tools: The online pre-training test and, a written exam and an observed structured clinical exam (OSCE).    3. Timing: Before the initial training (1 day hands-on training), during the 1-day training, and 3-4 months after initial training (in-facility testing). 2. Summative Evaluation:    1. Purpose: Evaluate the overall effectiveness of the training program on POCUS knowledge and skills    2. Methods/Tools: 3. A post-course OSCE to access the scanning ability. And a standardized, 30-question multiple choice question (MCQ) exam (which is same as the previous written exam) was used to access knowledge retention. 4. A survey on the use of POCUS in clinical practices. 5. A standardized scoring system (from 0 to 4) to access the image quality.    1. Timing: 6. 3-4 months after initial training for in-facility testing. 7. 3-4 months after the in-facility assessment for refresher training if needed. |
|  | |
| **4. Outcome Characteristics** | |
| **Item** | **Extracted Data** |
| **4.1 Training Outcomes** |  |
| 4.1.1 Diagnostic Accuracy | NI |
| 4.1.2 Knowledge Acquisition | 1. The passing score of the standardized 30-question multiple choice question (MCQ) exam was defined as 27/30 (> 90%), the pass rate of MCQ: 27.3% (9/33). 2. The average written test score (total score of 30) for all trainees increased from 68.2% (at initial training exam) to 73.6% (after in-facility practice). 3. Mean written exam scores related to number of training sessions (total score of 30)    1. Only initial training was conducted (n=15): 4. Prior score (Mean): 74.7%. 5. Present score (Mean): 71.1%. 6. Difference (Mean): 3.6% (*p*=0.33).    1. The initial training and one refresher session were conducted (n=8): 7. Prior training score (Mean): 61.2%. 8. Present score (Mean): 76.3%. 9. Difference (Mean): 15.1% (*p*=0.03).    1. The initial training and two refresher sessions were conducted (n=9): 10. Prior score (Mean): 64.2%. 11. Present score (Mean): 77.8%. 12. Difference (Mean): 13.6% (*p*<0.01). 13. Mean written exam scores related to time elapsed since the initial training     1. For those trained < 3 months (n=7): 14. Previous score (Mean): 72.9%. 15. Present score (Mean): 81.4%. 16. Difference (Mean): 8.5% (*p*=0.06);     1. For those trained > 6 months (n=17): 17. Previous score (Mean): 56.6%. 18. Present score (Mean): 64.7%. 19. Difference (Mean): 8.1% (*p*=0.08). |
| 4.1.3 Skills Acquisition | 1. OSCE image quality scores for obstetric ultrasound related to number of training sessions (Image quality was scored from 0-4):    1. Image quality scores (Mean): 2.95 (95% *CI*: 2.67-3.24).    2. Only initial training session (Mean): 2.73 (95% *CI*: 2.31-3.15).    3. The initial training and one refresher session were conducted (Mean): 2.95 (95% *CI*: 2.21-3.69);    4. The initial training and two refresher sessions were conducted (Mean): 3.33 (95% *CI*: 2.84-3.83). 2. Image quality scores for obstetric ultrasound related to time elapsed since the initial training:    1. <3 months (Mean): 3.64 (95% *CI*: 3.37-3.91)    2. >6 months (Mean): 2.74 (95% *CI*: 2.34-3.13) |
| 4.1.4 Knowledge Retention | 1. Mean written exam scores (total score of 30) for trainees that were required to obtain a 90% score for initial training (n=8) after all the training:    1. Previous score (Mean): 90%    2. Present score (Mean): 86.2%    3. Difference (Mean): 3.8% (*p*=0.34) |
| 4.1.5 Practical Application | 1. The frequency of clinical use of POCUS    1. 3 months before the final evaluation, 31 trainees completed the survey: 2. < 10 scans: 22.6% (7/31) 3. 10-20 scans: 38.7% (12/31) 4. > 20 scans: 38.7% (12/31)    1. 1 month before the final evaluation, 12 trainees completed the survey: 5. < 10 scans: 8.3% (1/12) 6. 10-20 scans: 50.0% (6/12) 7. > 20 scans: 41.7% (5/12) 8. Of the 31 trainees who completed the survey, 54.8% (17/31) performed the 2nd/3rd trimester pregnancy scan, which is the most frequent clinical use, and then 25.8% (8/31) performed1st trimester pregnancy scan. |
| **4.2 Maternal and Neonatal Health Outcomes** (Including Physiological and Psychological Outcomes) | NI |
| **4.3 Health Economic Outcomes** | NI |
